# Microfluidic Photocatalytic Ring Expansion of Sulfonium Salts for the Synthesis of Cyclic Sulfides

**DOI:** 10.1021/acscatal.5c01231

**Published:** 2025-04-07

**Authors:** Jorge Humbrías-Martín, José J. Garrido-González, Katy Medrano-Uribe, Giorgio Pelosi, Loris Laze, Luca Dell’Amico

**Affiliations:** † Department of Chemical Sciences, University of Padova, Via Francesco Marzolo 1, 35131 Padova, Italy; ‡ Department of Chemistry, Life Sciences and Environmental Sustain-ability, University of Parma, Parco Area Delle Science 17, 43124 Parma, Italy; § Instituto de Síntesis Orgánica (ISO) and Departamento de Química Orgánica, Universidad de Alicante, 03080 Alicante, Spain

**Keywords:** photocatalysis, flow chemistry, sulfonium salts, sulfides, photoredox catalysis

## Abstract

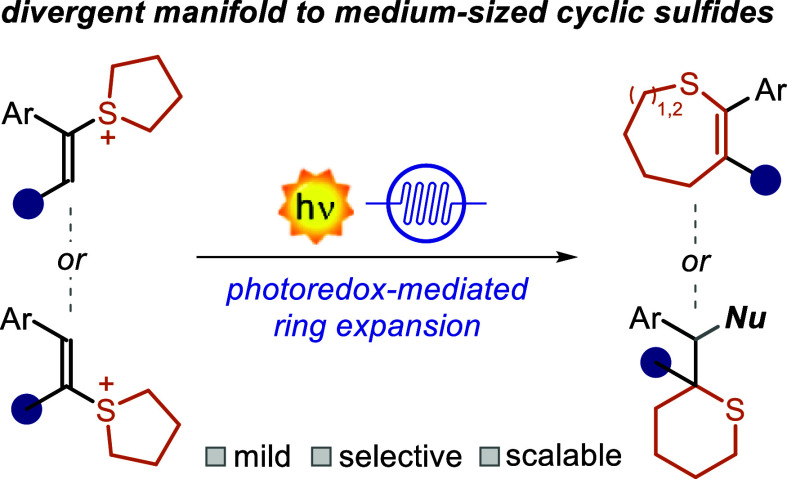

Cyclic sulfides are relevant building blocks in medicinal and synthetic chemistry, with applications ranging from drug discovery to materials science. However, the synthesis of medium-sized cyclic sulfides (6–8-membered rings) remains largely underdeveloped. Herein, we report a photocatalytic ring-expansion strategy for sulfonium salts, granting access to six-, seven-, and eight-membered cyclic sulfides with very high regio- and diastereocontrol. The implementation of the method under continuous flow was key to increasing the efficiency and minimizing product decomposition. Mechanistic investigations revealed the formation of benzylic radicals and carbocation intermediates that control the high regio- and diastereoselectivity observed. Finally, the synthetic utility of this approach was demonstrated in the synthesis of cyclic sulfoxides and sulfones, which can be easily obtained from the corresponding sulfide products.

## Introduction

Sulfur (S)-containing molecules play a pivotal role in medicinal and synthetic chemistry. The S atom infers unique electronic and structural properties, enabling the precise modulation of biological activity.^1−3^ An important class of sulfur-containing molecules is sulfides where the S atom is bonded to two carbon units. In particular, six and seven-membered cyclic sulfides are an important class of molecules, counting several FDA-approved drugs.^3,4^ In spite of their relevance, synthetic approaches for the construction of cyclic sulfides remain underdeveloped,^5−7^ especially when compared to the extensive advancements in the synthesis of S(IV)- or S(VI)-containing molecules, including sulfoxides, sulfones, and sulfonamides.^6,8−13^ Moreover, the construction of cyclic sulfides via ring expansion of a preexisting functionality presents additional hurdles, as the formation of larger rings is often disfavored due to entropic factors and ring strain.^14−16^ Thus, developing new methodologies to address these challenges is of paramount importance. Sulfonium salts (SSs) have emerged as key intermediates in organic synthesis, owing to their versatile reactivity and ability to serve as precursors for a variety of functional groups.^17,18^ These salts are renowned for their stability and ease of handling. Over the past few decades, SSs have been extensively employed in various synthetic transformations, including alkylation, arylation, and the generation of ylides (e.g., the Corey–Chaykovsky reaction).^17^

**Scheme 1:**
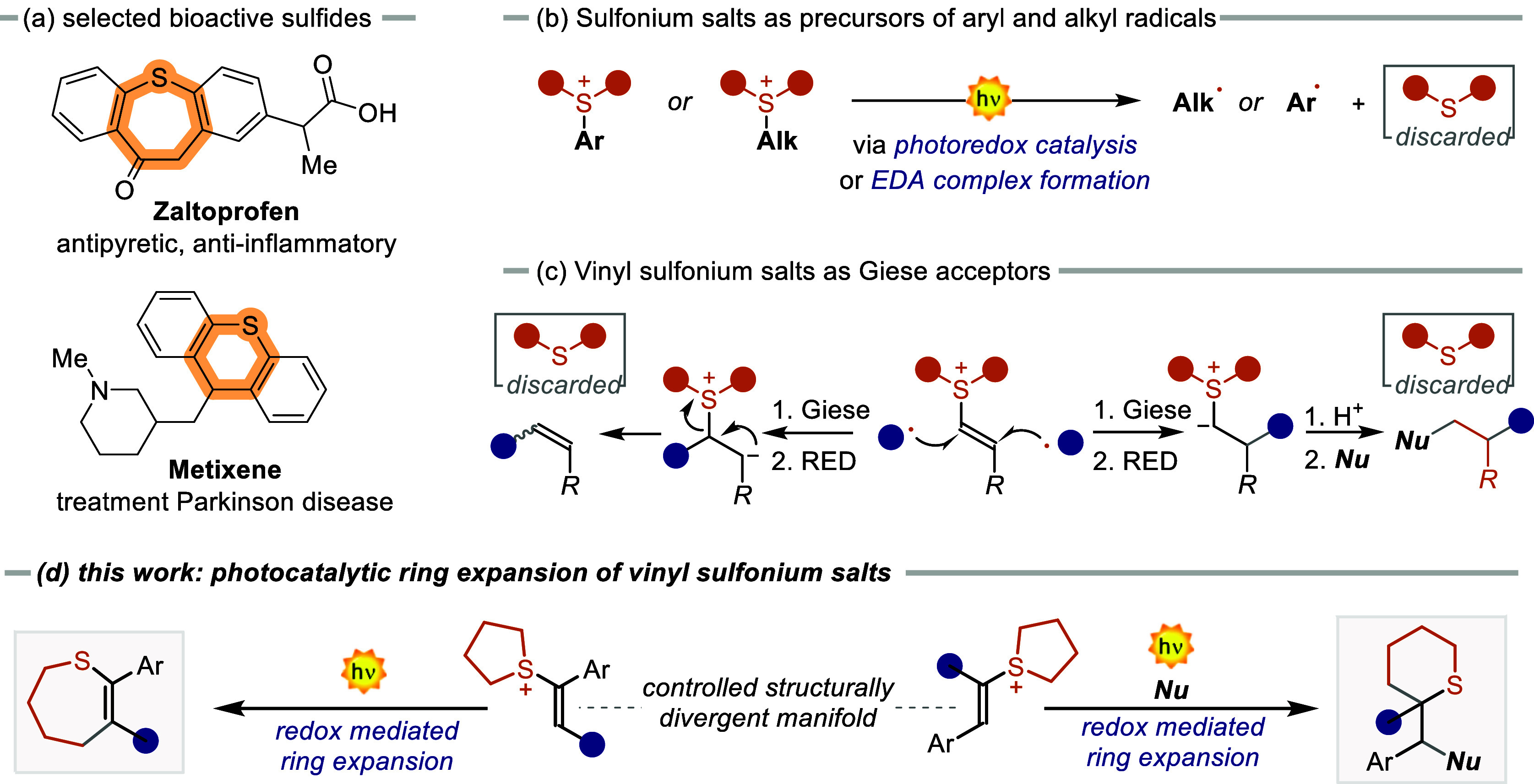
Relevance of Sulfides and Photocatalytic Application of SSs

Their utility extends beyond traditional organic synthesis to areas such as medicinal chemistry and materials science, making them invaluable building blocks in the development of pharmaceuticals and materials.^1−3^ In contrast to alkyl and aryl SSs,^19−26^ the photochemistry of alkenyl or vinyl SSs has been less explored. In 2020, Wang and co-workers reported a metal-free radical addition to vinyl SSs for the synthesis of olefins (Scheme 1c, left).^27^ More recently, Silvi and co-workers have shown that upon radical addition, the sulfonium moiety can be replaced by diverse functional groups granting access to formally polarity-mismatched products (Scheme 1c, right).^28^ Nevertheless, all of these synthetic strategies using SSs do not incorporate the sulfur atom into the final product. In contrast, in 2024 Yang and co-workers have reported a metal photocatalyzed ring opening of sulfonium salts via selective C–S bond cleavage, expanding the synthetic utility of SSs in the synthesis of organic sulfides.^29^ In this article, we describe a novel photocatalytic strategy for the cyclic expansion of SSs, where the sulfur atom is included into the final cyclic product. Our approach utilizes an organic photocatalyst (PC) to mediate selective sulfur-containing ring expansion, providing access to various cyclic sulfides with excellent regio- and stereocontrol. This method broadens the scope of accessible cyclic sulfides and offers a new paradigm for synthesizing valuable sulfur-containing compounds.

## Methods

We began our investigation with SS **1** using the organic PC **4DPAIPN** (5 mol %). Under these conditions, only traces of product **2** were observed, along with a 5% conversion of starting material **1** (Table 1, entry 1). The addition of a base (NEt3) resulted in improved performances (28% NMR yield), indicating its key role in product formation (vide infra). Moving to 2,6-lutidine improved the mass balance, delivering the product in 33% NMR yield (entry 3). Unfortunately, the use of other PCs, as well as a diverse PC’s loading did not improve this result (see Supporting Information, Section S3). We also realized that the yield did not improve when the reaction time was extended (entry 4) due to the decomposition of product **2**. In fact, **2** can be easily oxidized by **4DPAIPN**, leading to a complex degradation pattern. To circumvent the problem and exclude **2** from undesired overirradiation, we performed the reaction in flow.^30−33^ Here, with 5 min of residence time, we obtain an improved mass balance and 46% NMR yield of **2** (entry 5). Extending the residence time to 10 min resulted in optimal conditions with 77% yield of the isolated product. When in the absence of light or PC, the reaction does not proceed with a complete recovery of the starting SS **1** (entry 7 and Supporting Information, Section S3).

**Table 1:**
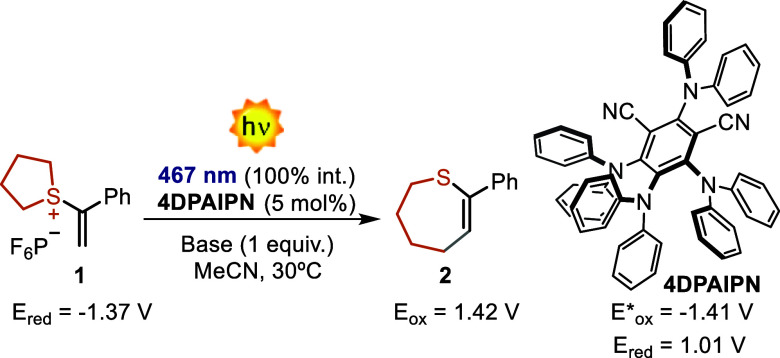
Optimization of the Reaction

entry^a^	cond	reaction time	base	conv. (%)	NMR yield^b^ (%)
1	batch	4 h		5	traces
2	batch	4 h	Et3N	98	28
3	batch	4 h	2,6-lutidine	58	33
4	batch	6 h	2,6-lutidine	73	28
5	flow	5 m	2,6-lutidine	54	46
6	**flow**	**10 mi**	2,6-lutidine	**>98**	(82)77
7^c^	batch	10 mi	2,6-lutidine		

a Reaction conditions: 1 (0.4 mmol), 4DPAIPN (5 mol %), base (0.4 mmol) in MeCN (4 mL), degassed with argon and irradiated with a Kessil lamp at room temperature.

b NMR yield measured by the addition of 0.05 equiv of dibromomethane as the internal standard.

c No photocatalyst.

With the optimized conditions in hand, we evaluated the generality of the developed ring expansion protocol (Table 2). The reaction proceeded well with both electron-poor and electron-rich aryl rings, affording the final 7-membered cyclic sulfides in yields up to 83% (**2**, **15–26**). The incorporation of an additional aryl ring in the double bond was also tolerated, affording product **22** in 40% yield. Interestingly, when 1,2-dihydronaphthalene SS was employed, we were able to obtain the heterocyclic compound **23** in 62% yield, which came from the aromatization of compound **24** via an oxidation manifold (see Supporting Information, Section S5). Moreover, we were also able to extend the methodology to six-membered cyclic SS, affording the elusive thiacyclooctene derivative **25**. Lastly, we could increase the scale of the reaction up to the 1 mmol scale without a significant decrease in the isolated yield of **2**.

**Scheme 2:**
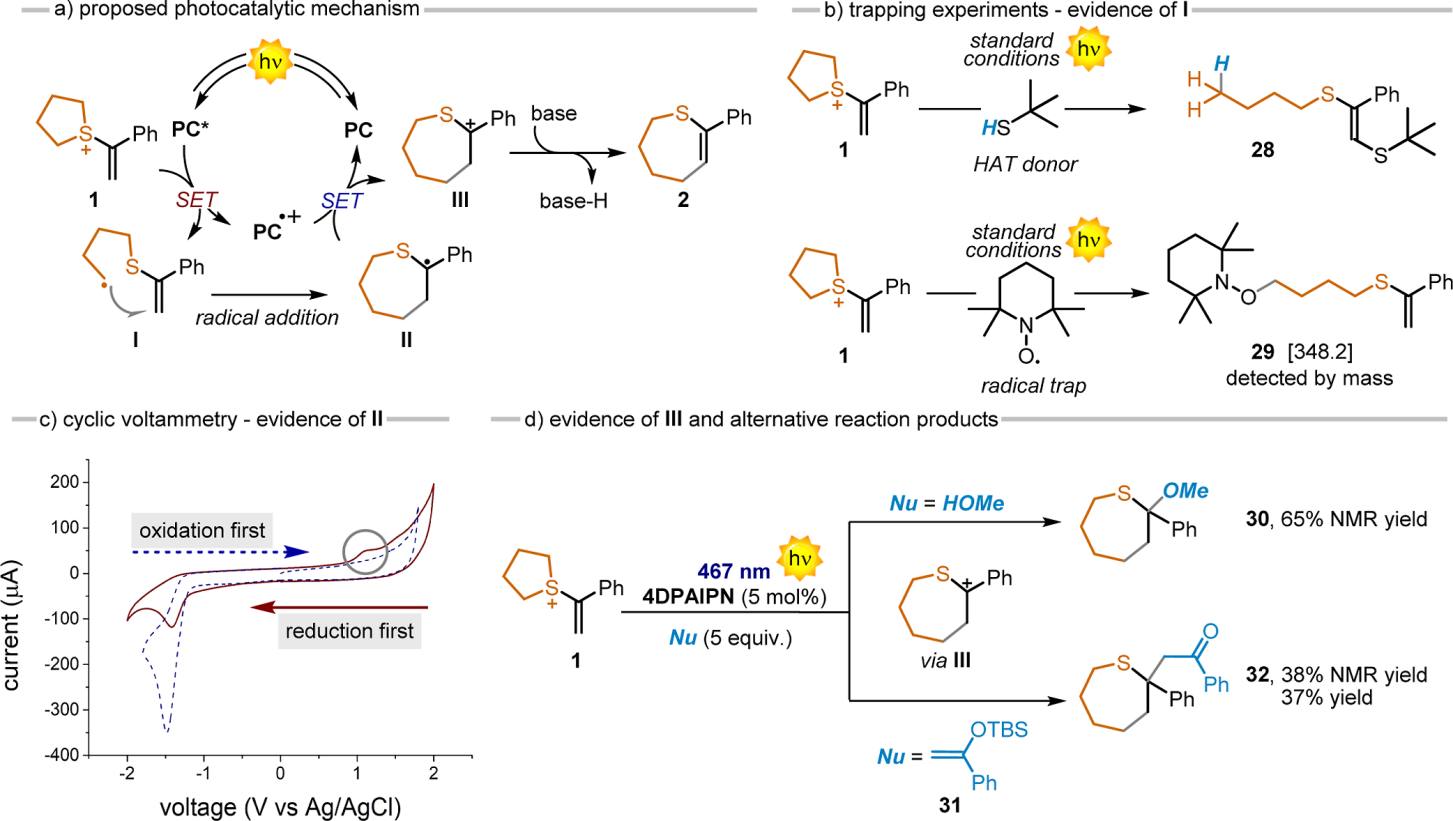
Mechanistic Investigations

Next, we investigated the reaction mechanism of the developed ring-expansion protocol (Scheme 2). We assumed that upon light excitation, the PC undergoes oxidative quenching, reducing the starting SS while delivering radical **I** and the PC radical cation (PC^•+^). Subsequently, a fast intramolecular radical trapping event delivers the more stable radical **II**, which is later oxidized by PC^•+^ with the formation of the stabilized carbocation **III** and the regeneration of the ground state PC. At this stage, the base is essential to promote the elimination process by generating the double bond and the final sulfide **2**. To support this mechanism, we gained several experimental evidence. First, the PC* is promptly quenched by the SS (see Supporting Information, Section S7). When performing the reaction in the presence of a HAT donor, the open sulfide **28** was obtained, supposedly derived from the radical intermediate **I**. When the reaction mixture was in the presence of TEMPO, the reaction did not proceed, and product **29** was observed by mass analysis. This data agree with the formation of radical **II**. When cyclic voltammetry of **1** was performed starting from cathodic current to anodic current, a peak at 1.12 V was observed and assigned to the oxidation of radical **II** to the carbocation **III**. Finally, to further prove the formation of carbocation **III**, we performed the reaction in the presence of diverse nucleophiles. When in the presence of MeOH, the trapping product **30** was observed by both mass and NMR. Isolation of **30** was not achieved due to its low chemical stability. We thus used a C-centered nucleophile, such as **31**, affording product **32** in a 37% yield, supporting the proposed reaction scheme.

Based on our discoveries, we further investigated the role of the aryl ring that stabilizes the formation of intermediate **II**, allowing the ring expansion. In the absence of an aryl group, no formation of the corresponding sulfide **27** was observed, with a complete recovery of **14** (Table 3). Instead, when the aryl group was installed in the β-position, the reaction proceeded smoothly, this time with the formation of hydroxylated cyclic compound **45**. The presence of **45** can be explained with the formation of a benzylic carbocation, that originates from the radical addition at the vicinal α-position, leading to an alternative pathway for the synthesis of α-functionalized cyclic sulphides.^34^ Intrigued by this alternative reactivity, we decided to evaluate its generality while shifting the aryl ring to the double-bond β-position. In fact, we speculated that the presence of an aryl group at the remote carbon atom would have favored the α-addition by generating a more stable benzylic radical. We thus synthesized a series of β-substituted SSs. We subjected these molecules to the developed reaction conditions with the addition of 5 equiv of H_2_O as the nucleophile. Interestingly, the process delivered a series of valuable α-hydroxymethyl cyclic sulfides in moderate to good yields (up to 75%) and excellent diastereocontrol (up to >20:1). The relative stereochemistry was inferred by structural correlation with the known compound **45** (see Supporting Information, Section S8).^35^ Taking advantage of the flow conditions, we could again scale up the reaction to 1 mmol, isolating **45** in 74% yield. While other SSs bearing ED- and EWGs furnished the corresponding hydroxy sulfides in good yields (up to 71%, **46–56**) and exquisite diastereocontrol.

**Table 2:**
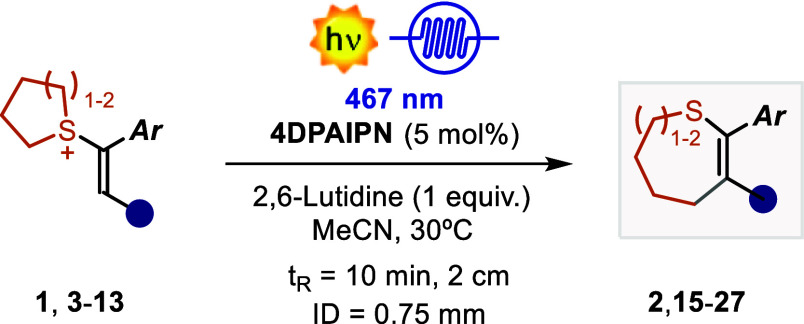

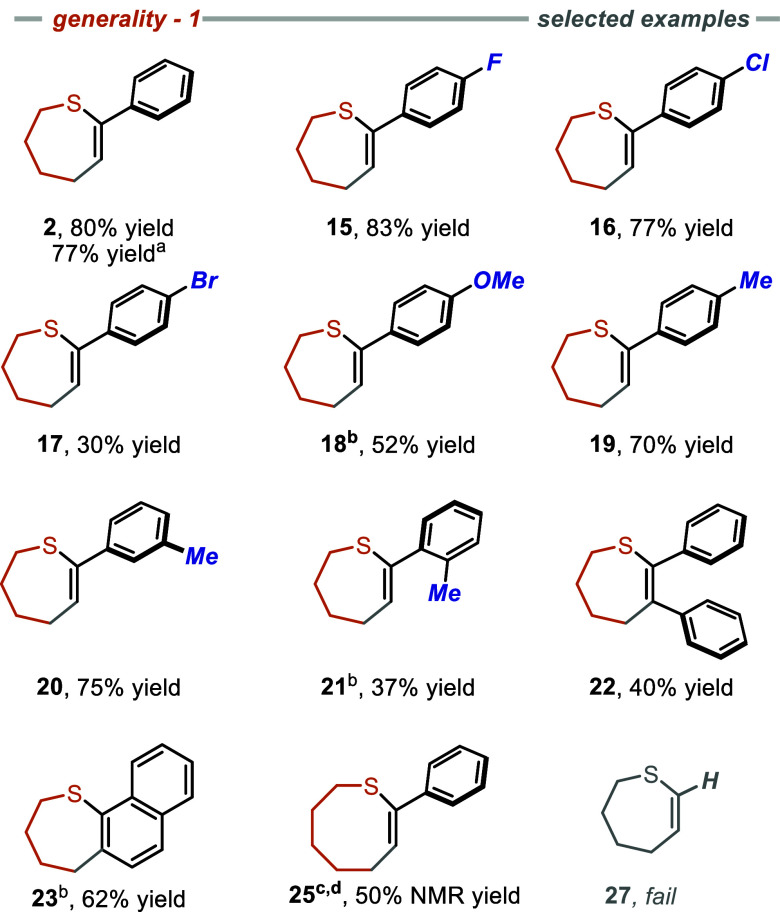
Structural Generality of Geminal SSs

a Yields are reported for 0.3 mmol scale (see Supporting Information, Section S5). One mmol scale (see Supporting Information, Section S3).

b Performed with a *t*_R_ of 30 min.

c 3DPAFBN was used as a photocatalyst.

d The compound was isolated in 31% yield due to low stability over silica-gel.

To further explore the generality of this ring expansion protocol, we investigated alternative nucleophiles. Remarkably, alcohols, aromatic amines, and Cl^–^ also delivered the corresponding product in yields of up to 73% (**57**–**60**). Carbon-centered nucleophiles such as silanes as well as silyl enol ether (SEE) resulted in more challenging substrates, delivering the corresponding products in 23% and 25% yield, respectively (**61–62**). In these reactions, oxidation of the starting nucleophile is a competitive pathway that can explain the inferior yields.

**Scheme 3:**
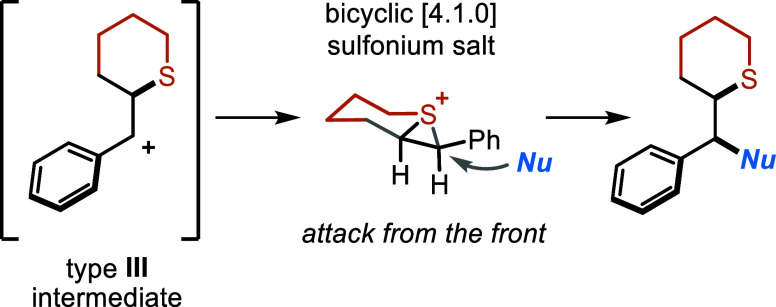
Origin of the Diastereoselectivity

It is worth noting that the process yields 2-hydroxysulfide building blocks with the formation of one new C–S bond along with a new C–O, C–N, C–Cl, or C–C bond in a single strike and with very high dr. To understand the reasons behind the high diastereocontrol, we performed DFT calculations that revealed the diastereoselective formation of a transient bicyclic [4.1.0] sulfonium salt (see Supporting Information, Section S9). The intermediate evolves from the intramolecular addition of the sulfide to the benzylic carbocation and governs the diastereoselectivity of the process, directing the attack of the incoming nucleophile (Nu) from the opposite side of the sulfonium atom (Scheme 3).

**Scheme 4:**
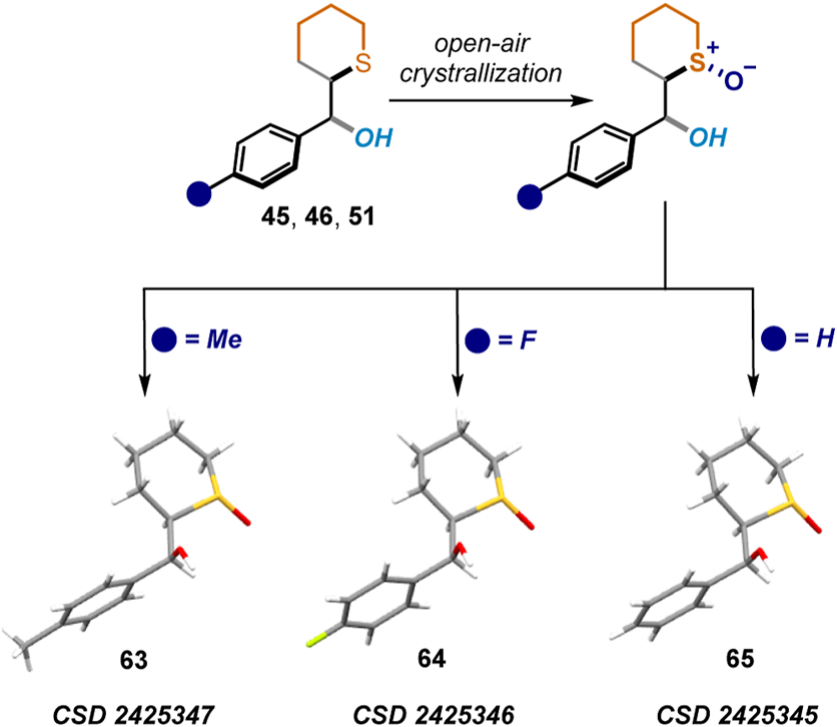
X-ray from Sulfoxides

**Scheme 5:**
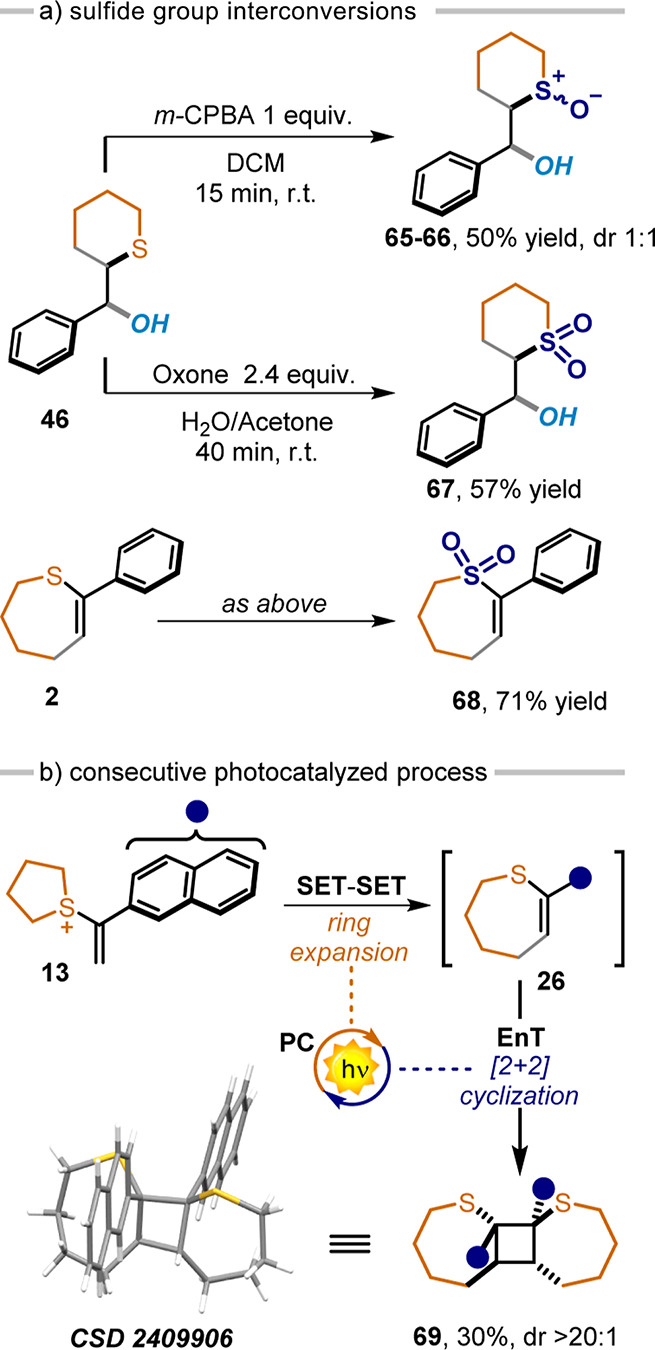
Manipulations

Interestingly, further attempts to recrystallize the α-hydroxymethyl cyclic sulfides **45**, **46**, and **51** led to the oxidation to the corresponding sulfoxides (Scheme 4). These structures further confirmed the relative configuration assigned. Given the importance and high synthetical value of sulfoxide and sulfone moieties,^9^ we performed selective oxidations of **45** to the corresponding sulfoxides **65** and **66** and sulfone **67** with 50% and 57% yield, respectively (Scheme 5a). The same oxidation procedure was applied to **2**, affording the corresponding sulfone **68** in 71% yield (Scheme 5a). Furthermore, by installing a 2-naphthyl moiety within **17**, we could access the triplet energy of **26** (coming from the ring expansion process), using the same PC **4DPAIPN** as a triplet sensitizer.

**Table 3:**
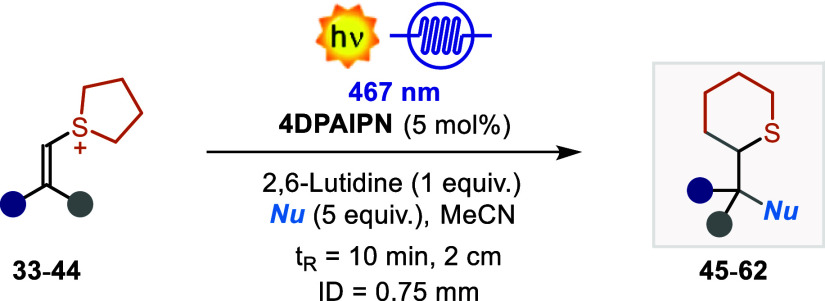

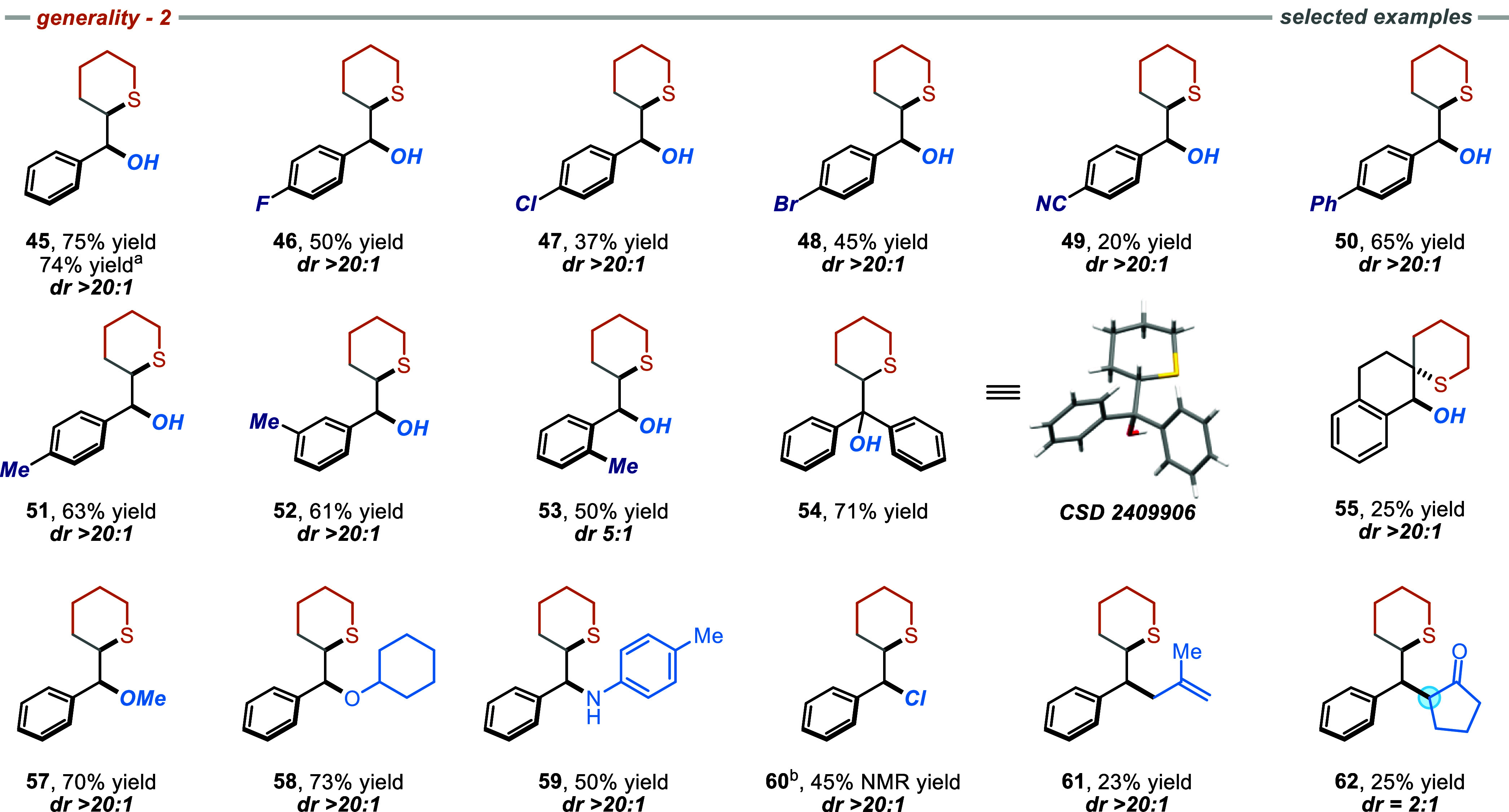
Structural Generality of Trans SSs

a Yields are reported for 0.3 mmol scale (see Supporting Information, Section S5). One mmol scale (see Supporting Information, Section S3).

b The product hydrolyzed to **45** after purification. ID = internal diameter.

Such a consecutive photocatalytic cascade process granted access to the [2 + 2] adduct **69** in 30% yield as a single diastereoisomer in an iterative SET-SET-EnT photocatalytic manifold (Scheme 5b).

## Conclusions

In conclusion, we have developed a visible-light photocatalyzed ring expansion process that allows easy access to functionalized cyclic sulfides of different sizes (6,7, and 8-member rings, 32 examples with up to >20:1 dr and 83% yield). This microfluidic method expands the existing synthetic toolkit for sulfur-containing molecules, offering a versatile and scalable protocol with potential applications in pharmaceuticals and materials science.

## Supporting Information Available

General information; experimental setup for light irradiation; reaction optimization; synthesis and characterization of the starting materials; synthesis and characterization of products 2, 15–26, and 45–62; product derivatizations; mechanistic investigations; assignation of the relative configuration of 45 and 55; DFT calculations; X-ray diffraction analysis; and NMR spectra (PDF)

## Abbreviations

HAT: hydrogen atom transfer
SET: single-electron transfer
SS: sulfonium salt
FDA: Food and Drug Administration
PC: photocatalyst
ID: internal diameter
