# Supplementary material for: Microfluidic Photocatalytic Ring Expansion of Sulfonium Salts for the Synthesis of Cyclic Sulfides
Source: ACS Catal. 2025 Apr 7;15(8):6507–13. doi: 10.1021/acscatal.5c01231 (PMC12012829; doi:10.1021/acscatal.5c01231)
Supplement: Supplementary file 1 — cs5c01231_si_001.pdf [file cs5c01231_si_001.pdf]

SUPPORTING INFORMATION

Microfluidic Photocatalytic Ring Expansion of Sulfonium Salts for the Synthesis of Cyclic Sulfides.

Jorge Humbrias-Martin, Jose J. Garrido-Gonzalez, Katy Medrano-Uribe, Giorgio Pelosi, Loris Laze, Luca Dell'Amico*

Dr. J. Humbrias-Martin, Dr. J. J. Garrido-Gonzalez, Dr. K. Medrano-Uribe, Prof. L. Dell'Amico

Department of Chemical Sciences, University of Padova.

Via Francesco Marzolo 1, 35131 Padova (Italy).

Prof. G. Pelosi

Department of Chemistry, Life Sciences and Environmental Sustain-ability, University of Parma.

Parco Area delle Science 17, 43124 Parma (Italy).

L. Laze

Instituto de Síntesis Orgánica (ISO) and Departamento de Química Orgánica, Universidad de Alicante, 03080 Alicante (Spain).

Corresponding Author: Luca Dell'Amico - Department of Chemical Sciences, Univer-sity of Padova, Via Francesco Marzolo 1, 35131, Padova (Ita-ly); email: luca.dellamico@unipd.it

Table of Contents

1. General information 1
2. Experimental setup for light irradiation 3
Kessil lamps 3
Batch reaction setup with Kessil lamp 3
Flow reaction setup with Kessil lamp 4
Construction of homemade flow reactors. 4
3. Reaction optimization 6
General procedure A for the photocatalytic ring expansion of sulfonium salts in batch 6
Photocatalytic ring expansion of Sulfonium Salts. Screening in batch conditions 6
Analysis of the reaction profile 8
Photocatalytic ring expansion of Sulfonium Salts. Screening in flow conditions 9
Steady state analysis 10
4. Synthesis and characterization of the starting materials (PC and sulfonium salts) 11
General procedure B for the preparation of geminal SSs 11
General procedure C for the preparation of *trans* SSs 11
Characterization of sulfonium salts 1, 3-13, 33-44 12
5. Synthesis and characterization of products 2, 15-26, 45-63 22
General procedure D for the photocatalytic ring expansion of sulfonium salts in flow 22
Characterization of products 2, 15-26, 45-63 22
6. Product derivatizations. Synthesis and characterization of 64-68 33
7. Mechanistic investigations 35
Redox properties of starting materials and products 35
Radical trapping: evidence of intermediate I 36
Alternative reaction products: evidence of intermediate III 37
Quenching experiments: Stern-Volmer 38
8. Assignation of the relative configuration of 45 and 55 39
9. DFT calculations 41
10. X-ray diffraction analysis 44
X-ray crystallographic data for compound 55 45
X-ray crystallographic data for compound 68 46
11. References 47
12. NMR spectra 49

1. General information

Reagents, solvents and experimental conditions

All reactions were carried out in anhydrous solvents purchased from commercial suppliers over molecular sieves in a sealed bottle which were used without further purification. Chemicals were purchased from commercial sources (Sigma–Aldrich, Fluorochem or TCI) and used without further purification. Organic solvents were purchased from Sigma–Aldrich. Reactions were monitored by thin-layer chromatography on silica gel 60F254, and/or by ^{1}H and ^{19}F NMR spectroscopy.

Thin-layer chromatography (TLC) analysis was performed on pre-coated Merck TLC plates (silica gel 60G F254, 0.25 mm). Visualization of the developed purification was performed by checking UV absorbance (254 and/or >320 nm) as well as with different stain solutions (aqueous ceric ammonium molybdate, potassium permanganate, phosphomolybdic acid solutions, among others). Chromatographic purification of the products was accomplished using flash chromatography on silica gel (SiO_2, pore size 60 Å, 230-400 mesh particle size and 40-63 μm particle size) purchased from Sigma-Aldrich, with the indicated solvent system according to the standard techniques, or with pre-coated Merck preparative TLC plates (silica gel 60G F254, 20x20 cm). Organic solutions were concentrated under reduced pressure on a Heidolph rotary evaporator.

Flow reactions were performed using a Syrris Atlas Syringe Pump equipped either with a 250 or 500 μL syringe. FEP tubing (natural), 1/16'' OD x 0.75 mm ID and 3.2 mm OD x 1.6 mm ID were purchased from BGB and were used to create the homemade flow reactors.

The Kessil lamps PR160L (50W) were purchased from Kessil webpage: https://www.kessil.com/products/science_PR160L.php.

Analytical techniques

NMR spectra were collected on a Bruker AC-300 spectrometer fitted with a Bruker PABBO BB/19F1H/D probe head, Bruker 200 equipped with a QNP probehead, Bruker 400 Avance III HD spectrometer equipped with a BBI-z grad probehead, Bruker 500 Avance III equipped with a BBI-ATM-z grad probehead, Bruker Neo 600 equipped with a Prodigy probehead, Bruker UltraShield 400 or Bruker UltraShield 500 operating at the denoted spectrometer frequency given in MHz for the specified nucleus. Reported coupling constants and chemicals shifts were based on a first order analysis. The internal reference for ^{1}H NMR was the residual peak of $CDCl_3$ (7.26 ppm) or CD_3CN (1.96 ppm); and for ^{13}C NMR was the residual peak of $CDCl_3$ (77.16 ppm) or CD_3CN (1.79 ppm).[1] All coupling constants (J) are reported in Hz with the following abbreviations: s = singlet, d = doublet, dd = double doublet, t = triplet, dt = double triplet, q = quadruplet, m = multiplet, br = broad.

Steady-state absorption spectroscopy

Steady-state absorption spectroscopy studies were performed at room temperature on a Varian Cary 50 UV-vis; 10 mm path length Hellma Analytics 100-10-40 QS quartz cuvettes were used.

Steady-state fluorescence spectroscopy

Steady-state emission spectroscopy studies were performed at room temperature on a Varian Cary Eclipse Fluorescence spectrophotometer; 10 mm path length Hellma Analytics 117-10-40.100F QS quartz cuvettes were used.

Electrochemical characterization

The electrochemical characterizations were carried out in acetonitrile (MeCN)/0.1 M tetrabutylammonium hexafluorophosphate ($TBAPF_6$) at room temperature, on an BASi EC Epsilon potentiostat-galvanostat in a glass cell. A typical three-electrode cell was employed, which was composed of a glassy carbon (GC) working electrode (3 mm diameter), a platinum electrode as counter electrode and an Ag/AgCl electrode as reference electrode. The glass electrochemical cell was kept closed with a stopper annexed to the potentiostat. Oxygen was removed by purging the solvent with high-purity Nitrogen (N_2), introduced from a line into the cell by means of a needle. The potential of ferrocenium/ferrocene (Fc+ /Fc) couple was used as external reference system to calibrate the potentiostat. All the results were subsequently converted in V vs SCE, in agreement with the value reported in literature [E1/2(Fc+ /Fc) = +0.395 V vs SCE]. The IR compensation implemented within the potentiostat was used, and every effort was made throughout the experiments to minimize the resistance of the solution. The full electrochemical reversibility of the voltammetric wave of ferrocene was taken as an indicator of the absence of uncompensated resistance effects. The GC electrode was polished before any measurement with diamond paste, carefully rinsed with de-ionized water, ethanol, acetone and ultrasonically rinsed with a methanol/ethanol/acetone 1:1:1 (v/v) mixture for 5 minutes. After each series of CV experiments, the electrochemical cell was carefully rinsed with ethanol, acetone and deionized water; afterwards, the cell and the magnetic stirrer were sonicated for 5-10 min with acetone.

X-ray diffraction analysis

Single crystal X-ray diffraction analyses were carried out with a Bruker D8Venture diffractometer equipped with a kappa goniometer and an Oxford cryosystem. Microfocused MoKα radiation (λ = 0.71073) and CuKα radiation (λ = 1.54178) were used as the X-ray source and Lorentz polarization and absorption correction were applied through the SADABS[2a] procedure. The phase problem was solved by direct methods and the structures were refined by full-matrix least-squares on all F2 using SHELXL,[2b-c] as implemented in the OLEX2[2d] suite of programs. The structure drawings were obtained using ORTEPIII[2e] and Mercury.[2f]

Mass Spectra

Nominal mass was acquired with Waters Acquity UPC2-MS (Ultraperformance Convergence Chromatography) analysis, employing an achiral stationary phase column.

High-resolution mass spectrometry (HRMS) analyses were performed on MicroTOF-Q Bruker (ESI) and a GC Thermo Scientific Trace 1300 GC unit coupled to an APPI MasCom source mounted on a Thermo Scientific Exactive Plus EMR mass unit (Orbitrap FT-HRMS analyzer) or on a Xevo G2-XS QToF.

2. Experimental setup for light irradiation

Kessil lamps

In Figure S1 the emission spectra of Kessil LED PR160L lights are reported. The image can be found in the Kessil website (more info at: https://www.kessil.com/products/science_PR160L.php).

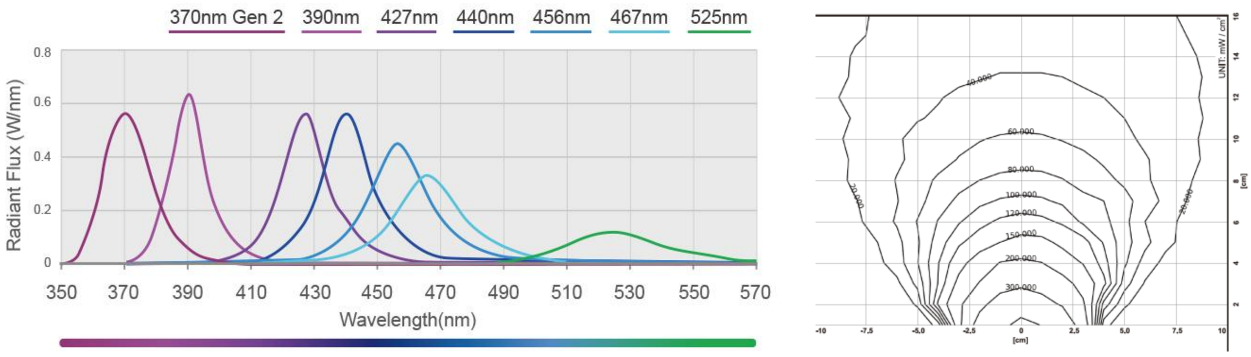

Figure S1. *Emission spectra of Kessil lights purchased by Kessil (left) and emission profile showing the lines of homogeneous irradiance provided by the producer (right).*

Batch reaction setup with Kessil lamp

Batch reaction setup was performed under Kessil LED PR160L light irradiation, using 390 (52W max.), 427 or 467 (45W max.) nm. The reaction mixture was placed into a 10 mL Schlenk tube (internal diameter, ID =1.2 cm) equipped with a glass stopper, sealed with high-vacuum grease, and was degassed through repeated freeze-pump-thaw cycles until no gas bubbles evolved upon thawing (usually 3 cycles). In the last thawing, Ar was introduced to keep an inert atmosphere. The Schlenk tube was placed at 6 cm from the light source and the reaction was stirred vigorously. A maximum of three reaction vessels were irradiated at the same time placing them following the lines of homogeneous irradiance provided by the producer. To maintain a stable reaction temperature, one fan was placed above the irradiated Schlenk tubes.

Flow reaction setup with Kessil lamp

Flow reaction setup with Kessil LED PR160L Figure S2 shows the general setup of a flow reaction performed under Kessil LED PR160L light irradiation (427 and 467 nm, 45W max.). The flow reactor was connected to the syringe pump using the FEP tube (ID = 0.75 or 1.6 mm), and it was placed at 2 cm from the light source. The system was filled with the solvent used in the reaction before every experiment. The reaction mixture was introduced into an 8-mL screw-cap vial equipped with a Teflon septum and was degassed for 30 seconds with a ballon filled with Ar, which was kept during the reaction progress. The reaction vial was connected to the syringe pump using FEP tube (ID = 0.75 mm). 4-mL vials were used to collect the fractions, which were equipped with a balloon filled with Ar. When the syringe pump is set into a certain flow rate, the Kessil lamp is turned on. Several fractions from the reaction are collected into 4-mL vials, which were equipped with an Ar ballon. To maintain a stable reaction temperature one fan was placed above the irradiated flow reactor and Kessil lamp.

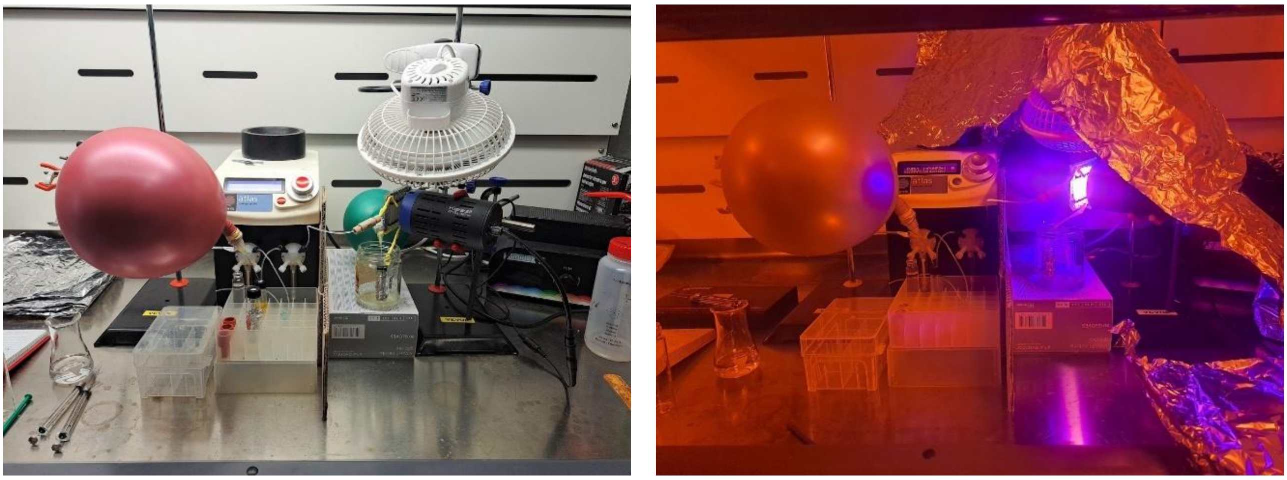

Figure S2. *Flow reaction setup with Kessil LED PR160*

Construction of homemade flow reactors.

Several homemade flow reactors were prepared for these investigations. In a general method, the tube is attached to a plastic matrix (with or without aluminium foil behind to reflect the light) using transparent nylon cable ties. Reactors with different lengths and diameters were prepared (150 μL, 300 μL and 600 μL), fixed in a 5x5cm square (maximum). In this work, two different scale up approaches were studied: sizing-up the length or the internal diameter of the tube.[3] The next equation presented was used to calculate the reactor's volume:

$$V = \frac{\pi}{4} \cdot N \cdot L \cdot D^2$$

Where V is the volume of the reactor (in mL), N is the number of tubes (in this case is 1), L is the length of the tube (in cm), and D is the internal diameter of the tube (in cm).

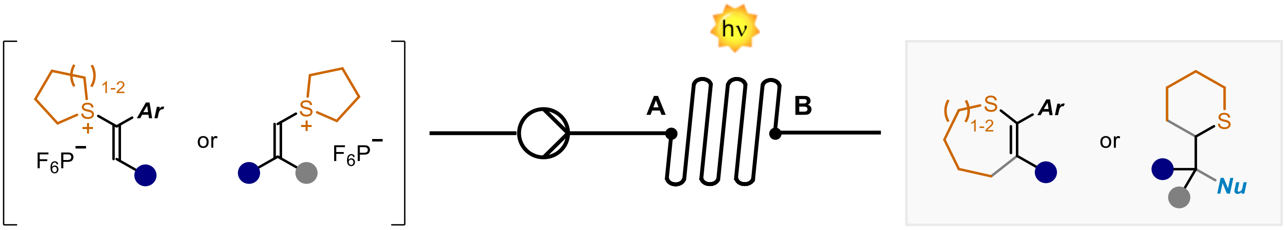

$L_{(A\text{-}B)}$ = 32 cm (ID = 0.75 mm); V_R = 150 μL
$L_{(A\text{-}B)}$ = 64 cm (ID = 0.75 mm); V_R = 300 μL
$L_{(A\text{-}B)}$ = 64 cm (ID = 1.60 mm); V_R = 600 μL

Figure S3. *Schematic figure of flow reactors and general information about their length and volume.*

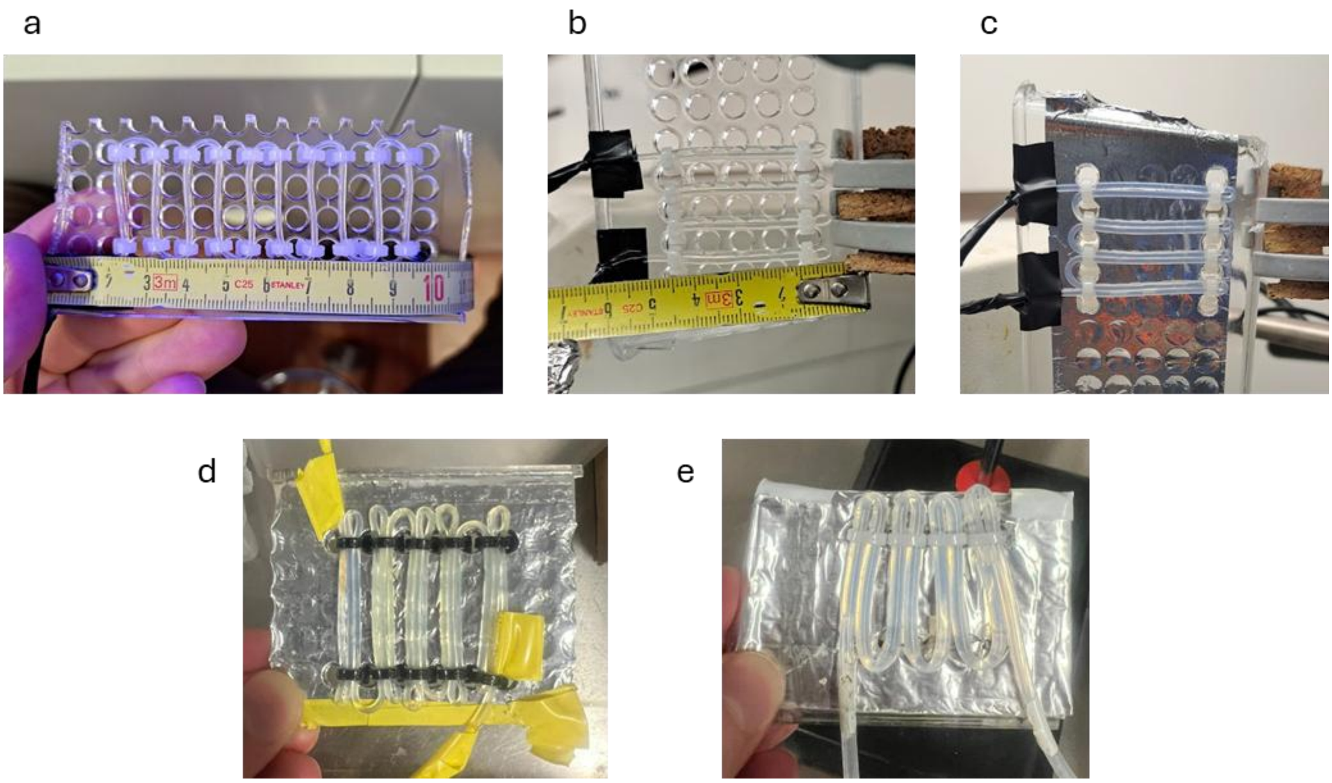

Figure S4. *Homemade flow reactors studied in this work: 150 μL (a, b and c), 300 μL (d) and 600 μL (e).*

3. Reaction optimization

General procedure A for the photocatalytic ring expansion of sulfonium salts in batch

A 10 mL Schlenk tube (internal diameter, ID =1.2 cm) was charged with the sulfonium salt substrate (0.1 mmol, 1 eq.), **4DPAIPN** (5 mol%), 2,6-lutidine (1 eq.) and filled with Argon. Then, dry CH_3CN (0.1 M) was added after degassed by sparging Argon for ten minutes. The tube was closed with a glass stopper, sealed with high-vacuum grease, and was degassed through repeated freeze-pump-thaw cycles until no gas bubbles evolved upon thawing (usually 3 cycles). In the last thawing, Ar was introduced to keep an inert atmosphere. The Schlenk tube was placed at 6 cm from the light source and the reaction was stirred vigorously. NMR yield was determined using dibromomethane (0.5 equiv.) as internal standard, and later the product was purified from the reaction mixture by column chromatography on silica gel (using cerium molybdate to stain the TLC).

Note: in the case of trans sulfonium salts, H_2O or the nucleophile (5 eq.) was added to the reaction mixture. Normal CH_3CN can be used when using H_2O as nucleophile, otherwise CH_3CN was dried over CaH_2.

Photocatalytic ring expansion of Sulfonium Salts. Screening in batch conditions

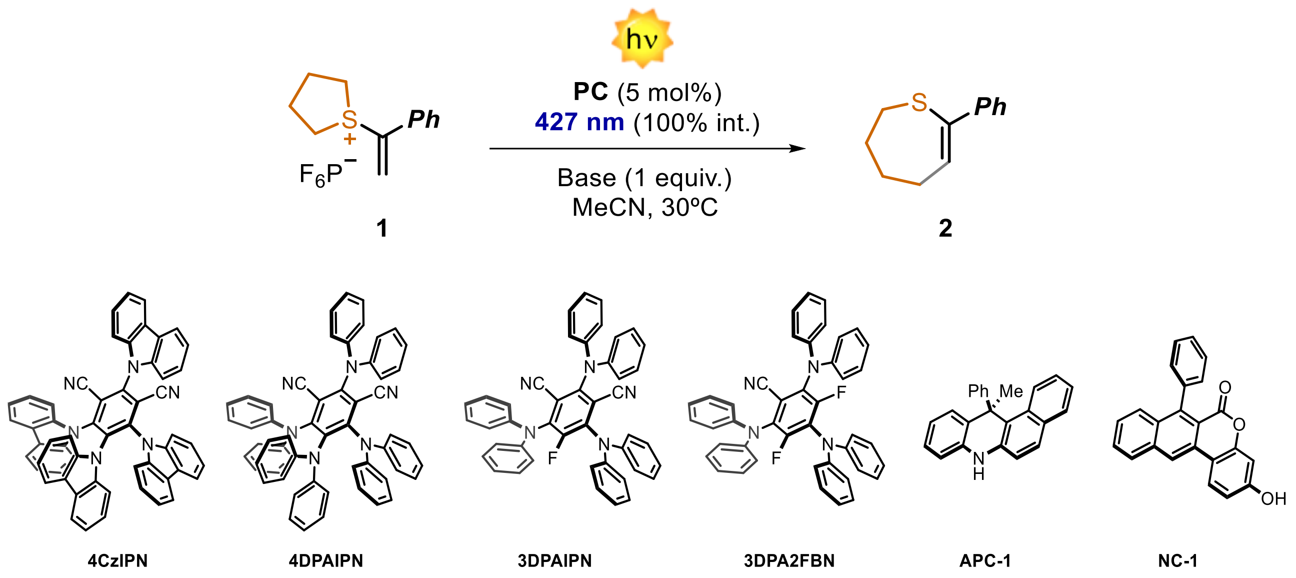

Compound **1** was prepared following General procedure A (see section 4). The photocatalysts studied in this work were synthesizes according to the procedures reported in the literature.[4]

Table 1. *Screening of various bases and photocatalysts in batch conditions. a) Substrate* ***1*** *(0.1 mmol),* ***PC*** *(5 mol%, 0.005 mmol), inert atmosphere. b) Conversion of* ***1*** *and yield of* ***2*** *correspond to the average of 2 reactions and were measured by* ^1H *NMR analysis of the crude reaction mixtures using dibromomethane (0.5 eq.) as internal standard.*

entry^a	*photocatalyst*	*reaction time*	*base*	*conversion (%)*^{b}	*yield (%)*^{b}
1	---	4h	---	<1	---
2	**4DPAIPN**	4h	---	5	traces
3	**4DPAIPN**	4h	K_2CO_3	23	traces
4	**4DPAIPN**	4h	NH_4Oac	>99	traces
5	**4DPAIPN**	4h	Et_3N	98	28
6	**4CzIPN**	4h	2,6-lutidine	2	traces
**7**	**4DPAIPN**	**4h**	**2,6-lutidine**	**58**	**33**
8	**3DPAFIPN**	4h	2,6-lutidine	77	32
9	**3DPA2FBN**	4h	2,6-lutidine	98	28
10	**APC-1**	4h	2,6-lutidine	11	traces
11	**NC-1**^{c}	4h	2,6-lutidine	33	14

Note: Et_3N opens the cyclic sulfide (entry 5), acting as a nucleophile. c) 400 nm Kessil lamp was used with **NC-1** (entry 11).

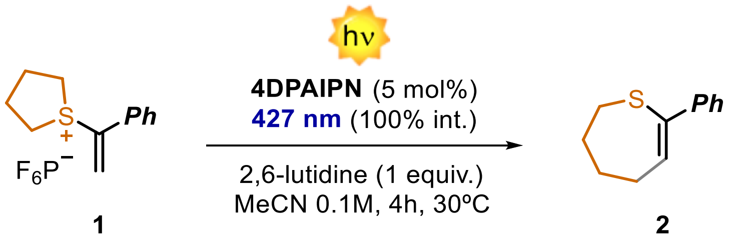

Table 2. *Screening of different parameters, stated as* *<u>variation</u>* *in the table, in batch conditions. a) Substrate* ***1*** *(0.1 mmol), inert atmosphere. b) Conversion of* ***1*** *and yield of* ***2*** *correspond to the average of 2 reactions and were measured by* ^1H *NMR analysis of the crude reaction mixtures using dibromomethane (0.5 eq.) as internal standard.*

entry^a	*variation*	*conversion (%)*^{b}	*yield (%)*^{b}
1	---	58	33
2	2 equiv. base	56	30
3	Concentration 0.05 M	39	20
4	6 hours	73	28
**5**	**467 nm**	**54**	**32**
6	467 nm and PC 10 mol%	52	30

Note: 467 nm Kessil lamp was employed as it was less energetic than 427 nm and the mass balance was similar (entries 1 and 5).

Analysis of the reaction profile

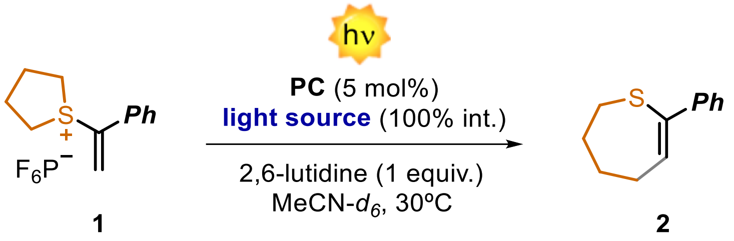

We decided to evaluate the reaction's profile by ^{1}H NMR, performing the reaction directly in deuterated MeCN. Reaction conditions: substrate **1** (0.1 mmol, 1 eq.), **PC** (5 mol%, 0.005 mmol, 2,6-lutidine (0.1 mmol, 1 eq.) in 1 mL of deuterated MeCN (0.05 mmol of 1,5-dibromopentane were added as internal standard (i.s). In order to maintain the inert atmosphere for the reaction, the mixture was degassed into an Schlenk tube, which was later introduced into a glove box to transfer the content into 2 NMR tubes. The NMR tubes were closed with a cap, sealed with parafilm® and they were placed at 5 cm from the light source and irradiated at 427 nm (set at 100% of its maximum intensity, 45W). The reaction progress was then checked acquiring the ^{1}H NMR directly form the NMR tube.

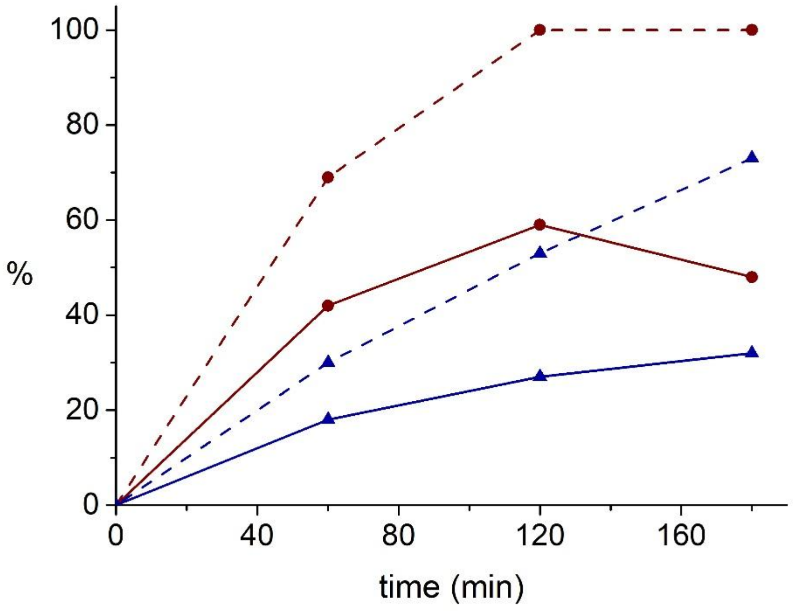

Figure S5. *Reaction profile using* ***4PDAIPN*** *(red) and* ***3DPAFIPN*** *(blue) as PC. Conversion of* ***1*** *(dashed lines) and yield of* ***2*** *(straight lines) correspond to the average of two reactions and were measured by* ^{1}H *NMR.*

Photocatalytic ring expansion of Sulfonium Salts. Screening in flow conditions

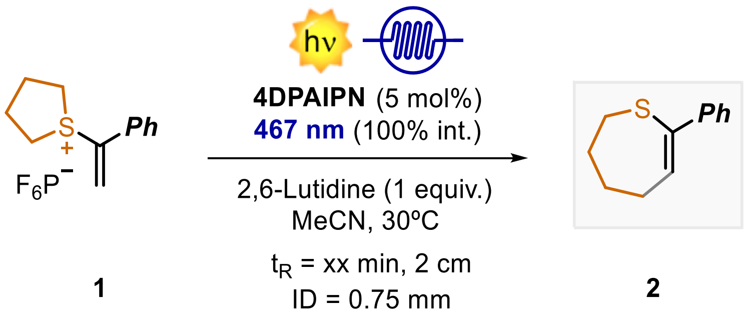

Table 3. *Screening of various residence times and reactor volumes. a) Substrate* ***1*** *(0.1 mmol),* ***PC*** *(5 mol%, 0.005 mmol), inert atmosphere. b) Conversion of* ***1*** *and yield of* ***2*** *correspond to the average of 2 reactions and were measured by* ^{1}*H NMR analysis of the crude reaction mixtures using dibromomethane (0.5 eq.) as internal standard.*

entry[a]	*reactor volume (μL)*	*residence time (min)*	*conversion (%)*[b]	*yield (%)*[b]
1	150[c]	10		
2	150[d]	10		
3	150[e]	10		
4	300[f]	5	54	46
5	300[f]	7.5	83	63
**6**	**300[f]**	**10**	**>99**	**82[h] (77)[i]**
7	300[f]	15	>99	80
**8**	**600[g]**	**10**	**>99**	**83[j]**

Note: c) reactor from Figure S6 a; d) reactor from Figure S6 b; e) reactor from Figure S6 c; f) reactor from Figure S6 d; g) reactor from Figure S6 e; h) productivity in a 300μL reactor: 28 mg/h; i) yield of isolated **2** in a 0.3 mmol scale; j) productivity in a 600μL reactor: 56 mg/h.

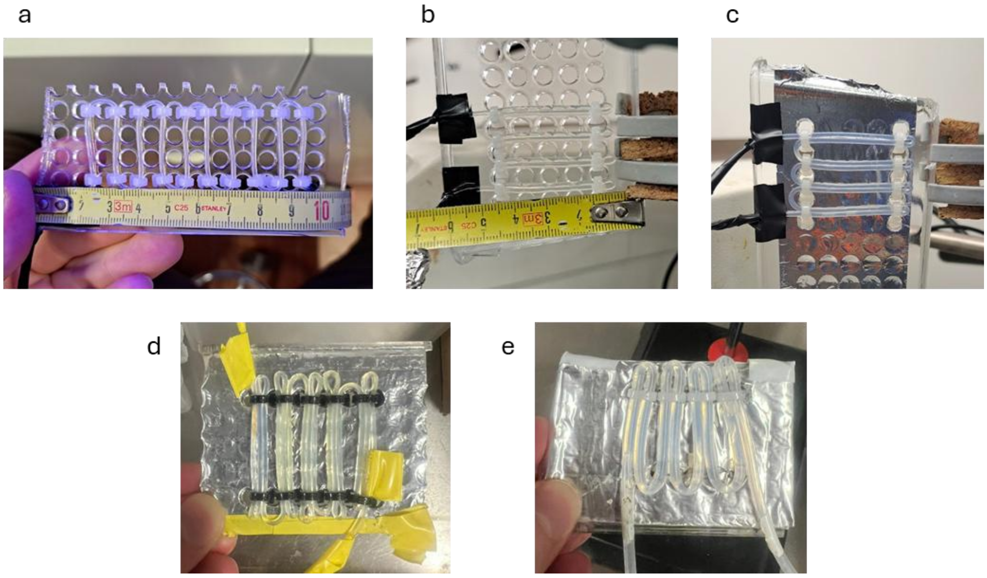

Figure S6. *Homemade flow reactors studied: 150 μL (a, b and c), 300 μL (d) and 600 μL (e).*

Steady state analysis

In order to study the robustness of this flow system, we analyse the steady state for both geminal and trans SSs, collecting every reactor volume (V_R) to analyse the NMR yield of the products. These data were collected using a 600μL reactor, increasing also the scale of the reaction up to 1 mmol.

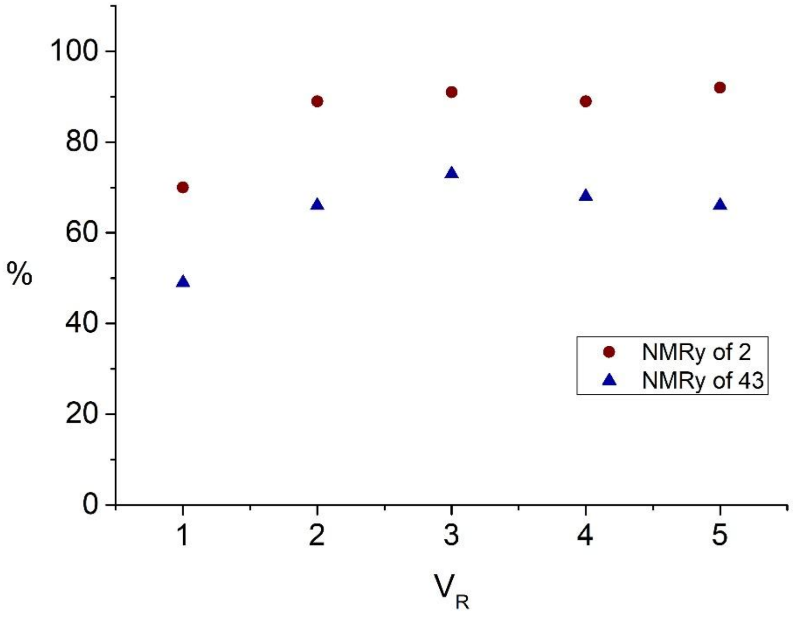

Figure S7. *Steady state analysis for the photochemical ring expansion of geminal SSs (red) and trans SSs (blue) in flow.*

4. Synthesis and characterization of the starting materials (PC and sulfonium salts)

General procedure B for the preparation of geminal SSs

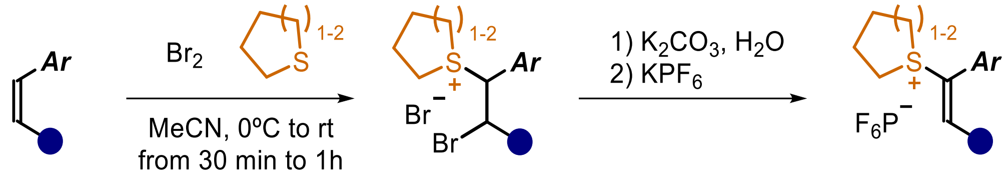

1, **3-13**

In a general procedure, Br_2 (1 equiv.) is added *via* syringe into a 100 mL round bottom flask equipped with a stirring bar, a suba-seal septum and a balloon with Ar, containing the solvent (MeCN or DCM, 0.5M) at 0ºC. Then, the sulfide (4 equiv.) is added dropwise over 5 min *via* syringe (a yellowish precipitate is usually formed but later is dissolved). Thereafter, the alkene (1.1 equiv.) is added dropwise over 5 min via syringe. At this moment, the reaction is stirred for 15 min at 0ºC and then allowed to rise to r.t. for 1 hour. Usually, a solid should appear and, if that is the case, filtration and washing with Et_2O gives the corresponding SSs bromides. Otherwise, solvent is removed (taking into consideration the volatility and smell of the sulfides, a bleach trap is mandatory), and the crude is used in the next step without further purification.

In the next step, SSs bromides are dispersed in a 1:2 mixture MeOH : K_2CO_3 sat. (2 mL, 4 mL). Once the reaction is complete (usually when all the solid is dissolved and formed a clear solution), KPF_6 (1.1 equiv.) is added in the solution and geminal SSs hexafluorophosphates (V) precipitate immediately as a white solid. After 10 min stirring vigorously, the solid is filtrated and washed with water and Et_2O.

Note: the synthesis of geminal SS **6** and **14** were performed following different protocols, which are described in their characterization.

General procedure C for the preparation of *trans* SSs

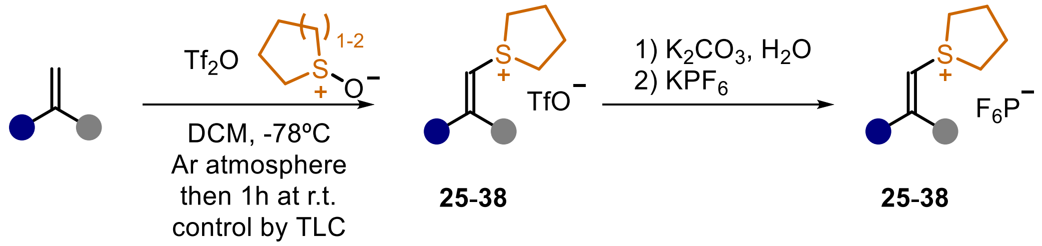

25-38 **25-38**

In a general procedure, sulfoxide (1.1 equiv.) is added *via* syringe into a 100 mL round bottom flask equipped with a stirring bar, a suba-seal septum and a balloon with Ar, containing the solvent (DCM, 0.5M) at -78ºC. Then, Tf_2O (1.1 equiv.) is added dropwise *via* syringe and the reaction is then stirred for 10 min. At this moment, the styrene derivative (1.0 equiv.) is added dropwise *via* syringe and the reaction is allowed to rise to r.t. for 1 hour (reaction monitored by TLC). After completion of the reaction, the solution is added dropwise into Et_2O (15 mL) under stirring. Usually, a solid should appear and, if

that is the case, filtration and washing with Et_2O gives the corresponding trans SSs triflate. Otherwise, solvent is removed, and the crude is used in the next step without further purification.

In the next step, SSs triflates are dissolved in a 1:2 mixture MeOH : H_2O (2 mL, 4 mL). Then, KPF_6 (1.1 equiv.) is added in the solution and *trans* SSs hexafluorophosphates (V) precipitate immediately as a white solid. After 10 min stirring vigorously, the solid is filtrated and washed with water and Et_2O.

Note: the synthesis of geminal SSs **37** and **41** were performed following a modified general procedure B as described below.

Characterization of sulfonium salts 1, 3-13, 33-44

1-(1-phenylvinyl)tetrahydro-1*H*-thiophen-1-ium hexafluorophosphate (V) (1)

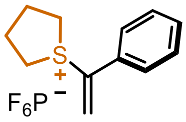

1

Synthesized following general procedure B starting from tetrahydrothiophene (1.06 mL, 12 mmol, 4 equiv.), bromine (306 µL, 3 mmol, 1 equiv.) and styrene (381 µL, 3.3 mmol, 1.1 equiv.) in 7.5 mL of dry MeCN. Further elimination and anion exchange yielded **1** (514 mg, 51%) by precipitation as a white solid.

^{1}H NMR (400 MHz, CD_3CN) δ 7.65 – 7.44 (m, 5H), 6.32 (d, *J* = 3.7 Hz, 1H), 6.14 (d, *J* = 3.7 Hz, 1H), 3.86 – 3.59 (m, 2H), 3.51 (m, 2H), 2.21 (m, 4H).
^{19}F NMR (376 MHz, CD_3CN) δ -72.6 (d, $J_{P\text{-}F}$ = 707.4 Hz).
^{13}C NMR (101 MHz, CD_3CN) δ 136.2, 133.6, 131.9, 130.5, 129.2, 127.6, 46.1, 29.1.
HRMS (ESI+): m/z calc. for $[C_{12}H_{15}S]^+$: 191.0889; found: 191.0892.

1-(1-(4-fluorophenyl)vinyl)tetrahydro-1*H*-thiophen-1-ium hexafluorophosphate (V) (3)

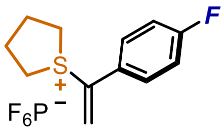

3

Synthesized following the general procedure B starting from tetrahydrothiophene (1.06 mL, 12 mmol, 4 equiv.), bromine (306 µL, 3 mmol, 1 equiv.) and 4-fluorostyrene (393 µL, 3.3 mmol, 1.1 equiv.) in 7.5 mL of dry MeCN. Further elimination and anion exchange yielded **3** (446 mg, 42%) by precipitation as a white solid.

^{1}H NMR (400 MHz, CD_3CN) δ 7.68 – 7.49 (m, 2H), 7.36 – 7.17 (m, 2H), 6.30 (d, *J* = 3.7 Hz, 1H), 6.15 (d, *J* = 3.7 Hz, 1H), 3.76 – 3.60 (m, 2H), 3.56 – 3.42 (m, 2H), 2.31 – 2.12 (m, 4H).
^{19}F NMR (376 MHz, CD_3CN) δ -72.6 (d, $J_{P\text{-}F}$ = 707.4 Hz), -110.9.
^{13}C NMR (101 MHz, CD_3CN) δ 165.0 (d, *J* = 249.6 Hz), 135.2, 131.8 (d, *J* = 8.9 Hz), 130.8 (d, *J* = 36.2 Hz), 130.0 (d, *J* = 3.3 Hz), 128.2, 118.5, 117.5 (d, *J* = 22.3 Hz), 46.2, 29.1.
HRMS (ESI+): m/z calc. for $[C_{12}H_{14}FS]^+$: 209.0795; found: 209.0792.

1-(1-(4-chlorophenyl)vinyl)tetrahydro-1*H*-thiophen-1-ium hexafluorophosphate (V) (4)

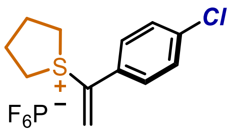

4

Synthesized following the general procedure B starting from tetrahydrothiophene (1.06 mL, 12 mmol, 4 equiv.), bromine (306 µL, 3 mmol, 1 equiv.) and 4-chlorostyrene (396 µL, 3.3 mmol, 1.1 equiv.) in 7.5 mL of dry MeCN. Further elimination and anion exchange yielded **4** (533.8 mg, 48%) by precipitation as a white solid.

^{1}H NMR (400 MHz, $CDCl_3$) δ 7.49 (d, *J* = 8.7 Hz, 2H), 7.41 (d, *J* = 8.7 Hz, 2H), 6.27 (d, *J* = 3.6 Hz, 1H), 6.22 (d, *J* = 3.5 Hz, 1H), 4.03 – 3.78 (m, 2H), 3.62 – 3.44 (m, 2H), 2.53 – 2.31 (m, 2H), 2.30 – 2.17 (m, 2H).
^{19}F NMR (376 MHz, $CDCl_3$) δ -72.0 (d, J_{P-F} = 713.0 Hz).
^{13}C NMR (101 MHz, CD_3CN) δ 137.5, 135.1, 132.4, 130.9, 130.6, 128.5, 46.3, 29.1.
HRMS (ESI+): m/z calc. for $[C_{12}H_{14}ClS]^+$: 225.0499; found: 225.0501.

1-(1-(4-bromophenyl)vinyl)tetrahydro-1*H*-thiophen-1-ium hexafluorophosphate (V) (**5**)

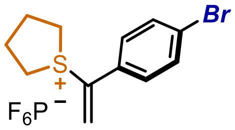

5

Synthesized following the general procedure B starting from tetrahydrothiophene (1.06 mL, 12 mmol, 4 equiv.), bromine (306 µL, 3 mmol, 1 equiv.) and 4-bromostyrene (431 µL, 3.3 mmol, 1.1 equiv.) in 7.5 mL of dry MeCN. Further elimination and anion exchange yielded **5** (711 mg, 52%) by precipitation as a white solid.

^{1}H NMR (400 MHz, CD_3CN) δ 7.71 (d, *J* = 8.4 Hz, 2H), 7.47 – 7.40 (d, *J* = 8.4 Hz, 2H), 6.35 (d, *J* = 3.8 Hz, 1H), 6.16 (d, *J* = 3.8 Hz, 1H), 3.72 – 3.61 (m, 2H), 3.55 – 3.46 (m, 2H), 2.27 – 2.16 (m, 4H).
^{19}F NMR (376 MHz, CD_3CN) δ -72.0 (d, J_{P-F} = 706.6 Hz)
^{13}C NMR (101 MHz, CD_3CN) δ 35.24, 133.6, 132.8, 131.1, 128.5, 125.8, 46.3, 29.1.
HRMS (ESI+): m/z calc. for $[C_{12}H_{14}BrS]^+$: 268.9994; found: 269.0001.

1-(1-(4-methoxyphenyl)vinyl)tetrahydro-1*H*-thiophen-1-ium hexafluorophosphate (V) (**6**)

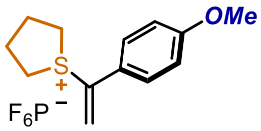

6

The corresponding triflate SS **5** was synthesized following a described procedure in the literature[5] from 4-methoxyacetophenone (751 mg, 5 mmol, 1 equiv.), 2,6-di-tert-butyl-4 methylpyridine (1.078 g, 5.25 mmol, 1.05 equiv.), trifluoromethanesulfonic acid anhydride (0.93 mL, 5.5 mmol, 1.1 equiv.) and tetrahydrothiophene (0.44 mL, 5 mmol, 1 equiv.) in 50 mL of dry DCM. Then, following anion exchange (general procedure A) yielded **6** (1.090 g, 60%) by extraction with DCM as a brownish oil.

^{1}H NMR (400 MHz, CD_3CN) δ 7.46 (d, *J* = 8.6 Hz, 2H), 7.05 (d, *J* = 8.6 Hz, 2H), 6.23 (d, *J* = 3.6 Hz, 1H), 6.03 (d, *J* = 3.6 Hz, 1H), 3.84 (s, 3H), 3.71 – 3.59 (m, 2H), 3.53 – 3.43 (m, 2H), 2.27 – 2.18 (m, 4H).
^{19}F NMR (376 MHz, CD_3CN) δ -72.9 (d, J_{P-F} = 706.6 Hz)
^{13}C NMR (151 MHz, CD_3CN) δ 162.7, 130.8, 125.9, 125.6, 124.2, 115.8, 56.3, 46.0, 29.1.
HRMS (ESI+): m/z calc. for $[C_{13}H_{17}OS]^+$: 221.0995; found: 221.0996.

1-(1-(*p*-tolyl)vinyl)tetrahydro-1*H*-thiophen-1-ium hexafluorophosphate (V) (**7**)

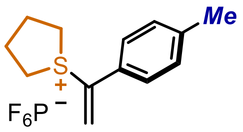

7

Synthesized following the general procedure B starting from tetrahydrothiophene (1.06 mL, 12 mmol, 4 equiv.), bromine (306 μL, 3 mmol, 1 equiv.) and 4-methylstyrene (435 μL, 3.3 mmol, 1.1 equiv.) in 7.5 mL of dry MeCN. Further elimination and anion exchange yielded **7** (588.5 mg, 56%) by precipitation as a white solid.

^{1}H NMR (400 MHz, $CDCl_3$) δ 7.31 (bs, 4H), 6.21 (d, *J* = 3.3 Hz, 1H), 6.15 (d, *J* = 3.3 Hz, 1H), 3.93 – 3.81 (m, 2H), 3.56 – 3.45 (m, 2H), 2.41 (s, 3H), 2.40 – 2.35 (m, 2H), 2.23 – 2.14 (m, 2H).

^{19}F NMR (376 MHz, $CDCl_3$) δ -72.43 (d, $J_{P\text{-}F}$ = 713.0 Hz).

^{13}C NMR (101 MHz, CD_3CN) δ 142.5, 136.2, 131.1, 130.8, 129.1, 126.7, 46.1, 29.1, 21.4.

HRMS (ESI+): m/z calc. for $[C_{13}H_{17}S]^+$: 205.1045; found: 205.1041.

1-(1-(*m*-tolyl)vinyl)tetrahydro-1*H*-thiophen-1-ium hexafluorophosphate (V) (**8**)

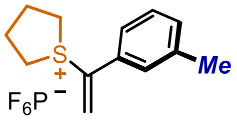

8

Synthesized following the general procedure B starting from tetrahydrothiophene (1.06 mL, 12 mmol, 4 equiv.), bromine (306 μL, 3 mmol, 1 equiv.) and 3-methylstyrene (438 μL, 3.3 mmol, 1.1 equiv.) in 7.5 mL of dry MeCN. Further elimination and anion exchange yielded **8** (557 mg, 53%) by precipitation as a white solid.

^{1}H NMR (400 MHz, $CDCl_3$) δ 7.47 – 7.31 (m, 2H), 7.22 (m,2H), 6.23 (d, *J* = 3.4 Hz, 1H), 6.17 (d, *J* = 3.4 Hz, 1H), 3.89 – 3.79 (m, 2H), 3.58 – 3.46 (m, 2H), 2.41 (s, 3H), 2.42 – 2.35 (m, 2H), 2.25 – 2.18 (m, 2H).

^{19}F NMR (376 MHz, $CDCl_3$) δ -72.3(d, $J_{P\text{-}F}$ = 712.8 Hz).

^{13}C NMR (101 MHz, CD_3CN) δ 140.8, 136.3, 133.6, 132.6, 130.4, 129.7, 127.2, 126.2, 46.1, 29.1, 21.4.

HRMS (ESI+): m/z calc. for $[C_{13}H_{17}S]^+$: 205.1045; found: 205.1046.

1-(1-(*o*-tolyl)vinyl)tetrahydro-1*H*-thiophen-1-ium hexafluorophosphate (V) (**9**)

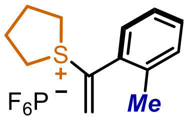

9

Synthesized following the general procedure B starting from tetrahydrothiophene (1.06 mL, 12 mmol, 4 equiv.), bromine (306 μL, 3 mmol, 1 equiv.) and 2-methylstyrene (427 μL, 3.3 mmol, 1.1 equiv.) in 7.5 mL of dry MeCN. Further elimination and anion exchange yielded **9** (520 mg, 49%) by precipitation as a white solid.

^{1}H NMR (400 MHz, CD_3CN) δ 7.71 (d, *J* = 8.4 Hz, 2H), 7.47 – 7.40 (d, *J* = 8.4 Hz, 2H), 6.35 (d, *J* = 3.8 Hz, 1H), 6.16 (d, *J* = 3.8 Hz, 1H), 3.72 – 3.61 (m, 2H), 3.55 – 3.46 (m, 2H), 2.27 – 2.16 (m, 4H).

^{19}F NMR (376 MHz, CD_3CN) δ -72.0 (d, $J_{P\text{-}F}$ = 706.6 Hz).

^{13}C NMR (101 MHz, CD_3CN) δ 35.24, 133.6, 132.8, 131.1, 128.5, 125.8, 46.3, 29.1.

HRMS (ESI+): m/z calc. for $[C_{13}H_{17}S]^+$: 205.1045; found: 205.1048.

(*E*)-1-(1,2-diphenylvinyl)tetrahydro-1*H*-thiophen-1-ium hexafluorophosphate (V) (**10**)

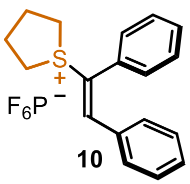

10

Synthesized following the general procedure B starting from tetrahydrothiophene (1.06 mL, 12 mmol, 4 equiv.), bromine (306 µL, 3 mmol, 1 equiv.) and *trans*-stilbene (613 µL, 3.3 mmol, 1.1 equiv.) in 7.5 mL of dry MeCN. Further elimination and anion exchange yielded **10** (631 mg, 51%) by precipitation as a white solid.

^{1}H NMR (400 MHz, $CDCl_3$) δ 7.60 – 7.47 (m, 8H), 7.46 – 7.39 (m, 2H), 3.80 (m, 2H), 3.40 (m, 2H), 2.19 – 2.10 (m, 2H), 1.88 – 1.79 (m, 2H).
^{19}F NMR (376 MHz, $CDCl_3$) δ -72.6 (d, $J_{P\text{-}F}$ = 713.1 Hz).
^{13}C NMR (101 MHz, CD_3CN) δ 150.0, 133.8, 133.2, 131.6, 131.5, 131.3, 130.6, 130.4, 130.0, 127.0, 46.8, 29.9.
HRMS (ESI+): m/z calc. for $[C_{18}H_{19}S]^+$: 267.1202; found: 267.1207.

1-(3,4-dihydronaphthalen-1-yl)tetrahydro-1*H*-thiophen-1-ium hexafluorophosphate (V) (**11**)

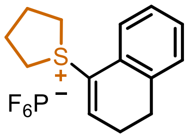

11

Synthesized following the general procedure B starting from tetrahydrothiophene (1.06 mL, 12 mmol, 4 equiv.), bromine (306 µL, 3 mmol, 1 equiv.) and 1,2-dihydronaphthalene (431 µL, 3.3 mmol, 1.1 equiv.) in 7.5 mL of dry MeCN. Further elimination and anion exchange yielded **11** (848 mg, 78%) by precipitation as a white solid.

1H NMR (400 MHz, $CDCl_3$) δ 7.44 – 7.37 (m, 1H), 7.36 – 7.32 (m, 2H), 6.81 (t, *J* = 4.9 Hz, 1H), 3.96 (m, 2H), 3.65 (m, 2H), 2.87 (t, *J* = 8.1 Hz, 2H), 2.65 (m, 2H), 2.55 – 2.41 (m, 4H).
^{19}F NMR (376 MHz, $CDCl_3$) δ -72.6 (d, $J_{P\text{-}F}$ = 713.0 Hz).
^{13}C NMR (101 MHz, CD_3CN) δ 139.4, 137.8, 130.8, 129.7, 129.7, 128.2, 125.4, 123.9, 46.4, 29.2, 26.8, 25.3.
HRMS (ESI+): m/z calc. for $[C_{14}H_{17}S]^+$: 217.1045; found: 217.1044.

1-(1-phenylvinyl)hexahydrothiopyrylium hexafluorophosphate (V) (**12**)

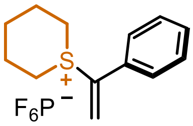

12

Synthesized following the general procedure B starting from pentamethylene sulfide (1.23 mL, 12 mmol, 4 equiv.), bromine (306 µL, 3 mmol, 1 equiv.) and styrene (381 µL, 3.3 mmol, 1.1 equiv.) in 7.5 mL of dry MeCN. Further elimination and anion exchange yielded **12** (420 mg, 40%) by precipitation as a white solid.

^{1}H NMR (400 MHz, CD_3CN) δ 7.61 – 7.47 (m, 5H), 6.40 (d, *J* = 3.5 Hz, 1H), 6.19 (d, *J* = 3.5 Hz, 1H), 3.56 (m, 2H), 3.20 (ddd, *J* = 13.1, 10.3, 3.0 Hz, 2H), 2.16 (m, 2H), 1.91 (m, 2H), 1.89 – 1.70 (m, 1H), 1.64 – 1.49 (m, 1H).
^{19}F NMR (376 MHz, CD_3CN) δ -72.3 (d, $J_{P\text{-}F}$ = 707.9 Hz).
^{13}C NMR (101 MHz, CD_3CN) δ 135.4, 132.9, 131.9, 130.6, 128.9, 128.8, 39.3, 23.3, 22.4.
HRMS (ESI+): m/z calc. for $[C_{13}H_{17}S]^+$: 205.1045; found: 205.1053.

1-(1-(naphthalen-2-yl)vinyl)tetrahydro-1*H*-thiophen-1-ium hexafluorophosphate (V) (**13**)

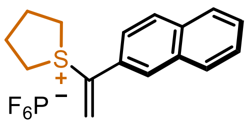

13

Synthesized following the general procedure B starting from tetrahydrothiophene (1.23 mL, 12 mmol, 4 equiv.), bromine (306 µL, 3 mmol, 1 equiv.) and 2-vinylnaphthalene (509 mg, 3.3 mmol, 1.1 equiv.) in 7.5 mL of dry MeCN. Further elimination and anion exchange yielded **13** (580 mg, 54%) by precipitation as a white solid.

^{1}H NMR (400 MHz, CD_3CN) δ 8.07 (s, 1H), 8.05 – 7.88 (m, 3H), 7.61 (m, 3H), 6.43 (d, J = 3.7 Hz, 1H), 6.20 (d, J = 3.7 Hz, 1H), 3.76 – 3.68 (m, 2H), 3.61 – 3.50 (m, 2H), 2.35 – 2.15 (m, 4H).
^{19}F NMR (376 MHz, CD_3CN) δ -72.6 (d, $J_{P\text{-}F}$ = 707.3 Hz).
^{13}C NMR (101 MHz, CD_3CN) δ 136.2, 134.9, 133.9, 131.0, 130.4, 129.6, 129.3, 129.0, 128.8, 128.5, 127.5, 125.6, 46.2, 29.1.
HRMS (ESI+): m/z calc. for $[C_{16}H_{17}S]^+$: 241.1045; found: 241.1040.

1-vinyltetrahydro-1H-thiophen-1-ium hexafluorophosphate (V) (**14**)

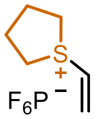

14

We followed a described procedure in the literature.[6] To a solution of 2-bromoethyl trifluoromethanesulfonate (3.40 g, 13.2 mmol, 1 equiv.) in dry DCM (25 mL) was added tetrahydrothiophene (1.20 mL, 13.6 mmol, 1.03 equiv.) dropwise over 5 min at r.t. under inert atmosphere. Then, the reaction mixture was refluxed at 45 °C for 1 day after which the DCM was removed under vacuum and anhydrous diethyl ether (20 mL) was added to the resulting residue and it was stirred for 1 h to precipitate the product 1-(2-bromoethyl)tetrahydro-1H-thiophenium trifluoromethanesulfonate, which was isolated by filtration as a white solid and was used in the next step without further purification. A suspension of the latter (4.70 g, 13.6 mmol, 1.03 equiv.) and silver (I) oxide (6.30 g, 27.2 mmol, 2.06 equiv.) in deionized water (10 mL) and THF (10 mL) was stirred for 20 h at r.t.. Then, the reaction mixture was filtered through Celite and the filtrate was concentrated under reduced pressure. The residue was dissolved in DCM (30 mL), dried over $MgSO_4$, filtered, evaporated and the residue was again dissolved in DCM (10 mL) and passed through silica. The residue was washed with DCM (aprox. 200 mL) and then with 10% MeOH in DCM. The solvent was removed under vacuum and the corresponding SS triflate was redissolved in a 1:2 mixture MeOH: H_2O. Then, KPF_6 (1.1 equiv.) is added in the solution and *trans* SSs hexafluorophosphates (V) precipitate immediately as a white solid. After 10 min stirring vigorously, the solid is filtrated and washed with water and Et_2O.

^{1}H NMR (400 MHz, CD_3CN) δ 6.50 (dd, J = 15.9, 9.3 Hz, 1H), 6.41 – 6.29 (m, 2H), 3.64 (m, 2H), 3.41 (dt, J = 12.4, 5.6 Hz, 2H), 2.29 (m, 4H).
^{19}F NMR (376 MHz, CD_3CN) δ -72.92 (d, $J_{P\text{-}F}$ = 706.5 Hz).

(*E*)-1-styryltetrahydro-1*H*-thiophen-1-ium hexafluorophosphate (V) (**33**)

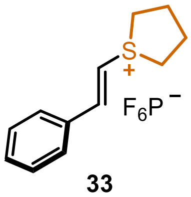

33

Synthesized following the general procedure C starting from tetrahydrothiophene 1-oxide (206 µL, 2.2 mmol, 1.1 equiv.), trifluoromethanesulfonic acid anhydride (372 µL, 2.2 mmol, 1.1 equiv.) and styrene (229 µL, 2 mmol, 1 equiv.) in 4 mL of dry MeCN. Further anion exchange yielded **33** (580 mg, 54%) by precipitation as a white solid.

^{1}H NMR (400 MHz, CD_3CN) δ 7.76 – 7.48 (m, 6H), 6.78 (d, *J* = 15.0 Hz, 1H), 3.69 (m, 2H), 3.45 (m, 2H), 2.48 – 2.37 (m, 2H), 2.34 – 2.23 (m, 2H).
^{19}F NMR (376 MHz, CD_3CN) δ -72.5 (d, $J_{P\text{-}F}$ = 707. Hz).
^{13}C NMR (101 MHz, CD_3CN) δ 149.8, 133.8, 132.7, 130.3, 129.4, 113.7, 48.6, 29.6.
HRMS (ESI+): m/z calc. for $[C_{12}H_{15}S]^+$: 191.0889; found: 191.0890.

(*E*)-1-(4-fluorostyryl)tetrahydro-1*H*-thiophen-1-ium hexafluorophosphate (V) (**34**)

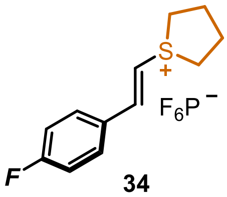

34

Synthesized following the general procedure C starting from tetrahydrothiophene 1-oxide (206 µL, 2.2 mmol, 1.1 equiv.), trifluoromethanesulfonic acid anhydride (372 µL, 2.2 mmol, 1.1 equiv.) and 4-fluorostyrene (238 µL, 2 mmol, 1 equiv.) in 4 mL of dry MeCN. Further anion exchange yielded **34** (460 mg, 65%) by precipitation as a white solid.

^{1}H NMR (400 MHz, CD_3CN) δ 7.70 – 7.61 (m, 2H), 7.55 (d, *J* = 15.3 Hz, 1H), 7.30 – 7.18 (m, 2H), 6.72 (d, *J* = 15.3 Hz, 1H), 3.68 (m, 2H), 3.49 – 3.38 (m, 2H), 2.47 – 2.35 (m, 2H), 2.27 (m 2H).
^{19}F NMR (376 MHz, CD_3CN) δ -72.9 (d, $J_{P\text{-}F}$ = 706.5 Hz), -109.3.
^{13}C NMR (101 MHz, CD_3CN) δ 165.50(d, *J* = 250.9 Hz), 148.5, 131.8 (d, *J* = 8.9 Hz), 130.4 (d, *J* = 3.3 Hz), 117.3 (d, *J* = 22.4 Hz), 113.5 (d, *J* = 2.5 Hz), 48.6, 29.6.
HRMS (ESI+): m/z calc. for $[C_{12}H_{14}FS]^+$: 209.0795; found: 209.0797.

(*E*)-1-(4-chlorostyryl)tetrahydro-1*H*-thiophen-1-ium hexafluorophosphate (V) (**35**)

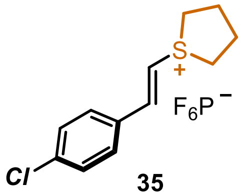

35

Synthesized following the general procedure C starting from tetrahydrothiophene 1-oxide (206 µL, 2.2 mmol, 1.1 equiv.), trifluoromethanesulfonic acid anhydride (372 µL, 2.2 mmol, 1.1 equiv.) and 4-chlorostyrene (240 µL, 2 mmol, 1 equiv.) in 4 mL of dry MeCN. Further anion exchange yielded **35** (430 mg, 58%) by precipitation as a white solid.

^{1}H NMR (400 MHz, CD_3CN) δ 7.59 (d, *J* = 8.6 Hz, 2H), 7.55 (d, *J* = 15.3 Hz, 1H), 7.50 (d, *J* = 8.6 Hz, 2H), 6.78 (d, *J* = 15.3 Hz, 1H), 3.74 – 3.62 (m, 2H), 3.50 – 3.36 (m, 2H), 2.46 – 2.35 (m, 4H).
^{19}F NMR (376 MHz, CD_3CN) δ -72.9 (d, $J_{P\text{-}F}$ = 706.7 Hz).
^{13}C NMR (101 MHz, CD_3CN) δ 148.3, 138.0, 132.6, 130.9, 130.4, 114.6, 48.6, 29.6.
HRMS (ESI+): m/z calc. for $[C_{12}H_{14}ClS]^+$: 225.0499; found: 225.0494.

(*E*)-1-(4-bromostyryl)tetrahydro-1*H*-thiophen-1-ium hexafluorophosphate (V) (**36**)

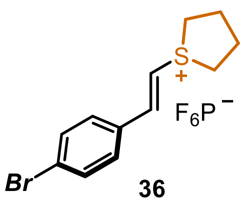

36

Synthesized following the general procedure C starting from tetrahydrothiophene 1-oxide (206 µL, 2.2 mmol, 1.1 equiv.), trifluoromethanesulfonic acid anhydride (372 µL, 2.2 mmol, 1.1 equiv.) and 4-bromostyrene (261 µL, 2 mmol, 1 equiv.) in 4 mL of dry MeCN. Further anion exchange yielded **36** (506 mg, 61%) by precipitation as a white solid.

^{1}H NMR (400 MHz, CD_3CN) δ 7.66 (d, *J* = 8.5 Hz, 2H), 7.57 – 7.48 (m, 3H), 6.80 (d, *J* = 15.3 Hz, 1H), 3.85 – 3.59 (m, 2H), 3.53 – 3.36 (m, 2H), 2.54 – 2.33 (m, 4H).
^{19}F NMR (376 MHz, CD_3CN) δ -72.9 (d, $J_{P\text{-}F}$ = 706.5 Hz).
^{13}C NMR (101 MHz, CD_3CN) δ 148.4, 133.3, 133.0, 131.1, 126.4, 114.7, 48.6, 29.6.
HRMS (ESI+): m/z calc. for $[C_{12}H_{14}BrS]^{+}$: 268.9994; found: 269.0003.

(*E*)-1-(4-cyanostyryl)tetrahydro-1*H*-thiophen-1-ium hexafluorophosphate (V) (**37**)

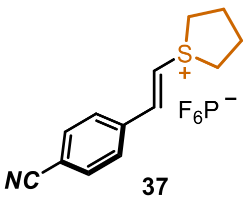

37

Synthesized following a modification of general procedure B starting from tetrahydrothiophene (1.06 mL, 12 mmol, 4 equiv.), bromine (306 µL, 3 mmol, 1 equiv.) and 4-cyanostyrene (426 mg, 3.3 mmol, 1.1 equiv.) in 7.5 mL of dry MeCN. After leaving the reaction to stir overnight, further elimination and anion exchange yielded **37** (574 mg, 53%) by precipitation as a white solid.

^{1}H NMR (400 MHz, CD_3CN) 7.83 (d, *J* = 8.0 Hz, 2H), 7.73 (d, *J* = 8.1 Hz, 2H), 7.60 (d, *J* = 15.3 Hz, 1H), 6.91 (d, *J* = 15.3 Hz, 1H), 3.76 – 3.65 (m, 2H), 3.55 – 3.44 (m, 2H), 2.77 – 2.38 (m, 2H), 2.38 – 2.25 (m, 2H).
^{19}F NMR (376 MHz, CD_3CN) δ -72.8 (d, $J_{P\text{-}F}$ = 706.7 Hz).
^{13}C NMR (101 MHz, CD_3CN) δ 147.4, 137.8, 134.0, 129.8, 119.1, 117.8, 115.2 48.6, 29.7.
HRMS (ESI+): m/z calc. for $[C_{13}H_{14}NS]^{+}$: 216.0841; found: 216.0836.

(*E*)-1-(2-([1,1'-biphenyl]-4-yl)vinyl)tetrahydro-1*H*-thiophen-1-ium hexafluorophosphate (V) (**38**)

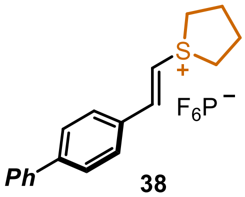

38

Synthesized following the general procedure C starting from tetrahydrothiophene 1-oxide (206 µL, 2.2 mmol, 1.1 equiv.), trifluoromethanesulfonic acid anhydride (372 µL, 2.2 mmol, 1.1 equiv.) and 4-vinylbiphenyl 360 mg, 2 mmol, 1 equiv.) in 4 mL of dry MeCN. Further anion exchange yielded **38** (660 mg, 80%) by precipitation as a white solid.

^{1}H NMR (400 MHz, CD_3CN) δ 7.61 – 7.46 (m, 2H), 7.46 – 7.36 (m, 4H), 7.33 (d, *J* = 15.3 Hz, 1H), 7.27 – 7.19 (m, 2H), 7.19 – 7.10 (m, 1H), 6.53 (d, *J* = 15.3 Hz, 1H), 3.66 – 3.34 (m, 2H), 3.32 – 3.06 (m, 2H), 2.23 – 2.11 (m, 2H), 2.09 – 1.95 (m, 2H).
^{19}F NMR (376 MHz, CD_3CN) δ -72.8 (d, $J_{P\text{-}F}$ = 706.3 Hz).
^{13}C NMR (101 MHz, CD_3CN) δ 149.4, 144.9, 140.5, 132.8, 130.1, 130.0, 129.3, 128.6, 128.0, 113.4, 48.6, 29.6.
HRMS (ESI+): m/z calc. for for $[C_{18}H_{19}S]^{+}$: 267.1202; found: 267.1204.

(*E*)-1-(4-methylstyryl)tetrahydro-1*H*-thiophen-1-ium hexafluorophosphate (V) (**39**)

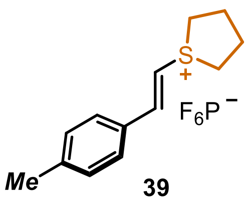

39

Synthesized following the general procedure C starting from tetrahydrothiophene 1-oxide (206 µL, 2.2 mmol, 1.1 equiv.), trifluoromethanesulfonic acid anhydride (372 µL, 2.2 mmol, 1.1 equiv.) and 4-methylstyrene (264 µL, 2 mmol, 1 equiv.) in 4 mL of dry MeCN. Further anion exchange yielded **39** (546 mg, 78%) by precipitation as a white solid.

^{1}H NMR (400 MHz, CD_3CN) δ 7.76 – 7.48 (m, 6H), 6.78 (d, *J* = 15.0 Hz, 1H), 3.69 (m, 2H), 3.45 (m, 2H), 2.48 – 2.37 (m, 2H), 2.34 – 2.23 (m, 2H).
^{19}F NMR (376 MHz, CD_3CN) δ -72.9 (d, $J_{P\text{-}F}$ = 706.2. Hz), -115.7.
^{13}C NMR (101 MHz, CD_3CN) δ 149.8, 133.8, 132.7, 130.3, 129.4, 113.7, 48.6, 29.6.
HRMS (ESI+): m/z calc. for $[C_{13}H_{17}S]^+$: 205.1045; found: 205.1046.

(*E*)-1-(3-methylstyryl)tetrahydro-1*H*-thiophen-1-ium hexafluorophosphate (V) (**40**)

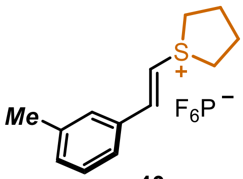

40

Synthesized following the general procedure C starting from tetrahydrothiophene 1-oxide (206 µL, 2.2 mmol, 1.1 equiv.), trifluoromethanesulfonic acid anhydride (372 µL, 2.2 mmol, 1.1 equiv.) and 3-methylstyrene (265 µL, 2 mmol, 1 equiv.) in 4 mL of dry MeCN. Further anion exchange yielded **40** (518 mg, 74%) by precipitation as a white solid.

^{1}H NMR (400 MHz, CD_3CN) δ 7.54 (d, *J* = 15.3 Hz, 1H), 7.47 (s, 1H), 7.45 – 7.39 (m, 1H), 7.39 – 7.29 (m, 2H), 6.77 (d, *J* = 15.3 Hz, 1H), 3.69 (m, 2H), 3.52 – 3.37 (m, 2H), 2.48 – 2.38 (m, 2H), 2.37 (s, 3H), 2.28 (m, 2H).
^{19}F NMR (376 MHz, CD_3CN) δ -72.3 (d, $J_{P\text{-}F}$ = 707.7 Hz).
^{13}C NMR (101 MHz, CD_3CN) δ 150.05, 140.2, 133.8, 133.4, 130.2, 129.9, 126.7, 113.4, 48.6, 29.6, 21.3.
HRMS (ESI+): m/z calc. for $[C_{13}H_{17}S]^+$: 205.1045; found: 205.1045.

(*E*)-1-(2-methylstyryl)tetrahydro-1*H*-thiophen-1-ium hexafluorophosphate (V) (**41**)

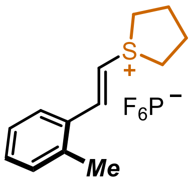

41

Synthesized following a modification of general procedure B starting from tetrahydrothiophene (1.06 mL, 12 mmol, 4 equiv.), bromine (306 µL, 3 mmol, 1 equiv.) and 2-methylstyrene (427 µL, 3.3 mmol, 1.1 equiv.) in 7.5 mL of dry MeCN. After leaving the reaction to stir overnight, further elimination and anion exchange yielded **41** (601 mg, 52%) by precipitation as a white solid.

^{1}H NMR (400 MHz, CD_3CN) δ 7.84 (d, *J* = 15.2 Hz, 1H), 7.66 – 7.55 (m, 1H), 7.47 – 7.36 (m, 1H), 7.34 – 7.25 (m, 3H), 6.67 (d, *J* = 15.2 Hz, 1H), 3.76 – 3.63 (m, 2H), 3.45 (m, 2H), 2.43 (s, 3H), 2.36 – 2.20 (m, 2H).
^{19}F NMR (376 MHz, CD_3CN) δ -73.0 (d, $J_{P\text{-}F}$ = 706.1 Hz).
^{13}C NMR (101 MHz, CD_3CN) δ 147.9, 139.2, 132.8, 132.5, 132.0, 127.9, 127.6, 114.6, 48.6, 29.6, 19.7.

HRMS (ESI+): m/z calc. for $[C_{13}H_{17}S]^+$: 205.1045; found: 205.1040.

1-(2,2-diphenylvinyl)tetrahydro-1*H*-thiophen-1-ium hexafluorophosphate (V) (42)

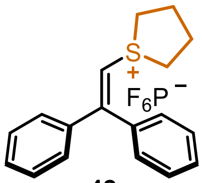
42

Synthesized following the general procedure C starting from tetrahydrothiophene 1-oxide (206 µL, 2.2 mmol, 1.1 equiv.), trifluoromethanesulfonic acid anhydride (372 µL, 2.2 mmol, 1.1 equiv.) and 1,1-diphenylethylene (353 µL, 2 mmol, 1 equiv.) in 4 mL of dry MeCN. Further anion exchange yielded **42** (580 mg, 54%) by precipitation as a white solid.

^{1}H NMR (400 MHz, CD_3CN) δ 7.71 – 7.26 (m, 10H), 6.71 (s, 1H), 3.72 – 3.46 (m, 4H), 2.55 – 2.41 (m, 2H), 2.35 – 2.16 (m, 2H).
^{19}F NMR (376 MHz, CD_3CN) δ -72.6 (d, $J_{P\text{-}F}$ = 707.2 Hz).
^{13}C NMR (101 MHz, CD_3CN) δ 162.0, 138.8, 137.2, 132.1, 131.2, 130.7, 130.1, 129.9, 129.7, 113.2, 49.5, 29.9.
HRMS (ESI+): m/z calc. for $[C_{18}H_{19}S]^+$: 267.1202; found: 267.1199.

1-(3,4-dihydronaphthalen-2-yl)tetrahydro-1*H*-thiophen-1-ium hexafluorophosphate (V) (43)

Synthesized following the general procedure C starting from tetrahydrothiophene 1-oxide (206 µL, 2.2 mmol, 1.1 equiv.), trifluoromethanesulfonic acid anhydride (372 µL, 2.2 mmol, 1.1 equiv.) and 1,2-dihydronaphthalene (261 µL, 2 mmol, 1 equiv.) in 4 mL of dry MeCN. Further anion exchange yielded **43** (514 mg, 71%) by precipitation as a white solid.

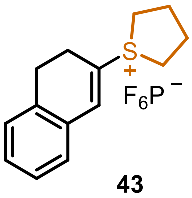
43

^{1}H NMR (400 MHz, CD_3CN) δ 7.43 (s, 1H), 7.41 – 7.35 (m, 1H), 7.33 – 7.23 (m, 3H), 3.78 – 3.63 (m, 2H), 3.61 – 3.50 (m, 2H), 3.06 (t, *J* = 8.2 Hz, 2H), 2.64 (t, *J* = 8.2 Hz, 2H), 2.43 – 2.31 (m, 2H), 2.31 – 2.19 (m, 2H).
^{19}F NMR (376 MHz, CD_3CN) δ -72.8 (d, $J_{P\text{-}F}$ = 707.0 Hz).
^{13}C NMR (101 MHz, CD_3CN) δ 143.4, 136.6, 132.3, 131.8, 129.6, 129.0, 128.3, 123.7, 45.2, 30.2, 28.0, 23.4.
HRMS (ESI+): m/z calc. for $[C_{14}H_{17}S]^+$: 217.1045; found: 217.1048.

(*E*)-1-(2-fluorostyryl)tetrahydro-1*H*-thiophen-1-ium hexafluorophosphate (V) (44)

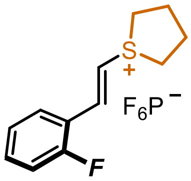
44

Synthesized following the general procedure C starting from tetrahydrothiophene 1-oxide (206 µL, 2.2 mmol, 1.1 equiv.), trifluoromethanesulfonic acid anhydride (372 µL, 2.2 mmol, 1.1 equiv.) and 2-fluorostyrene (238 µL, 2 mmol, 1 equiv.) in 4 mL of dry MeCN. Further anion exchange yielded **44** (368 mg, 52%) by precipitation as a white solid.

^{1}H NMR (400 MHz, CD_3CN) δ 7.72 – 7.62 (m, 2H), 7.60 – 7.50 (m, 1H), 7.34 – 7.29 (m, 1H), 7.28 – 7.19 (m, 1H), 6.86 (d, *J* = 15.4 Hz, 1H), 3.88 – 3.63 (m, 2H), 3.52 – 3.42 (m, 2H), 2.47 – 2.35 (m, 2H), 2.28 (d, *J* = 7.3 Hz, 2H).

^{19}F NMR (376 MHz, CD_3CN) δ -72.5 (d, $J_{P\text{-}F}$ = 707.3 Hz), -115.6 (s).

^{13}C NMR (101 MHz, CD_3CN) δ 162.0 (d, *J* = 253.1 Hz), 142.3 (d, *J* = 3.8 Hz), 134.8 (d, *J* = 9.1 Hz), 130.6 (d, *J* = 2.2 Hz), 126.2 (d, *J* = 3.6 Hz), 121.6 (d, *J* = 11.7 Hz), 117.3 (d, *J* = 21.6 Hz), 116.8 (d, *J* = 6.8 Hz), 48.6, 29.7.

HRMS (ESI+): m/z calc. for $[C_{12}H_{14}FS]^+$: 209.0795; found: 209.0790.

5. Synthesis and characterization of products 2, 15-26, 45-63

General procedure D for the photocatalytic ring expansion of sulfonium salts in flow

A 8 mL screw-cap vial was charged with the sulfonium salt substrate (0.4 mmol, 1 eq.), **4DPAIPN** (5 mol%), 2,6-lutidine (1 eq.) and filled with Argon. Then, dry CH_3CN (0.1 M) was added after degassed by sparging Argon for ten minutes.

Note: in the case of trans sulfonium salts, H_2O or the nucleophile (5 eq.) was added to the reaction mixture. Normal CH_3CN can be used when using H_2O as nucleophile, otherwise CH_3CN was dried over CaH_2.

The mixture was pumped into the flow system (see section 2 for detailed instructions) and irradiated with a 467 nm Kessil lamp set at 100% (45W) of its maximum output power. The reaction crude was collected in different fractions, where 0.5 mL were used to determine de NMR yield using dibromomethane (0.5 eq.) as internal standard and 3.0 mL (0.3 mmol) were used to putify the product by column chromatography on silica gel (using cerium molybdate to stain the TLC).

Characterization of products 2, 15-26, 45-63

7-phenyl-2,3,4,5-tetrahydrothiepine (2)

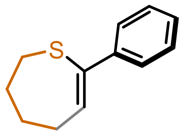

2

Synthesized following the general procedure D (0.4 mmol of **1**, 134.5 mg). After the purification of a fraction of 3 mL (0.3 mmol) by column chromatography on silica gel (hexane), **2** was isolated as a colorless oil (45.7 mg, 80% yield).

^{1}H NMR (400 MHz, $CDCl_3$) δ 7.61 – 7.54 (m, 2H), 7.35 – 7.21 (m, 3H), 6.43 (t, *J* = 6.9 Hz, 1H), 2.89 – 2.81 (m, 2H), 2.59 – 2.50 (m, 2H), 2.11 – 2.01 (m, 2H), 1.71 – 1.61 (m, 2H).
^{13}C NMR (101 MHz, $CDCl_3$) δ 141.6, 141.2, 133.6, 128.2, 127.7, 127.4, 34.6, 32.5, 30.0, 24.6.
HRMS (ESI+): m/z calc. for $[C_{12}H_{15}S]^+$: 191.0816; found: 191.0820.

7-(4-fluorophenyl)-2,3,4,5-tetrahydrothiepine (15)

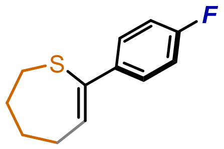

15

Synthesized following the general procedure D (0.4 mmol of **3**, 141.7 mg). After the purification of a fraction of 3 mL (0.3 mmol) by column chromatography on silica gel (hexane), **15** was isolated as a colorless oil (51.9 mg, 83% yield).

^{1}H NMR (400 MHz, $CDCl_3$) δ 7.54 (dd, *J* = 8.7, 5.6 Hz, 2H), 6.98 (t, *J* = 8.7 Hz, 2H), 6.35 (t, *J* = 6.9 Hz, 1H), 2.88 – 2.80 (m, 2H), 2.57 – 2.48 (m, 2H), 2.11 – 2.00 (m, 2H), 1.69 – 1.60 (m, 2H).
^{19}F NMR (376 MHz, $CDCl_3$) δ -115.2.
^{13}C NMR (101 MHz, $CDCl_3$) δ 162.6 (d, *J* = 246.7 Hz), 140.3, 137.7 (d, *J* = 3.1 Hz), 133.3 (d, *J* = 1.4 Hz), 129.1 (d, *J* = 8.0 Hz), 115.0 (d, *J* = 21.4 Hz), 34.6, 32.4, 30.0, 24.6.
HRMS (ESI+): m/z calc. for $[C_{12}H_{14}FS]^+$: 209.0795; found: 209.0789.

7-(4-chlorophenyl)-2,3,4,5-tetrahydrothiepine (16)

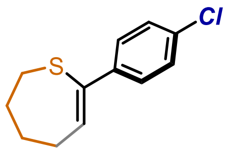

16

Synthesized following the general procedure D (0.4 mmol of **4**, 206 mg). After the purification of a fraction of 3 mL (0.3 mmol) by column chromatography on silica gel (hexane), **16** was isolated as a colorless oil (51.9 mg, 77% yield).

^{1}H NMR (400 MHz, $CDCl_3$) δ 7.54 – 7.47 (m, 2H), 7.30 – 7.23 (m, 2H), 6.41 (t, J = 6.9 Hz, 1H), 2.88 – 2.80 (m, 2H), 2.57 – 2.48 (m, 2H), 2.11 – 2.00 (m, 2H), 1.70 – 1.62 (m, 2H).
^{13}C NMR (101 MHz, $CDCl_3$) δ 140.2, 140.1, 134.0, 133.5, 128.7, 128.3, 34.5, 32.4, 30.0, 24.4.
HRMS (ESI+): m/z calc. for $[C_{12}H_{14}ClS]^+$: 225.0499; found: 225.0503.

7-(4-bromophenyl)-2,3,4,5-tetrahydrothiepine (17)

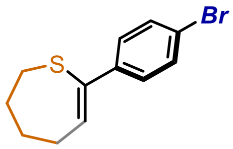

17

Synthesized following the general procedure D (0.4 mmol of **5**, 166.1 mg). After the purification of a fraction of 3 mL (0.3 mmol) by column chromatography on silica gel (hexane), **17** was isolated as a white solid (24.2 mg, 30% yield).

^{1}H NMR (400 MHz, $CDCl_3$) δ 7.48 – 7.38 (m, 4H), 6.41 (t, J = 6.9 Hz, 1H), 2.87 – 2.80 (m, 2H), 2.57 – 2.48 (m, 2H), 2.11 – 2.00 (m, 2H), 1.70 – 1.60 (m, 2H).
^{13}C NMR (101 MHz, $CDCl_3$) δ 140.6, 140.3, 134.1, 131.3, 129.1, 121.7, 34.5, 32.4, 30.0, 24.4.
HRMS (ESI+): m/z calc. for $[C_{12}H_{14}BrS]^+$: 268.9994; found: 268.5001.

7-(4-methoxyphenyl)-2,3,4,5-tetrahydrothiepine (18)

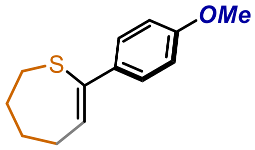

18

Synthesized following the general procedure D (0.4 mmol of **6**, 146.5 mg). After the purification of a fraction of 3 mL (0.3 mmol) by column chromatography on silica gel (hexane), **18** was isolated as a colorless oil (34.4 mg, 52% yield).

^{1}H NMR (400 MHz, $CDCl_3$) δ 7.56 – 7.48 (m, 2H), 6.89 – 6.80 (m, 2H), 6.32 (t, J = 6.9 Hz, 1H), 3.81 (s, 3H), 2.88 – 2.80 (m, 2H), 2.56 – 2.47 (m, 2H), 2.10 – 1.99 (m, 2H), 1.70 – 1.59 (m, 2H).
^{13}C NMR (101 MHz, $CDCl_3$) δ 159.4, 140.7, 134.2, 131.7, 128.6, 113.6, 55.4, 34.5, 32.4, 29.9, 24.7.
HRMS (ESI+): m/z calc. for $[C_{13}H_{17}OS]^+$: 221.0995; found: 221.0987.

7-(*p*-tolyl)-2,3,4,5-tetrahydrothiepine (19)

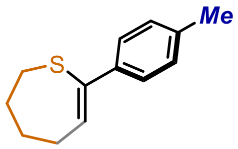

19

Synthesized following the general procedure D (0.4 mmol of **7**, 140.1 mg). After the purification of a fraction of 3 mL (0.3 mmol) by column chromatography on silica gel (hexane), **19** was isolated as a colorless oil (42.9 mg, 70% yield).

^{1}H NMR (400 MHz, $CDCl_3$) δ 7.48 (d, *J* = 8.1 Hz, 2H), 7.12 (d, *J* = 7.9 Hz, 2H), 6.39 (t, *J* = 6.9 Hz, 1H), 2.88 – 2.81 (m, 2H), 2.57 – 2.48 (m, 2H), 2.34 (s, 3H), 2.11 – 2.00 (m, 2H), 1.70 – 1.60 (m, 2H).

^{13}C NMR (101 MHz, $CDCl_3$) δ 141.1, 138.8, 137.6, 132.6, 128.9, 127.3, 34.5, 32.5, 29.9, 24.6, 21.2.

HRMS (ESI+): m/z calc. for $[C_{13}H_{17}S]^+$: 205.1045; found: 205.1046.

7-(*m*-tolyl)-2,3,4,5-tetrahydrothiepine (**20**)

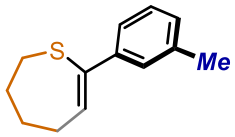

20

Synthesized following the general procedure D (0.4 mmol of **8**, 140.1 mg). After the purification of a fraction of 3 mL (0.3 mmol) by column chromatography on silica gel (hexane), **20** was isolated as a colorless oil (46.0 mg, 75% yield).

^{1}H NMR (400 MHz, $CDCl_3$) δ 7.40 (s, 1H), 7.37 (d, *J* = 7.9 Hz, 1H), 7.20 (t, *J* = 7.6 Hz, 1H), 7.08 (d, *J* = 7.6 Hz, 1H), 6.41 (t, *J* = 6.9 Hz, 1H), 2.89 – 2.81 (m, 2H), 2.58 – 2.49 (m, 2H), 2.36 (s, 3H), 2.11 – 2.01 (m, 2H), 1.71 – 1.60 (m, 2H).

^{13}C NMR (101 MHz, $CDCl_3$) δ 141.6, 141.2, 137.8, 133.9, 128.5, 128.2, 128.1, 124.5, 34.6, 32.5, 30.0, 24.6, 21.6.

HRMS (ESI+): m/z calc. for $[C_{13}H_{17}S]^+$: 205.1045; found: 205.1046

7-(*o*-tolyl)-2,3,4,5-tetrahydrothiepine (**21**)

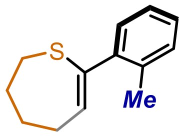

21

Synthesized following the general procedure D (0.4 mmol of **9**, 140.1 mg). After the purification of a fraction of 3 mL (0.3 mmol) by column chromatography on silica gel (hexane), **21** was isolated as a colorless oil (22.7 mg, 37% yield).

^{1}H NMR (400 MHz, $CDCl_3$) δ 7.22 – 7.08 (m, 4H), 6.03 (t, *J* = 6.6, 1.3 Hz, 1H), 2.95 – 2.87 (m, 2H), 2.55 – 2.46 (m, 2H), 2.38 (s, 3H), 2.15 – 2.04 (m, 2H), 1.80 – 1.70 (m, 2H).

^{13}C NMR (101 MHz, $CDCl_3$) δ 142.7, 140.2, 135.9, 134.6, 130.2, 129.2, 127.5, 125.6, 34.6, 32.5, 29.4, 25.1, 20.2.

HRMS (ESI+): m/z calc. for $[C_{13}H_{17}S]^+$: 205.1045; found: 205.1046

6,7-diphenyl-2,3,4,5-tetrahydrothiepine (**22**)

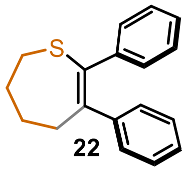

22

Synthesized following the general procedure D (0.4 mmol of **10**, 164.9 mg). After the purification of a fraction of 3 mL (0.3 mmol) by column chromatography on silica gel (hexane), **22** was isolated as a white solid (32.0 mg, 40% yield).

^{1}H NMR (400 MHz, $CDCl_3$) δ 7.23 – 7.16 (m, 2H), 7.16 – 7.01 (m, 8H), 3.10 – 3.02 (m, 2H), 3.02 – 2.94 (m, 2H), 2.11 (m, 2H), 1.63 (m, 2H).

^{13}C NMR (101 MHz, $CDCl_3$) δ 146.6, 143.9, 141.7, 136.6, 130.9, 129.4, 128.0, 127.5, 126.8, 126.3, 38.7, 35.1, 30.9, 23.6.

HRMS (ESI+): m/z calc. for $[C_{18}H_{19}S]^+$: 267.1202; found: 267.1207.

1,2,3,4-tetrahydronaphtho[2,1-b]thiepine (**23**)

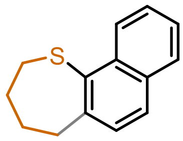

23

Synthesized following the general procedure D (0.4 mmol of **11**, 144.9 mg). After the purification of a fraction of 3 mL (0.3 mmol) by column chromatography on silica gel (hexane), **23** was isolated as a white solid (39.9 mg, 62% yield).

^{1}H NMR (400 MHz, $CDCl_3$) δ 8.69 (d, *J* = 8.5 Hz, 1H), 7.81 (d, *J* = 8.1 Hz, 1H), 7.70 (d, *J* = 8.3 Hz, 1H), 7.58 – 7.50 (m, 1H), 7.45 (t, *J* = 7.4 Hz, 1H), 7.37 (d, *J* = 8.3 Hz, 1H), 3.30 – 3.23 (m, 2H), 2.82 – 2.74 (m, 2H), 2.19 – 2.09 (m, 2H), 1.82 – 1.72 (m, 2H).
^{13}C NMR (101 MHz, $CDCl_3$) δ 146.3, 134.7, 134.3, 132.5, 128.5, 128.1, 127.9, 126.4, 126.2, 125.3, 37.4, 34.2, 32.3, 26.6.
HRMS (ESI+): m/z calc. for $[C_{14}H_{15}S]^+$: 215.0889; found: 215.0894.

1,2,3,4,6,7-hexahydronaphtho[2,1-b]thiepine (**24**)

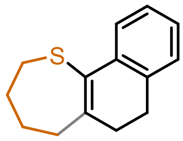

24

Synthesized following a modified general procedure D using t_R = 10 min (0.4 mmol of **11**, 144.9 mg). After the purification of a fraction of 3 mL (0.3 mmol) by column chromatography on silica gel (hexane), **24** was isolated as a colorless oil (22.1 mg, 34% yield).

^{1}H NMR (400 MHz, $CDCl_3$) δ 7.74 (d, *J* = 7.8 Hz, 1H), 7.25 – 7.17 (m, 1H), 7.15 – 7.06 (m, 2H), 2.78 (t, *J* = 8.0 Hz, 2H), 2.73 – 2.64 (m, 4H), 2.44 (t, *J* = 8.0 Hz, 2H), 2.11 – 2.00 (m, 2H), 1.64 – 1.56 (m, 2H).
^{13}C NMR (101 MHz, $CDCl_3$) δ 147.7, 136.3, 134.6, 130.1, 127.0, 126.6, 126.4, 124.6, 37.3, 33.9, 32.4, 32.1, 28.6, 24.4.
HRMS (ESI+): m/z calc. for $[C_{14}H_{17}S]^+$: 217.1045; found: 217.1052.

(*Z*)-8-phenyl-3,4,5,6-tetrahydro-2*H*-thiocine (**25**)

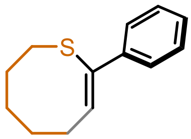

25

Synthesized following the general procedure D (0.4 mmol of **12**, 104.1 mg), After the purification of a fraction of 3 mL (0.3 mmol) by column chromatography on silica gel (hexane), **2** was isolated as a colorless oil (30.6 mg, 50% yield).

^{1}H NMR (400 MHz, $CDCl_3$) δ 7.76 – 7.48 (m, 6H), 6.78 (d, *J* = 15.0 Hz, 1H), 3.69 (m, 2H), 3.45 (m, 2H), 2.48 – 2.37 (m, 2H), 2.34 – 2.23 (m, 2H).
^{19}F NMR (376 MHz, CD_3CN) δ -72.3 (d, $J_{P\text{-}F}$ = 707.7 Hz).
^{13}C NMR (101 MHz, CD_3CN) δ 149.8, 133.8, 132.7, 130.3, 129.4, 113.7, 48.6, 29.6.
HRMS (ESI+): m/z calc. for $[C_{13}H_{17}S]^+$: 205.1045; found: 205.1044.

7-(naphthalen-2-yl)-2,3,4,5-tetrahydrothiepine (**26**)

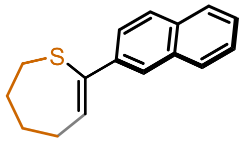

26

Synthesized following the general procedure D (0.4 mmol of **13**, 154.5 mg), After the purification of a fraction of 3 mL (0.3 mmol) by column chromatography on silica gel (hexane), **26** was isolated as a colorless oil (36.1 mg, 50% yield).

^{1}H NMR (400 MHz, $CDCl_3$) δ 8.10 (s, 1H), 8.05 – 7.66 (m, 5H), 7.58 – 7.33 (m, 2H), 6.59 (t, *J* = 6.9 Hz, 1H), 2.96 – 2.88 (m, 2H), 2.71 – 2.57 (m, 2H), 2.16 – 1.83 (m, 2H), 1.75 – 1.67 (m, 2H).

^{13}C NMR (101 MHz, $CDCl_3$) δ 141.2, 138.8, 134.2, 133.4, 133.1, 128.4, 127.7, 127.6, 126.4, 126.2, 126.0, 125.6, 34.6, 32.5, 30.1, 24.6.

HRMS (ESI+): m/z calc. for $[C_{16}H_{17}S]^+$: 241.1045; found: 241.1048.

(*S)-phenyl((*R**)-tetrahydro-2*H*-thiopyran-2-yl)methanol** (**45**)[7]

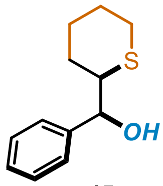

45

Synthesized following the general procedure E (0.4 mmol of **33**, 134.5 mg). After the purification of a fraction of 3 mL (0.3 mmol) by column chromatography on silica gel (hexane), **45** was isolated as a colorless oil (46.9 mg, 75% yield).

^{1}H NMR (400 MHz, $CDCl_3$) δ 7.36 (d, *J* = 4.2 Hz, 4H), 7.34 – 7.27 (m, 1H), 4.69 (d, *J* = 5.5 Hz, 1H), 3.13 – 3.03 (d, *J* = 11.0, 5.5, 2.3 Hz, 1H), 2.76 – 2.54 (m, 2H), 2.19 (bs, 1H), 2.04 (m, 1H), 1.97 – 1.82 (m, 2H), 1.64 – 1.47 (m, 2H), 1.35 – 1.20 (m, 2H).

HRMS (ESI+): m/z calc. for $[C_{12}H_{17}OS]^+$: 209.0995; found: 209.0996.

(*S)-(4-fluorophenyl)((*R**)-tetrahydro-2*H*-thiopyran-2-yl)methanol** (**46**)

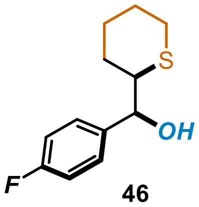

46

Synthesized following the general procedure E (0.4 mmol of **34**, 141.7 mg). After the purification of a fraction of 3 mL (0.3 mmol) by column chromatography on silica gel (hexane), **46** was isolated as a colorless oil (33.9 mg, 50% yield).

^{1}H NMR (400 MHz, $CDCl_3$) δ 7.37 – 7.28 (m, 2H), 7.09 – 6.99 (m, 2H), 4.65 (d, *J* = 5.8 Hz, 1H), 3.07 – 2.98 (m, 1H), 2.74 – 2.56 (m, 2H), 2.29 (d, *J* = 2.9 Hz, 1H), 2.08 – 1.97 (m, 1H), 1.97 – 1.82 (m, 2H), 1.62 – 1.46 (m, 2H), 1.34 – 1.20 (m, 1H).

^{19}F NMR (376 MHz, $CDCl_3$) δ -114.5.

^{13}C NMR (101 MHz, $CDCl_3$) δ 162.5 (d, *J* = 245.9 Hz), 137.4 (d, *J* = 3.2 Hz), 128.2 (d, *J* = 8.0 Hz), 115.2 (d, *J* = 21.4 Hz), 76.1, 49.4, 29.4, 28.6, 27.0, 25.8.

HRMS (ESI+): m/z calc. for $[C_{12}H_{16}FOS]^+$: 227.0900; found: 227.0904.

(*S)-(4-chlorophenyl)((*R**)-tetrahydro-2*H*-thiopyran-2-yl)methanol** (**47**)

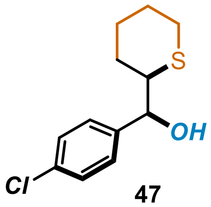

47

Synthesized following the general procedure E (0.4 mmol of **35**, 148.3 mg). After the purification of a fraction of 3 mL (0.3 mmol) by column chromatography on silica gel (hexane), **47** was isolated as a colorless oil (26.9 mg, 37% yield).

^{1}H NMR (400 MHz, $CDCl_3$) δ 7.38 – 6.94 (m, 4H), 4.66 (dd, *J* = 5.6, 2.8 Hz, 1H), 3.03 (ddd, *J* = 11.1, 5.6, 2.6 Hz, 1H), 2.82 – 2.54 (m, 2H), 2.32 (d, *J* = 2.8 Hz, 1H), 2.04 – 1.82 (m, 3H), 1.61 – 1.46 (m, 2H), 1.34 – 1.19 (m, 1H).
^{13}C NMR (101 MHz, $CDCl_3$) δ 140.1, 133.6, 128.5, 127.9, 76.0, 49.3, 29.4, 28.4, 27.0, 25.8.
HRMS (ESI+): m/z calc. for $[C_{12}H_{16}FS]^+$: 243.0605; found: 243.0611.

(*S)-(4-bromophenyl)((*R**)-tetrahydro-2*H*-thiopyran-2-yl)methanol** (**48**)

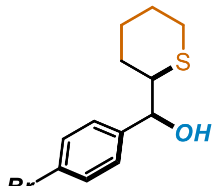

48

Synthesized following the general procedure E (0.4 mmol of **36**, 166.1 mg). After the purification of a fraction of 3 mL (0.3 mmol) by column chromatography on silica gel (hexane), **48** was isolated as a colorless oil (38.8 mg, 45% yield).

^{1}H NMR (400 MHz, $CDCl_3$) δ 7.50 – 7.43 (d, *J* = 8.3 Hz, 2H), 7.22 (d, *J* = 8.3 Hz, 2H), 4.63 (dd, *J* = 5.6, 2.9 Hz, 1H), 3.02 (ddd, *J* = 11.1, 5.6, 2.6 Hz, 1H), 2.74 – 2.56 (m, 2H), 2.36 (d, *J* = 2.9 Hz, 1H), 2.03 – 1.82 (m, 3H), 1.61 – 1.45 (m, 2H), 1.34 – 1.18 (m, 1H).
^{13}C NMR (101 MHz, $CDCl_3$) δ 140.6, 131.4, 128.2, 121.8, 76.0, 49.2, 29.4, 28.3, 27.0, 25.8.
HRMS (ESI+): m/z calc. for $[C_{12}H_{16}BrS]^+$: 287.0100; found: 287.0104.

4-((*S)-hydroxy((*R**)-tetrahydro-2*H*-thiopyran-2-yl)methyl)benzonitrile** (**49**)

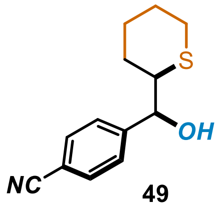

49

Synthesized following the general procedure E (0.4 mmol of **37**, 144.5 mg). After the purification of a fraction of 3 mL (0.3 mmol) by column chromatography on silica gel (hexane), **49** was isolated as a colorless oil (14.0 mg, 20% yield).

^{1}H NMR (400 MHz, $CDCl_3$) δ 7.63 (d, *J* = 8.2 Hz, 2H), 7.47 (d, *J* = 8.0 Hz, 2H), 4.74 (d, *J* = 5.1 Hz, 1H), 3.05 (ddd, *J* = 11.2, 5.1, 2.4 Hz, 1H), 2.83 – 2.62 (m, 2H), 2.49 (s, 1H), 1.89 (tdq, *J* = 10.7, 7.0, 3.8 Hz, 3H), 1.61 – 1.45 (m, 2H), 1.34 – 1.18 (m, 1H).
^{13}C NMR (101 MHz, $CDCl_3$) δ 146.9, 132.1, 127.3, 118.9, 111.6, 75.9, 49.1, 29.4, 28.0, 26.9, 25.7.
HRMS (ESI+): m/z calc. for $[C_{13}H_{16}NOS]^+$: 234.0947; found: 234.0942.

(*S)-[1,1'-biphenyl]-4-yl((*R**)-tetrahydro-2*H*-thiopyran-2-yl)methanol** (**50**)

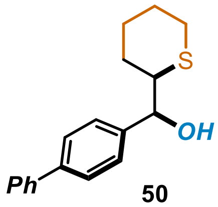

50

Synthesized following the general procedure E (0.4 mmol of **38**, 141.7 mg). After the purification of a fraction of 3 mL (0.3 mmol) by column chromatography on silica gel (hexane), **50** was isolated as a colorless oil (55.5 mg, 65% yield).

^{1}H NMR (400 MHz, $CDCl_3$) δ 7.64 – 7.56 (m, 4H), 7.48 – 7.40 (m, 4H), 7.35 (t, *J* = 7.4 Hz, 1H), 4.74 (d, *J* = 5.7 Hz, 1H), 3.13 (ddd, *J* = 11.1, 5.7, 2.6 Hz, 1H), 2.82 – 2.54 (m, 2H), 2.31 (s, 1H), 2.16 – 2.06 (m, 1H), 1.99 – 1.85 (m, 2H), 1.68 – 1.49 (m, 2H), 1.39 – 1.23 (m, 1H).

^{13}C NMR (101 MHz, $CDCl_3$) δ 140.9, 140.8, 140.7, 128.8, 127.4, 127.2, 127.1, 127.0, 76.5, 49.3, 29.4, 28.6, 27.1, 25.8.

HRMS (ESI+): m/z calc. for $[C_{18}H_{21}OS]^+$: 285.1308; found: 285.1313.

(*S)-((*R**)-tetrahydro-2*H*-thiopyran-2-yl)(*p*-tolyl)methanol** (**51**)

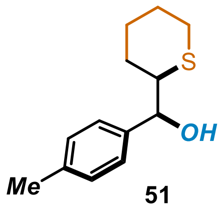

51

Synthesized following the general procedure E (0.4 mmol of **39**, 140.1 mg). After the purification of a fraction of 3 mL (0.3 mmol) by column chromatography on silica gel (hexane), **51** was isolated as a white solid (42.0 mg, 63% yield).

^{1}H NMR (400 MHz, $CDCl_3$) δ 7.25 (d, *J* = 8.0 Hz, 2H), 7.17 (d, *J* = 8.0 Hz, 2H), 4.64 (d, *J* = 6.0 Hz, 1H), 3.06 (ddd, *J* = 11.0, 6.0, 2.6 Hz, 1H), 2.74 – 2.56 (m, 2H), 2.35 (s, 3H), 2.20 – 2.05 (m, 2H), 1.97 – 1.84 (m, 2H), 1.60 – 1.47 (m, 2H), 1.35 – 1.19 (m, 1H).

^{13}C NMR (101 MHz, $CDCl_3$) δ 138.8, 137.7, 129.1, 126.5, 76.7, 49.4, 29.4, 28.9, 27.1, 25.9, 21.3.

HRMS (ESI+): m/z calc. for $[C_{13}H_{19}OS]^+$: 223.1151; found: 223.1152.

(*S)-((*R**)-tetrahydro-2*H*-thiopyran-2-yl)(*m*-tolyl)methanol** (**52**)

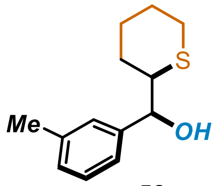

52

Synthesized following the general procedure E (0.4 mmol of **40**, 140.1 mg). After the purification of a fraction of 3 mL (0.3 mmol) by column chromatography on silica gel (hexane), **52** was isolated as a colorless oil (40.7 mg, 61% yield).

^{1}H NMR (400 MHz, $CDCl_3$) 7.24 (t, *J* = 7.5 Hz, 1H), 7.19 – 7.07 (m, 3H), 4.63 (d, *J* = 5.7 Hz, 1H), 3.06 (ddd, *J* = 11.0, 5.7, 2.6 Hz, 1H), 2.92 – 2.51 (m, 2H), 2.36 (s, 3H), 2.27 (s, 1H), 2.12 – 2.02 (m, 1H), 1.97 – 1.82 (m, 2H), 1.64 – 1.47 (m, 2H), 1.36 – 1.21 (m, 1H).

^{13}C NMR (101 MHz, $CDCl_3$) δ 141.6, 138.0, 128.7, 128.2, 127.1, 123.6, 76.7, 49.3, 29.4, 28.6, 27.1, 25.8, 21.6.

HRMS (ESI+): m/z calc. for $[C_{13}H_{19}OS]^+$: 223.1151; found: 223.1159.

(*S)-((*R**)-tetrahydro-2*H*-thiopyran-2-yl)(*o*-tolyl)methanol** (**53**)

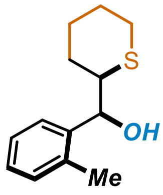

53

Synthesized following the general procedure E (0.4 mmol of **41**, 140.1 mg). After the purification of a fraction of 3 mL (0.3 mmol) by column chromatography on silica gel (hexane), **53** was isolated as a colorless oil (33.4 mg, 50% yield) as a mixture of 2 diastereoisomers (*dr* 4:1).

^{1}H NMR (400 MHz, CD_3Cl) δ 7.53 (d, *J* = 7.7 Hz, 0.25 H, minor diastereoisomer), 7.52 – 7.43 (d, *J* = 7.6 Hz, 1H, major diastereoisomer), 7.28 – 7.10 (m, 4H), 5.01 (d, *J* = 8.6 Hz, 0.25H, minor diastereoisomer), 4.92 (d, *J* = 5.3 Hz, 1H, major diastereoisomer), 3.15 – 3.02 (m, 1H, major

diastereoisomer), 2.91 – 2.82 (m, 0.25H, minor diastereoisomer), 2.80 – 2.56 (m, 3H), 2.36 (d, *J* = 1.7 Hz, 3H), 2.34 (s, 3H, major diastereoisomer), 2.16 (s, 0.75H, minor diastereoisomer), 2.12 – 2.01 (m, 2H), 1.96 – 1.85 (m, 2H), 1.77 – 1.46 (m, 3H), 1.39 – 1.14 (m, 1H).
^{13}C NMR (101 MHz, $CDCl_3$) δ 140.5, 139.8, 135.1, 134.4, 130.6, 127.7, 126.6, 126.4, 126.1, 73.0, 67.9, 47.9, 42.9, 29.6, 28.2, 27.1, 25.9, 19.5, 19.40.
HRMS (ESI+): m/z calc. for $[C_{13}H_{19}OS]^+$: 223.1151; found: 223.1156.

diphenyl(tetrahydro-2*H*-thiopyran-2-yl)methanol (**54**)

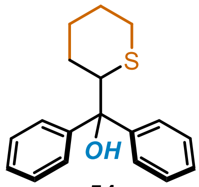

54

Synthesized following the general procedure E (0.4 mmol of **42**, 164.9 mg). After the purification of a fraction of 3 mL (0.3 mmol) by column chromatography on silica gel (hexane), **54** was isolated as a white solid (64 mg, 75% yield).

^{1}H NMR (400 MHz, $CDCl_3$) δ 7.65 – 7.58 (m, 2H), 7.53 – 7.46 (m, 2H), 7.37 – 7.25 (m, 5H), 7.25 – 7.14 (m, 2H), 3.92 (dd, *J* = 8.4, 5.7 Hz, 1H), 2.81 (ddd, *J* = 14.7, 12.3, 2.8 Hz, 1H), 2.71 – 2.61 (m, 1H), 1.90 (m, 2H), 1.70 (m, 2H), 1.56 – 1.44 (m, 2H), 1.41 – 1.23 (m, 1H).
^{13}C NMR (101 MHz, $CDCl_3$) δ 145.6, 144.4, 128.3, 128.1, 127.2, 126.7, 126.4, 125.7, 79.6, 51.4, 29.4, 28.0, 26.6, 26.2.
HRMS (ESI+): m/z calc. for $[C_{18}H_{21}OS]^+$: 285.1308; found: 285.1313.

(1*S,2*R**)-3,3',4,4',5',6'-hexahydro-1*H*-spiro[naphthalene-2,2'-thiopyran]-1-ol** (**55**)

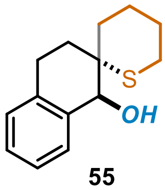

55

Synthesized following the general procedure E (0.4 mmol of **43**, 144.9 mg). After the purification of a fraction of 3 mL (0.3 mmol) by column chromatography on silica gel (hexane), **55** was isolated as a white solid (38.0 mg, 54% yield).

^{1}H NMR (400 MHz, $CDCl_3$) δ 7.45 – 7.38 (m, 1H), 7.28 – 7.17 (m, 2H), 7.15 – 7.08 (m, 1H), 4.70 (d, *J* = 4.8 Hz, 1H), 2.98 (ddd, *J* = 16.8, 10.1, 6.2 Hz, 1H), 2.77 (dt, *J* = 17.3, 5.8 Hz, 1H), 2.73 – 2.57 (m, 2H), 2.26 – 2.09 (m, 2H), 1.94 – 1.64 (m, 7H).
^{13}C NMR (101 MHz, $CDCl_3$) δ 137.0, 135.6, 129.8, 128.9, 128.0, 126.5, 72.7, 46.1, 33.8, 28.9, 27.2, 25.8, 25.7, 21.3.
HRMS (ESI+): m/z calc. for $[C_{12}H_{14}FS]^+$: 209.0795; found: 209.0789.

(*S)-(2-fluorophenyl)((*R**)-tetrahydro-2*H*-thiopyran-2-yl)methanol** (**56**)

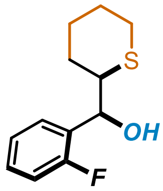

56

Synthesized following the general procedure E (0.4 mmol of **44**, 141.7 mg). After the purification of a fraction of 3 mL (0.3 mmol) by column chromatography on silica gel (hexane), **56** was isolated as a colorless oil (30.6 mg, 45% yield).

^{1}H NMR (400 MHz, $CDCl_3$) δ 7.48 (d, *J* = 7.5, 1H), 7.33 – 7.22 (m, 1H), 7.15 (t, *J* = 7.5 Hz, 1H), 7.07 – 6.98 (m, 1H), 5.01 (d, *J* = 5.7 Hz, 1H), 3.21 – 3.12 (m, 1H), 2.94 – 2.54 (m, 2H), 2.10 – 1.95 (m, 1H), 1.96 – 1.81 (m, 2H), 1.68 – 1.49 (m, 2H), 1.37 – 1.23 (m, 1H).

^{19}F NMR (376 MHz, $CDCl_3$) δ -115.6.

^{13}C NMR (101 MHz, $CDCl_3$) 160.04 (d, *J* = 245.6 Hz), 129.33 (d, *J* = 8.3 Hz), 128.68 (d, *J* = 12.8 Hz), 128.43 (d, *J* = 4.4 Hz), 124.10 (d, *J* = 3.5 Hz), 115.38 (d, *J* = 21.8 Hz), 70.88 (d, *J* = 1.8 Hz), 47.93, 29.20, 28.47, 27.08, 25.59.

HRMS (ESI+): m/z calc. for $[C_{12}H_{16}FOS]^+$: 227.0900; found: 227.0904.

(R*)-2-((S*)-methoxy(phenyl)methyl)tetrahydro-2H-thiopyran (**57**)

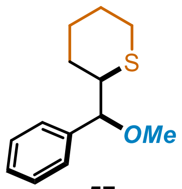

57

Synthesized following the general procedure E (0.4 mmol of **33**, 134.5 mg). After the purification of a fraction of 3 mL (0.3 mmol) by column chromatography on silica gel (hexane), **57** was isolated as a colorless oil (46.7 mg, 70% yield).

^{1}H NMR (400 MHz, $CDCl_3$) δ 7.41 – 7.27 (m, 5H), 4.07 (d, *J* = 7.0 Hz, 1H), 3.24 (s, 3H), 3.05 – 2.95 (m, 1H), 2.67 – 2.52 (m, 2H), 2.28 – 2.17 (m, 1H), 1.95 – 1.83 (m, 1H), 1.64 – 1.48 (m, 0H), 1.30 (q, *J* = 13.1 Hz, 1H).

^{13}C NMR (101 MHz, $CDCl_3$) δ 139.4, 128.2, 128.1, 127.4, 86.6, 57.3, 48.6, 30.1, 29.5, 27.3, 26.0.

HRMS (ESI+): m/z calc. for $[C_{13}H_{19}OS]^+$: 223.1151; found: 223.1147.

(*R)-2-((*S**)-(cyclohexyloxy)(phenyl)methyl)tetrahydro-2*H*-thiopyran** (**58**)

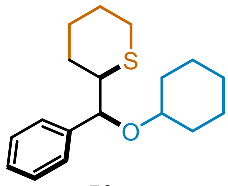

58

Synthesized following the general procedure E (0.4 mmol of **33**, 134.5 mg). After the purification of a fraction of 3 mL (0.3 mmol) by column chromatography on silica gel (hexane), **58** was isolated as a colorless oil (63.6 mg, 73% yield).

^{1}H NMR (400 MHz, $CDCl_3$) δ 7.33 (h, *J* = 7.4 Hz, 5H), 4.11 (d, *J* = 7.7 Hz, 1H), 3.37 – 3.28 (m, 1H), 3.28 – 3.18 (m, 1H), 2.99 (ddd, *J* = 10.4, 7.7, 2.6 Hz, 1H), 2.65 – 2.50 (m, 1H), 2.39 – 2.28 (m, 1H), 1.95 – 1.83 (m, 1H), 1.64 – 1.49 (m, 3H), 1.42 – 1.19 (m, 2H), 0.92 – 0.84 (m, 3H).

^{13}C NMR (101 MHz, CD_3CN) δ ^{13}C NMR (101 MHz, $CDCl_3$) δ 140.4, 128.2, 128.0, 127.5, 84.9, 69.5, 48.6, 31.7, 30.6, 29.8, 29.5, 27.3, 25.9, 25.9, 22.7, 14.2.

HRMS (ESI+): m/z calc. for $[C_{18}H_{27}OS]^+$: 291.1777; found: 291.1783.

4-methyl-*N*-((*S)-phenyl((*R**)-tetrahydro-2*H*-thiopyran-2-yl)methyl)aniline** (**59**)

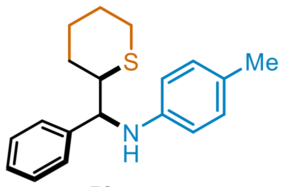

59

Synthesized following the general procedure E (0.4 mmol of **33**, 134.5 mg). After the purification of a fraction of 3 mL (0.3 mmol) by column chromatography on silica gel (hexane), **59** was isolated as a colorless oil (44.6 mg, 50% yield).

^{1}H NMR (400 MHz, $CDCl_3$) δ 7.50 – 7.31 (m, 4H), 7.31 – 7.22 (m, 1H), 6.93 – 6.86 (d, *J* = 8.2 Hz, 2H), 6.43 (d, *J* = 8.2 Hz, 2H), 4.44 (d, *J* = 4.5 Hz, 1H), 3.26 – 3.17 (m, 1H), 2.87 – 2.71 (m, 1H), 2.70 – 2.61 (m, 1H), 2.19 (s, 3H), 1.99 – 1.84 (m, 3H), 1.62 – 1.43 (m, 2H), 1.35 – 1.19 (m, 1H).

^{13}C NMR (101 MHz, $CDCl_3$) δ 145.1, 140.7, 129.7, 129.6, 128.4, 127.4, 126.7, 113.8, 113.7, 62.4, 49.6, 29.9, 29.5, 27.3, 26.7, 20.5, 20.4.

HRMS (ESI+): m/z calc. for $[C_{19}H_{24}NS]^+$: 298.1624; found: 298.1632.

(*R)-2-((*S**)-chloro(phenyl)methyl)tetrahydro-2*H*-thiopyran** (**60**)

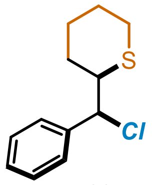

60

Synthesized following the general procedure E (0.4 mmol of **33**, 134.5 mg). After the purification of a fraction of 3 mL (0.3 mmol) by column chromatography on silica gel (hexane), **60** was isolated as a colorless oil (22.9 mg, 31% yield).

^{1}H NMR (400 MHz, $CDCl_3$) δ 7.55 – 7.27 (m, 5H), 4.69 (d, *J* = 5.6 Hz, 1H), 3.08 (ddd, *J* = 11.0, 5.6, 2.6 Hz, 1H), 2.79 – 2.48 (m, 2H), 2.09 – 2.00 (m, 1H), 2.03 – 1.82 (m, 1H), 1.64 – 1.48 (m, 2H), 1.35 – 1.18 (m, 2H).

^{13}C NMR (101 MHz, $CDCl_3$) δ 141.7, 128.4, 128.0, 126.5, 76.7, 49.4, 29.4, 28.5, 27.1, 25.89.

HRMS (ESI+): m/z calc. for $[C_{12}H_{16}ClS]^+$: 227.0656; found: 227.0656.

(*R)-2-((*S**)-bromo(phenyl)methyl)tetrahydro-2*H*-thiopyran** (**61**)

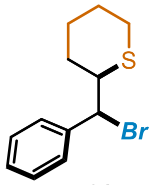

61

Synthesized following the general procedure E (0.4 mmol of **33**, 134.5 mg). After the purification of a fraction of 3 mL (0.3 mmol) by column chromatography on silica gel (hexane), **61** was isolated as a colorless oil (15.5 mg, 21% yield) as a mixture of 2 diastereoisomers (*dr* 4:1).

^{1}H NMR (400 MHz, $CDCl_3$) δ 7.41 – 7.29 (m, 6H), 4.69 (d, *J* = 5.6 Hz, 1H, major diastereoisomer), 4.66 (d, *J* = 8.7 Hz, 0.20H, minor diastereoisomer), 3.08 (ddd, *J* = 11.1, 5.6, 2.6 Hz, 1H, major diastereoisomer), 2.95 (td, *J* = 8.7, 2.9 Hz, 0.20H, minor diastereoisomer), 2.86 – 2.49 (m, 1H), 2.09 – 1.99 (m, 0.65H), 1.96 – 1.82 (m, 1H), 1.76 – 1.68 (m, 2H), 1.64 – 1.46 (m, 6H), 1.44 – 1.18 (m, 1H).

^{13}C NMR (101 MHz, $CDCl_3$) δ 141.7, 128.6, 128.4, 128.0, 126.9, 126.5, 76.8, 49.8, 49.4, 29.5, 29.4, 28.5, 27.1, 27.0, 27.0, 25.9, 24.0.

HRMS (ESI+): m/z calc. for $[C_{12}H_{16}BrS]^+$: 271.0151; found: 271.0154.

(*R)-2-((*S**)-3-methyl-1-phenylbut-3-en-1-yl)tetrahydro-2*H*-thiopyran** (**62**)

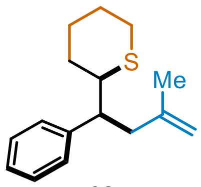

62

Synthesized following the general procedure E (0.4 mmol of **33**, 134.5 mg). After the purification of a fraction of 3 mL (0.3 mmol) by column chromatography on silica gel (hexane), **62** was isolated as a colorless oil (18.9 mg, 23% yield).

^{1}H NMR (400 MHz, $CDCl_3$) δ 7.41 – 7.24 (m, 2H), 7.28 – 7.08 (m, 3H), 4.65 (d, *J* = 2.4 Hz, 1H), 4.60 (d, *J* = 2.4 Hz, 1H), 3.01 – 2.86 (m, 2H), 2.72 – 2.50 (m, 3H), 2.44 (dd, *J* = 14.2, 9.7 Hz, 1H), 2.11 – 2.02 (m, 1H), 1.94 – 1.81 (m, 2H), 1.63 (s, 3H), 1.54 – 1.20 (m, 3H).

^{13}C NMR (101 MHz, $CDCl_3$) δ 143.6, 141.8, 128.8, 127.9, 126.7, 112.6, 49.2, 48.7, 40.2, 32.6, 30.0, 27.4, 26.9, 22.5.

HRMS (ESI+): m/z calc. for $[C_{16}H_{23}S]^+$: 247.1515; found: 247.1514.

(*S)-2-((*S**)-phenyl((*R**)-tetrahydro-2*H*-thiopyran-2-yl)methyl)cyclopentan-1-one** (**63**)

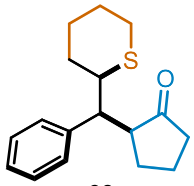

63

Synthesized following the general procedure E (0.4 mmol of **33**, 134.5 mg). After the purification of a fraction of 3 mL (0.3 mmol) by column chromatography on silica gel (hexane), **63** was isolated as a colorless oil (20.6 mg, 25% yield) as mixture of 2 diastereoisomers (*dr* 2:1).

^{1}H NMR (400 MHz, $CDCl_3$) δ 7.33 – 7.14 (m, 15H), 3.60 – 3.45 (m, 1H), 3.17 (t, *J* = 7.0 Hz, 2H), 2.93 (dd, *J* = 10.0, 4.8 Hz, 4H), 2.83 – 2.73 (m, 1H), 2.72 – 2.44 (m, 4H), 2.34 – 2.10 (m, 2H), 2.07 – 1.60 (m, 8H), 1.59 – 1.33 (m, 4H).

^{13}C NMR (101 MHz, $CDCl_3$) δ 141.3, 139.0, 130.1, 129.0, 129.0, 128.4, 127.8, 127.1, 127.0, 51.7, 50.9, 50.6, 50.2, 45.3, 44.8, 39.5, 38.9, 33.4, 33.4, 29.8, 29.7, 27.6, 27.4, 27.3, 27.3, 26.6, 26.4, 20.8, 20.5.

HRMS (ESI+): m/z calc. for $[C_{17}H_{23}OS]^+$: 275.1464; found: 275.1462.

6. Product derivatizations. Synthesis and characterization of 64-68

(1*R*,2*R*)-2-((*S*)-hydroxy(phenyl)methyl)tetrahydro-2*H*-thiopyran 1-oxide (**64**) and **(1*S*,2*R*)-2-((*S*)-hydroxy(phenyl)methyl)tetrahydro-2*H*-thiopyran 1-oxide** (**65**)

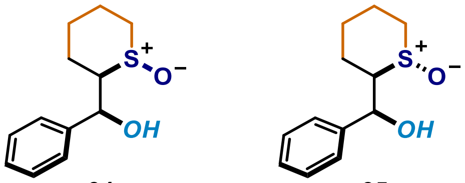

64 **65**

Following a prodecure reported by Magnier and co-workers,[8] *m*-CPBA 77% w/w (74.0 mg, 0.33 mmol, 1 equiv.) was added portionwise to a solution of sulfide **45** (69.0 mg, 0.33 mmol, 1 equiv.) in CH_2Cl_2 (0.56 M). The resulting suspension was then vigorously stirred for 1 hour at room temperature. The crude reaction mixture was filtrated over a pad of Celite® and rinsed with CH_2Cl_2 (5-10 mL). The filtrate was then washed with saturated aqueous solution of $NaHCO_3$. After extraction of the aqueous layer with CH_2Cl_2 (x2 times), the combined organic phases were dried over anhydrous $MgSO_4$, and the solvent was removed under reduced pressure. Purification by flash column chromatography (EtOAc) afforded sulfoxides **64** (15.7 mg, 21%) and **65** (20.8 mg, 28%) as white solids.

*Characterization of compound **64***

^{1}H NMR (400 MHz, $CDCl_3$) δ 7.48 – 7.21 (m, 5H), 5.31 (d, *J* = 3.4 Hz, 1H), 3.87 (s, 1H), 3.06 (dt, *J* = 14.0, 2.2 Hz, 1H), 2.55 – 2.12 (m, 4H), 1.93 – 1.63 (m, 3H), 1.38 – 1.23 (m, 1H).
^{13}C NMR (101 MHz, $CDCl_3$) δ 140.9, 128.5, 127.9, 126.2, 75.6, 60.6, 47.0, 24.6, 15.7, 15.4.
HRMS (ESI+): m/z calc. for $[C_{12}H_{17}O_2S]^+$: 225.0904; found: 225.0908.

*Characterization of compound **65***

^{1}H NMR (400 MHz, $CDCl_3$) δ 7.44 – 7.22 (m, 5H), 5.57 – 5.51 (m, 1H), 4.71 (d, *J* = 5.1 Hz, 1H), 3.41 (dt, *J* = 12.0, 3.6 Hz, 1H), 2.74 (dt, *J* = 12.4, 2.4 Hz, 1H), 2.63 (ddd, *J* = 14.3, 11.8, 2.9 Hz, 1H), 1.99 (ddd, *J* = 18.2, 7.0, 3.7 Hz, 1H), 1.78 – 1.46 (m, 4H), 1.19 (dddd, *J* = 22.5, 13.1, 9.6, 4.4 Hz, 1H).
^{13}C NMR (101 MHz, $CDCl_3$) δ 141.3, 128.4, 127.4, 126.3, 71.5, 68.9, 51.62, 3.15, 23.3, 22.0.
HRMS (ESI+): m/z calc. for $[C_{12}H_{17}O_2S]^+$: 225.0904; found: 225.0907.

(*R*)-2-((*S*)-hydroxy(phenyl)methyl)tetrahydro-2*H*-thiopyran 1,1-dioxide (**66**)

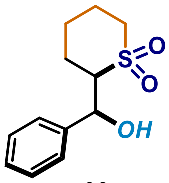

66

Following a prodecure reported by Quallich and co-workers,[9] oxone® (187.0 mg, 0.61 mmol, 2.6 equiv.) was added portionwise to a solution of sulfide **45** (53.0 mg, 0.23 mmol, 1 equiv.) in acetone (0.2 M) and H_2O (0.2 M). Te resulting suspension was vigorously stirred for 40 min at room temperature. Thereafter, the organic phase was separed, dried over anhydrous $MgSO_4$, and the solvent was removed under reduced pressure. Purification by flash column chromatography (hexane/EtOAc 8:2) afforded sulfone **66** (31.5 mg, 57%).

^{1}H NMR (400 MHz, $CDCl_3$) δ 7.41 – 7.27 (m, 5H), 5.80 (s, 1H), 3.25 – 3.14 (m, 2H), 3.02 – 2.87 (m, 2H), 2.25 – 2.03 (m, 3H), 1.97 – 1.79 (m, 2H), 1.37 – 1.21 (m, 1H).
^{13}C NMR (101 MHz, $CDCl_3$) δ 139.7, 128.6, 127.9, 125.9, 67.7, 67.2, 53.1, 24.4, 23.8, 22.2.
HRMS (ESI+): m/z calc. for $[C_{12}H_{17}O_2S]^+$: 241.0893; found: 241.0890.

7-phenyl-2,3,4,5-tetrahydrothiepine 1,1-dioxide (**67**)

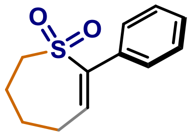

67

Following a prodecure reported by Quallich and co-workers,[9] oxone® (164.0 mg, 0.53 mmol, 2.5 equiv.) was added portionwise to a solution of sulfide **45** (42.0 mg, 0.22 mmol, 1 equiv.) in acetone (0.2 M) and H_2O (0.2 M). Te resulting suspension was vigorously stirred for 40 min at room temperature. Thereafter, the organic phase was separed, dried over anhydrous $MgSO_4$, and the solvent was removed under reduced pressure. Purification by flash column chromatography (hexane/EtOAc 7:3) afforded sulfone **67** (34.7 mg, 71%).

^{1}H NMR (400 MHz, $CDCl_3$) δ 7.36 – 7.34 (m, 5H), 6.60 (t, *J* = 7.6 Hz, 1H), 3.32 – 3.24 (m, 2H), 2.71 (dd, J = 11.4, 7.5 Hz, 2H), 2.28 – 2.18 (m, 2H), 1.81 (dd, J = 11.3, 5.7 Hz, 2H).
^{13}C NMR (101 MHz, $CDCl_3$) δ 148.6, 141.2, 134.4, 129.3, 128.9, 128.3, 56.0, 27.5, 24.4, 24.3.
HRMS (ESI+): m/z calc. for $[C_{12}H_{15}O_2S]^+$: 223.0787; found: 223.0790.

(5a*R*,5b*R*,10a*R*,10b*R*)-10a,10b-di(naphthalen-2-yl)dodecahydrocyclobuta[1,2-b:4,3-b']bis(thiepine) (**68**)

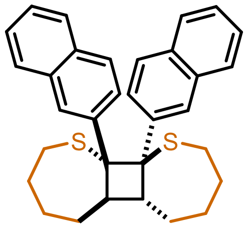

68

^{1}H NMR (400 MHz, $CDCl_3$) δ 8.42 (s, 2H), 8.12 (dd, *J* = 8.7, 2.0 Hz, 2H), 7.97 – 7.84 (m, 4H), 7.42 – 7.55 (m, 6H), 4.11 – 3.93 (m, 2H), 2.41 (dd, *J* = 14.4, 6.7 Hz, 2H), 2.46 – 2.31 (m, 2H), 2.19 – 1.87 (m, 8H), 1.73 – 1.42 (m, 4H).

^{13}C NMR (101 MHz, $CDCl_3$) δ 139.5, 134.2, 133.1, 132.6, 128.7, 128.4, 127.7, 127.1, 126.1, 126.1, 64.6, 56.9, 33.7, 30.8, 28.6, 27.2.

HRMS (ESI+): m/z calc. for $[C_{32}H_{33}S_2]^+$:481.2018; found: 481.2020.

7. Mechanistic investigations

Redox properties of starting materials and products

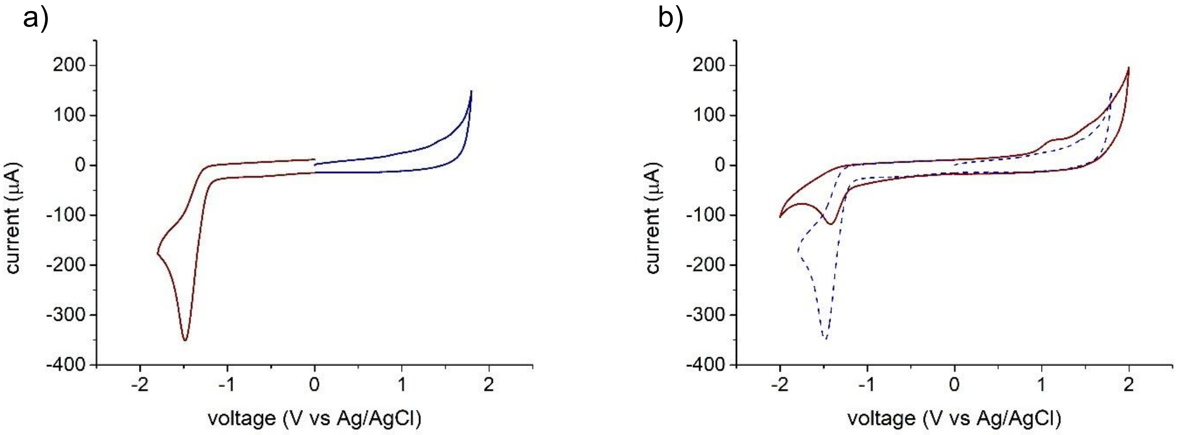

Figure S8. *a) oxidation (blue trace) and reduction (red trace) cyclic voltammetry of **1** (5mM, 0.1M TBAPF$_6$, scan rate 100 mV/s); b) full cyclic voltammetry of **1** starting with oxidation (dashed blue trace) and reduction (straight red trace), (5mM, 0.1M TBAPF$_6$, scan rate 100 mV/s).*

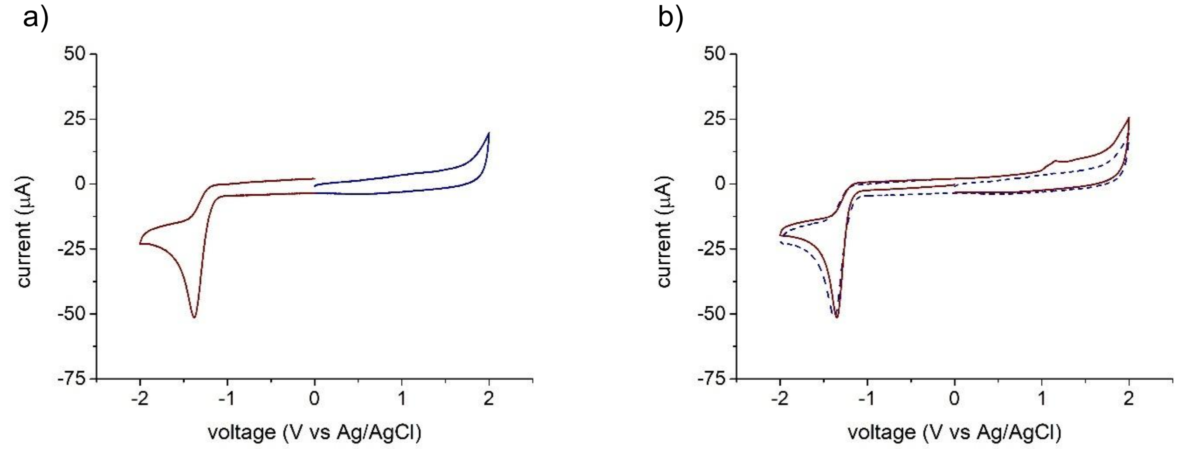

Figure S9. *a) oxidation (blue trace) and reduction (red trace) cyclic voltammetry of **33**, (5mM, 0.1M TBAPF$_6$, scan rate 100 mV/s); b) full cyclic voltammetry of **33** starting with oxidation (dashed blue trace) and reduction (straight red trace), (5mM, 0.1M TBAPF$_6$, scan rate 100 mV/s).*

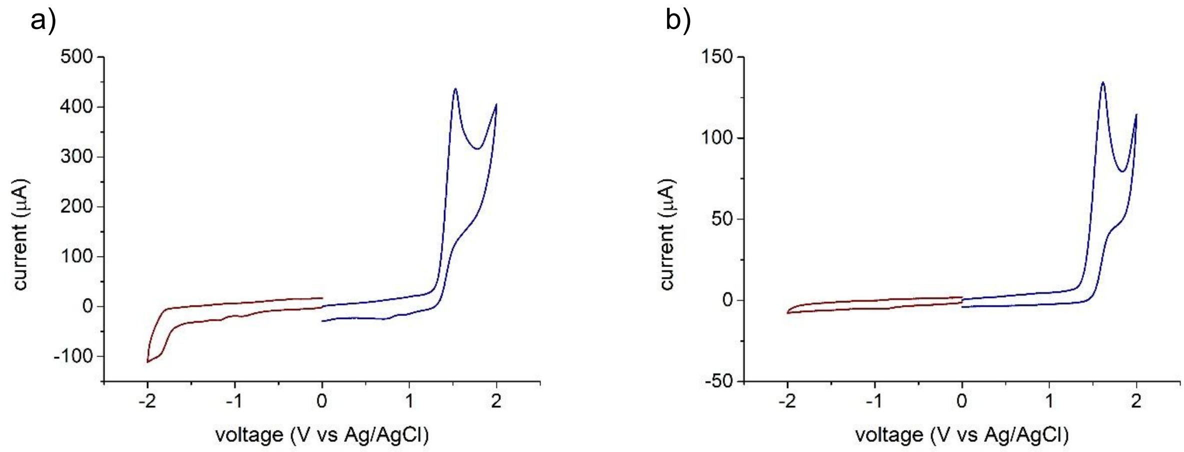

Figure S10. *a) oxidation (blue trace) and reduction (red trace) cyclic voltammetry of **2**, (5mM, 0.1M $TBAPF_6$, scan rate 1000 mV/s); b) oxidation (blue trace) and reduction (red trace) cyclic voltammetry of **45**, (5mM, 0.1M $TBAPF_6$, scan rate 100 mV/s).*

Radical trapping: evidence of intermediate I

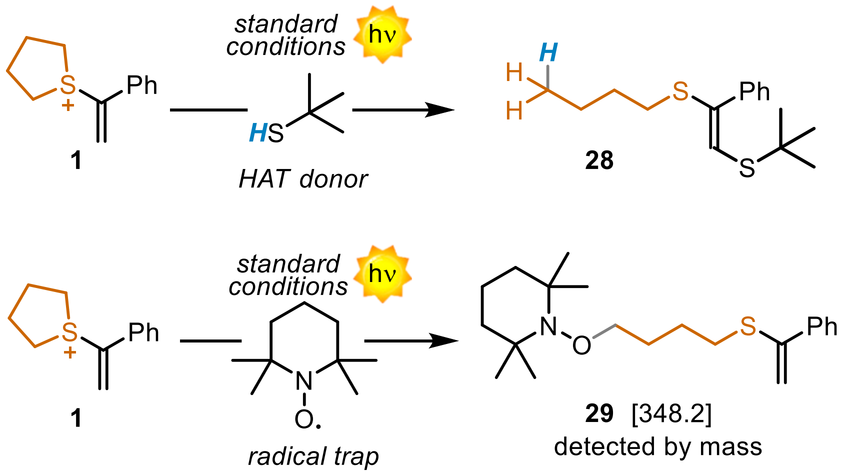

Compound **28** (33% NMR yield, 30% for the isolated compound) was obtained following general procedure A for the photocatalytic ring expansion of sulfonium salts in batch (from 0.2 mmol of **1**), using 2-methyl-2-propanethiol (5 equiv.) as HAT donor.

Compound **29** was detected by UPC^2 analysis from the crude reaction mixture following general procedure A for the photocatalytic ring expansion of sulfonium salts in batch (from 0.1 mmol of **1**), using TEMPO (1 equiv.) as radical trap.

Alternative reaction products: evidence of intermediate III

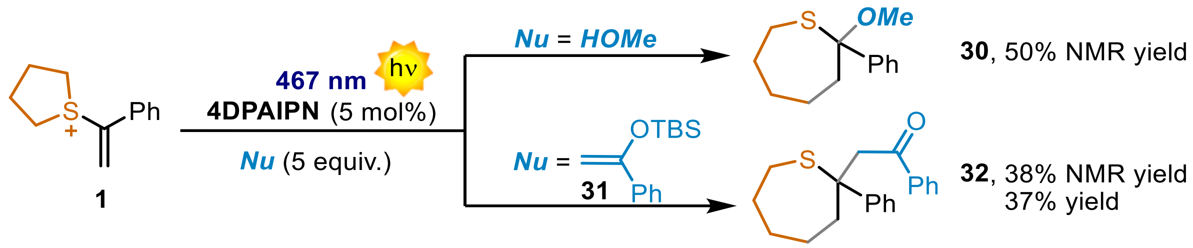

Compound **30** (50% NMR yield) was detected from the reaction mixture general procedure A for the photocatalytic ring expansion of sulfonium salts in batch (from 0.1 mmol of **1**), using a 4:1 mixture of MeCN : MeOH as solvent. **Note**: 8% of **2** was also detected under these conditions. Compound **30** was not possible to purify most likely due to decomposition in the column chromatography. NMR yield was measured using dibromomethane (0.5 equiv.) and EtOAc (0.5 equiv.) as i.s. The mass of the product was confirmed by UPC^2 analysis from the reaction mixture.

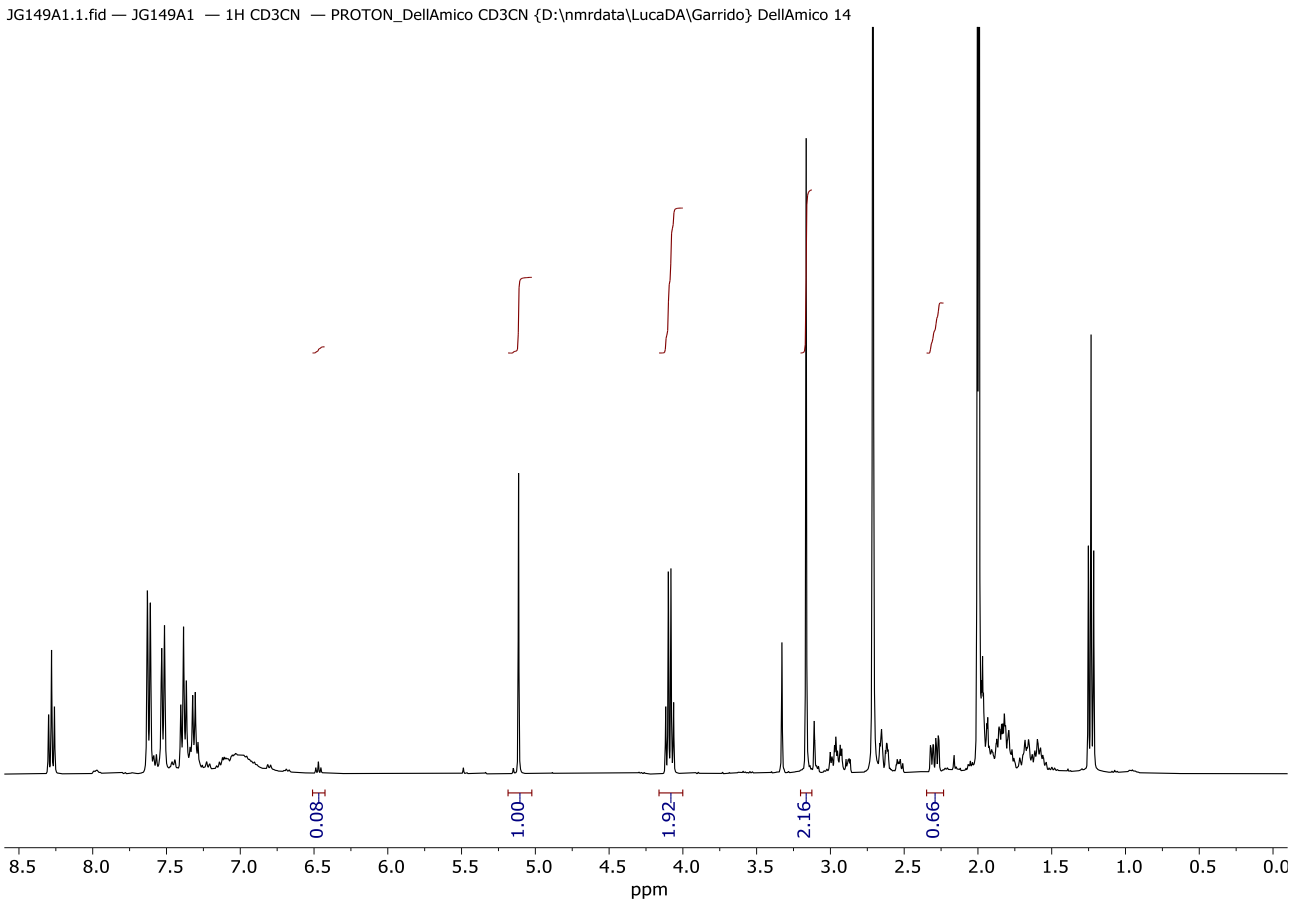

Figure S11. *^{1}H NMR spectrum of the reaction mixture using MeOH as co-solvent. Dibromomethane (0.05 mmol) and EtOAc (0.05 mmol) were used as i.s.*

Compound **32** (38% NMR yield, 37% for the isolated compound) was obtained following general procedure A for the photocatalytic ring expansion of sulfonium salts in batch (from 0.1 mmol of **1**), using

31 (5 equiv.) as nucleophile. **Note**: Compound **31** was prepared following a procedure reported by Jiao and co-workers.[10]

^{1}H NMR (400 MHz, $CDCl_3$) δ 7.77 (d, *J* = 7.1 Hz, 2H), 7.58 (d, *J* = 8.7 Hz, 2H), 7.51 – 7.44 (m, 1H), 7.34 (t, *J* = 7.7 Hz, 2H), 7.31 – 7.22 (m, 2H), 7.20 – 7.12 (m, 1H), 3.72 (d, *J* = 15.8 Hz, 1H), 3.55 (d, *J* = 15.8 Hz, 1H), 2.95 (dd, *J* = 14.9, 7.9 Hz, 1H), 2.75 – 7.65 (m, 1H), 2.47 (ddd, *J* = 15.6, 8.7, 3.0 Hz, 1H), 2.20 (ddd, *J* = 15.5, 9.8, 1.7 Hz, 1H), 1.96 – 1.81 (m, 2H), 1.81 – 1.58 (m, 4H).

^{13}C NMR (101 MHz, $CDCl_3$) δ 197.4, 144.4, 137.8, 133.3, 128.5, 128.3, 128.3, 127.5, 126.6, 54.9, 52.8, 40.7, 31.1, 29.3, 29.0, 23.5.

HRMS (ESI+): m/z calc. for $[C_{20}H_{23}OS]^+$: 311.1464; found: 311.1466.

Quenching experiments: Stern-Volmer

The analysis was performed at room temperature on a Varian Cary Eclipse Fluorescence spectrophotometer. Samples were degassed through repeated freeze-pump-thaw cycles until no gas bubbles evolved upon thawing (usually 3 cycles) and introduced into a 1 cm cuvette fitted with a septum. Excitation was performed at 450 nm. **33** was added as a 0.1 M solution in MeCN. The trend observed in the Stern-Volmer equation is in agreement with the latest results reported by Limburg regarding TDAF photocatalysts, where both the quenching of fluorescence and delayed fluorescence are considered.[11]

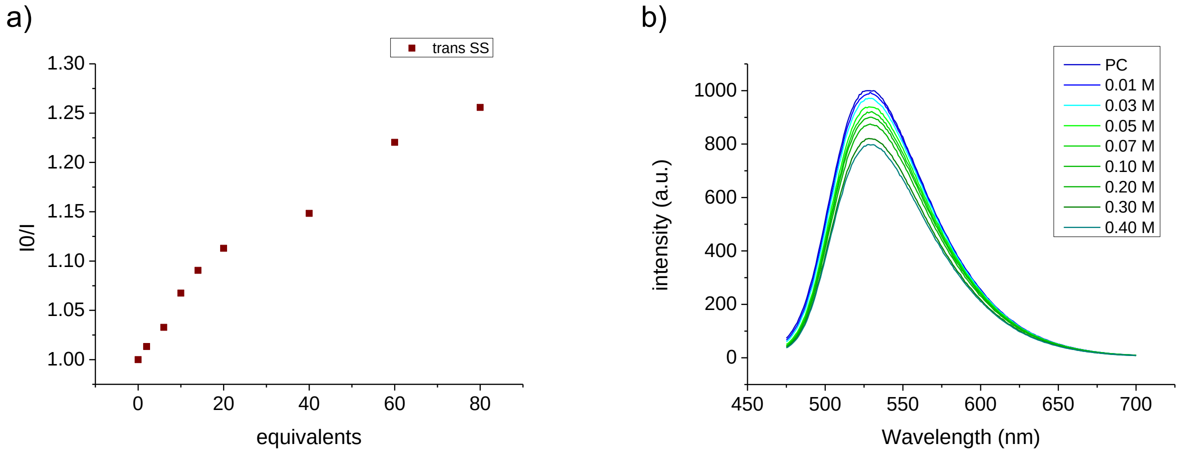

Figure S12. *a) Stern-Volmer plot of **4DPAIPN** in MeCN using **33** as quencher; b) Fluorescence emission quenching of **4DPAIPN** (5μM) using **33** as quencher.*

8. Assignation of the relative configuration of 45 and 55

Compound **45** is described in the literature[7] and compared to its diastereoisomer. To assign the relative configuration of the centers, we compared the chemical shift of the compounds as well as the coupling constants.

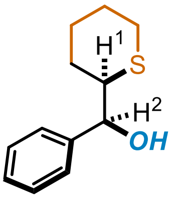

Described

H^1: 2.95 ppm (ddd, J = 8.5, 8.5, 3.1 Hz)

H^2: 4.86 ppm (d, J = 8.5 Hz)

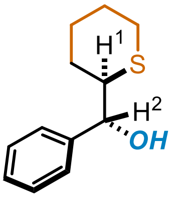

Described

H^1: 3.08 ppm (ddd, J = 11.0, 5.5, 2.4 Hz)

H^2: 4.69 ppm (d, J = 5.5 Hz)

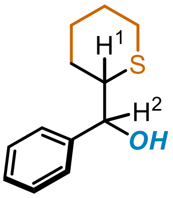

Observed

H^1: 3.08 ppm (ddd, J = 11.0, 5.5, 2.3 Hz)

H^2: 4.69 ppm (d, J = 5.5 Hz)

To assign the relative configuration in compound **55** we first performed a full NMR characterization to assign the corresponding protons to the structure. Then, we performed a NOESY to determined which diastereoisomer was obtained. As shown below, depending on the diastereoisomer, the characteristic proton at 4.70 ppm (H_a) will interact with two different proton systems. From the NOESY experiment we found an interaction between the H at 4.70 ppm and two other protons at 2.6 ppm and 1.8 ppm which correspond to H_e and H_d respectively. Given the assignation performed (see the NMR spectra below) we assigned the relative configuration as (1*S**, 2*R**).

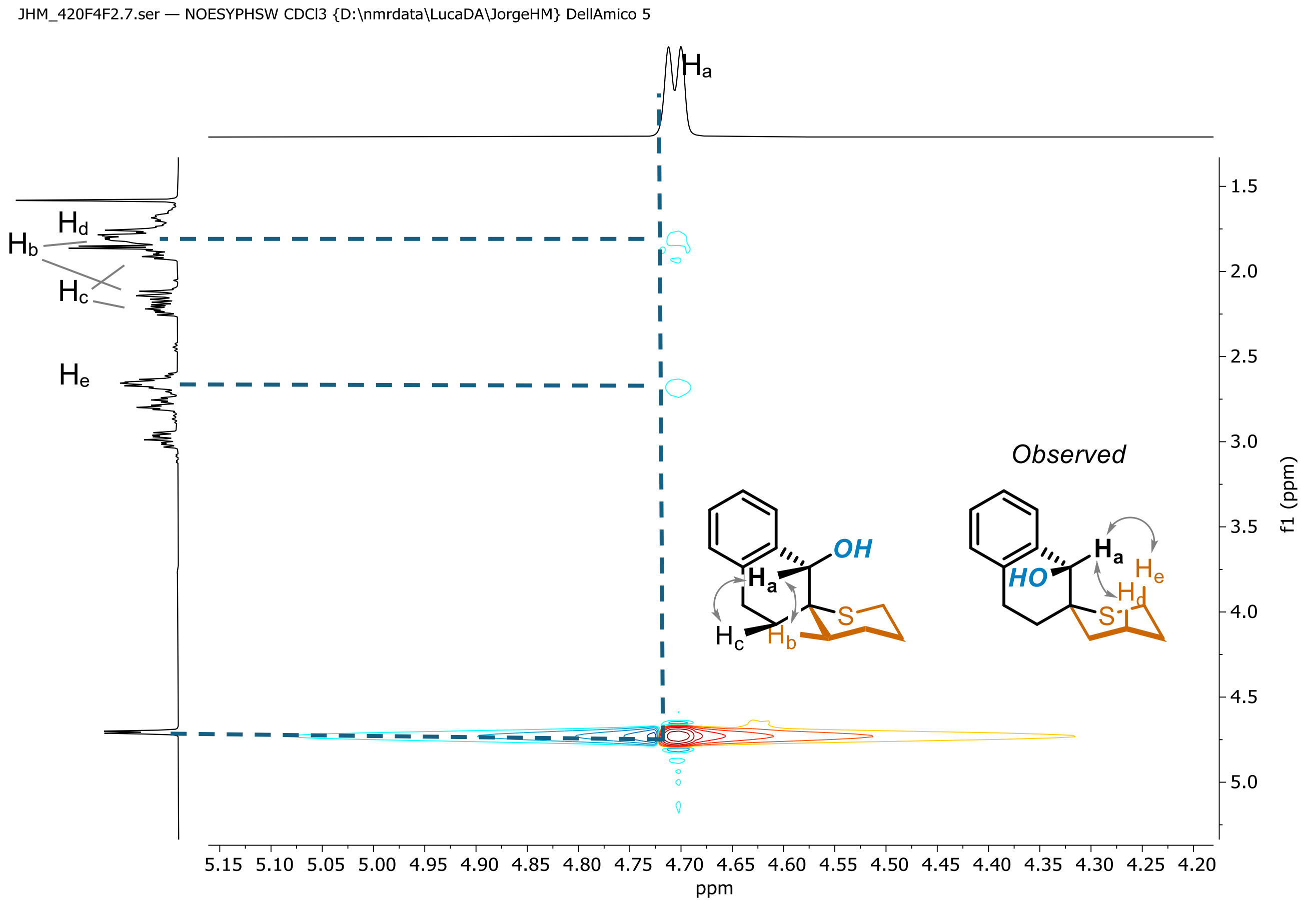

Figure S13. *NOESY spectrum expansion of compound* ***55****, employed to assign the relative configuration.*

9. DFT calculations

All the calculations were performed with the GAUSSIAN16 suite of programs (Revision C.01). Each geometry was optimized with default convergence thresholds (RMS values of 3x10-4 hartree/bohr and 1.2x10-3 bohr and maximum values of 4.5x10-4 hartree/bohr and 1.8x10-3 bohr on forces and displacements, respectively) at M062X/Def2TZVP level of theory. Polarization effects were taken into account with the polarizable continuum model (PCM).

Given the high diastereoselectivity observed in the presented transformation for the *trans* SSs and based on previous reports,[12] we hypothesized that the sulfur atom could be interacting with the *in situ* generated carbocation, generating a thiiranium intermediate with high diastereoselectivity. To further prove this, we decided to perform some DFT calculations in order to gain some insight into the geometry of intermediate type **III**. To our delight, we observed that no matter the initial coordinates we introduced, we always observed the formation of a thiiranium cation. At this point, we compared the relative energy of the two different possible diastereoisomers of the bicyclic [4.1.0] sulfonium salt that would lead to the two possible diastereoisomers of the product. Interestingly, The relative energy of **A** is 6.0 kcal/mol higher than **B**, what could suggest that the equilibrium is displaced towards **B**.

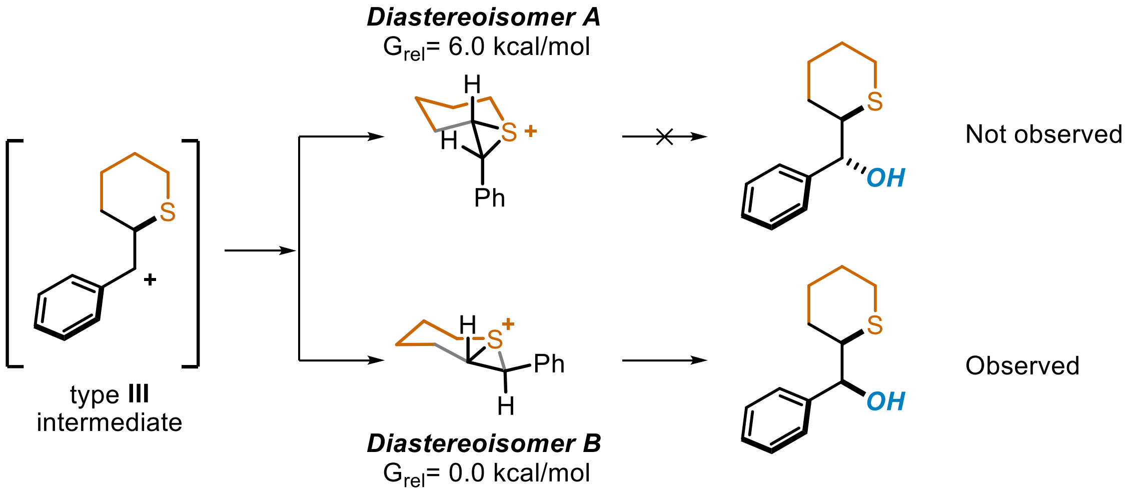

Optimized geometries and SCF energies

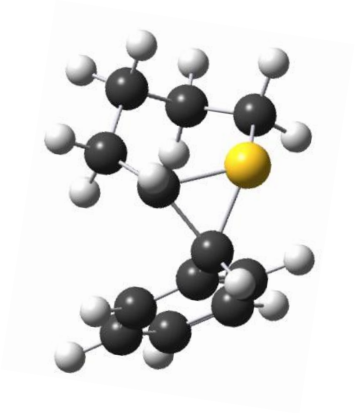

Diastereoisomer A SCF energy: -864.280010 Hartree

EE + Thermal Free Energy Correction: -864.073313 Hartree

Cation singlet, total number of atoms: 28

C -2.14421300 1.27259200 1.16511500

C -1.67543600 1.67923500 -0.22964700

C -1.52359900 0.54602100 -1.22763700

S -1.70808600 -1.20593000 -0.64290900

C -1.45494500 -1.15785700 1.15670400

C -1.23717700 0.21547700 1.77337900

C -0.31708300 -0.28866800 -1.42147300

C 0.97211600 -0.13277200 -0.68993900

C 1.61575300 1.10268900 -0.70566400

C 2.84617400 1.25128200 -0.08106900

C 3.43976100 0.17043600 0.55821500

C 2.81041000 -1.06795900 0.55313300

C 1.58332100 -1.22216600 -0.07603900

H -2.14351800 2.16390000 1.79141200

H -3.17289000 0.90310000 1.12413700

H -2.38233900 2.38337200 -0.66452100

H -0.71697900 2.18974400 -0.13784300

H -2.13097500 0.63584600 -2.12062700

H -0.64803700 -1.85793000 1.36726700

H -2.39143300 -1.59427000 1.51036000

H -1.44605500 0.09721200 2.83760600

H -0.19217900 0.51610200 1.67719000

H -0.21727700 -0.68644600 -2.42817500

H 1.16316100 1.94194700 -1.21840400

H 3.34132400 2.21372700 -0.09687400

H 4.39600100 0.29060400 1.05130500

H 3.27605900 -1.91773200 1.03552000

H 1.10402000 -2.19416800 -0.09227300

Diastereoisomer B

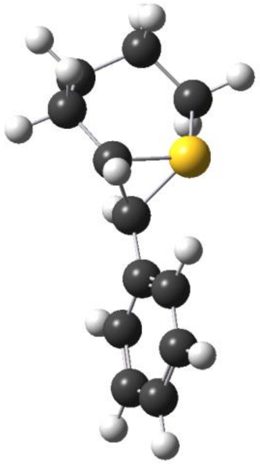

SCF energy: -1759.848854 Hartree

EE + Thermal Free Energy Correction: -864.073313 Hartree

Cation singlet total number of atoms: 28

C 3.08821300 -0.10912300 1.24104200

C 2.18548200 -1.31400500 1.00408300

C 0.95658600 -1.06652700 0.16738300

S 1.06825500 0.12518300 -1.24673300

C 2.48806800 1.19462100 -0.84453200

C 3.58471200 0.49033800 -0.06754500

C 0.07929500 0.09275500 0.34495700

C -1.39611200 0.05144500 0.20665100

C -2.13149200 1.07659800 0.79819000

C -3.51633600 1.08060300 0.71494600

C -4.17292600 0.06618400 0.03123600

C -3.44178500 -0.95377600 -0.56717600

C -2.05829600 -0.96583400 -0.47941600

H 3.94023300 -0.44491800 1.83154400

H 2.57489100 0.65477500 1.83129200

H 1.81661500 -1.71142200 1.95324200

H 2.75737800 -2.10791000 0.52099600

H 0.47764800 -1.96618600 -0.20519000

H 2.82747600 1.52818400 -1.82551200

H 2.08190300 2.04932800 -0.30393000

H 4.35162700 1.24198100 0.12614400

H 4.03840000 -0.28673600 -0.68756900

H 0.45532300 0.88341400 0.98651700

H -1.61358900 1.86687100 1.32833800

H -4.08068400 1.87664500 1.18338600

H -5.25340900 0.06861600 -0.03656500

H -3.95103500 -1.74508000 -1.10197200

H -1.50730800 -1.77016100 -0.95190700

10. X-ray diffraction analysis

CCDC 2409907 and 2409906 contain the supplementary crystallographic data for compounds 55 and 68. These data can be obtained free of charge via http://www.ccdc.cam.ac.uk/conts/retrieving.html, or from the Cambridge Crystallographic Data Centre,12 Union Road, Cambridge CB2 1EZ, UK; fax: (+44) 1223-336-033, or e-mail:deposit@ccdc.cam.ac.uk.

X-ray crystallographic data for compound 54

Empirical formula	C18 H20 O S
Formula weight	284.425
Temperature/K	200.0
Diffractometer/detector	Bruker D8 Venture / PhotonII area detector
Radiation	CuKα (λ = 1.54178)
Crystal system	Triclinic
Space group	P-1
a/Å	8.9711(12)
b/Å	9.2931(11)
c/Å	9.879(1)
α/°	94.588(4)
β/°	97.417(3)
γ/°	112.277(3)
Volume/Å^3	748.26(15)
Z	2
ρcalc /g·cm^{-3}	1.262
μ/mm^{-1}	0.210
F(000)	304
Θ range for data collection/°	2.69 – 26.40
	-11<h<11
Index ranges	-11<k<10
	-12<l<12
Reflections collected	18569
Unique reflections	2967
Parameters	182
Goodness-of-fit on F^2	1.047
Final R indexes [I>=2σ (I)]	R=0.040 wR2=0.097
CCDC Number	2409907

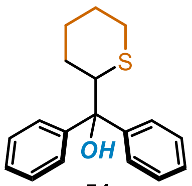

54

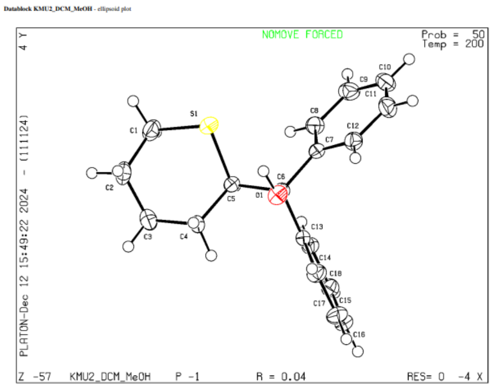

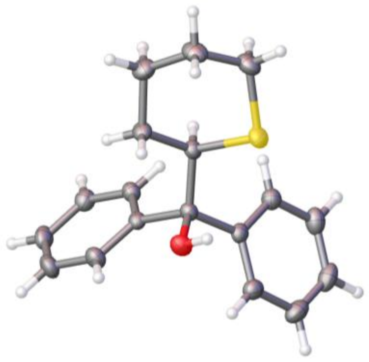

Figure S14. *ORTEP representation of **54**.*

X-ray crystallographic data for compound 68

Empirical formula	C32 H32 S2
Formula weight	480.743
Temperature/K	200.0
Diffractometer/detector	Bruker D8 Venture / PhotonII area detector
Radiation	CuKα (λ = 1.54178)
Crystal system	Monoclinic
Space group	P21/c
a/Å	8.0122(3)
b/Å	24.4744(8)
c/Å	13.3965(6)
α/°	90
β/°	107.291(2)
γ/°	90
Volume/Å^3	2701.8(3)
Z	4
ρcalc /g·cm^{-3}	1.273
μ/mm^{-1}	2.048
F(000)	1029
Θ range for data collection/°	3.61 - 70.04
	-8<h<9
Index ranges	-29<k<29
	-16<l<16
Reflections collected	37750
Unique reflections	4757
Parameters	308
Goodness-of-fit on F^2	1.035
Final R indexes [I>=2σ (I)]	R=0.058, wR2=0.145
CCDC Number	2409906

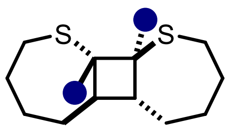

68

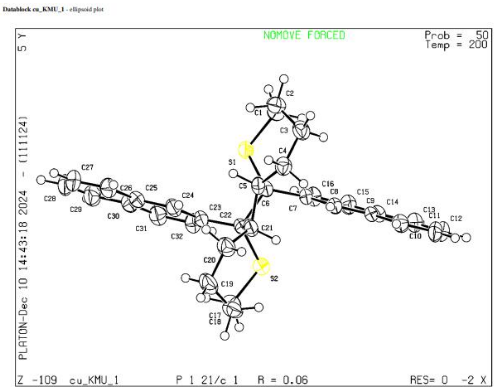

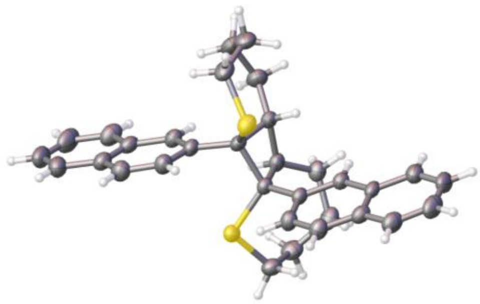

Figure S15. *ORTEP representation of* ***68****.*

11. References

1. (a) N. R. Babij, E. O. McCusker, G. T. Whiteker, B. Canturk, N. Choy, L. C. Creemer, C. V. De Amicis, N. M. Hewlett, P. L. Johnson, J. A. Knobelsdorf, F. Li, B. A. Lorsbach, B. M. Nugent, S. J. Ryan, M. R. Smith, Q. Yang, "NMR Chemical Shifts of Trace Impurities: Industrially Preferred Solvents Used in Process and Green Chemistry" *Org. Process Res. Dev.* **2016**, *20,* 661–667; (b) H. E. Gottlieb, V. Kotlyar, A. Nudelman, "NMR Chemical Shifts of Common Laboratory Solvents as Trace Impurities" *J. Org. Chem.* **1997**, *62,* 7512–7515.

2. (a) Bruker. SAINT and SADABS. Bruker AXS. APEX3, SAINT SADABS. Bruker AXS Inc., Madison, Wisconsin, USA. **2016**.

(b) G. M. Sheldrick, "Crystal Structure Refinement with SHELXL" *Acta Crystallogr. Sect. C Struct. Chem.* **2015**, *71*, 3–8.

(c) G. M. Sheldrick. "A Short History of SHELX" *Acta Crystallogr. Sect. A Found. Crystallogr.* **2008**, *64*, 112–122.

(d) O. V. Dolomanov, L. J. Bourhis, R. J. Gildea, J. A. K. Howard, H. Puschmann, "OLEX2: A Complete Structure Solution, Refinement and Analysis Program" *J. Appl. Crystallogr.* **2009**, *42*, 339–341.

(e) M. N. Burnett, C. K. Johnson, "ORTEP-III: Oak Ridge Thermal Ellipsoid Plot Program for Crystal Structure Illustrations" Citeseer, **1996**.

(f) C. F. Macrae, I. Sovago, S. J. Cottrell, P. T. A. Galek, P. McCabe, E. Pidcock, M. Platings, G. P. Shields, J. S. Stevens, M. Towler, "Mercury 4.0: From Visualization to Analysis, Design and Prediction" *J. Appl. Crystallogr.* **2020**, *53*, 226–235.

3. S. D. A. Zondag, D. Mazzarella, T. Noël, "Scale-Up of Photochemical Reactions: Transitioning from Lab Scale to Industrial Production" *Annu. Rev. Chem. Biomol. Eng.* **2023**, *14*, 283–300.

4. (a) Synthesis of **4CzIPN**: H. Huang, X. Li, C. Yu, Y. Zhang, P. S. Mariano, W. Wang, "Visible-Light-Promoted Nickel- and Organic-Dye-Cocatalyzed Formylation Reaction of Aryl Halides and Triflates and Vinyl Bromides with Diethoxyacetic Acid as a Formyl Equivalent" *Angew. Chem. Int. Ed.* **2017**, *56,* 1500–1505.

(b) Synthesis of **4DPAIPN** (modified protocol using dimethylacetamide under reflux), **3DPAFIPN** and **3DPA2FBN**: E. Speckmeier, T. G. Fischer, K. Zeitler, "A Toolbox Approach To Construct Broadly Applicable Metal-Free Catalysts for Photoredox Chemistry: Deliberate Tuning of Redox Potentials and Importance of Halogens in Donor–Acceptor Cyanoarenes" *J. Am. Chem. Soc.* **2018**, *140,* 15353–15365.

(c) Synthesis of **APC-1**: T. Bortolato, G. Simionato, M. Vayer, C. Rosso, L. Paoloni, E. M. Benetti, A. Sartorel, D. Lebœuf, L. Dell'Amico, "The Rational Design of Reducing Organophotoredox Catalysts

Unlocks Proton-Coupled Electron-Transfer and Atom Transfer Radical Polymerization Mechanisms” *J. Am. Chem. Soc.* **2023**, *145,* 1835–1846.

(d) Synthesis of **NC-1**: J. Mateos, F. Rigodanza, A. Vega-Peñaloza, A. Sartorel, M. Natali, T. Bortolato, G. Pelosi, X. Companyó, M. Bonchio, L. Dell'Amico, “Naphthochromenones: Organic Bimodal Photocatalysts Engaging in Both Oxidative and Reductive Quenching Processes” *Angew. Chem. Int. Ed.* **2019**, *59,* 1302–1312.

5. W. Zawodny, C. J. Teskey, M. Mishevska, M. Völkl, B. Maryasin, L. González, N. Maulide, “α-Functionalisation of Ketones Through Metal-Free Electrophilic Activation” *Angew. Chem. Int. Ed.* **2020**, *59,* 20935–20939.

6. M. Yar, E. M. McGarrigle, V. K. Aggarwal, “An Annulation Reaction for the Synthesis of Morpholines, Thiomorpholines, and Piperazines from β-Heteroatom Amino Compounds and Vinyl Sulfonium Salts” *Angew. Chem. Int. Ed.* **2008**, *47,* 3784–3786.

7. S. Ozaki, E. Matsui, H. Yoshinaga, S. Kitagawa, “Synthesis of cyclic sulfides by intramolecular ring opening of epoxides by thiolates generated by nickel complex catalyzed electroreduction of thioacetates” *Tetrahedron Lett.* **2000**, *41*, 2621–2624.

8. C. Urban, Y. Mace, F. Cadoret, J. C. Blazejewski, E. Magnier, “Divergent Preparation of Fluoroalkylated Sulfilimine and Sulfilimino Iminium Salts” *Adv. Synth. Catal.* **2010**, *352*, 2805–2814.

9. G. J. Quallich, J. W. Lackey, “Diastereoselective oxidation of sulfides to sulfoxides with potassium peroxymonosulfate” *Tetraedron Lett.* **1990**, *31*, 3685–3686.

10. H. Cao, S. Ma, Y. Feng, Y. Guo, P. Jiao, “Synthesis of β-nitro ketones from geminal bromonitroalkanes and silyl enol ethers by visible light photoredox catalysis” *Chem. Commun.* **2022**, *58,* 1780–1783.

11. B. Limburg, “An Extension of the Stern–Volmer Equation for Thermally Activated Delayed Fluorescence (TADF) Photocatalysts” *J. Phys. Chem. Lett.* **2024**, *15*, 10495–10499.

12. C. M. Rayner, “Chapter 3 Synthetic transformations involving thiiranium ion intermediates” *Organosulfur Chemistry* **1995**, *1*, 89-131.

12. NMR spectra

1-(1-phenylvinyl)tetrahydro-1*H*-thiophen-1-ium hexafluorophosphate (V) 1

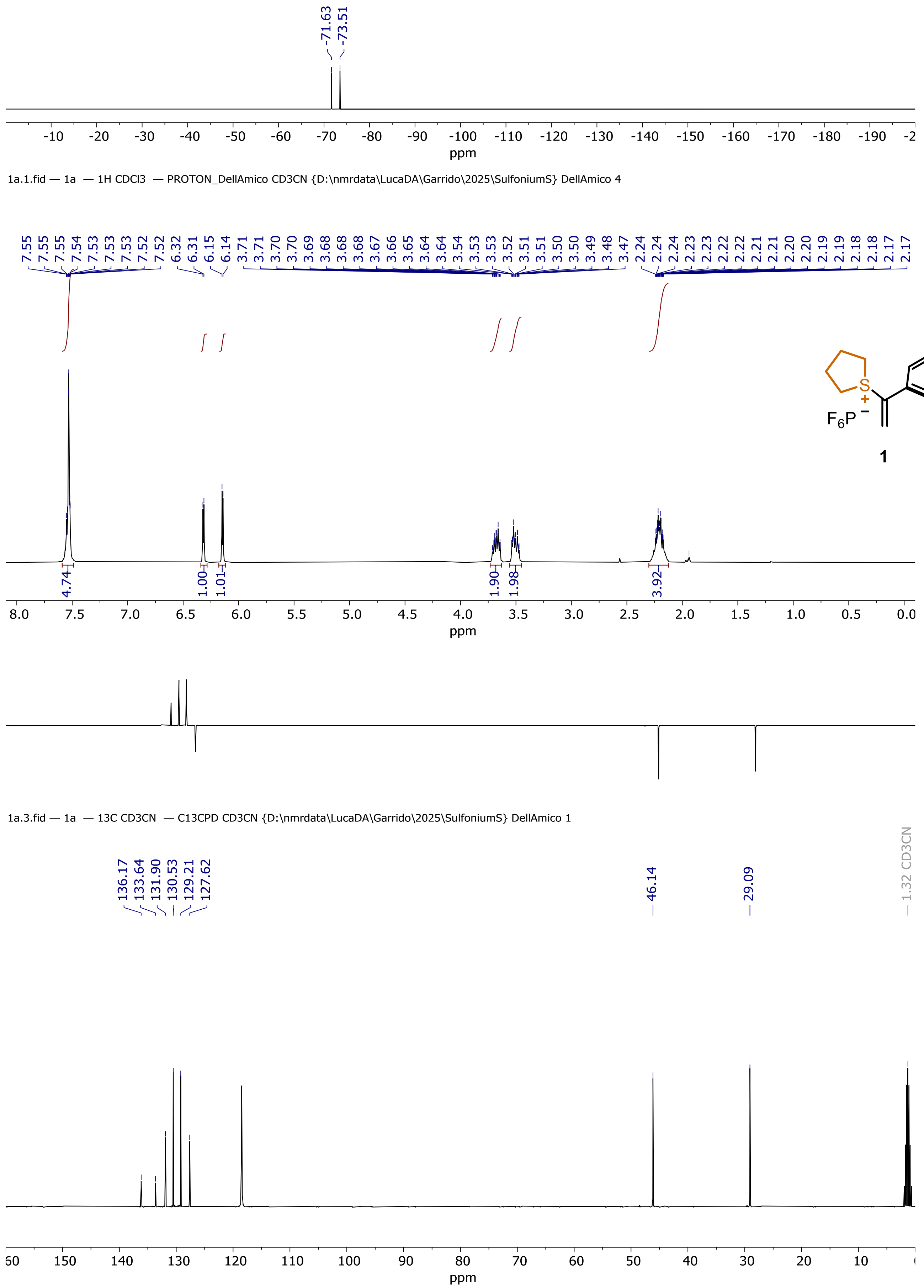

Figure S16. *^{19}F, ^{1}H and ^{13}C NMR spectra of compound **1** in CD_3CN.*

7-phenyl-2,3,4,5-tetrahydrothiepine 2

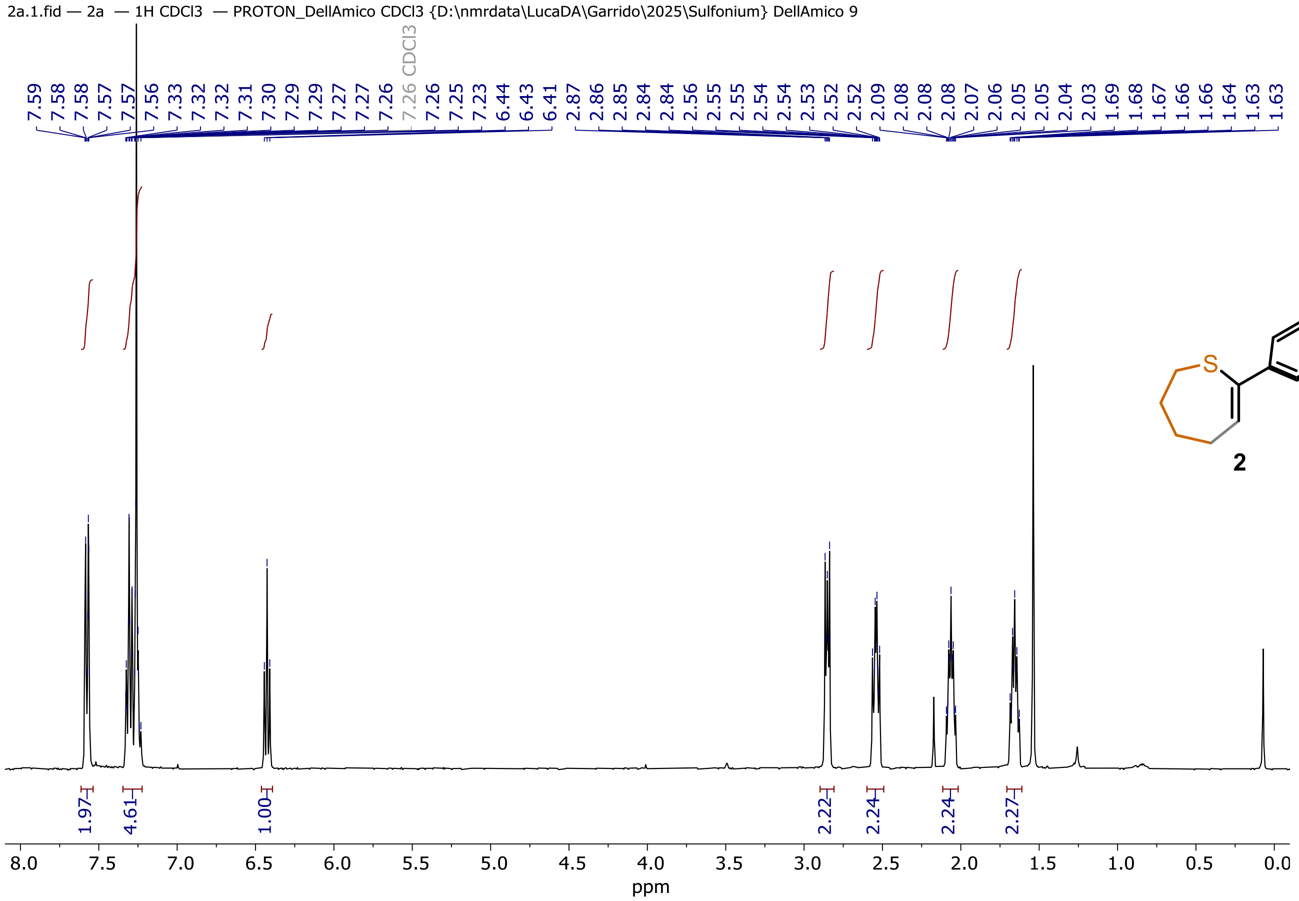

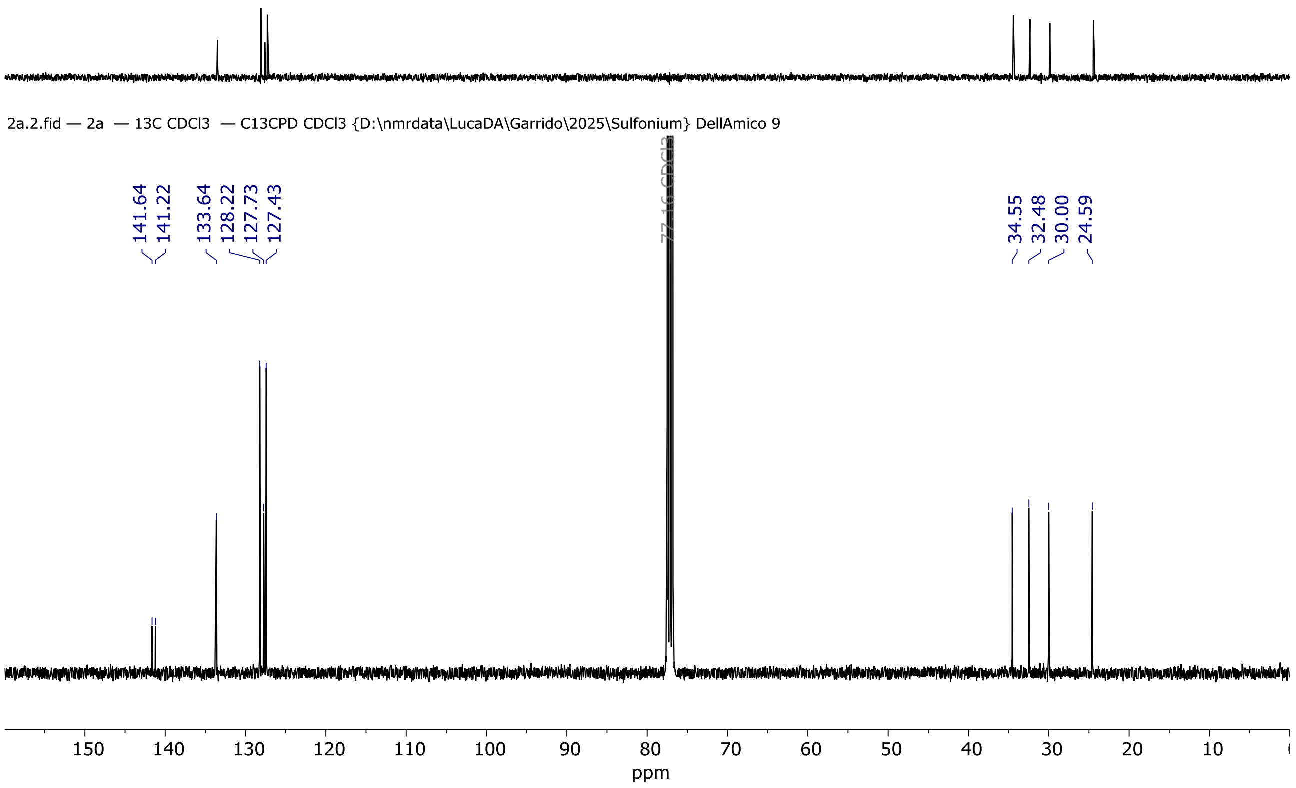

Figure S17. *^{1}H and ^{13}C NMR spectra of compound **2** in $CDCl_3$.*

1-(1-(4-fluorophenyl)vinyl)tetrahydro-1*H*-thiophen-1-ium hexafluorophosphate (V) 3

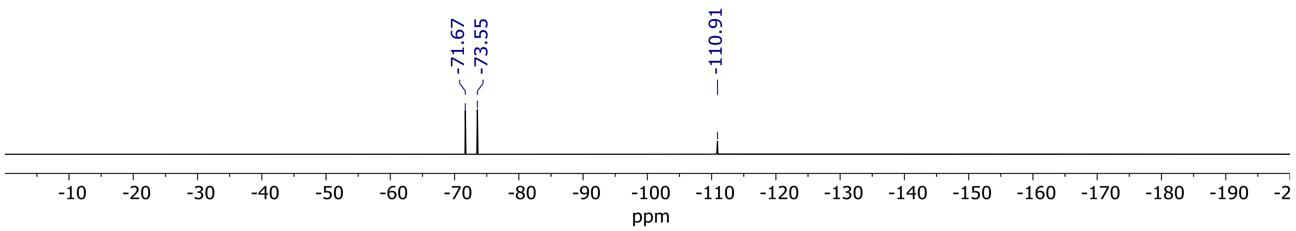

1b.1.fid — 1b — 1H CDCl3 — PROTON_DellAmico CD3CN {D:\nmrdata\LucaDA\Garrido\2025\SulfoniumS} DellAmico 5

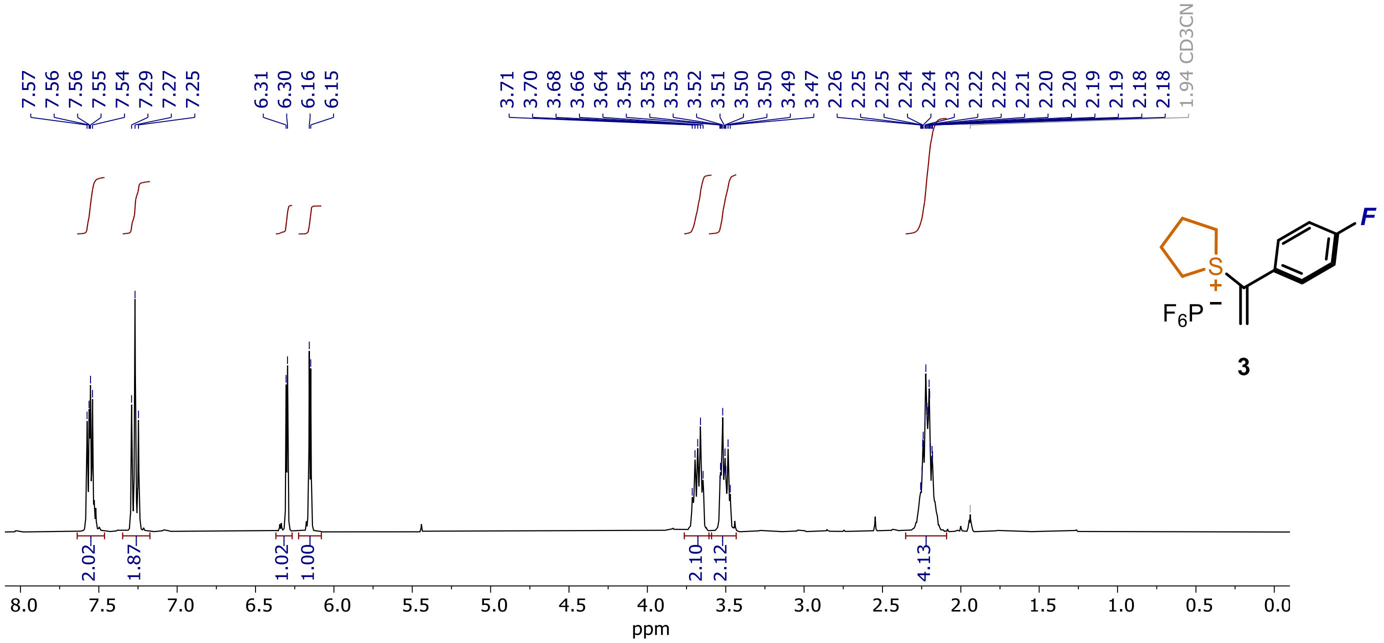

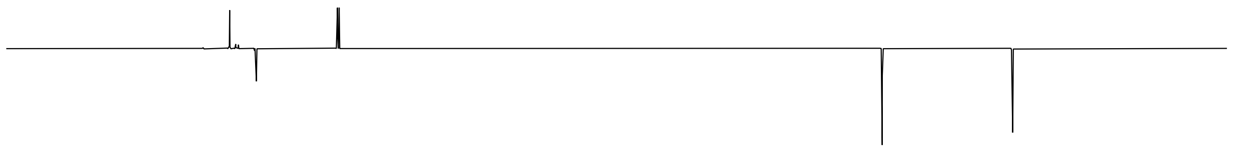

1b.3.fid — 1b — 13C CD3CN — C13CPD CD3CN {D:\nmrdata\LucaDA\Garrido\2025\SulfoniumS} DellAmico 2

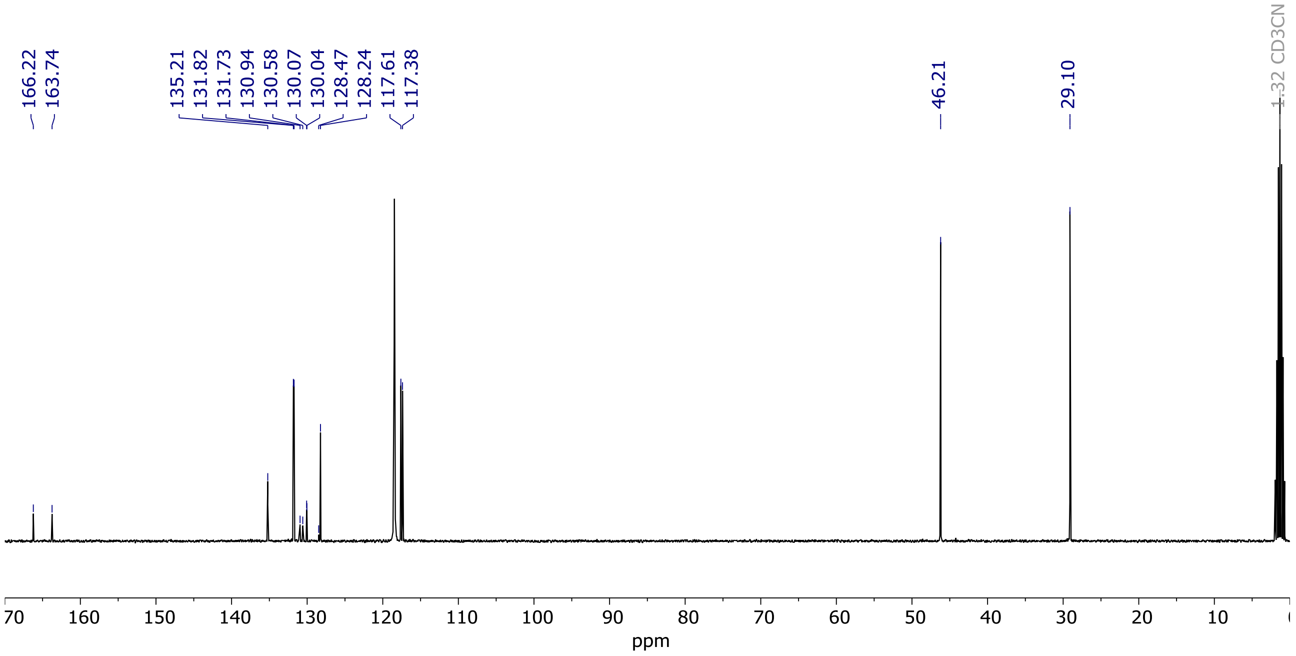

Figure S18. *^{19}F, ^{1}H and ^{13}C NMR spectra of compound **3** in CD_3CN.*

1-(1-(4-chlorophenyl)vinyl)tetrahydro-1*H*-thiophen-1-ium hexafluorophosphate (V) 4

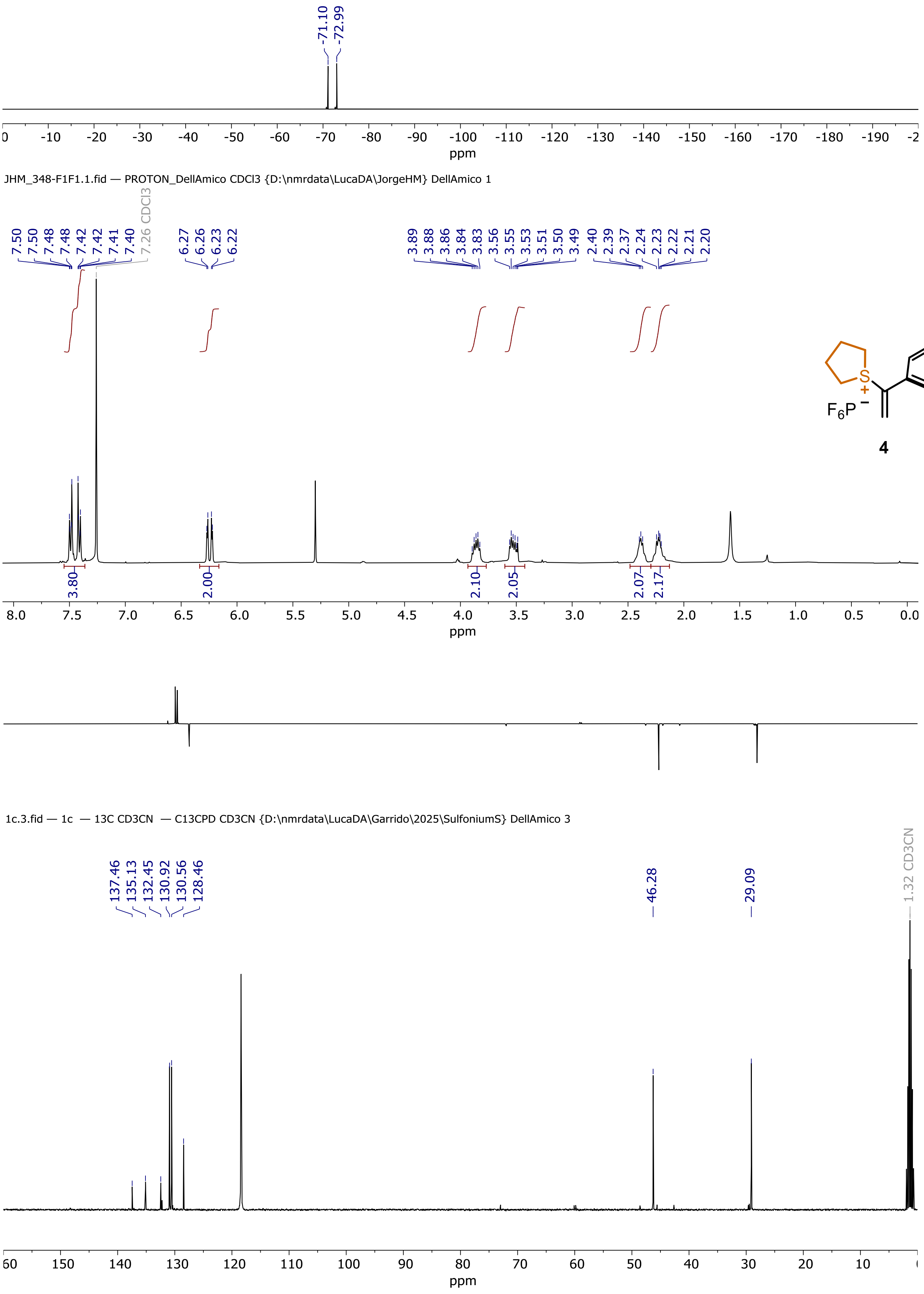

Figure S19. *^{19}F, ^{1}H ($CDCl_3$) and ^{13}C NMR (CD_3CN) spectra of compound **4**.*

1-(1-(4-bromophenyl)vinyl)tetrahydro-1*H*-thiophen-1-ium hexafluorophosphate (V) 5

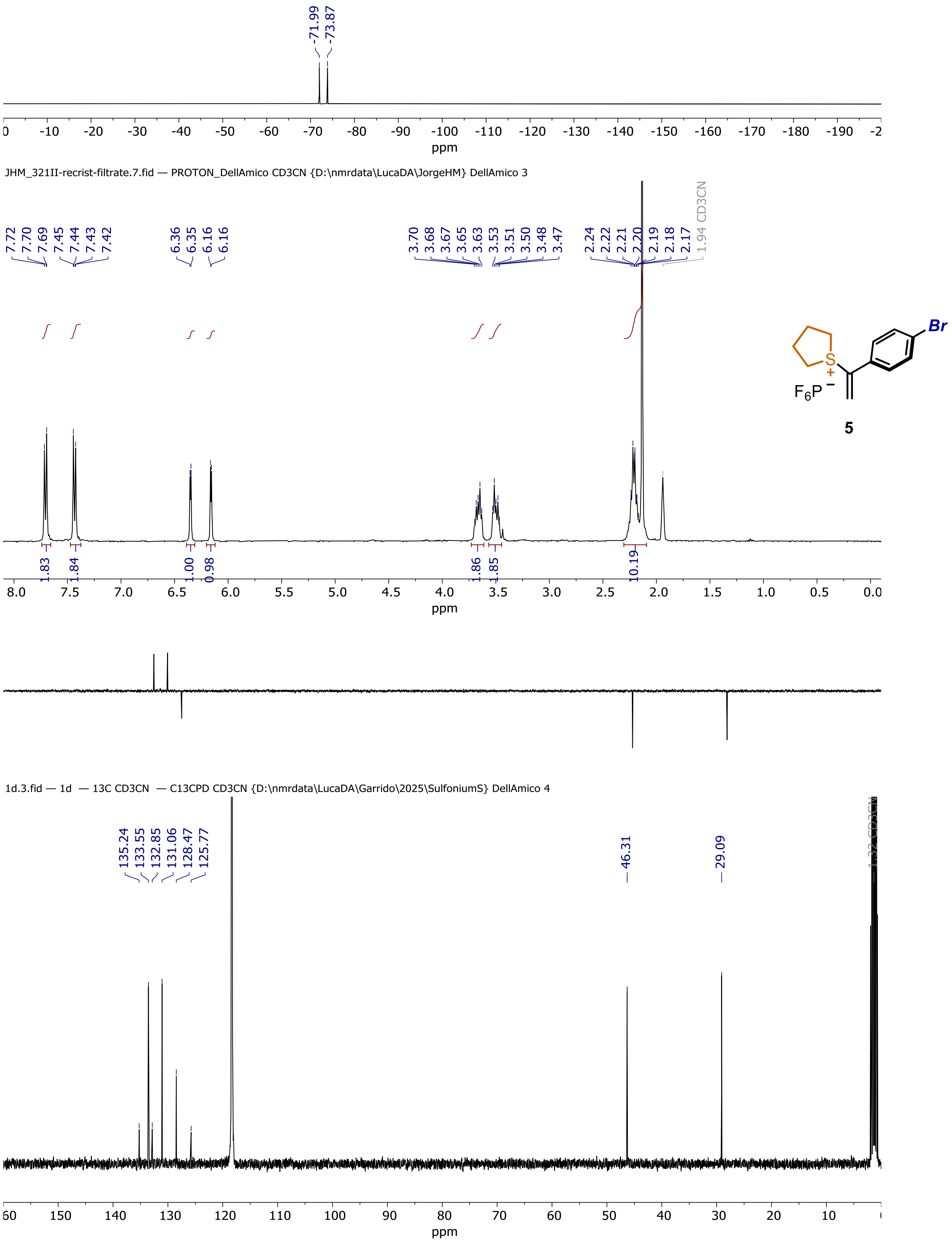

Figure S20. *^{19}F, ^{1}H and ^{13}C NMR spectra of compound **5** in CD_3CN.*

1-(1-(4-methoxyphenyl)vinyl)tetrahydro-1*H*-thiophen-1-ium hexafluorophosphate (V) 6

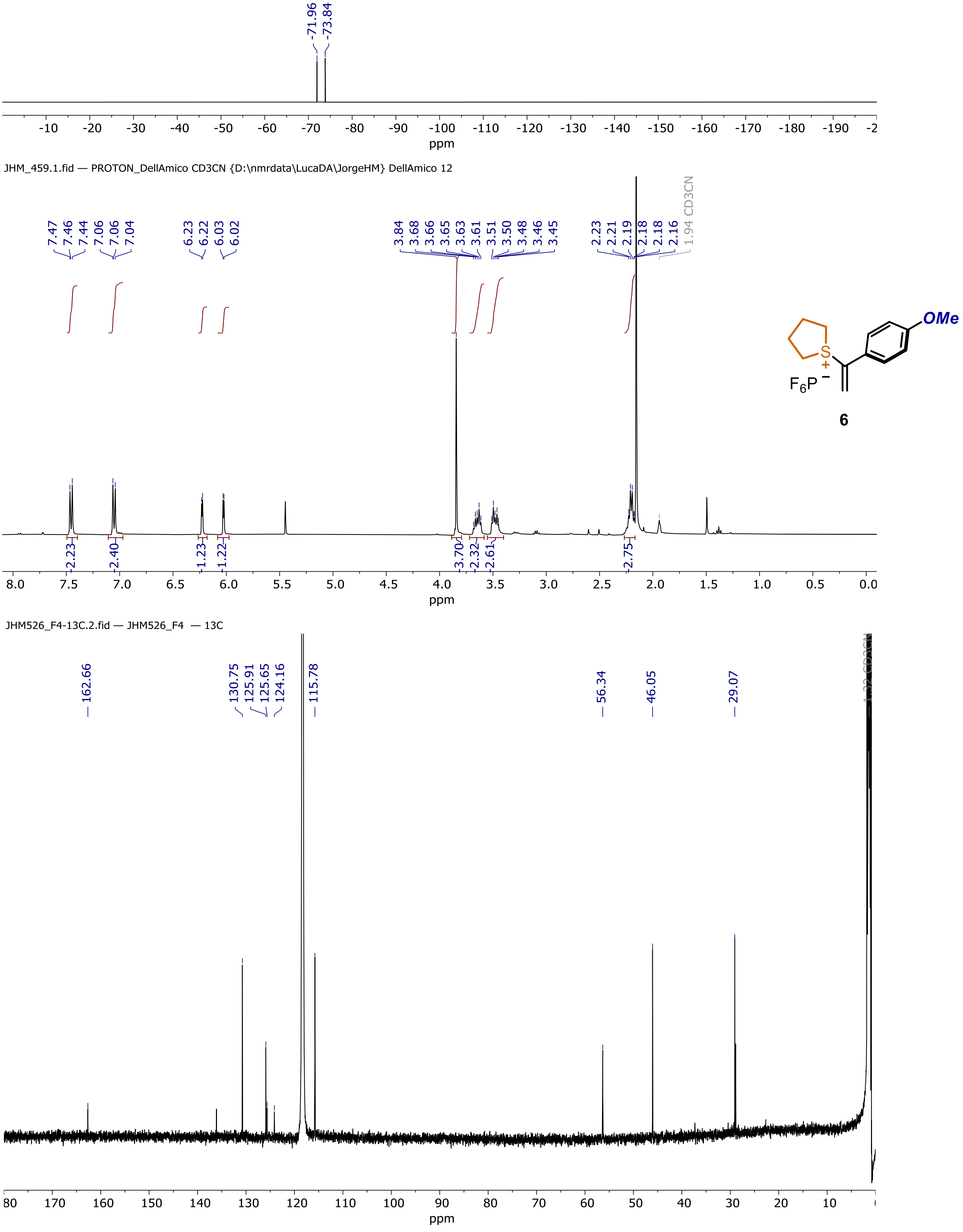

Figure S21. *^{19}F, ^{1}H and ^{13}C NMR spectra of compound **6** in CD_3CN.*

1-(1-(*p*-tolyl)vinyl)tetrahydro-1*H*-thiophen-1-ium hexafluorophosphate (V) 7

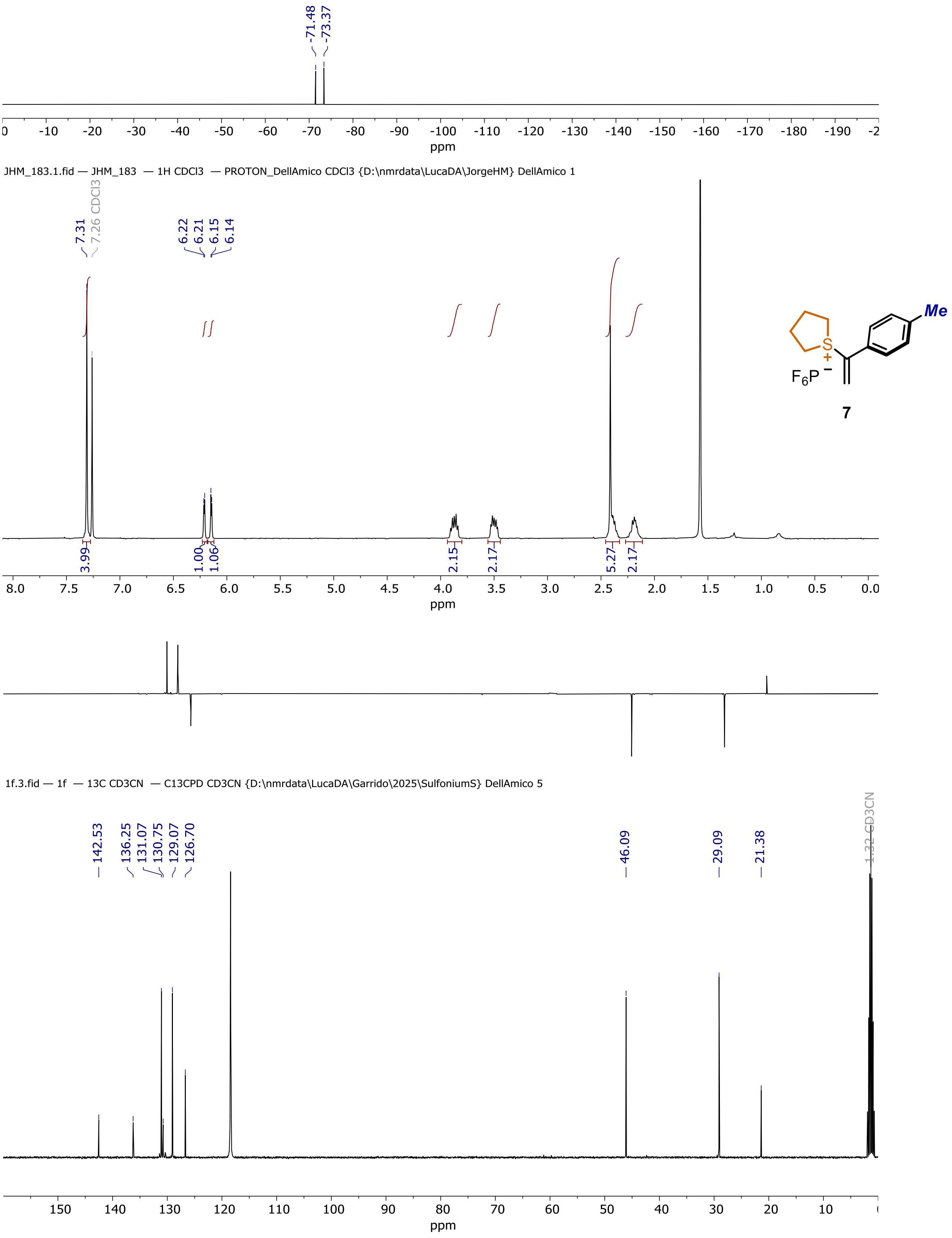

Figure S22. *^{19}F, ^{1}H ($CDCl_3$) and ^{13}C NMR (CD_3CN) spectra of compound **7**.*

1-(1-(*m*-tolyl)vinyl)tetrahydro-1*H*-thiophen-1-ium hexafluorophosphate (V) 8

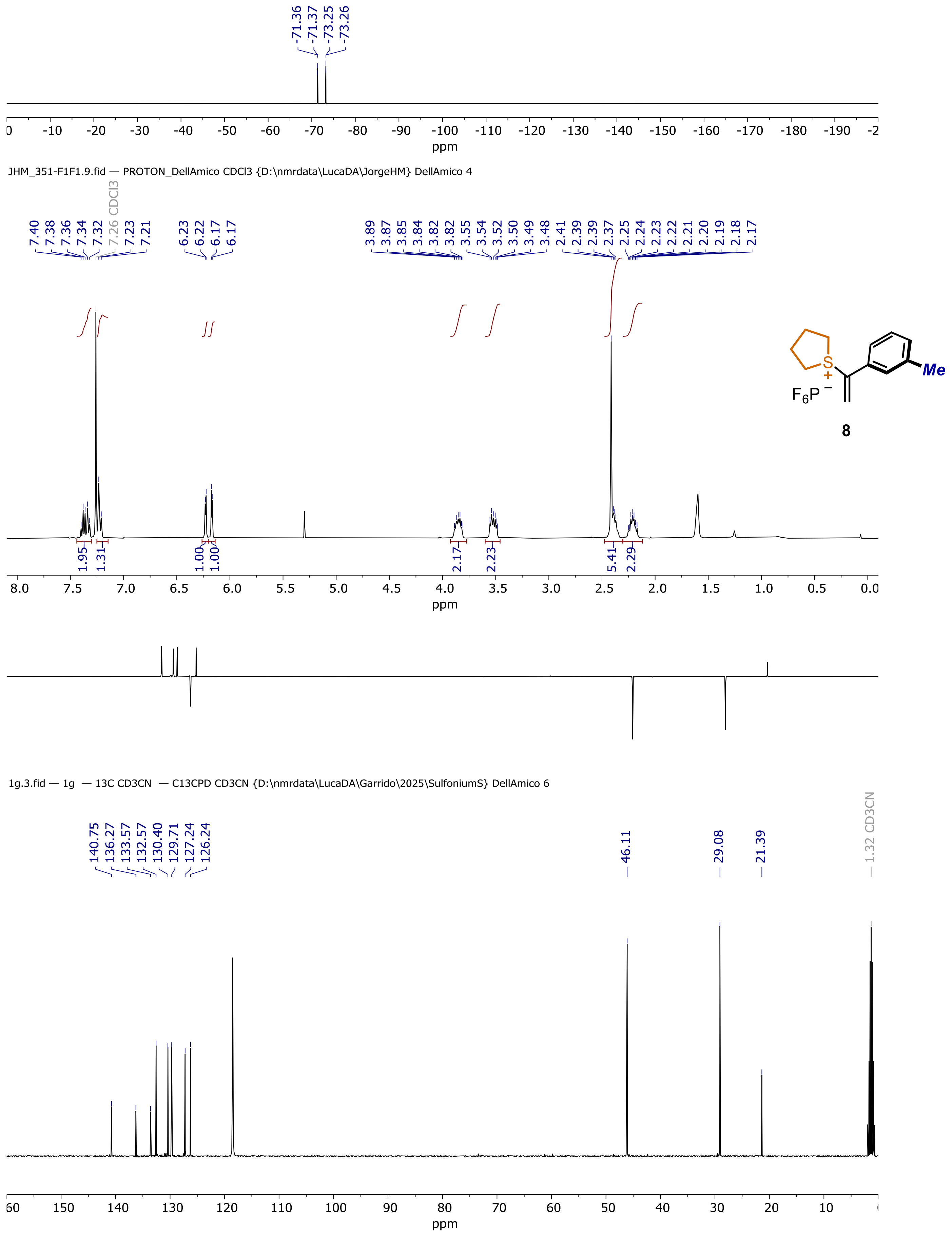

Figure S23. *^{19}F, ^{1}H ($CDCl_3$) and ^{13}C NMR (CD_3CN) spectra of compound **8**.*

1-(1-(*m*-tolyl)vinyl)tetrahydro-1*H*-thiophen-1-ium hexafluorophosphate (V) 8

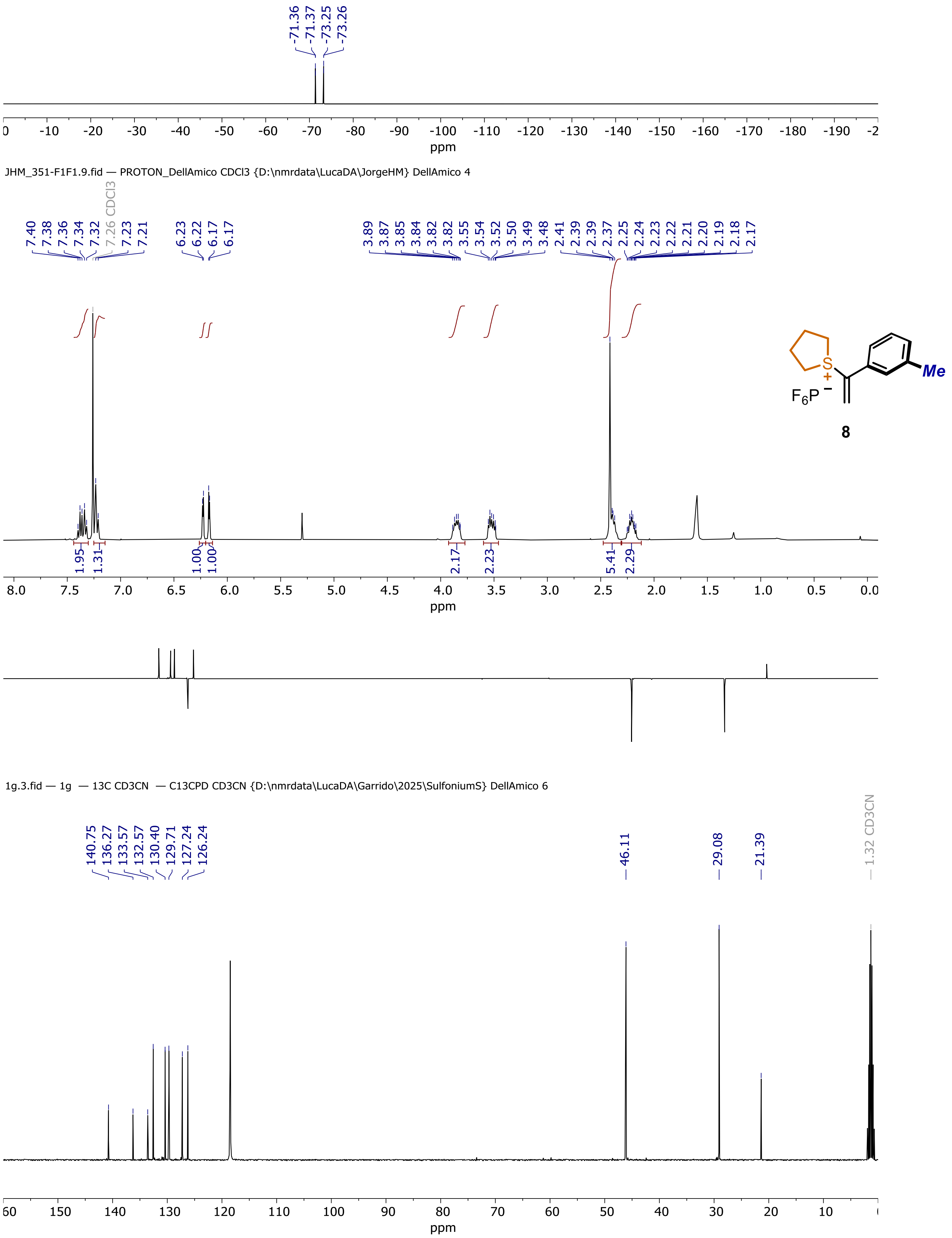

Figure S24. *^{19}F, ^{1}H ($CDCl_3$) and ^{13}C NMR (CD_3CN) spectra of compound **8**.*

(*E*)-1-(1,2-diphenylvinyl)tetrahydro-1*H*-thiophen-1-ium hexafluorophosphate (V) 10

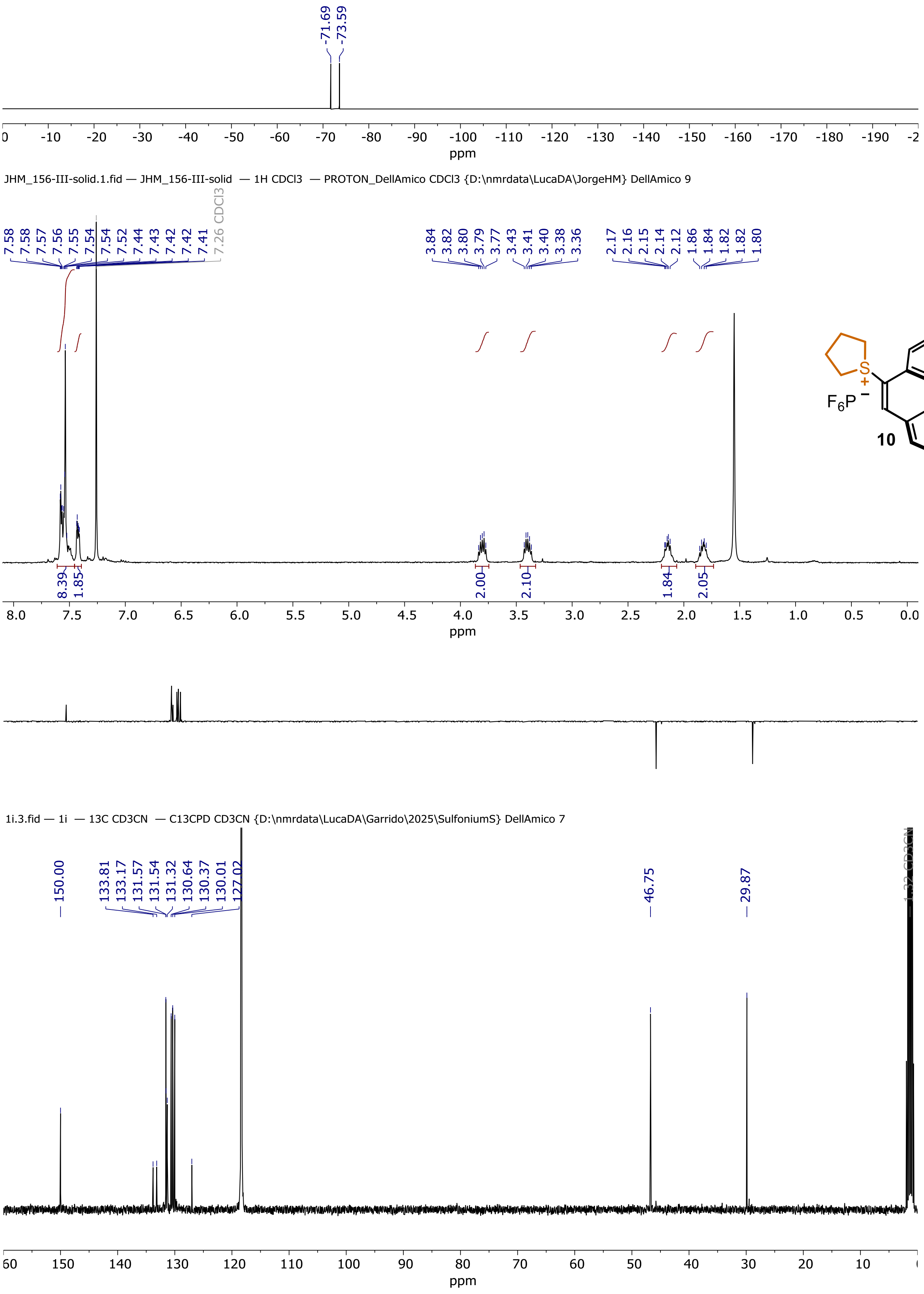

Figure S25. *^{19}F, ^{1}H ($CDCl_3$) and ^{13}C NMR (CD_3CN) spectra of compound* ***10****.*

1-(3,4-dihydronaphthalen-1-yl)tetrahydro-1*H*-thiophen-1-ium hexafluorophosphate (V) 11

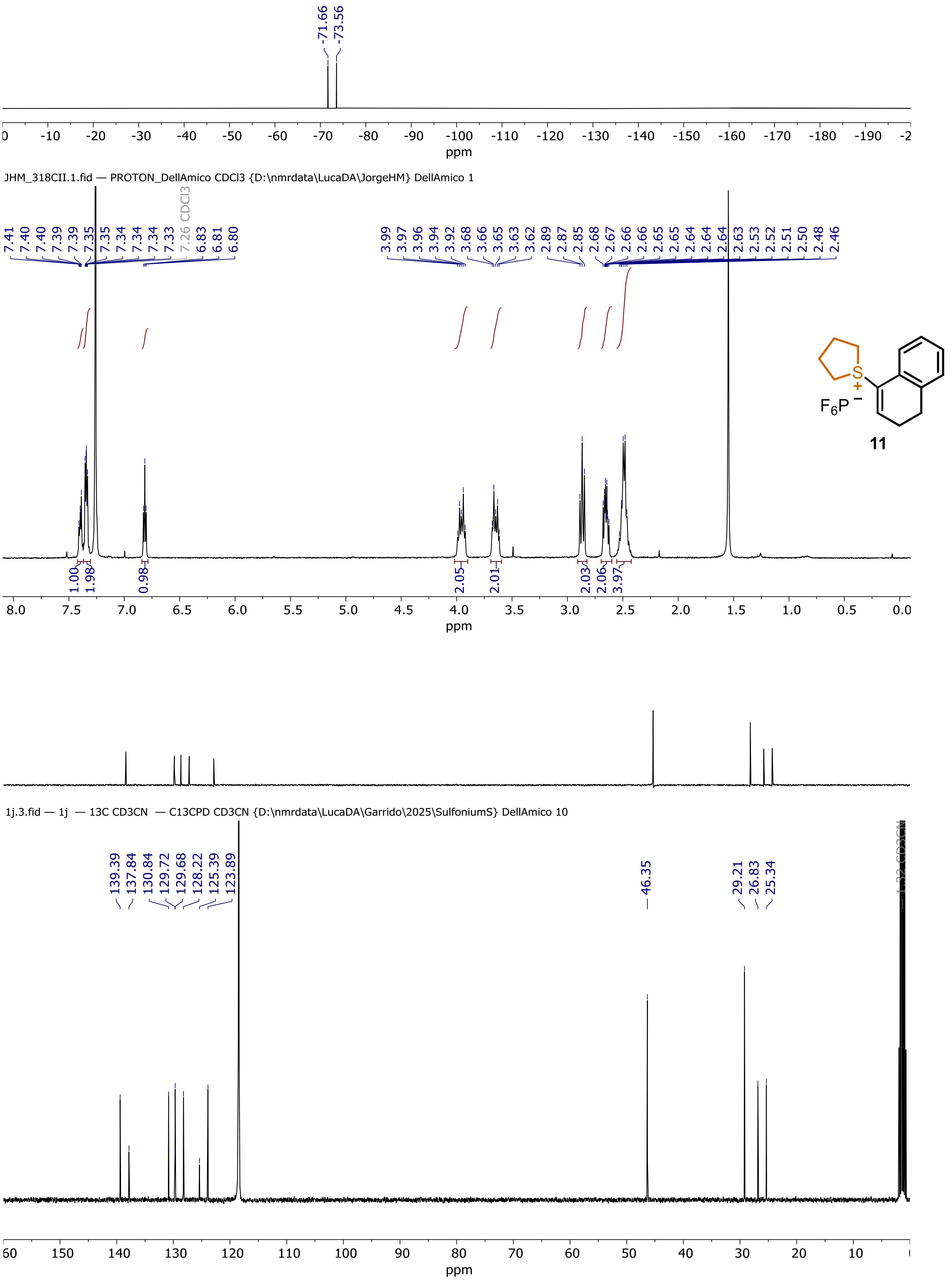

Figure S26. *^{19}F, ^{1}H ($CDCl_3$) and ^{13}C NMR (CD_3CN) spectra of compound **11**.*

1-(1-phenylvinyl)hexahydrothiopyrylium hexafluorophosphate (V) 12

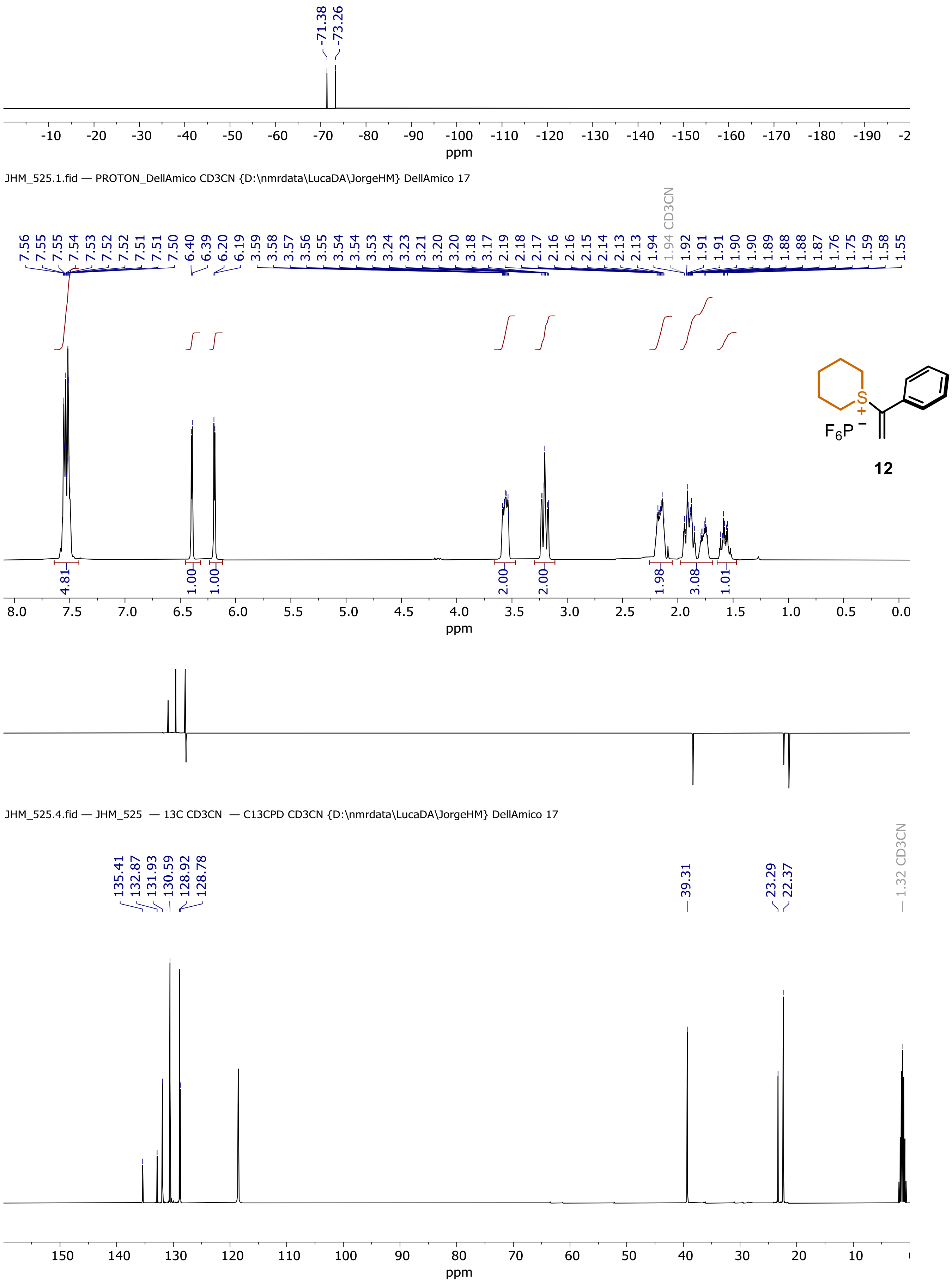

Figure S27. *^{19}F, ^{1}H and ^{13}C NMR spectra of compound **12** in CD_3CN.*

1-(1-(naphthalen-2-yl)vinyl)tetrahydro-1*H*-thiophen-1-ium hexafluorophosphate (V) 13

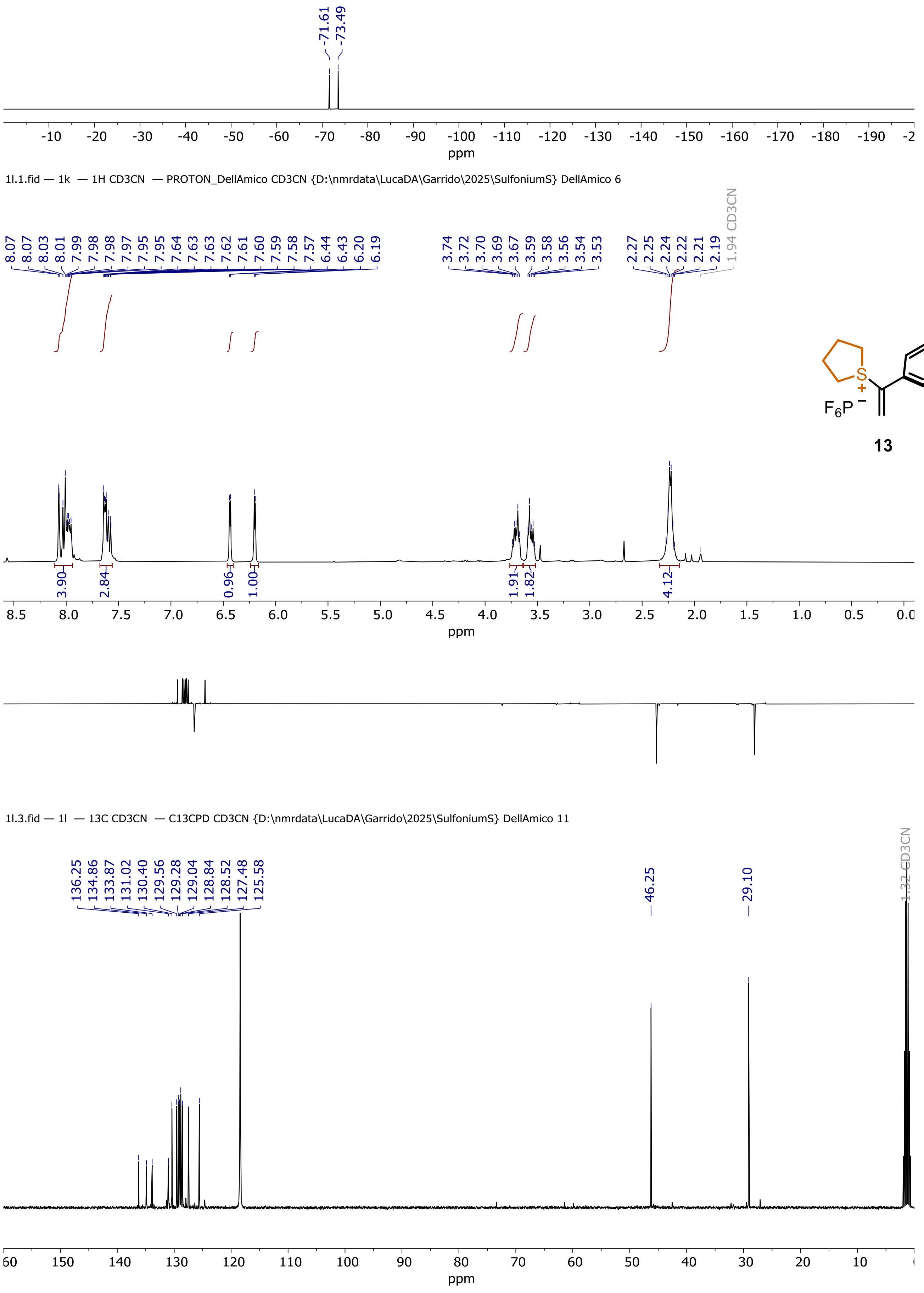

Figure S28. *^{19}F, ^{1}H and ^{13}C NMR spectra of compound* ***13*** *in* CD_3CN*.*

1-vinyltetrahydro-1H-thiophen-1-ium hexafluorophosphate (V) 14

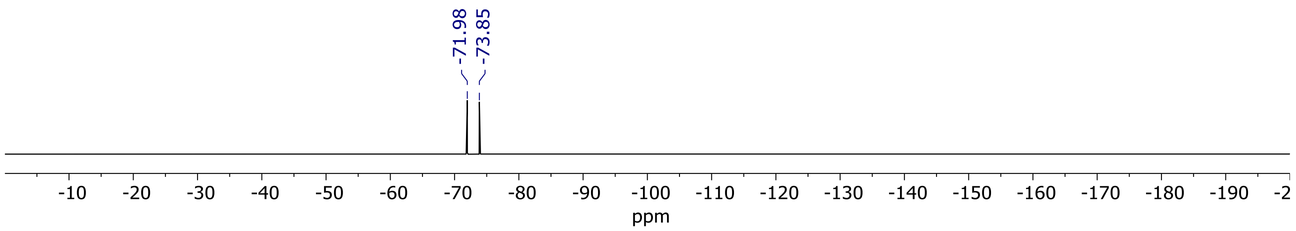

JG240_PF6-vinylsulfonium.1.fid — JG240_PF6 — 1H CD3CN — PROTON_DellAmico CD3CN {D:\nmrdata\LucaDA\Garrido\2024} DellAmico 18

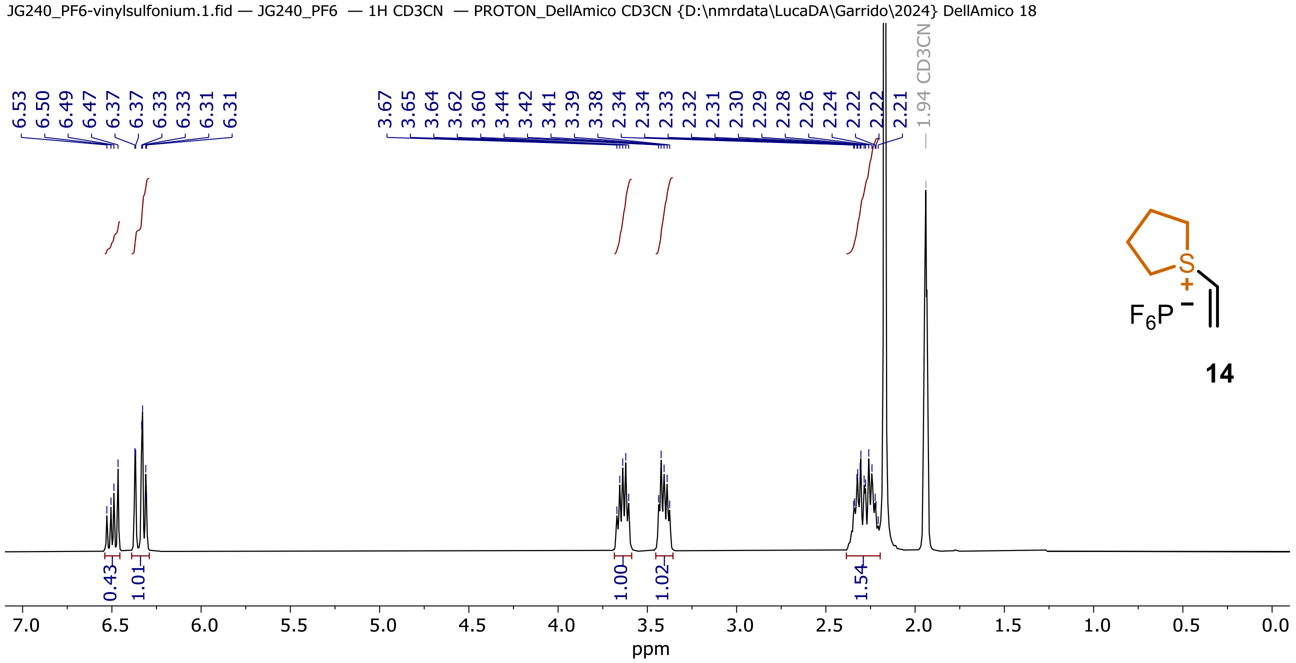

14

Figure S29. *^{19}F and ^{1}H spectra of compound **14** in CD_3CN.*

7-(4-fluorophenyl)-2,3,4,5-tetrahydrothiepine 15

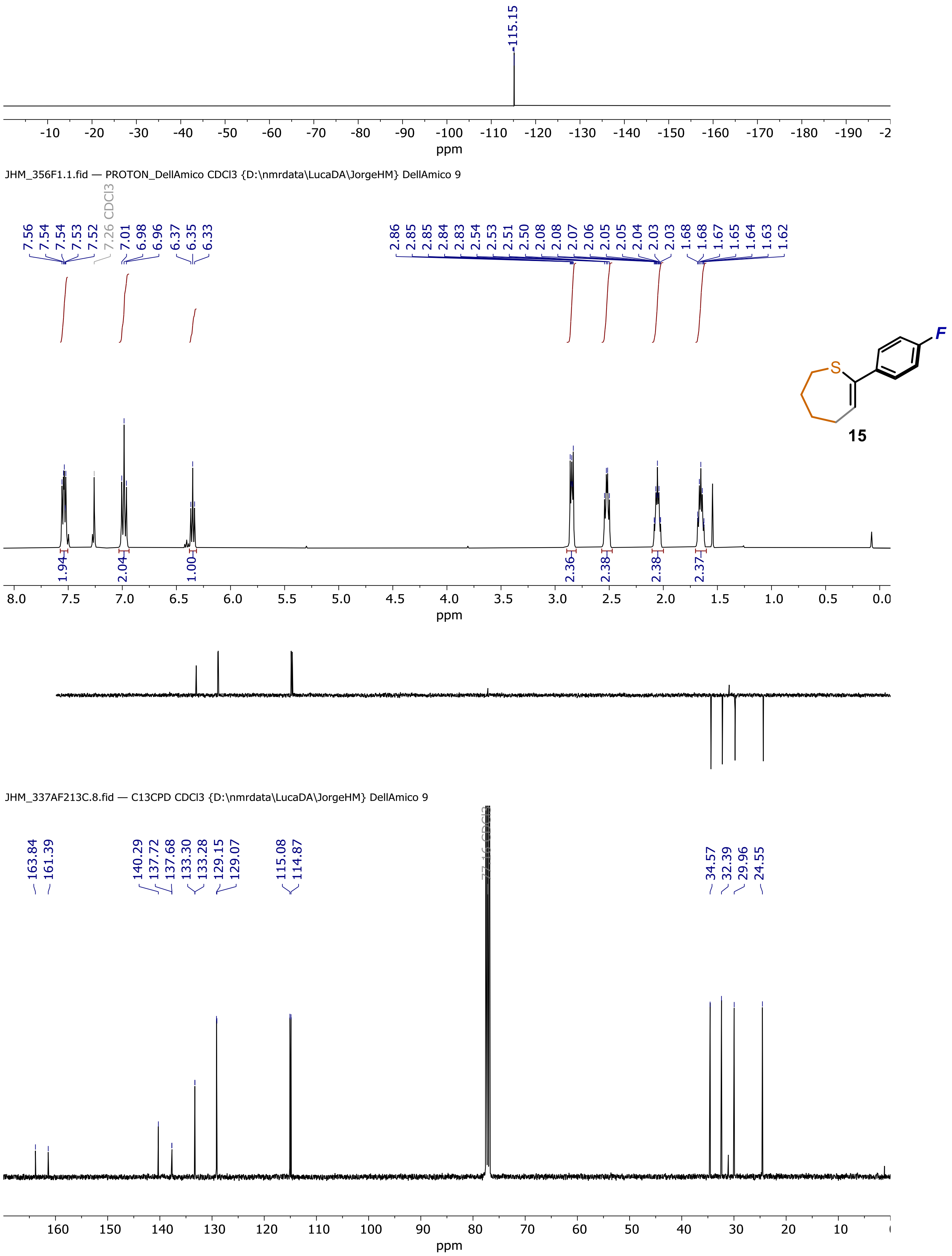

Figure S30. *^{19}F, ^{1}H and ^{13}C NMR spectra of compound **15** in $CDCl_3$.*

7-(4-chlorophenyl)-2,3,4,5-tetrahydrothiepine 16

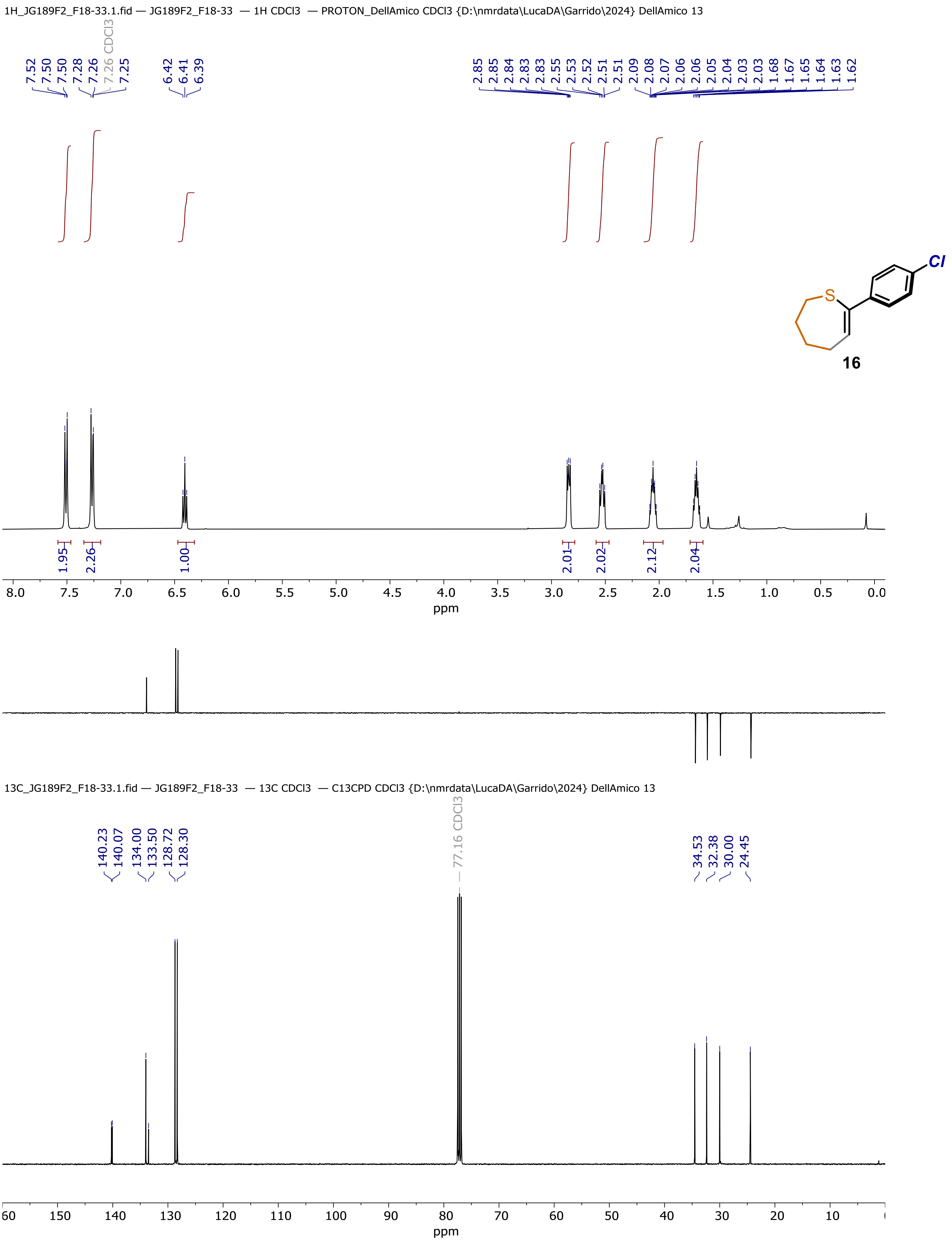

Figure S31. *^{1}H and ^{13}C NMR spectra of compound **16** in $CDCl_3$.*

7-(4-bromophenyl)-2,3,4,5-tetrahydrothiepine 17

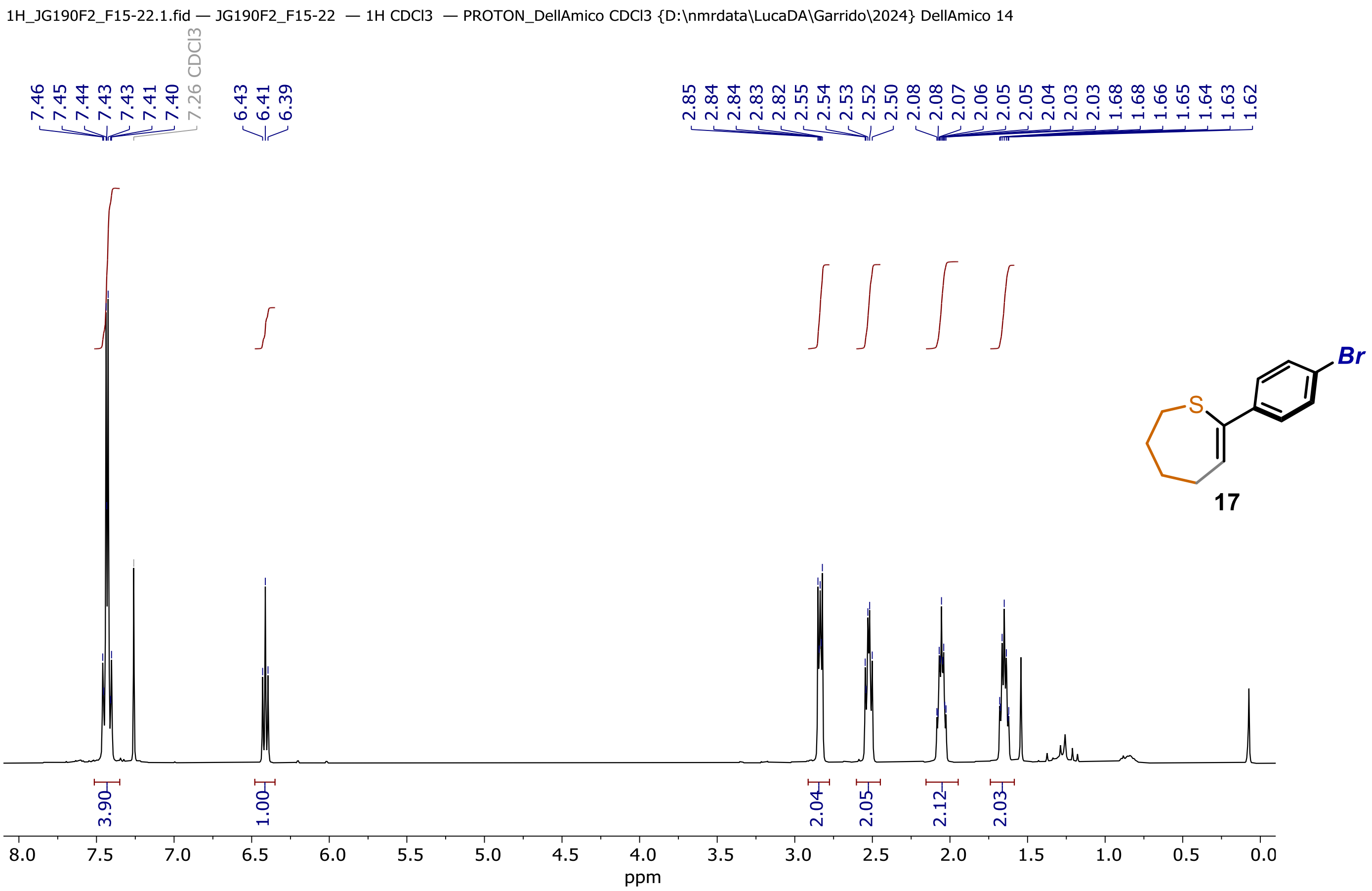

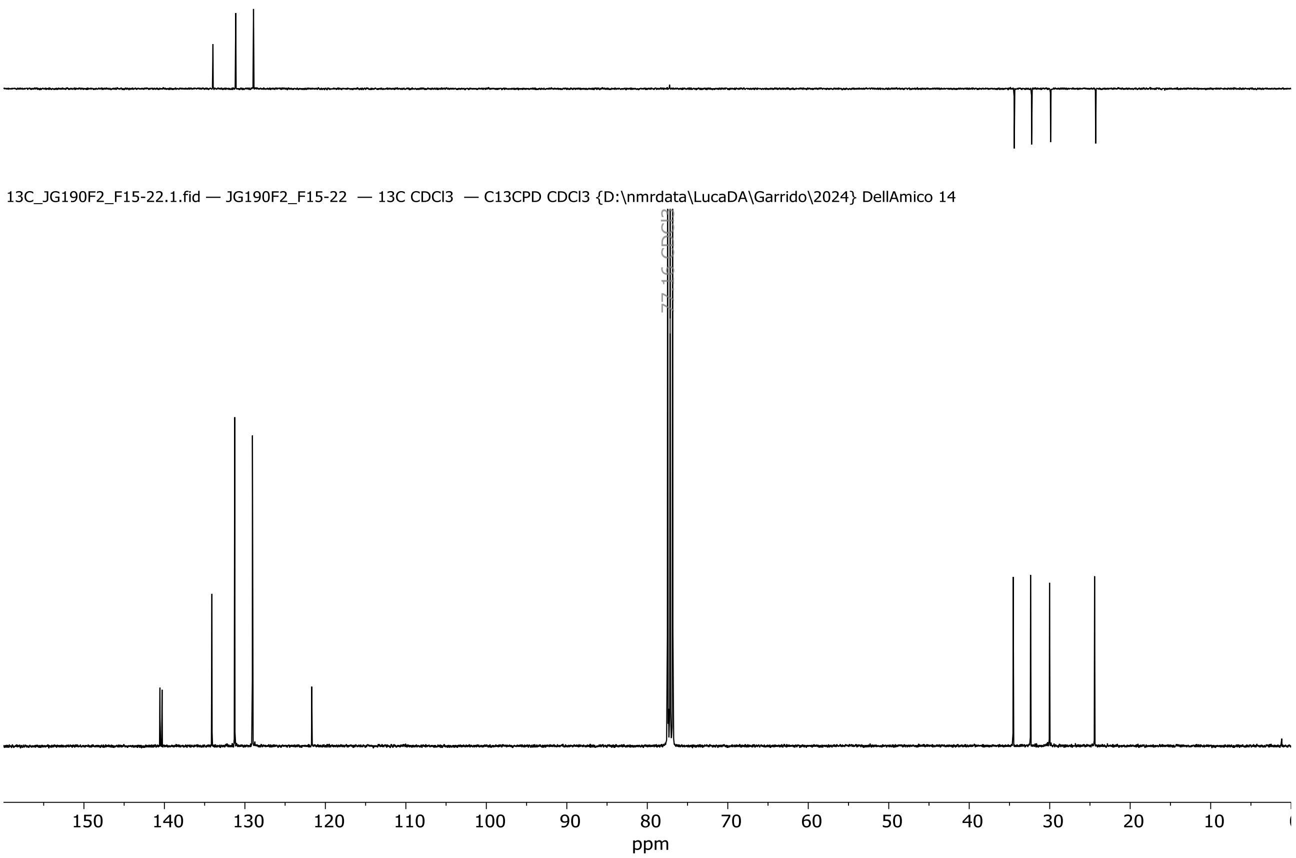

Figure S32. *^{1}H and ^{13}C NMR spectra of compound **17** in $CDCl_3$.*

7-(4-methoxyphenyl)-2,3,4,5-tetrahydrothiepine 18

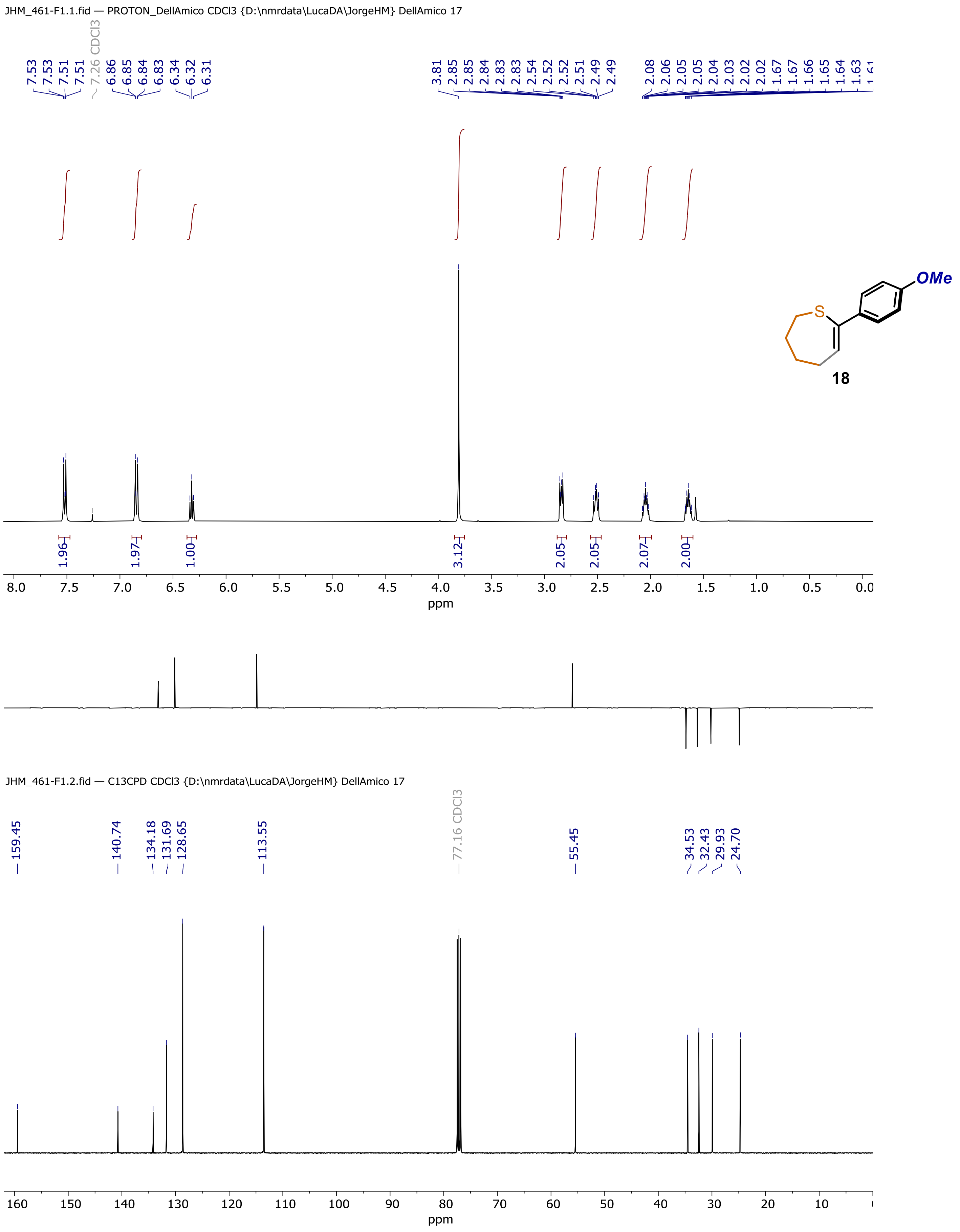

Figure S33. *^{1}H and ^{13}C NMR spectra of compound **18** in CDCl$_3$.*

7-(*p*-tolyl)-2,3,4,5-tetrahydrothiepine 19

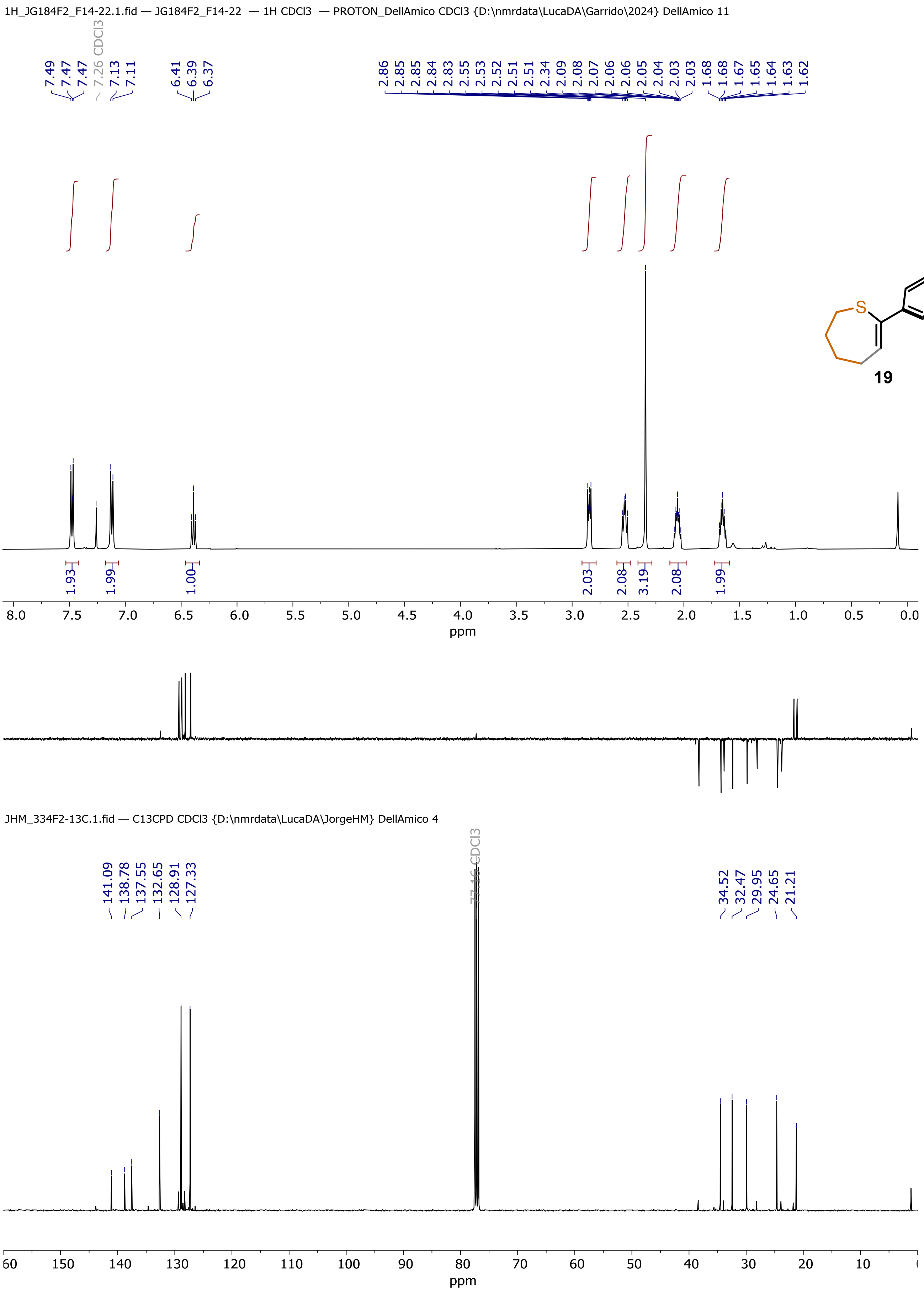

Figure S34. *^{1}H and ^{13}C NMR spectra of compound* ***19*** *in* $CDCl_3$.

7-(*m*-tolyl)-2,3,4,5-tetrahydrothiepine 20

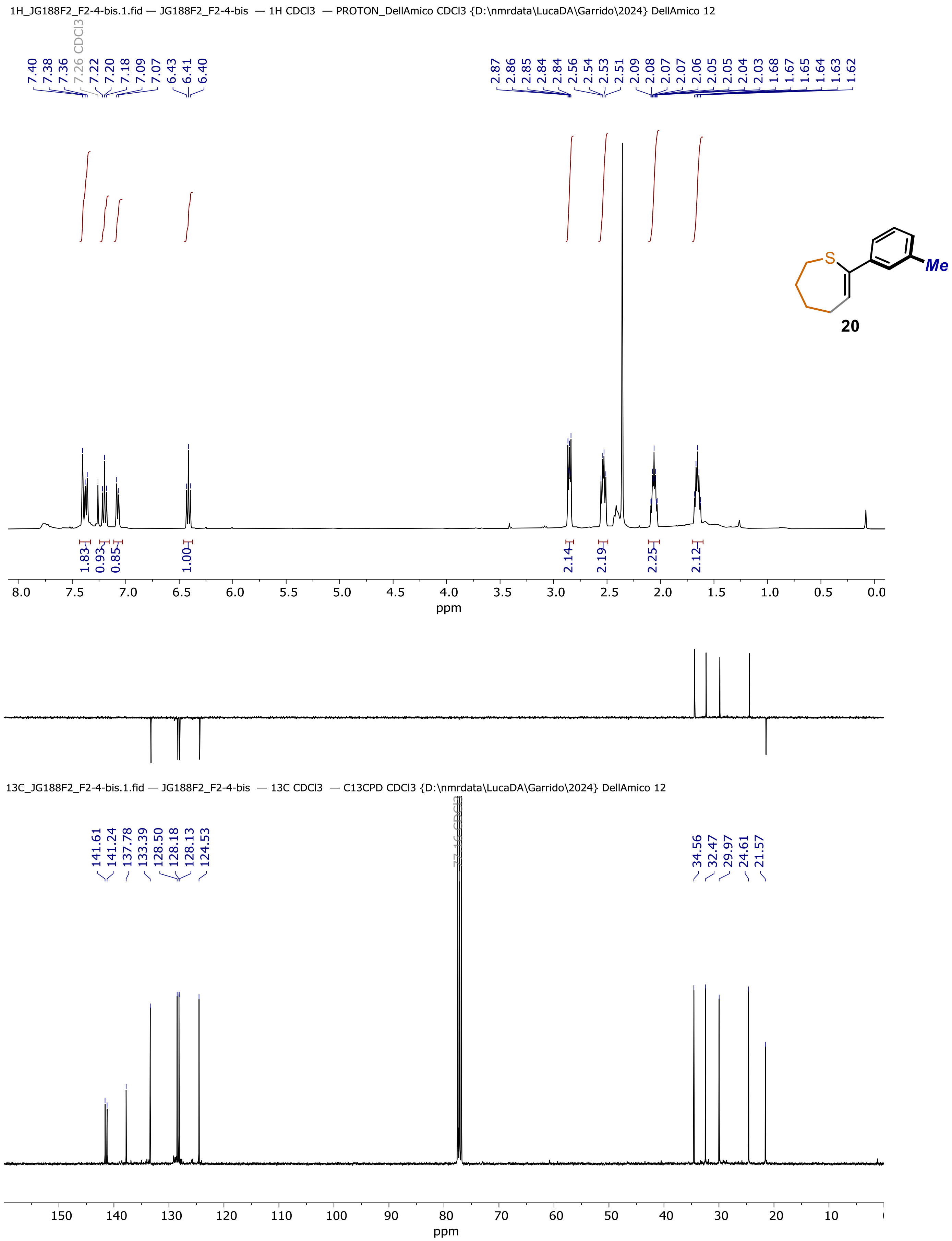

Figure S35. *^1H and ^{13}C NMR spectra of compound* ***20*** *in $CDCl_3$.*

7-(*o*-tolyl)-2,3,4,5-tetrahydrothiepine 21

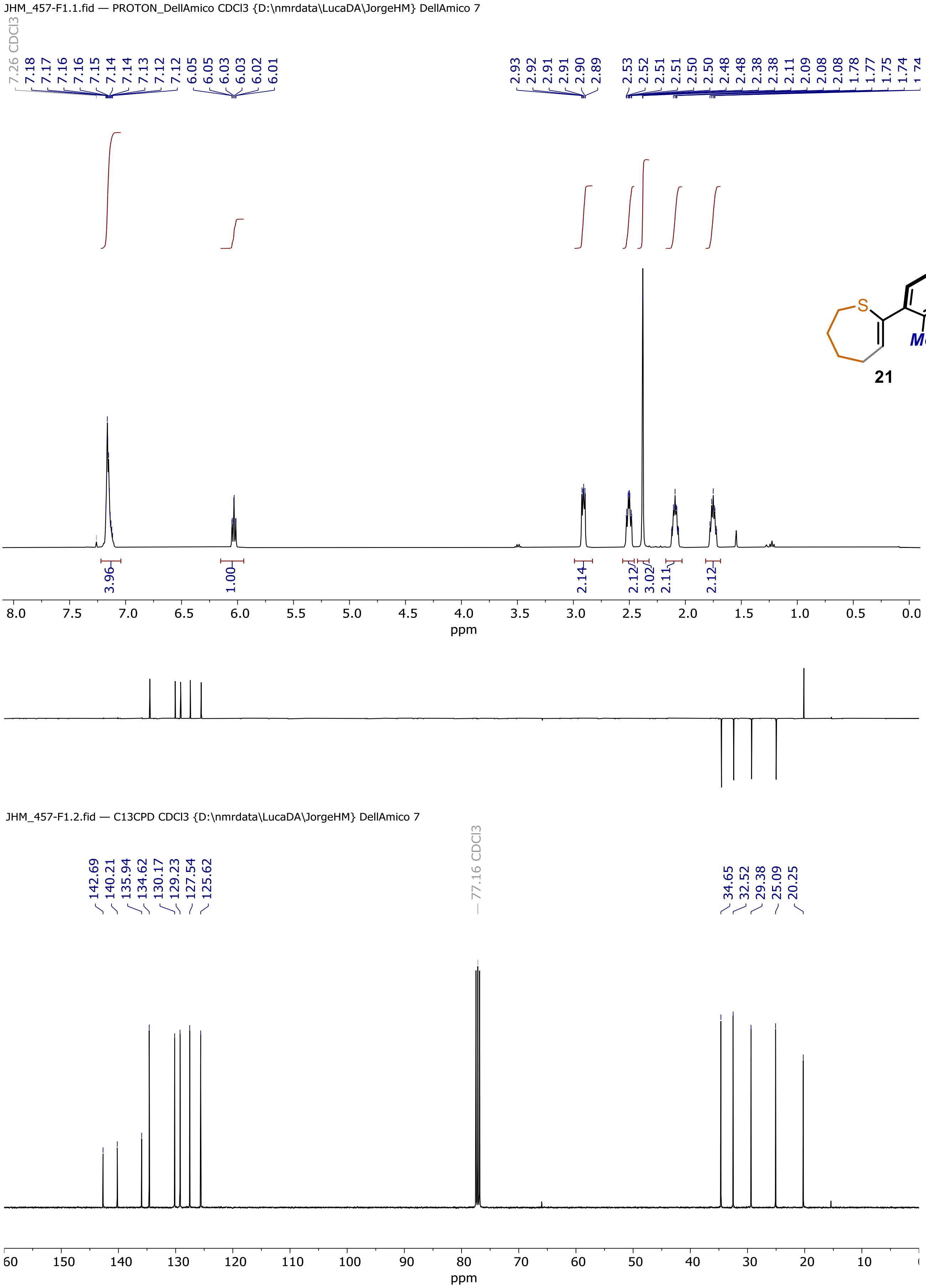

Figure S36. *^{1}H and ^{13}C NMR spectra of compound **21** in $CDCl_3$.*

6,7-diphenyl-2,3,4,5-tetrahydrothiepine 22

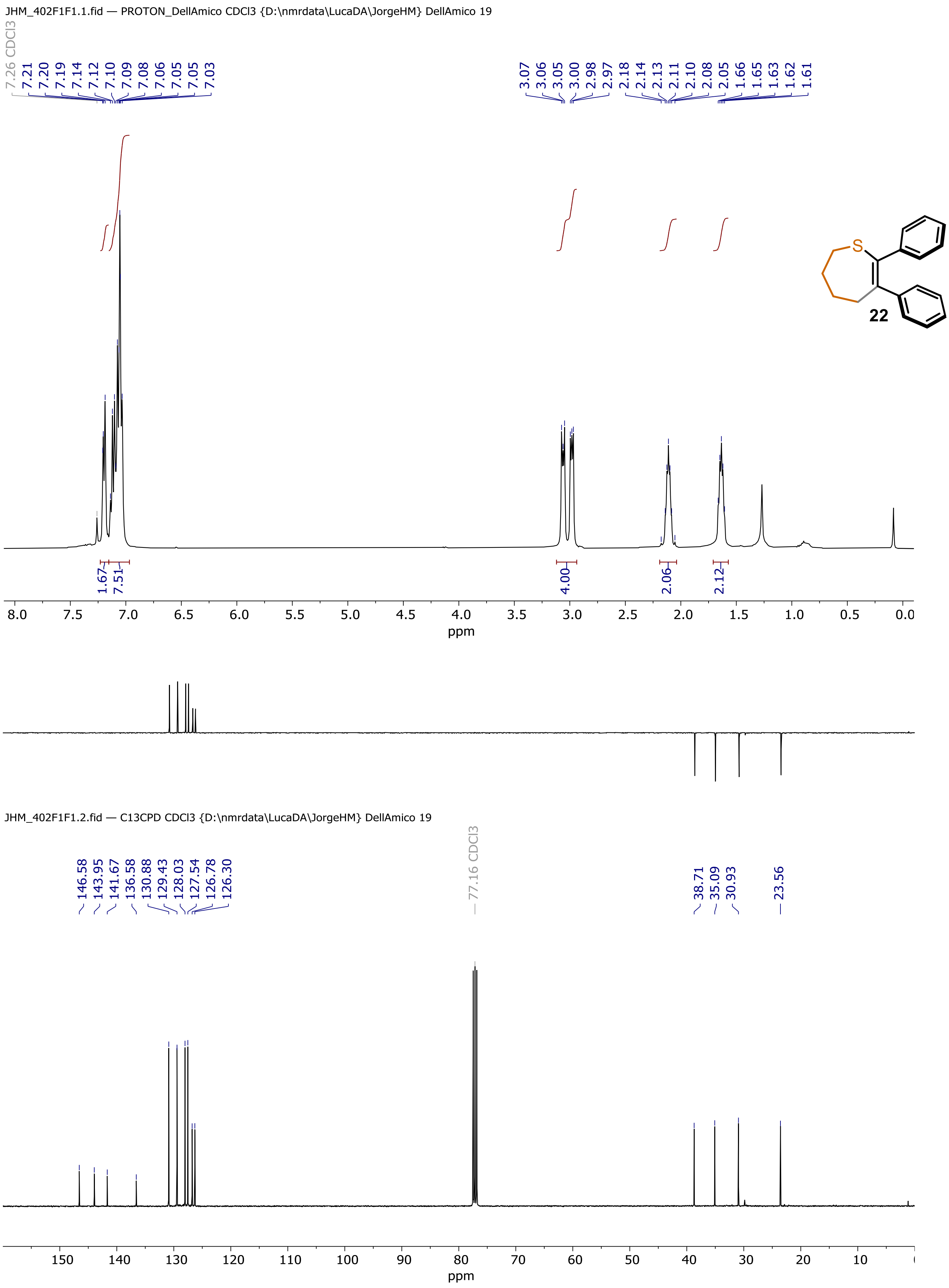

Figure S37. *^{1}H and ^{13}C NMR spectra of compound **22** in $CDCl_3$.*

2,3,4,5-tetrahydronaphtho[1,2-b]thiepine 23

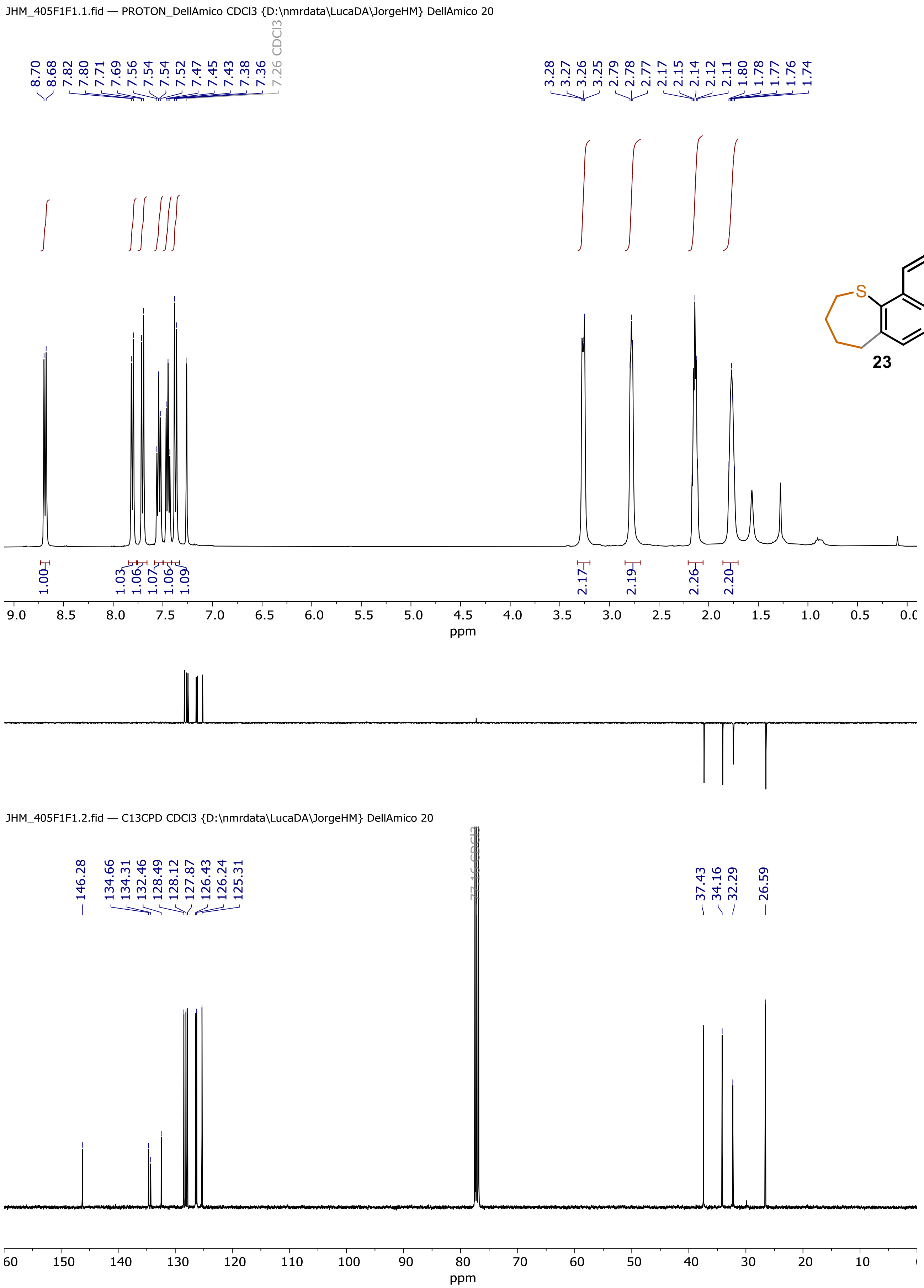

Figure S38. *^1H and ^{13}C NMR spectra of compound **23** in $CDCl_3$.*

2,3,4,5,6,7-hexahydronaphtho[1,2-b]thiepine 24

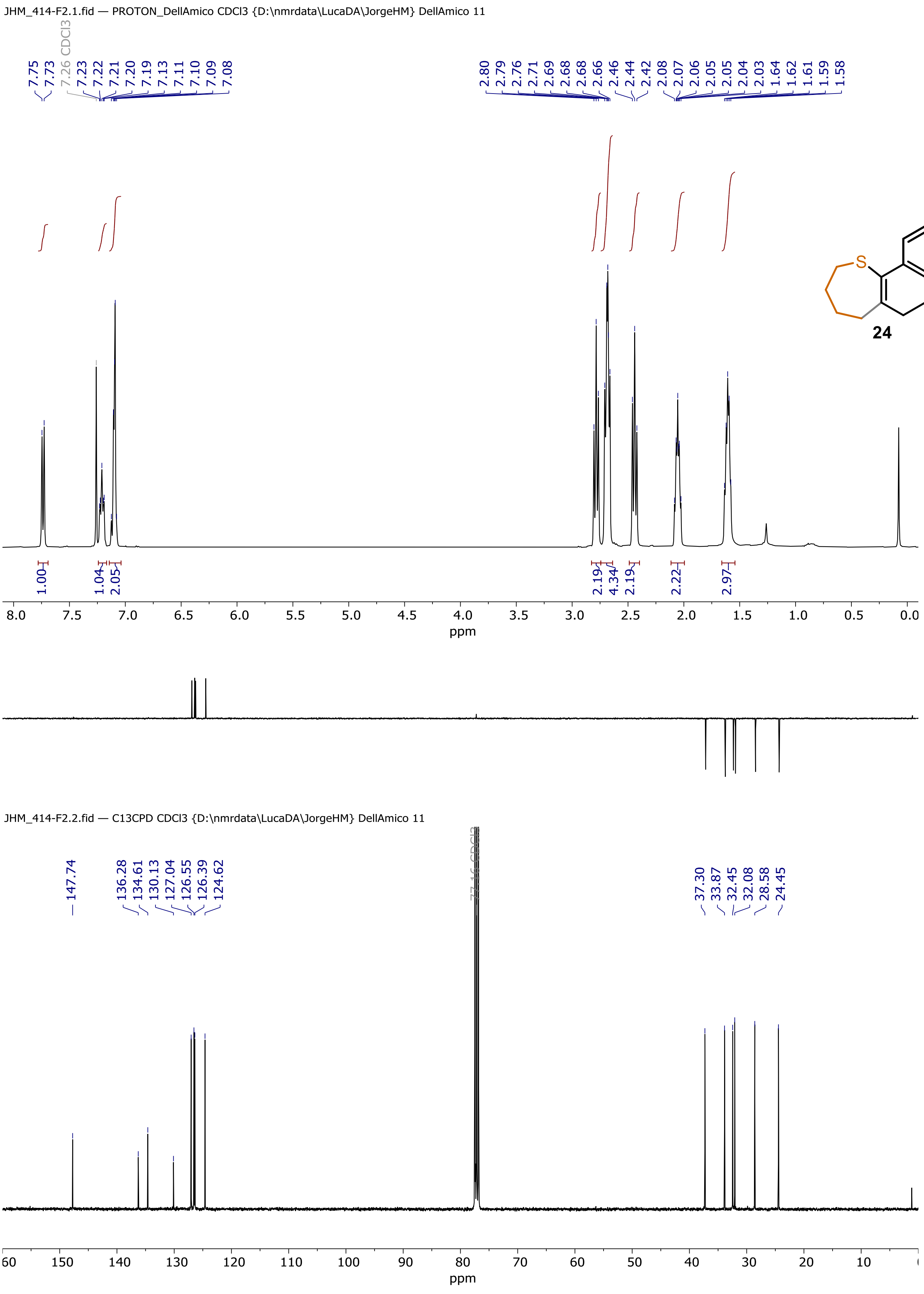

Figure S39. *^1H and ^{13}C NMR spectra of compound **24** in $CDCl_3$.*

7-(naphthalen-2-yl)-2,3,4,5-tetrahydrothiepine26

KMU-01-160R-P1YP2-1H.1.fid — PROTON_DellAmico CDCl3 {D:\nmrdata\LucaDA\KMU} DellAmico 18

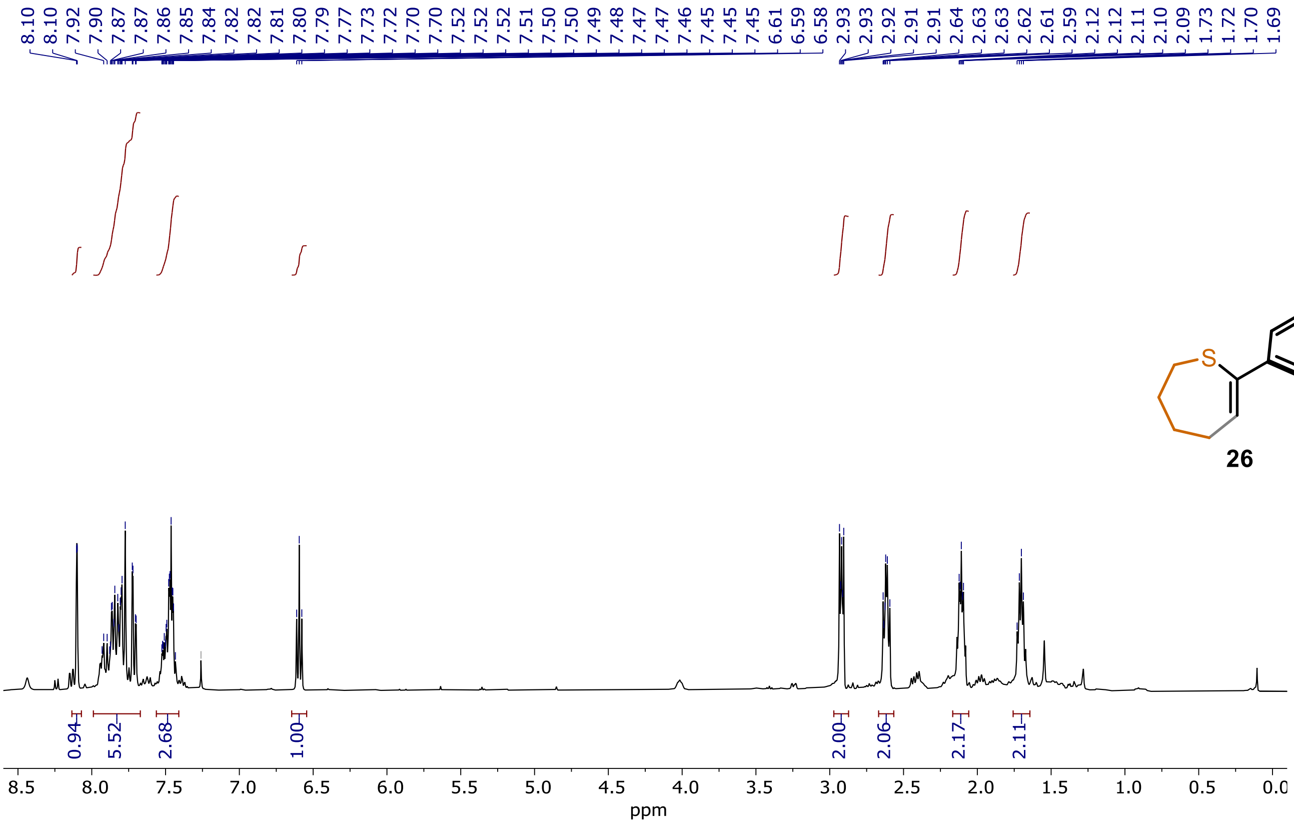

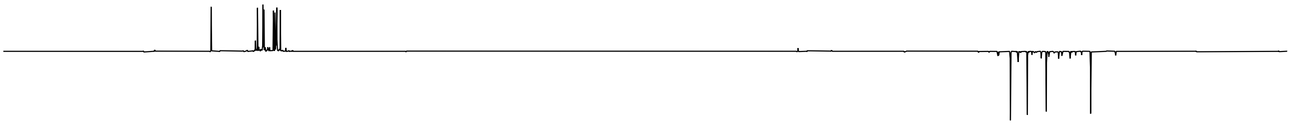

KMU-01-160R-P1.1.fid — C13CPD CDCl3 {D:\nmrdata\LucaDA\KMU} DellAmico 19

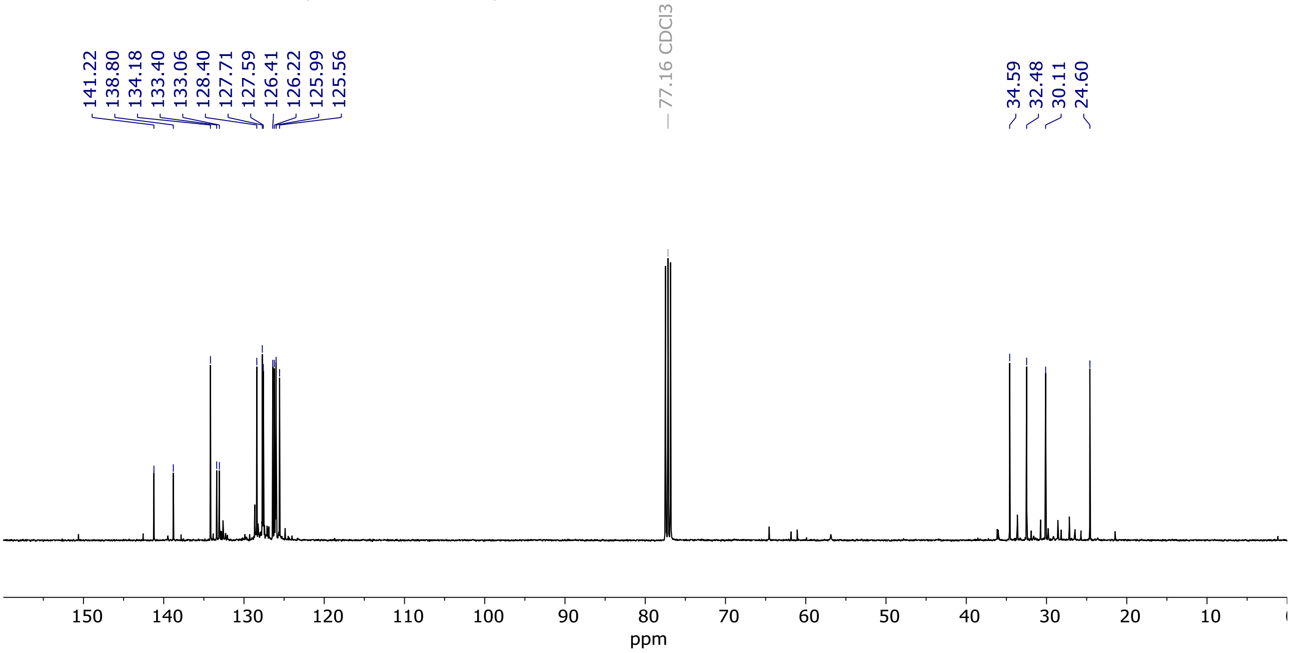

Figure S40. *^1H and ^{13}C NMR spectra of compound **26** in $CDCl_3$.*

1-phenyl-2-(2-phenylthiepan-2-yl)ethan-1-one 32

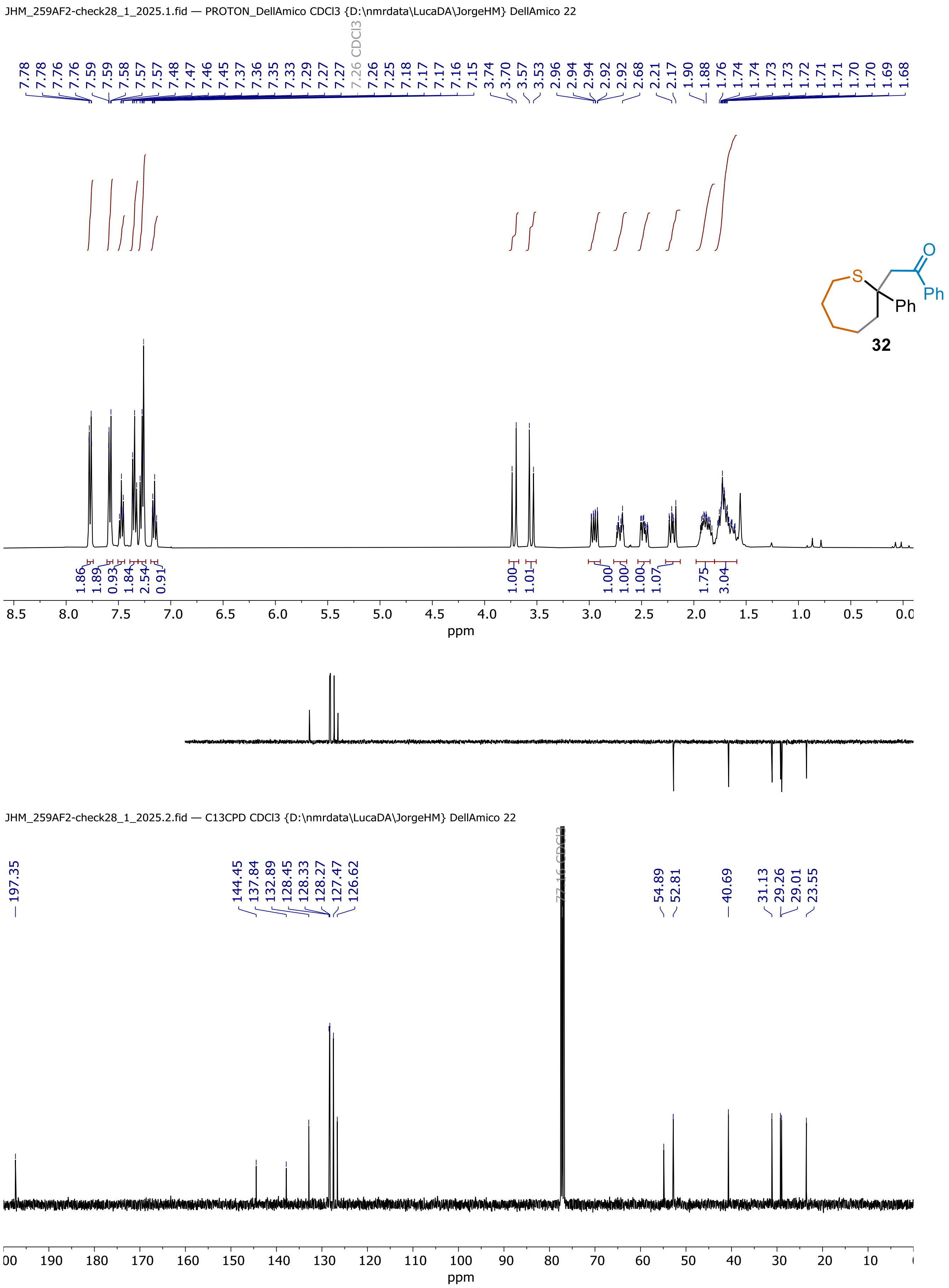

Figure S41. *¹H and ¹³C NMR spectra of compound **32** in CDCl₃.*

(*E*)-1-styryltetrahydro-1*H*-thiophen-1-ium hexafluorophosphate (V) 33

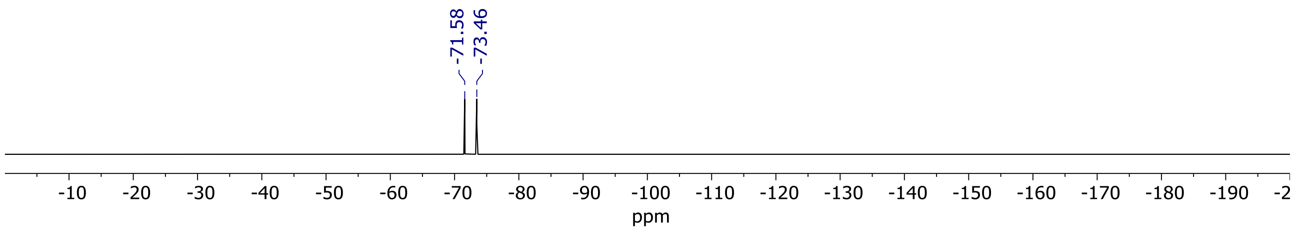

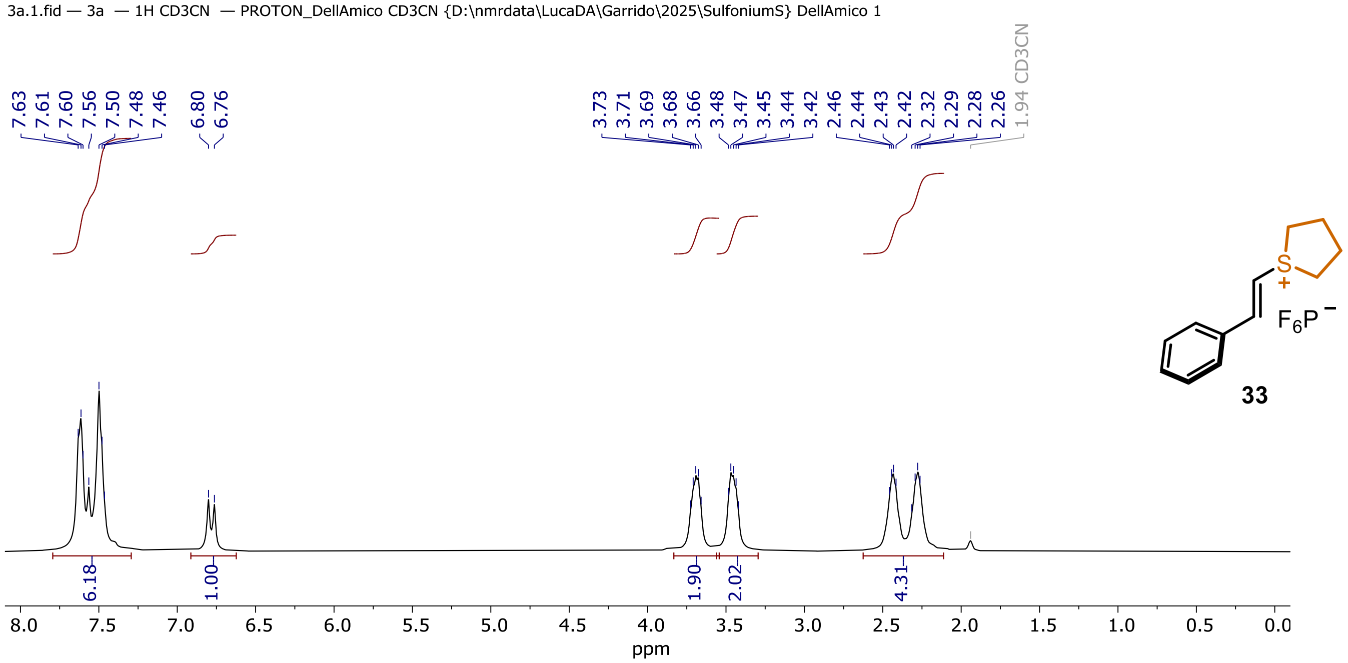

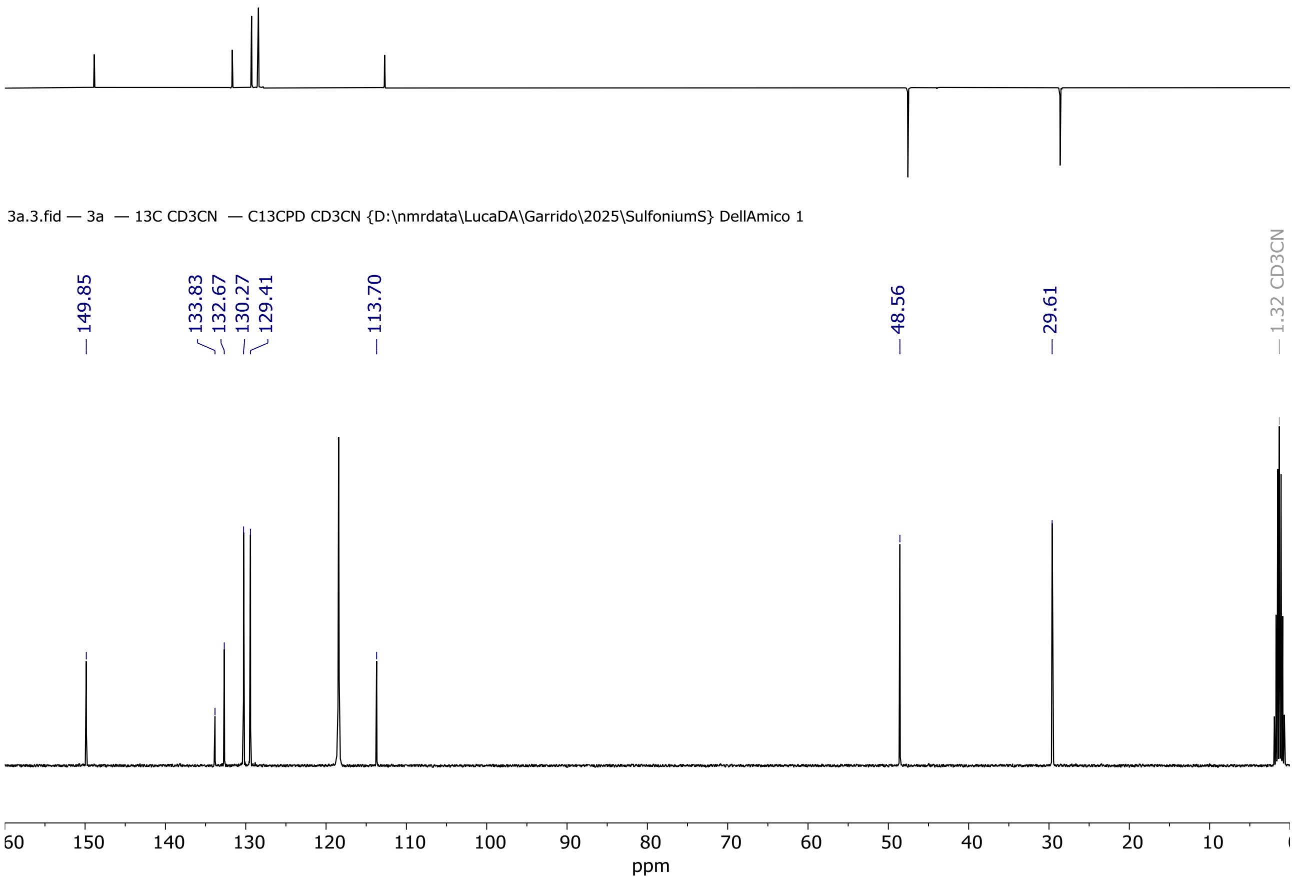

Figure S42. *^{19}F, ^{1}H and ^{13}C NMR spectra of compound **33** in CD_3CN.*

(*E*)-1-(4-fluorostyryl)tetrahydro-1*H*-thiophen-1-ium hexafluorophosphate (V) 34

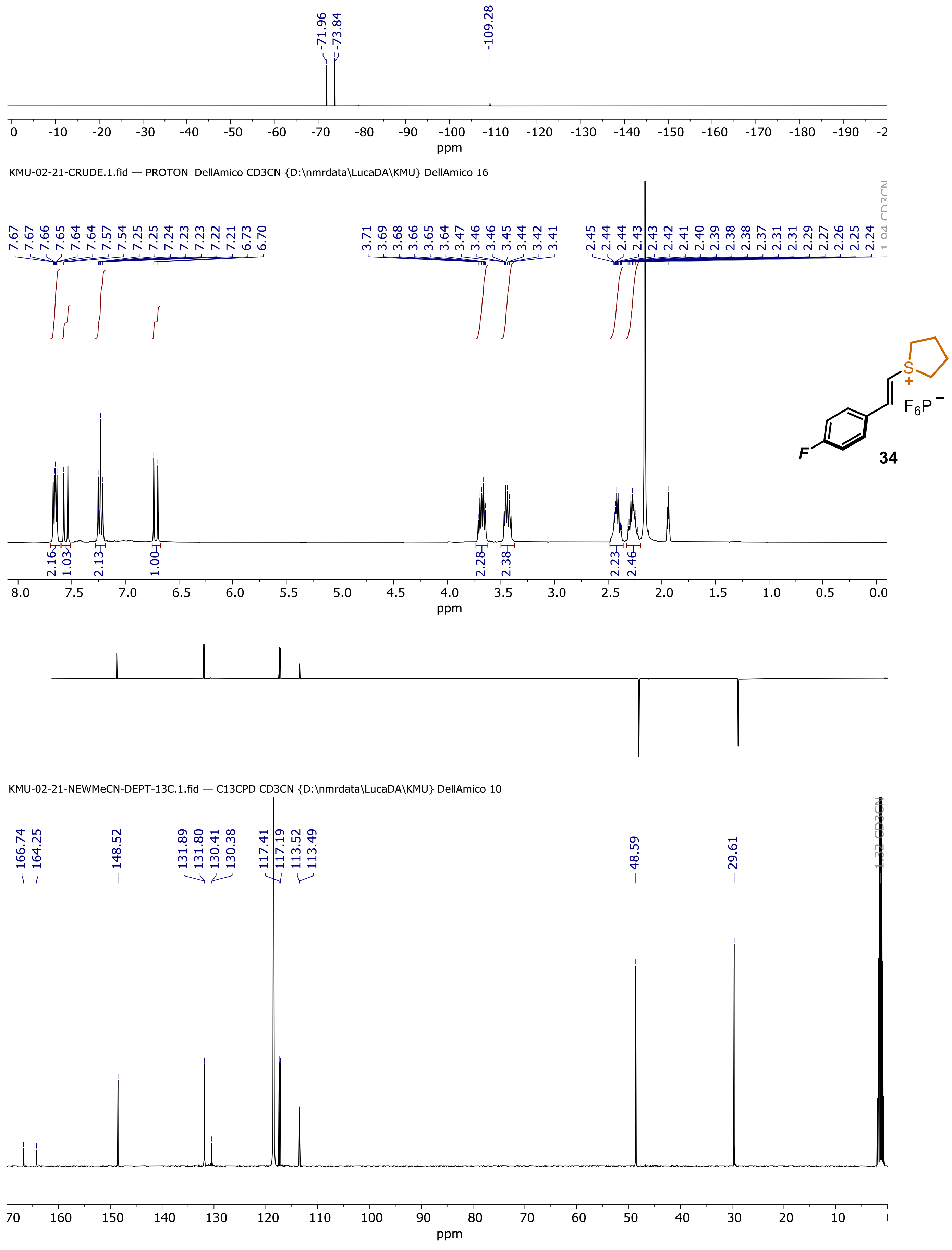

Figure S43. *^{19}F, ^{1}H and ^{13}C NMR spectra of compound* ***34*** *in CD_3CN.*

(*E*)-1-(4-chlorostyryl)tetrahydro-1*H*-thiophen-1-ium hexafluorophosphate (V) 35

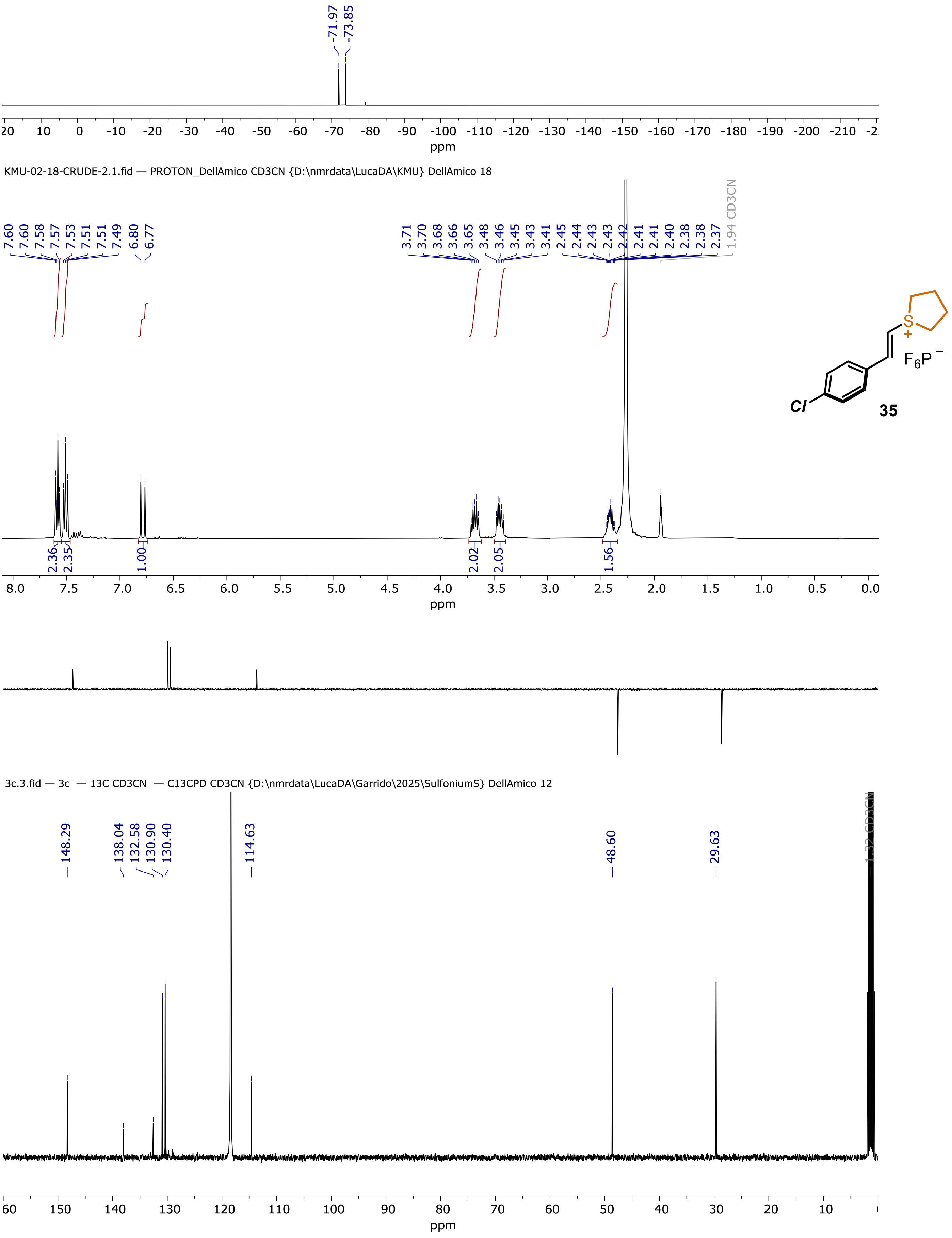

Figure S44. *^{19}F, ^{1}H and ^{13}C NMR spectra of compound* ***35*** *in CD_3CN.*

(*E*)-1-(4-bromostyryl)tetrahydro-1*H*-thiophen-1-ium hexafluorophosphate (V) 36

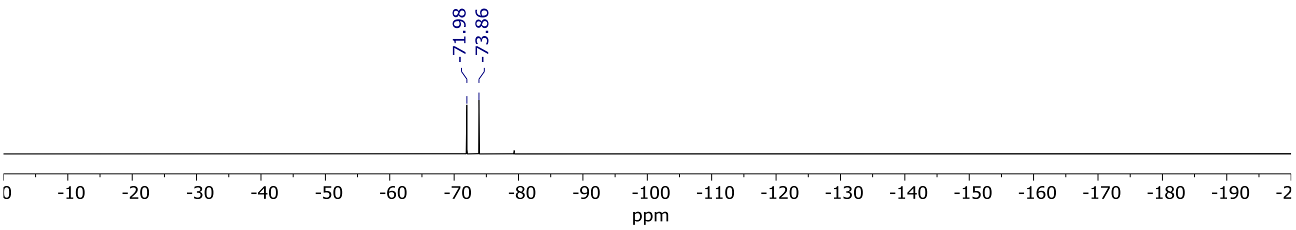

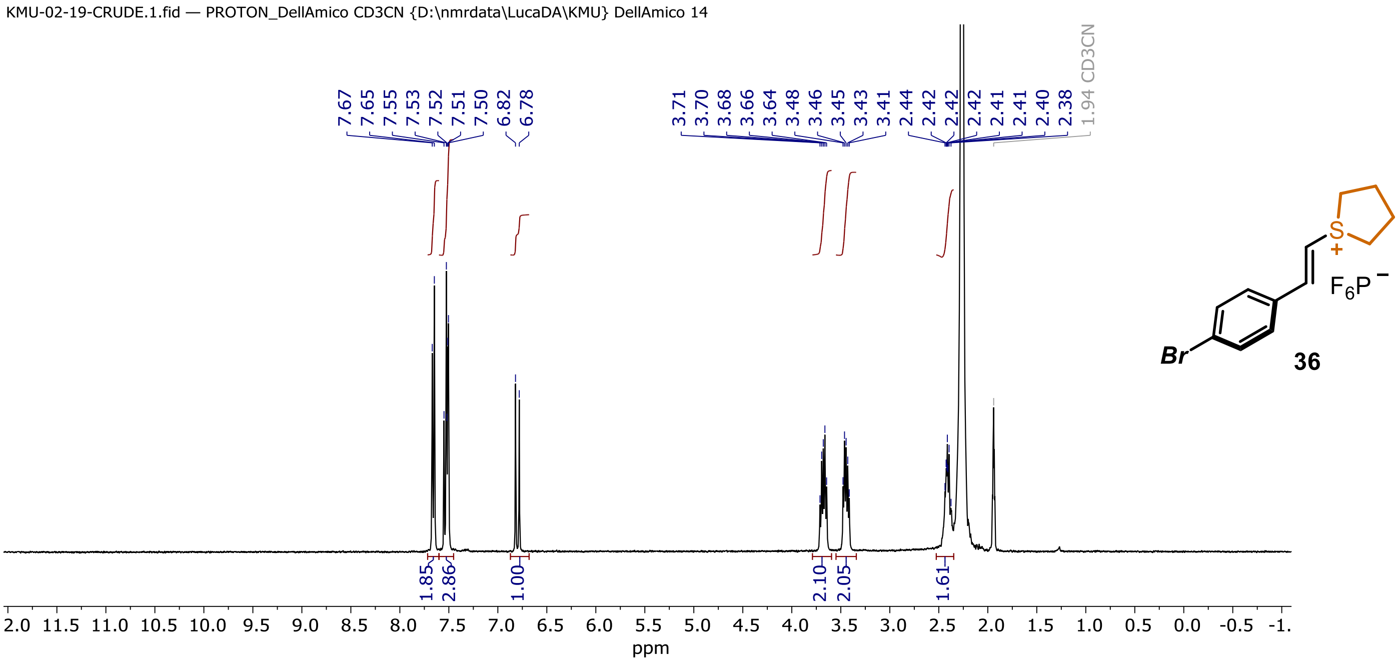

36

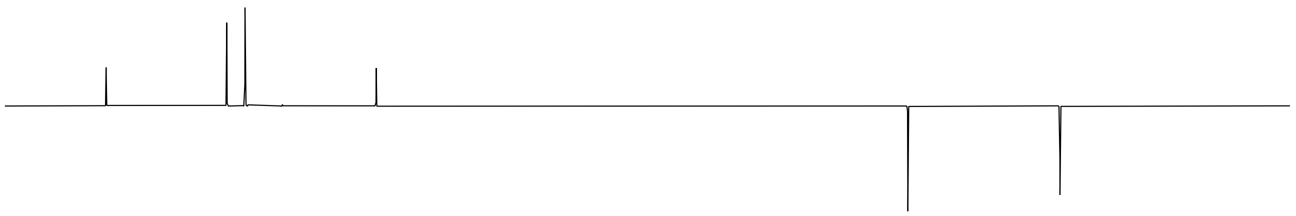

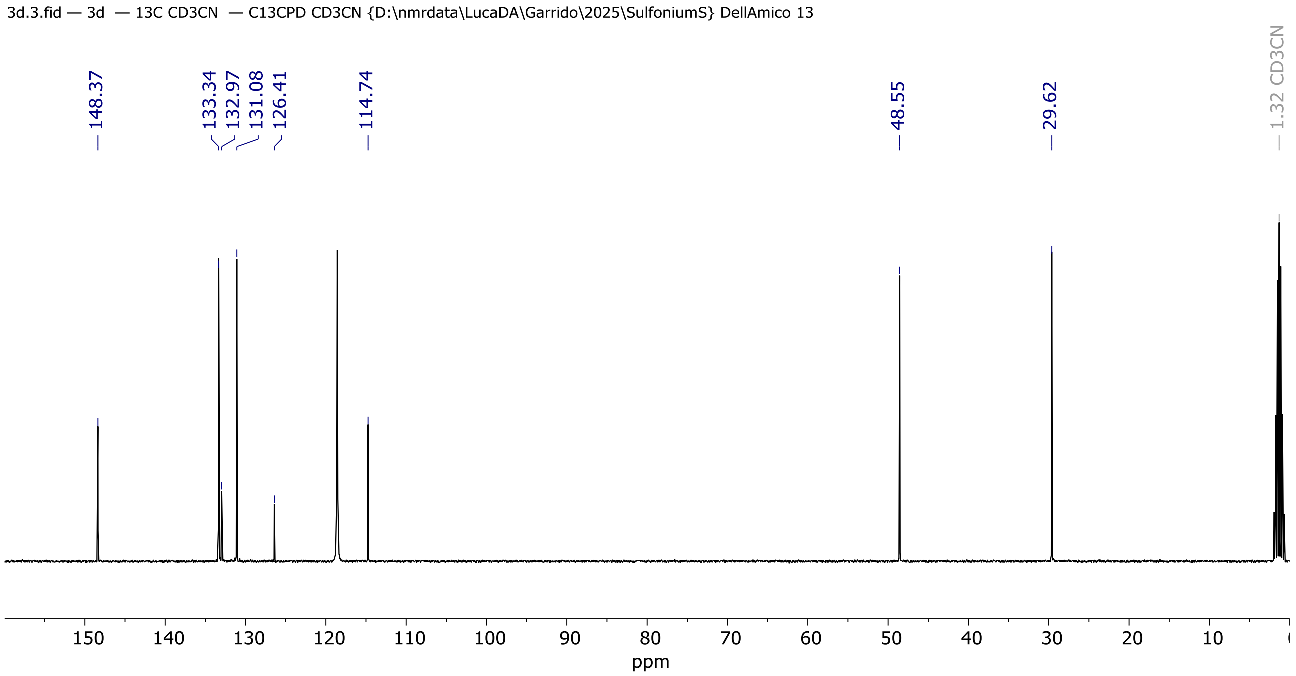

Figure S45. *^{19}F, ^{1}H and ^{13}C NMR spectra of compound **36** in CD_3CN.*

(*E*)-1-(4-cyanostyryl)tetrahydro-1*H*-thiophen-1-ium hexafluorophosphate (V) 37

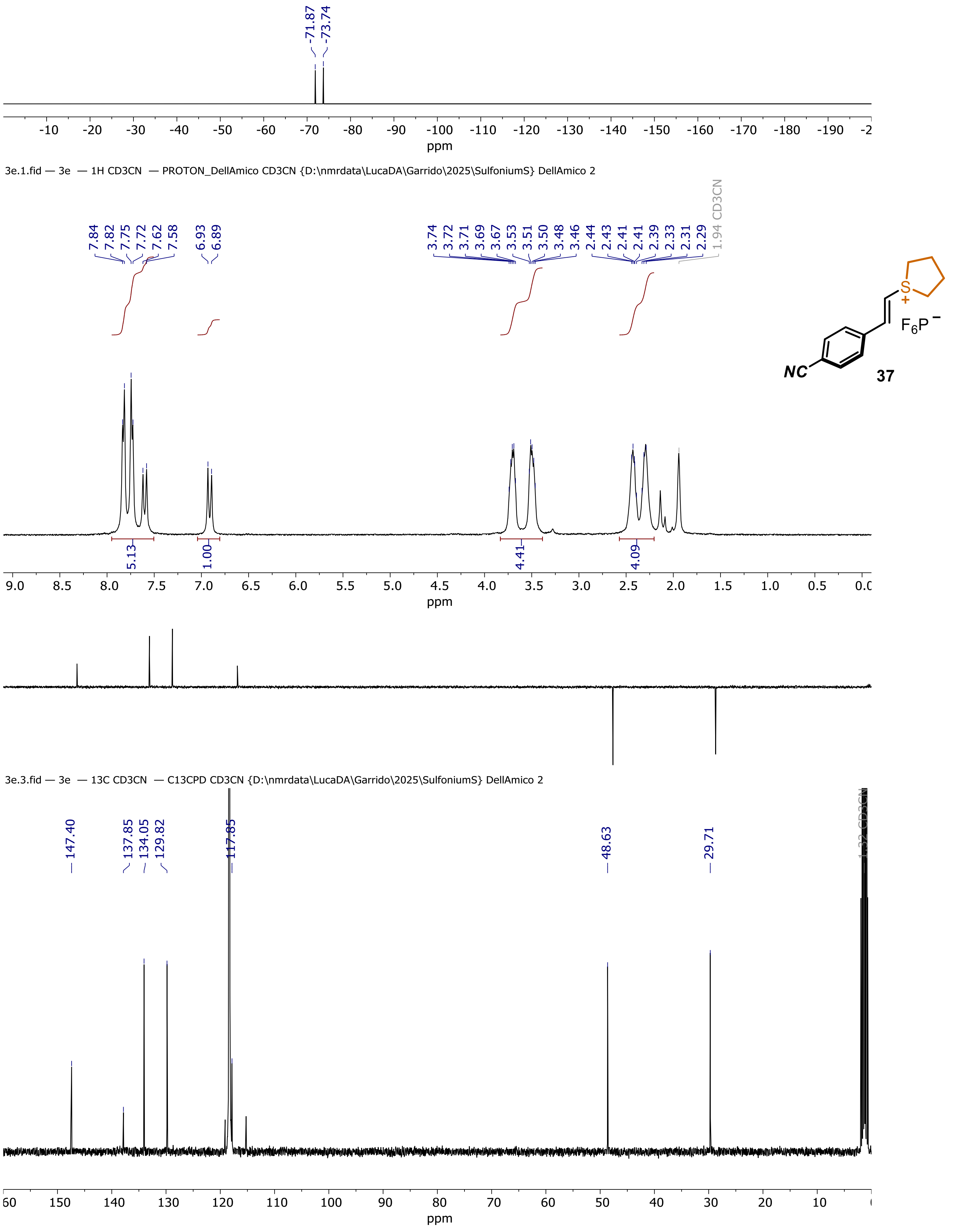

Figure S46. *^{19}F, ^{1}H and ^{13}C NMR spectra of compound **37** in CD_3CN.*

(*E*)-1-(2-([1,1'-biphenyl]-4-yl)vinyl)tetrahydro-1*H*-thiophen-1-ium hexafluorophosphate (V) 38

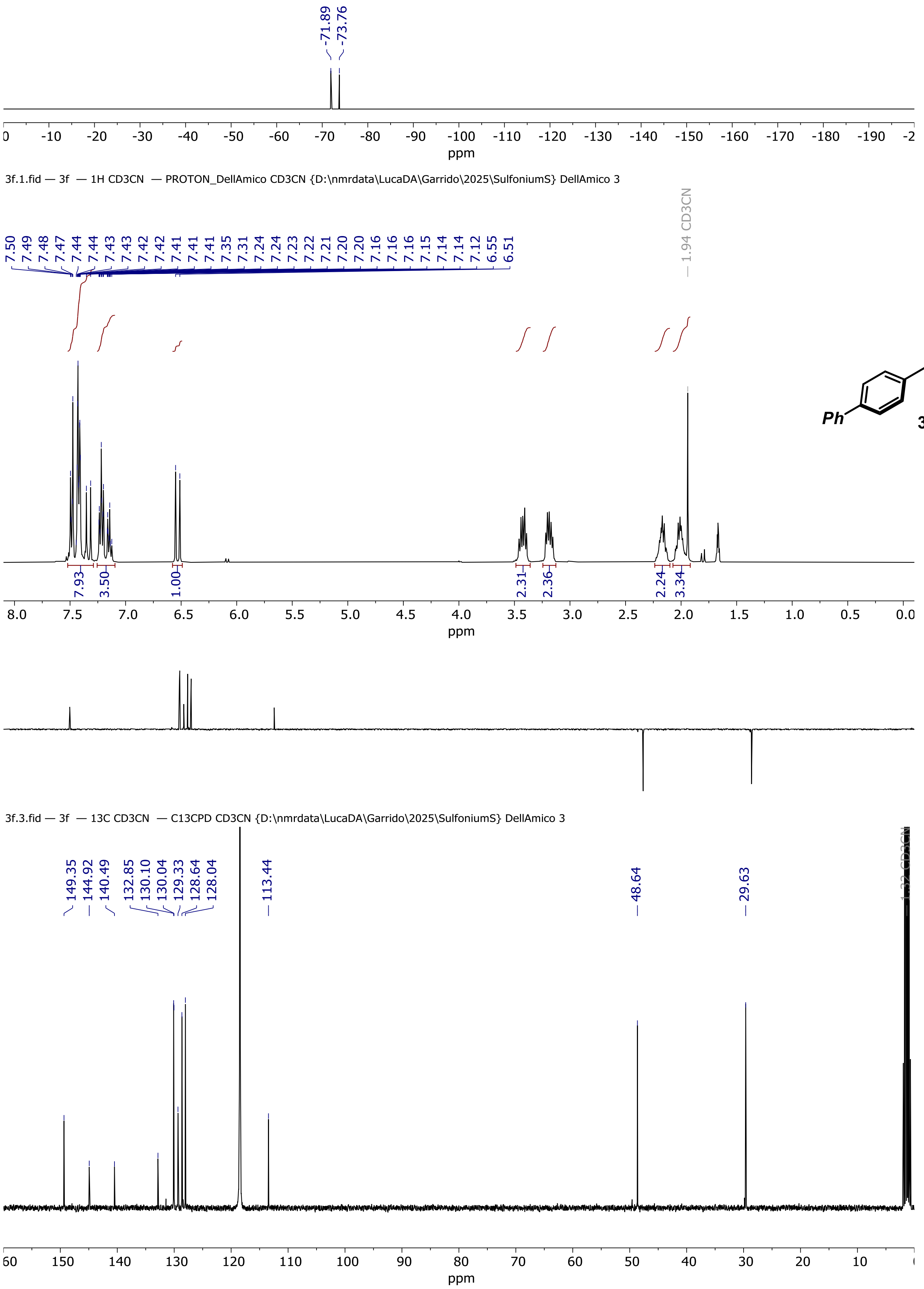

Figure S47. *^{19}F, ^{1}H and ^{13}C NMR spectra of compound* ***38*** *in CD_3CN.*

(*E*)-1-(2-([1,1'-biphenyl]-4-yl)vinyl)tetrahydro-1*H*-thiophen-1-ium hexafluorophosphate (V) 38

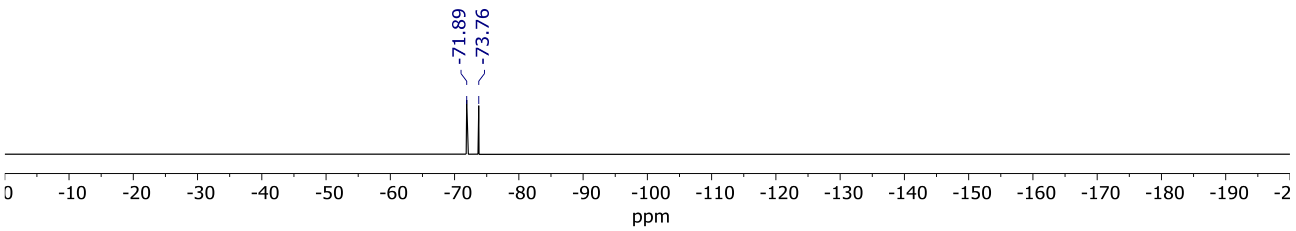

3f.1.fid — 3f — 1H CD3CN — PROTON_DellAmico CD3CN {D:\nmrdata\LucaDA\Garrido\2025\SulfoniumS} DellAmico 3

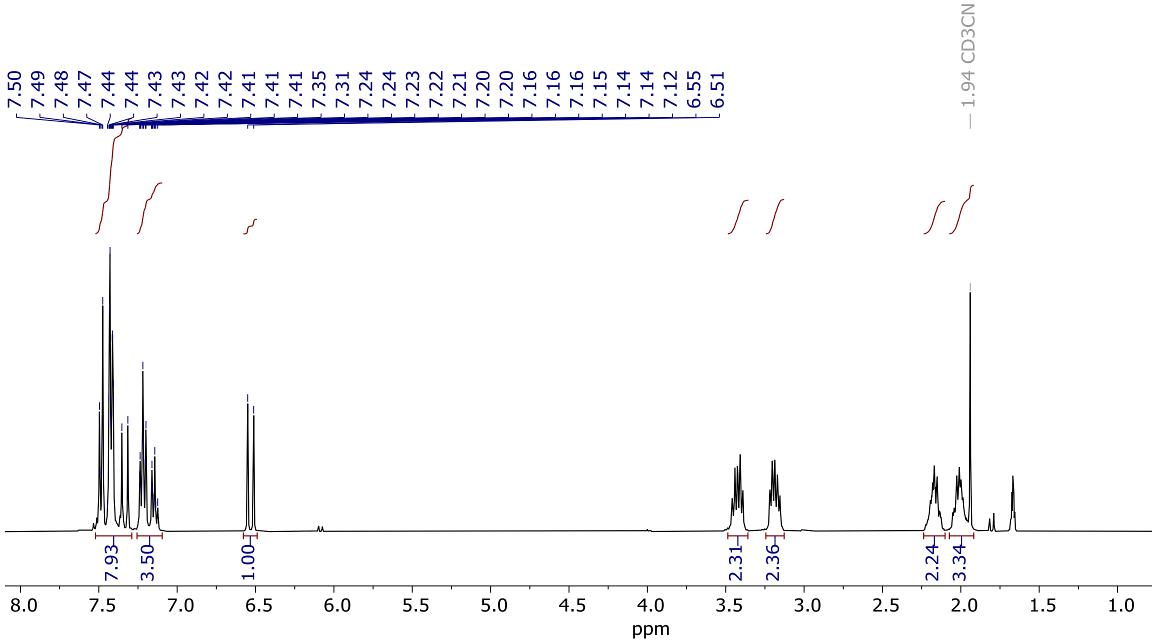

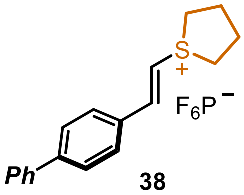

38

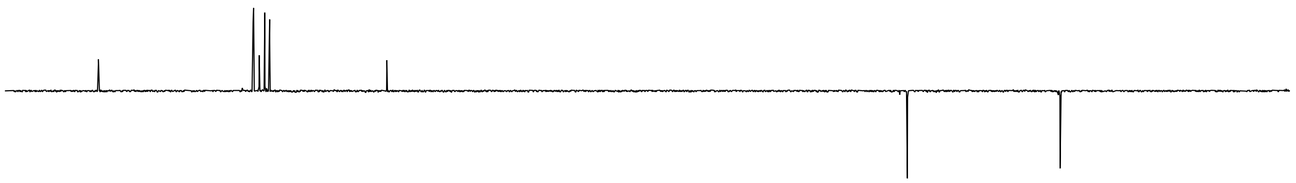

3f.3.fid — 3f — 13C CD3CN — C13CPD CD3CN {D:\nmrdata\LucaDA\Garrido\2025\SulfoniumS} DellAmico 3

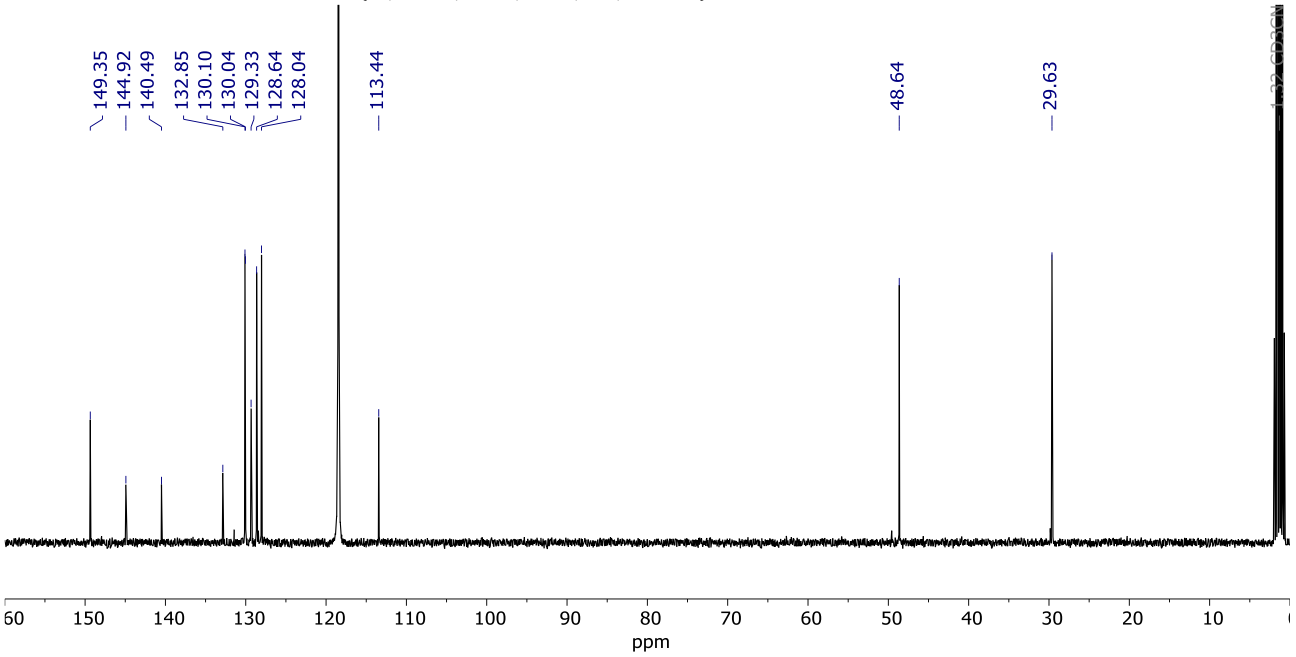

Figure S48. *^{19}F, ^{1}H and ^{13}C NMR spectra of compound* ***38*** *in CD_3CN.*

(*E*)-1-(3-methylstyryl)tetrahydro-1*H*-thiophen-1-ium hexafluorophosphate (V) 40

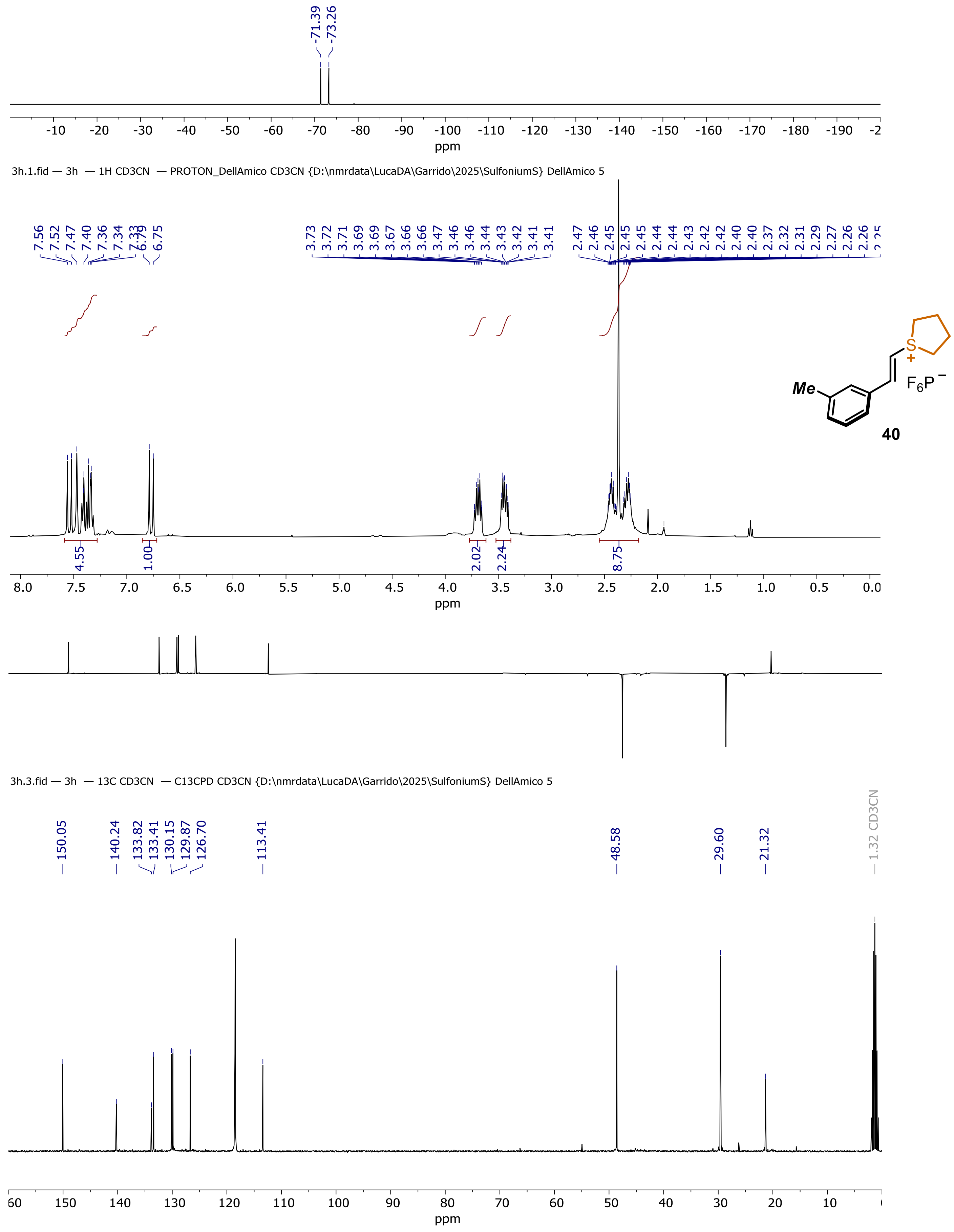

Figure S49. *^{19}F, ^{1}H and ^{13}C NMR spectra of compound* ***40*** *in CD_3CN.*

(*E*)-1-(2-methylstyryl)tetrahydro-1*H*-thiophen-1-ium hexafluorophosphate (V) 41

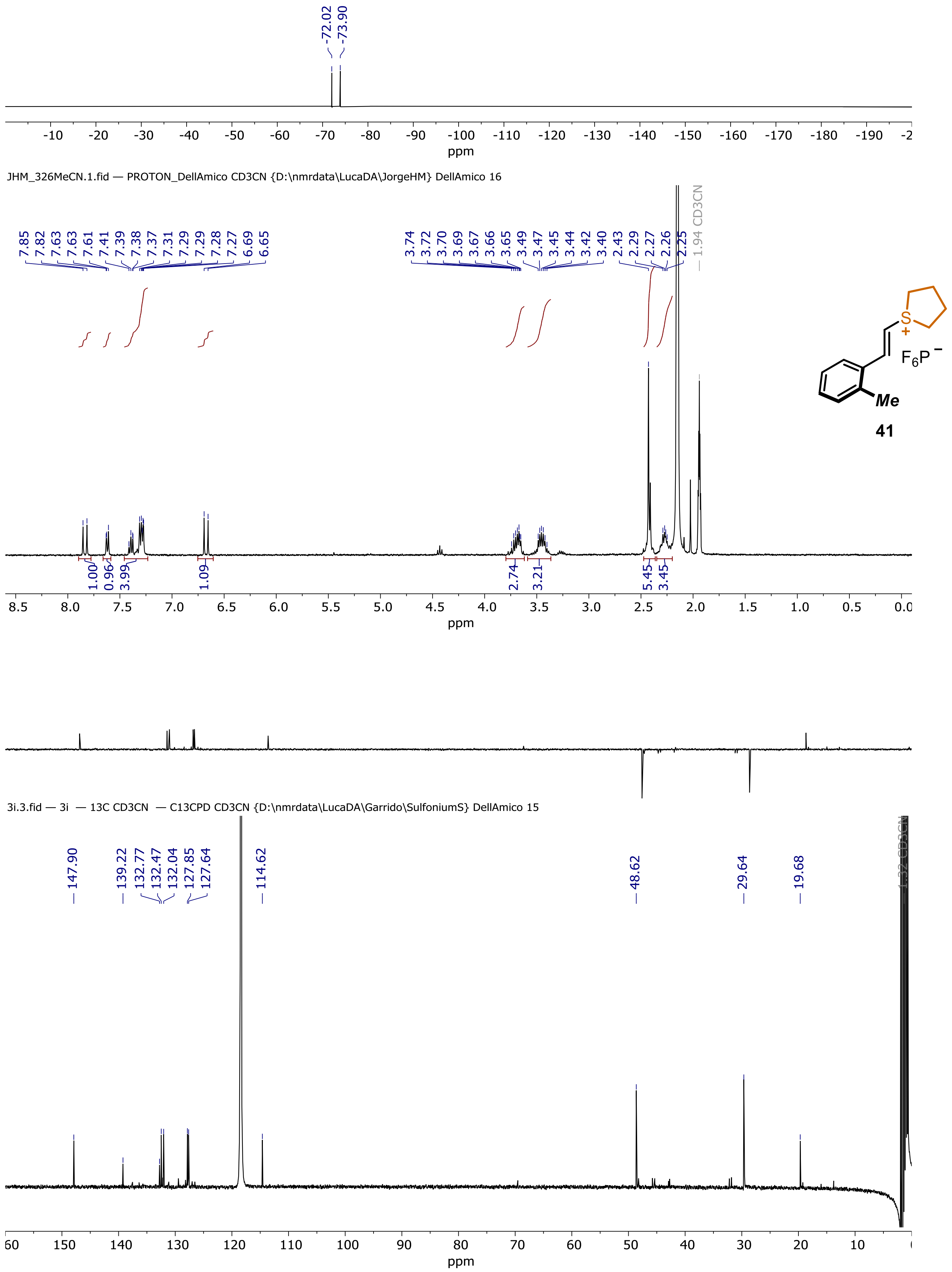

Figure S50. *^{19}F, ^{1}H and ^{13}C NMR spectra of compound* ***41*** *in CD_3CN.*

1-(2,2-diphenylvinyl)tetrahydro-1*H*-thiophen-1-ium hexafluorophosphate (V) 42

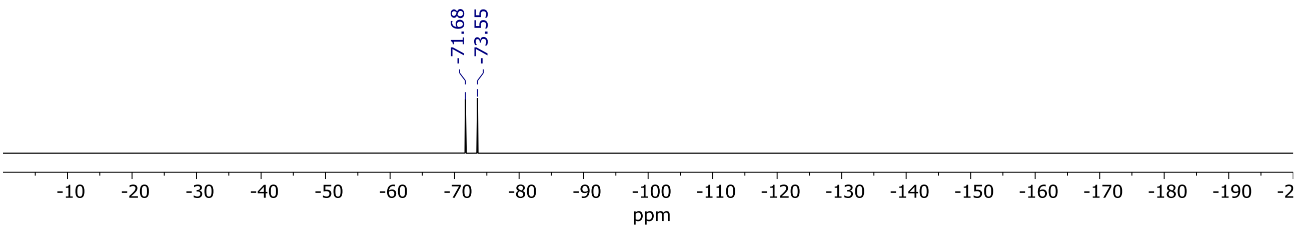

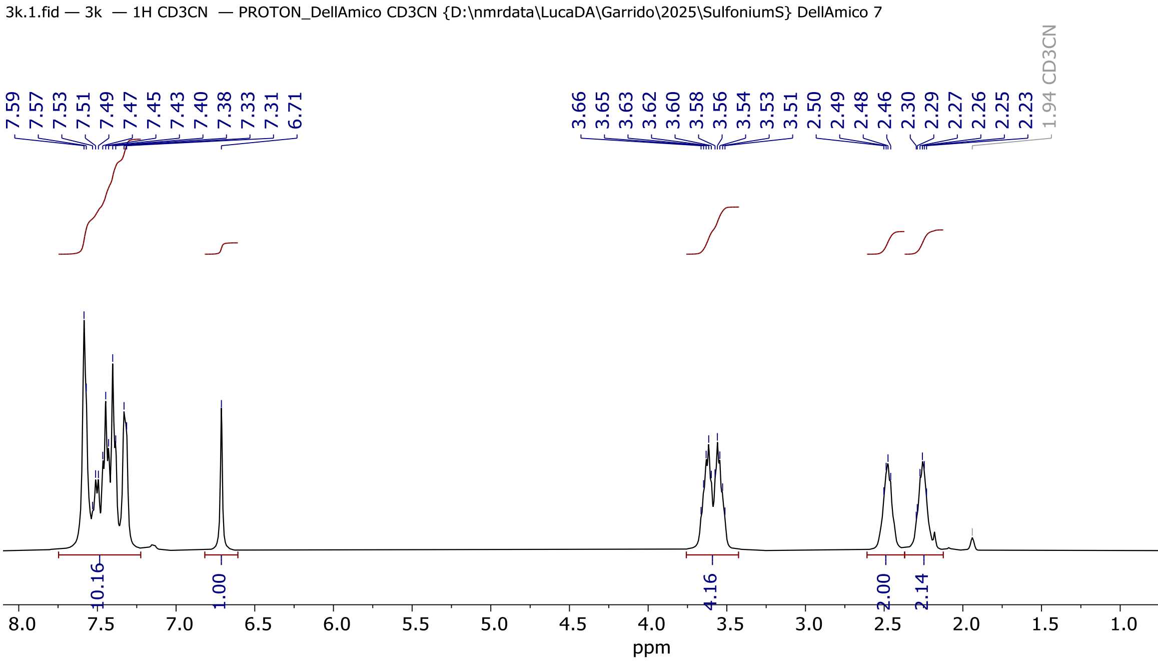

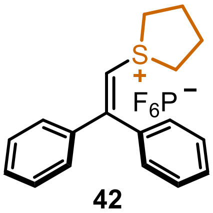

42

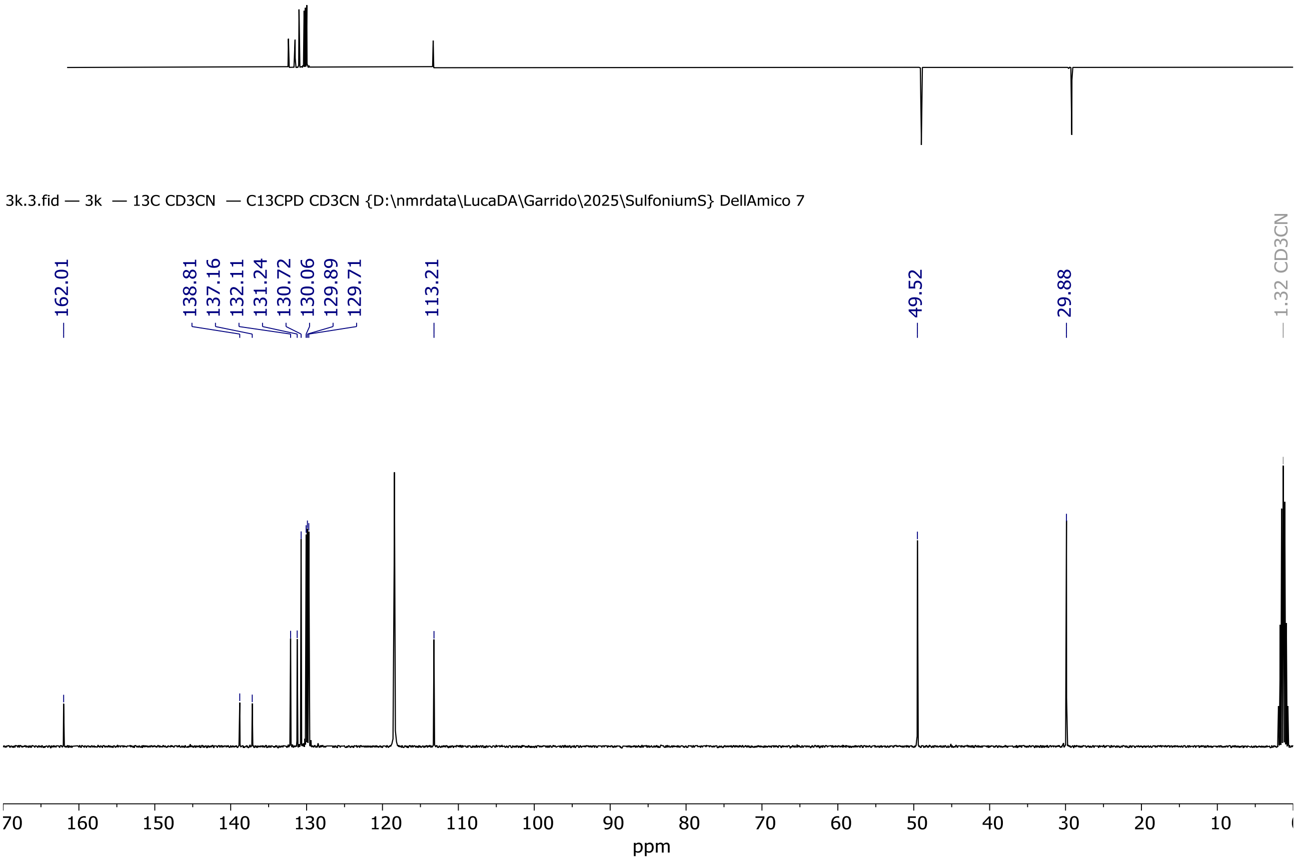

Figure S51. *^{19}F, ^{1}H and ^{13}C NMR spectra of compound **42** in CD_3CN.*

1-(3,4-dihydronaphthalen-2-yl)tetrahydro-1*H*-thiophen-1-ium hexafluorophosphate (V) 43

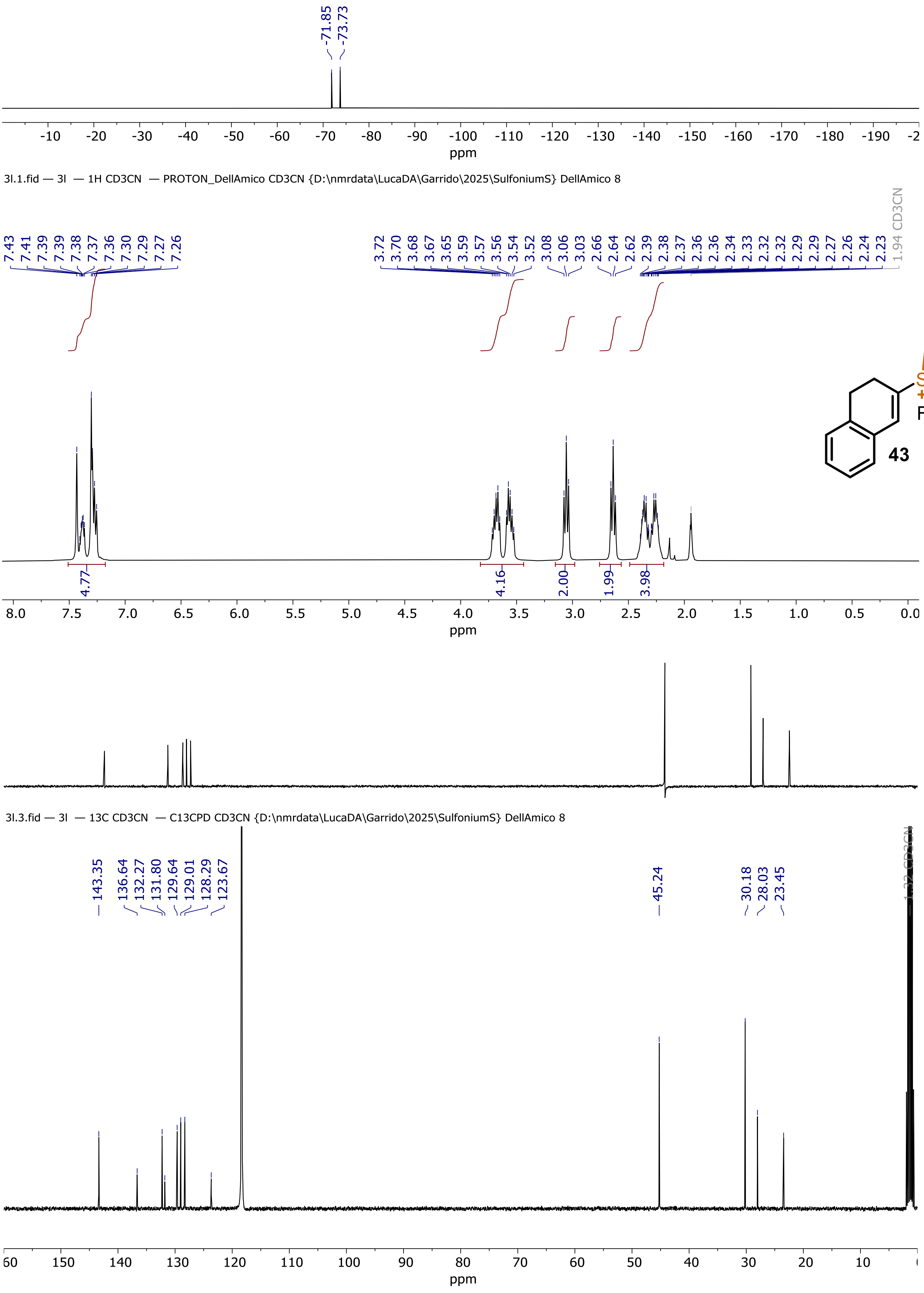

Figure S52. *^{19}F, ^{1}H and ^{13}C NMR spectra of compound* ***43*** *in CD_3CN.*

(*E*)-1-(2-fluorostyryl)tetrahydro-1*H*-thiophen-1-ium hexafluorophosphate (V) 44

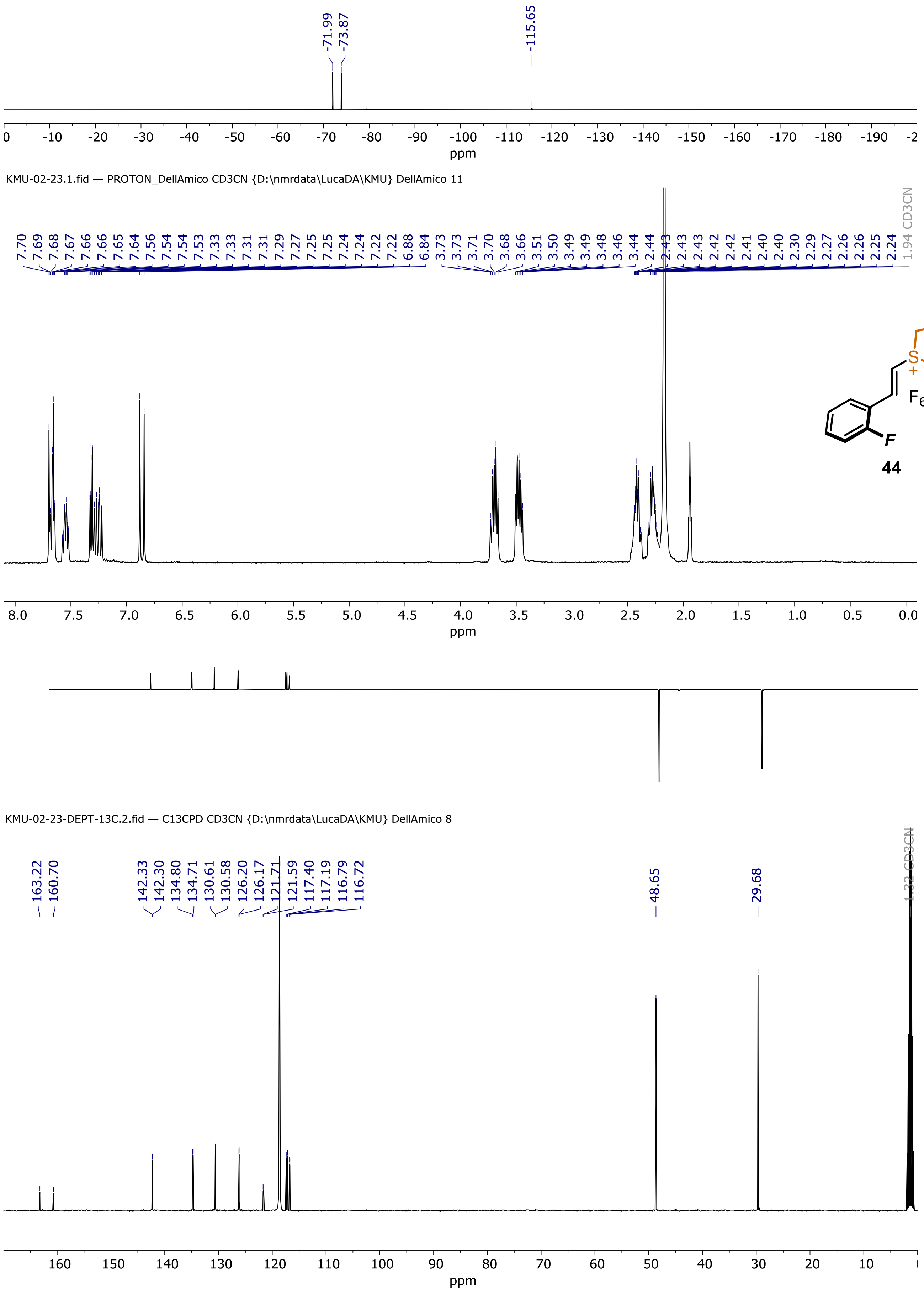

Figure S53. *^{19}F, ^{1}H and ^{13}C NMR spectra of compound **44** in CD_3CN.*

(*S*)-phenyl((*R*)-tetrahydro-2*H*-thiopyran-2-yl)methanol 45

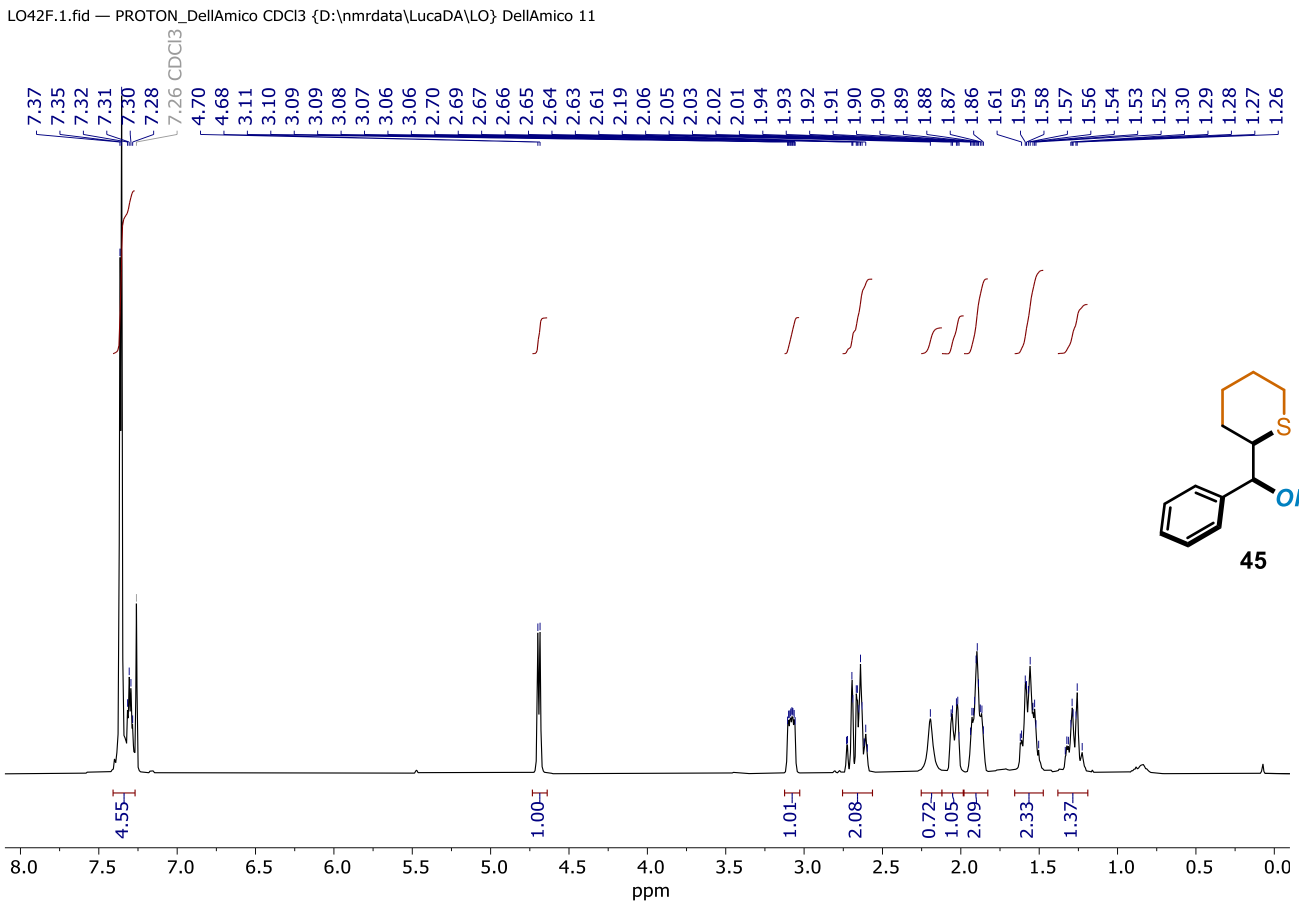

45

Figure S54. *^{1}H NMR spectrum of compound* ***45*** *in $CDCl_3$.*

(*S*)-(4-fluorophenyl)((*R*)-tetrahydro-2*H*-thiopyran-2-yl)methanol 46

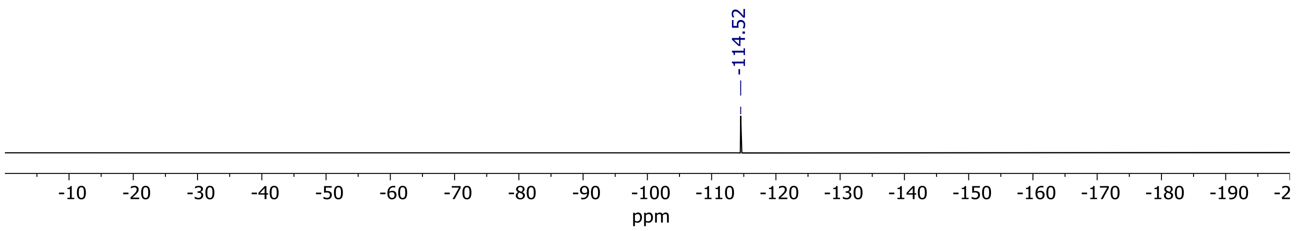

KMU-02-24PRODUCT-COSY.5.fid — PROTON CDCl3 {D:\nmrdata\LucaDA\KMU} DellAmico 2

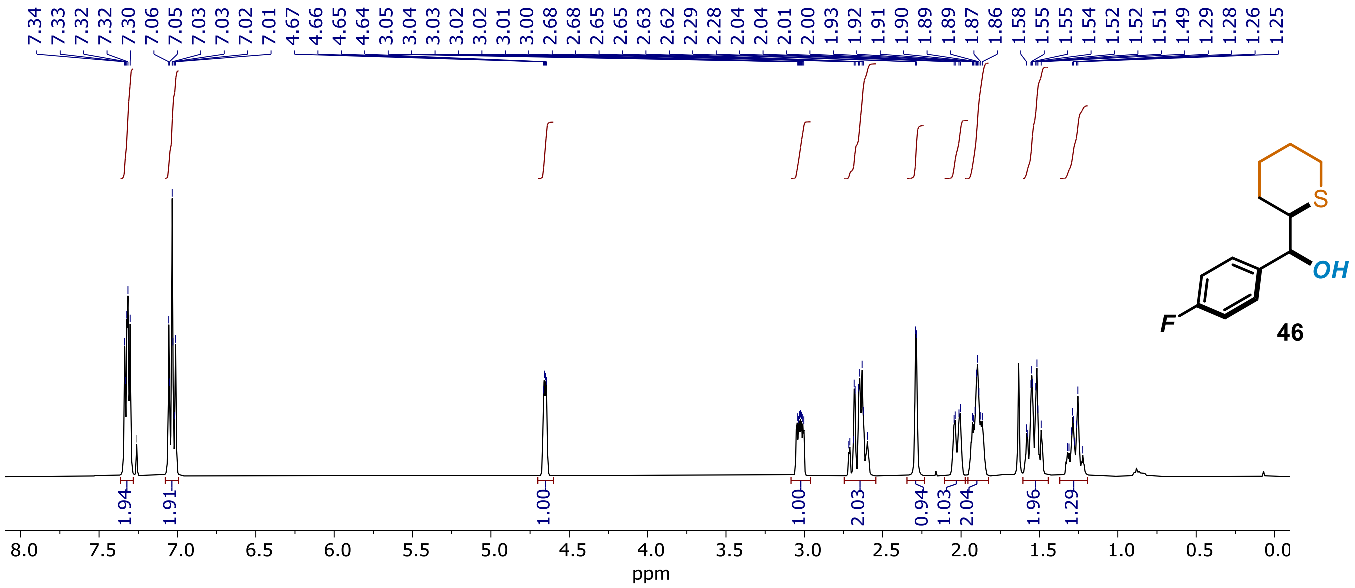

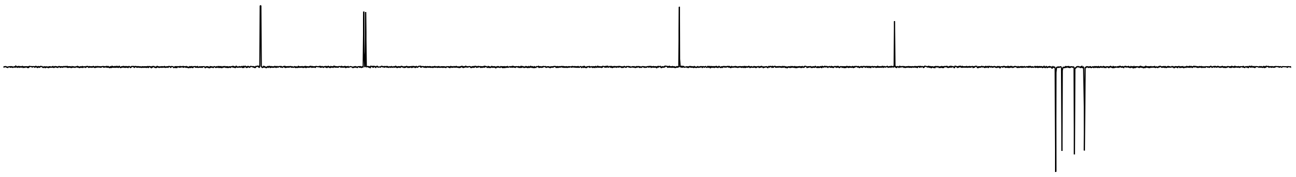

KMU-02-24PRODUCT.1.fid — C13CPD CDCl3 {D:\nmrdata\LucaDA\KMU} DellAmico 2

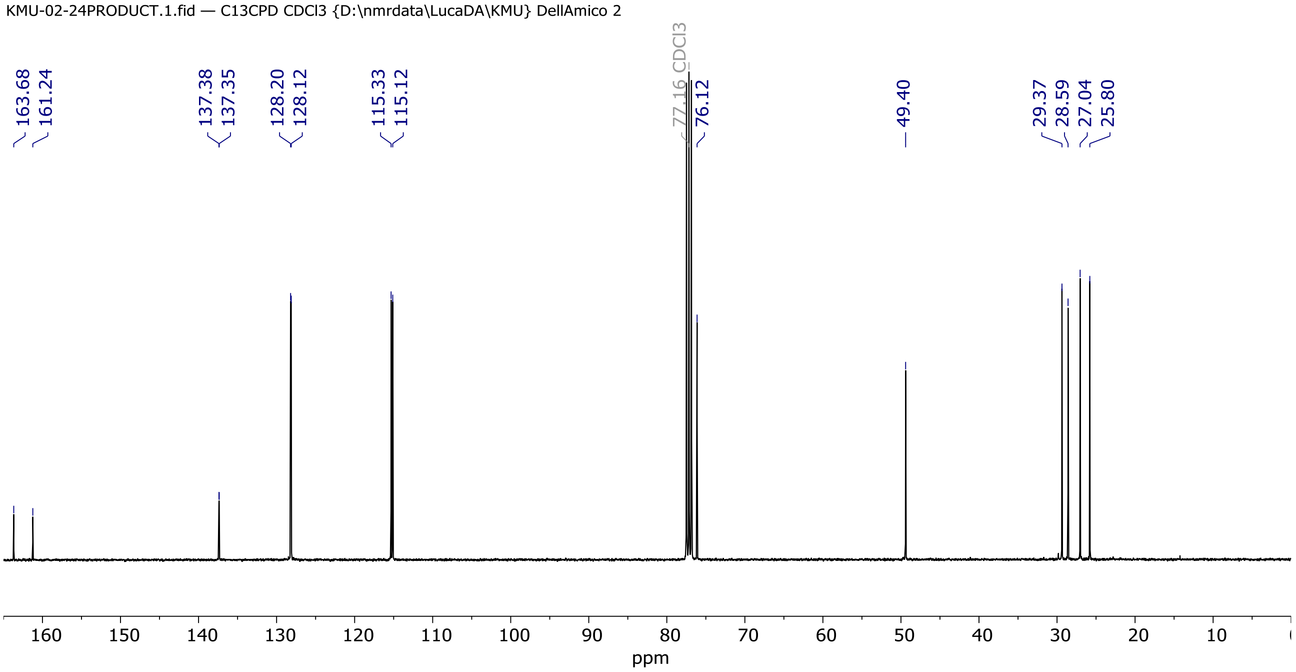

Figure S55. *^{1}H and ^{13}C NMR spectra of compound* ***46*** *in $CDCl_3$.*

(S)-(4-chlorophenyl)((R)-tetrahydro-2H-thiopyran-2-yl)methanol 47

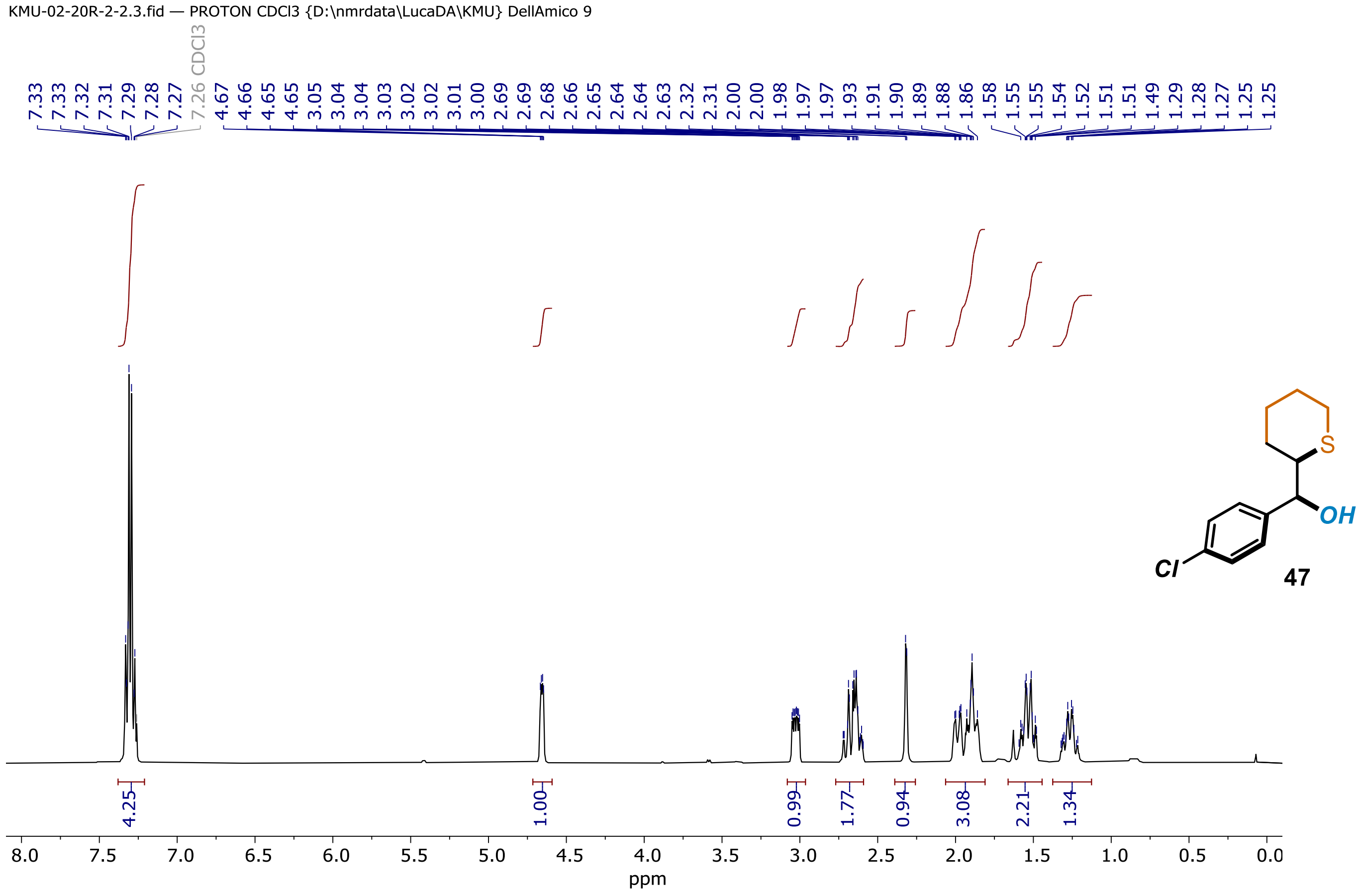

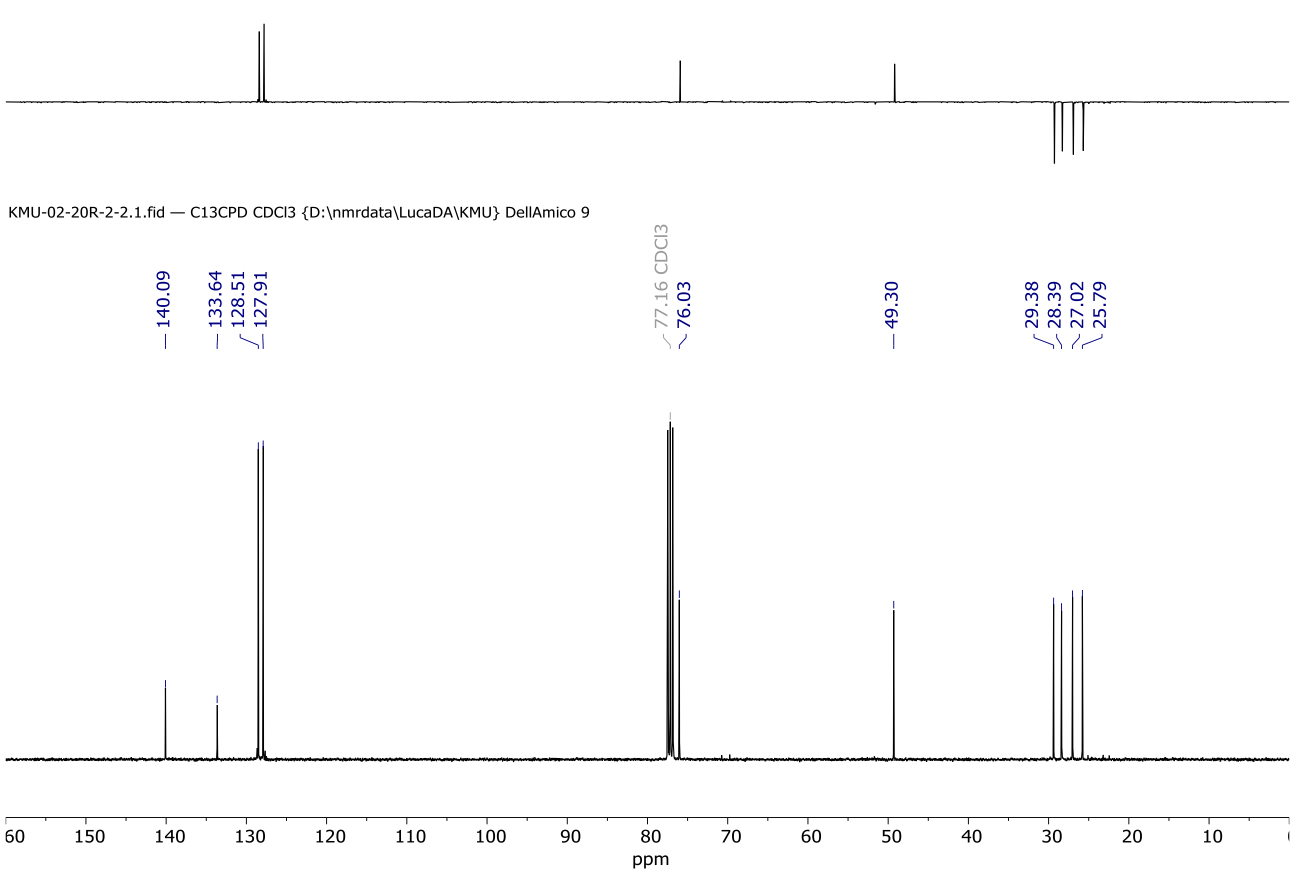

Figure S56. *^{1}H and ^{13}C NMR spectra of compound* ***47*** *in $CDCl_3$.*

(*S*)-(4-bromophenyl)((*R*)-tetrahydro-2*H*-thiopyran-2-yl)methanol 48

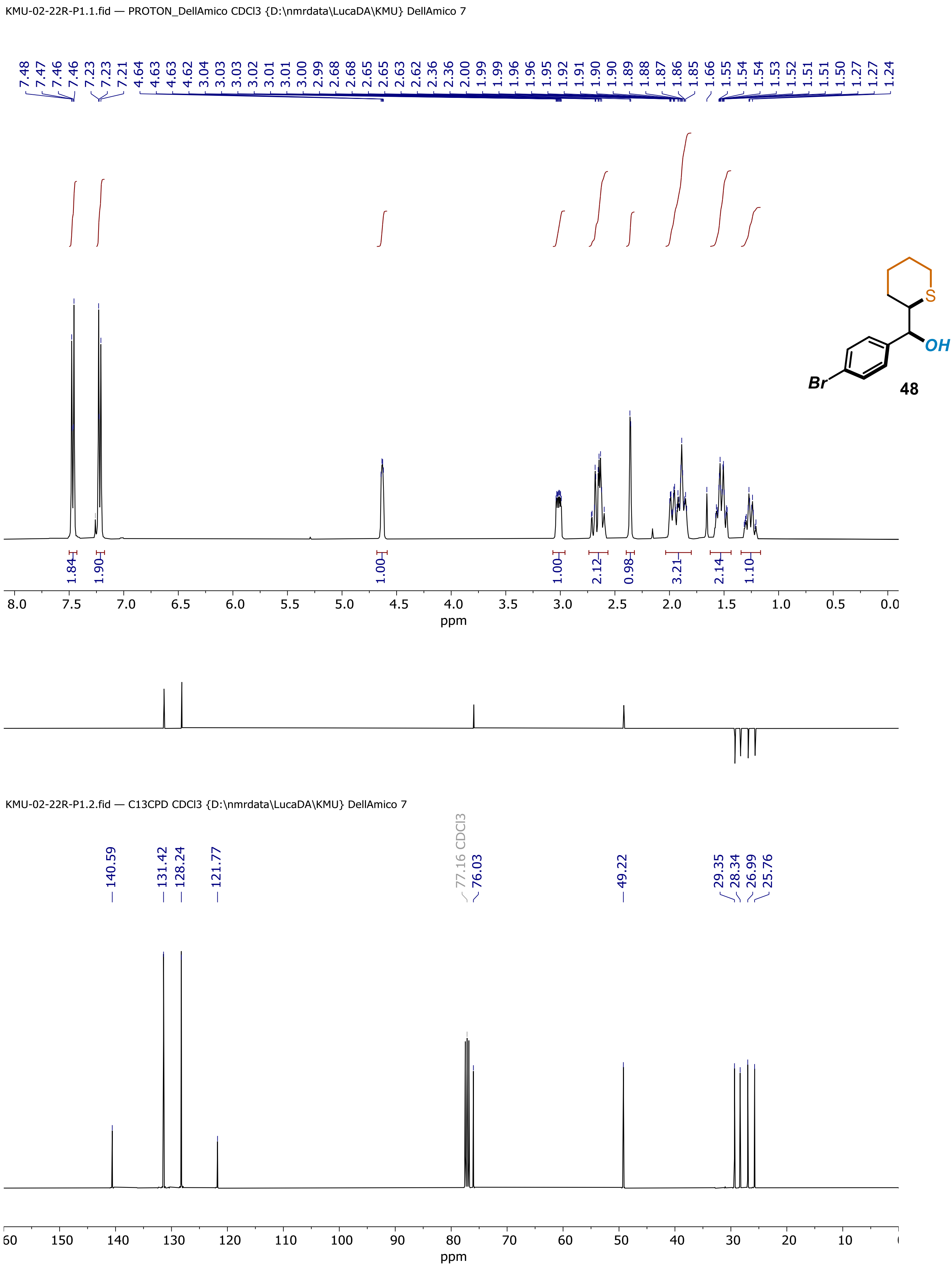

Figure S57. *^1H and ^{13}C NMR spectra of compound **48** in $CDCl_3$.*

4-((*S*)-hydroxy((*R*)-tetrahydro-2*H*-thiopyran-2-yl)methyl)benzonitrile 49

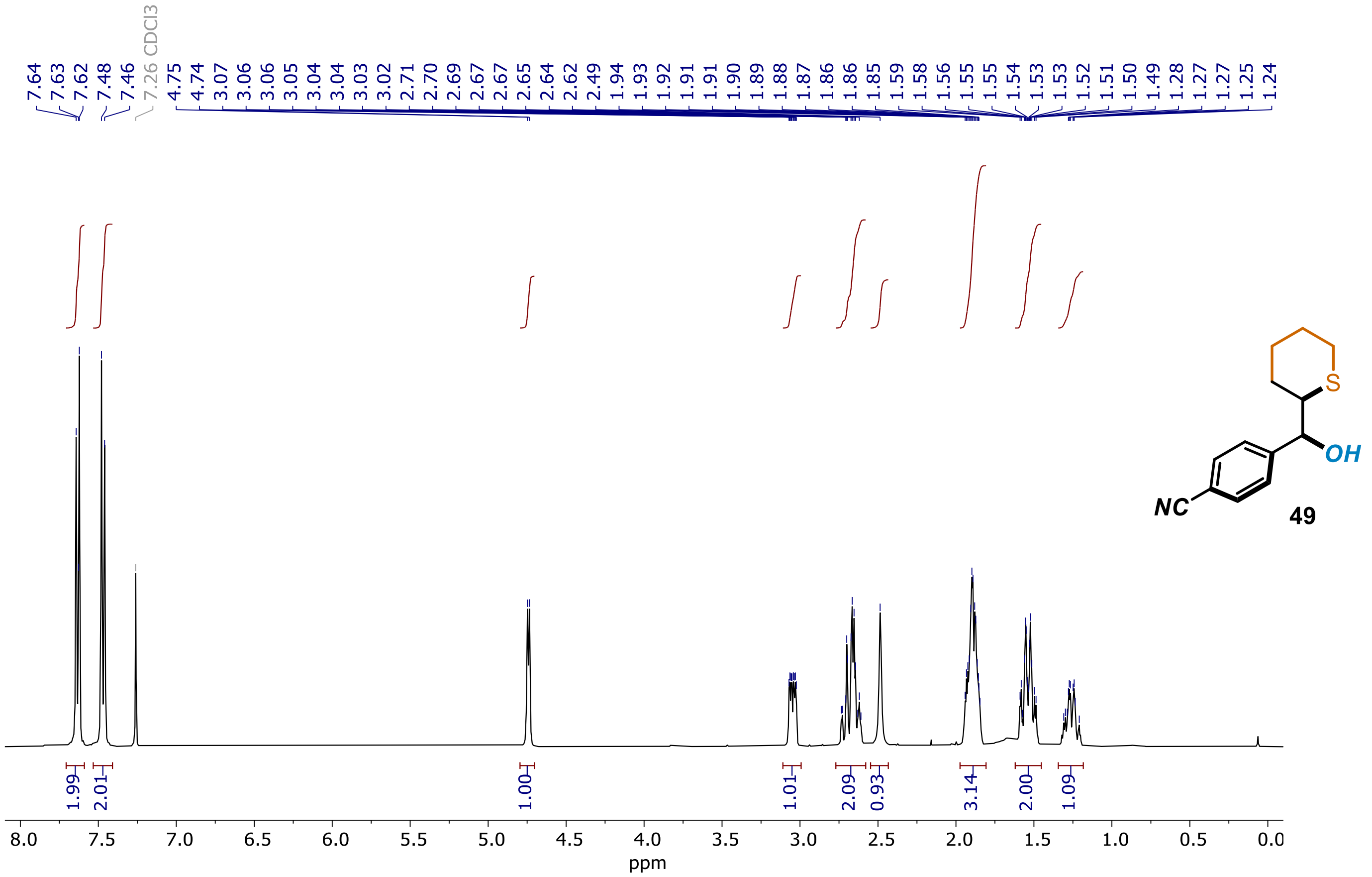

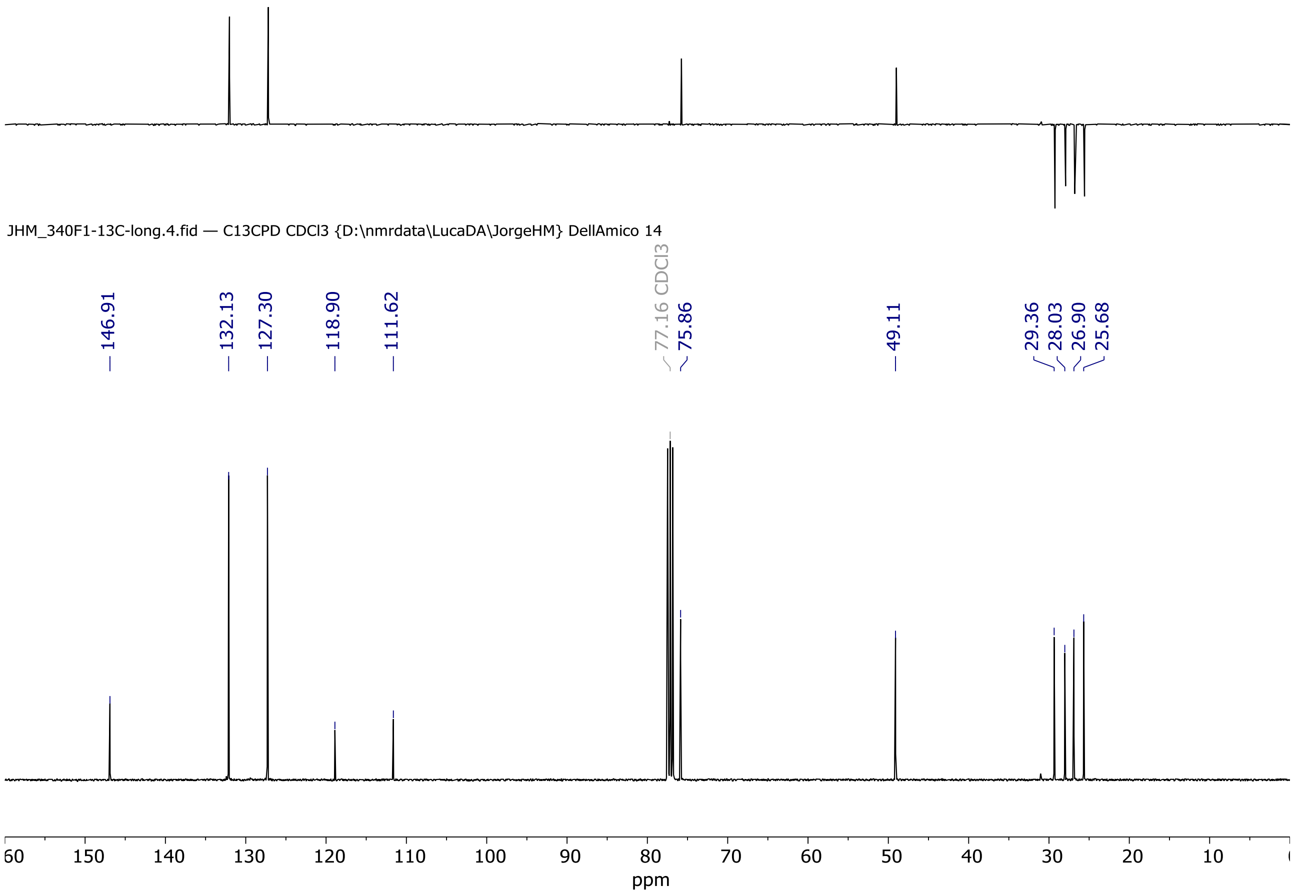

Figure S58. *^{1}H and ^{13}C NMR spectra of compound* ***49*** *in $CDCl_3$.*

(*S*)-[1,1'-biphenyl]-4-yl((*R*)-tetrahydro-2*H*-thiopyran-2-yl)methanol 50

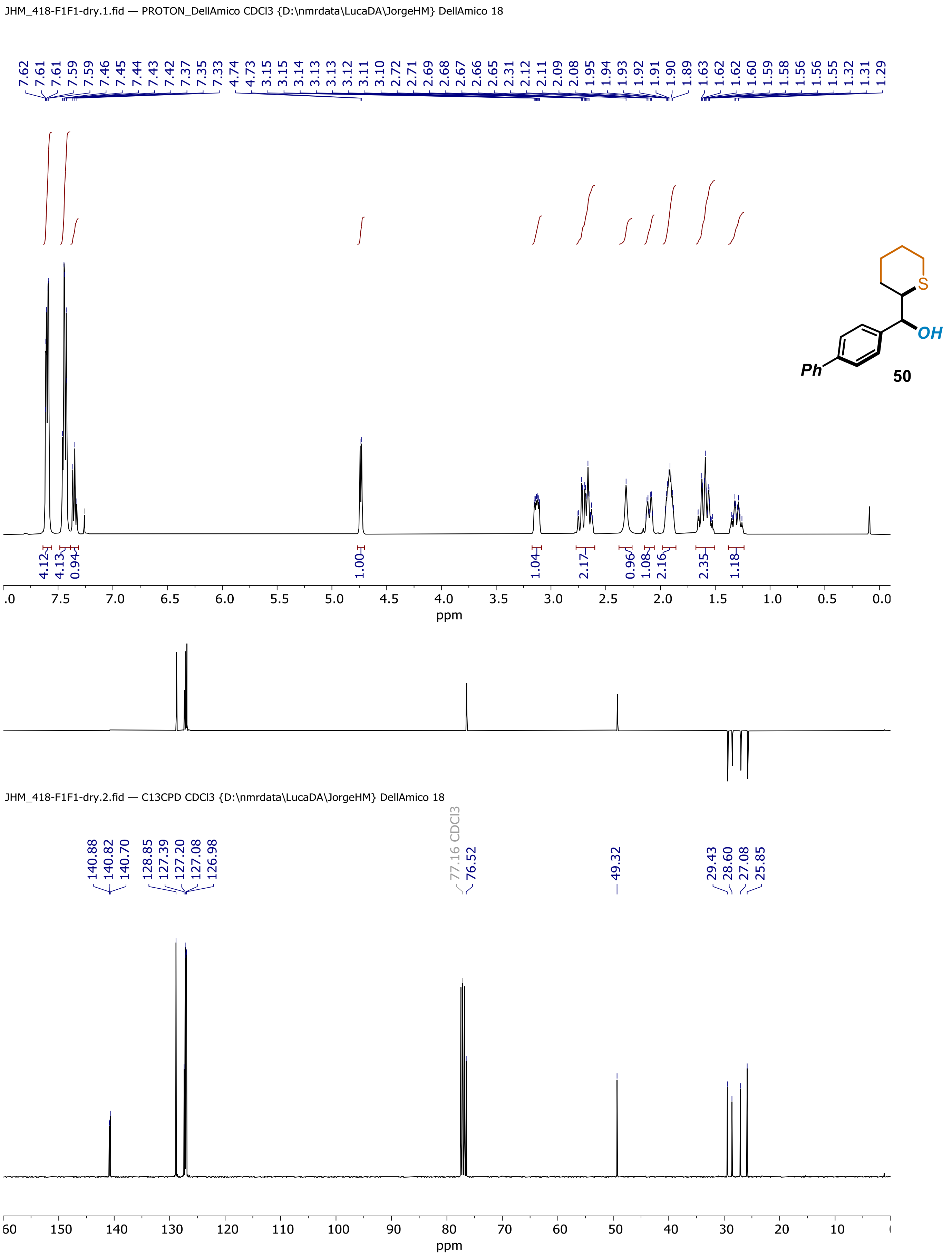

Figure S59. *^{1}H and ^{13}C NMR spectra of compound **50** in $CDCl_3$.*

(*S*)-((*R*)-tetrahydro-2*H*-thiopyran-2-yl)(*p*-tolyl)methanol 51

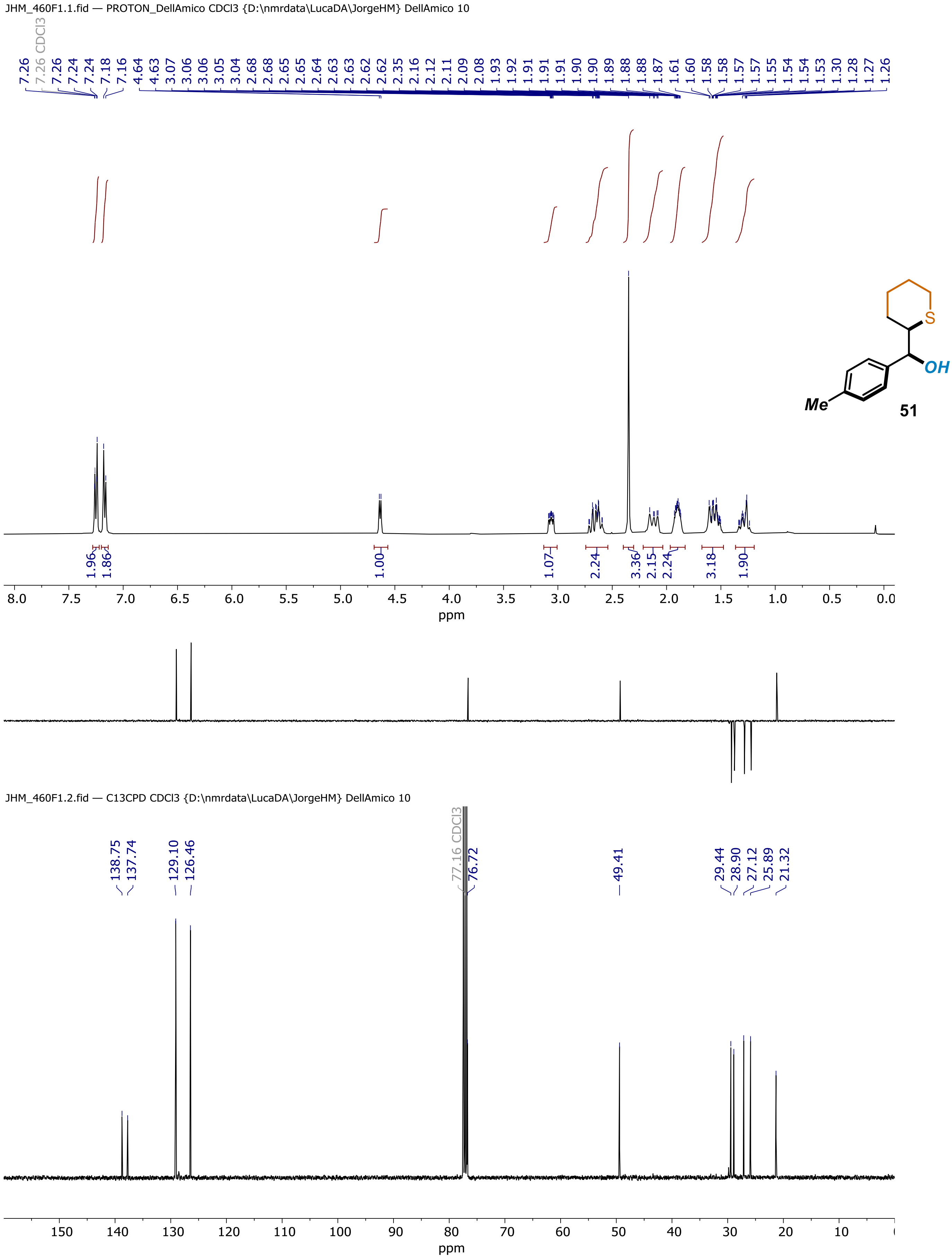

Figure S60. *^1H and ^{13}C NMR spectra of compound **51** in $CDCl_3$.*

(*S*)-((*R*)-tetrahydro-2*H*-thiopyran-2-yl)(*m*-tolyl)methanol 52

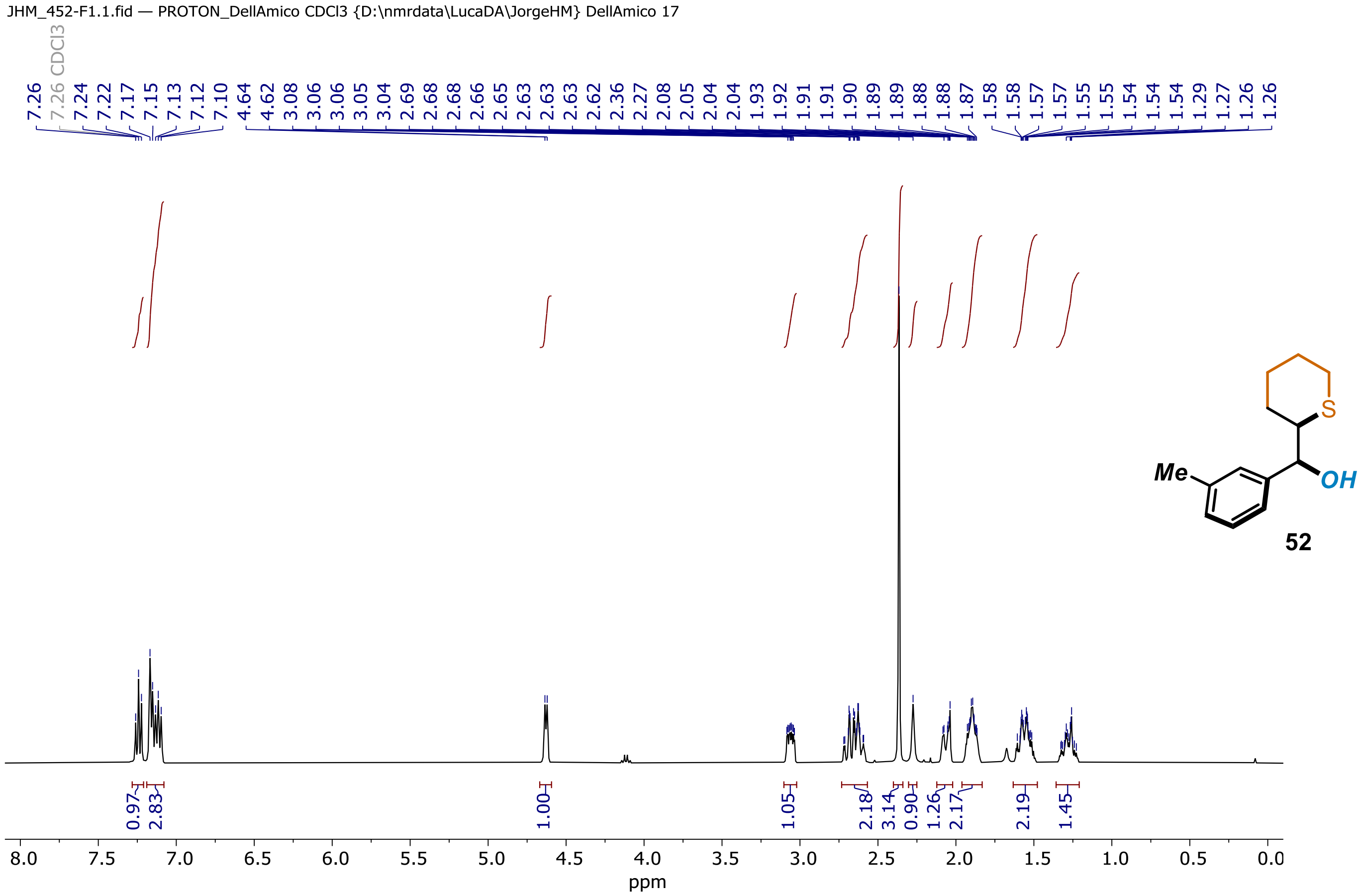

52

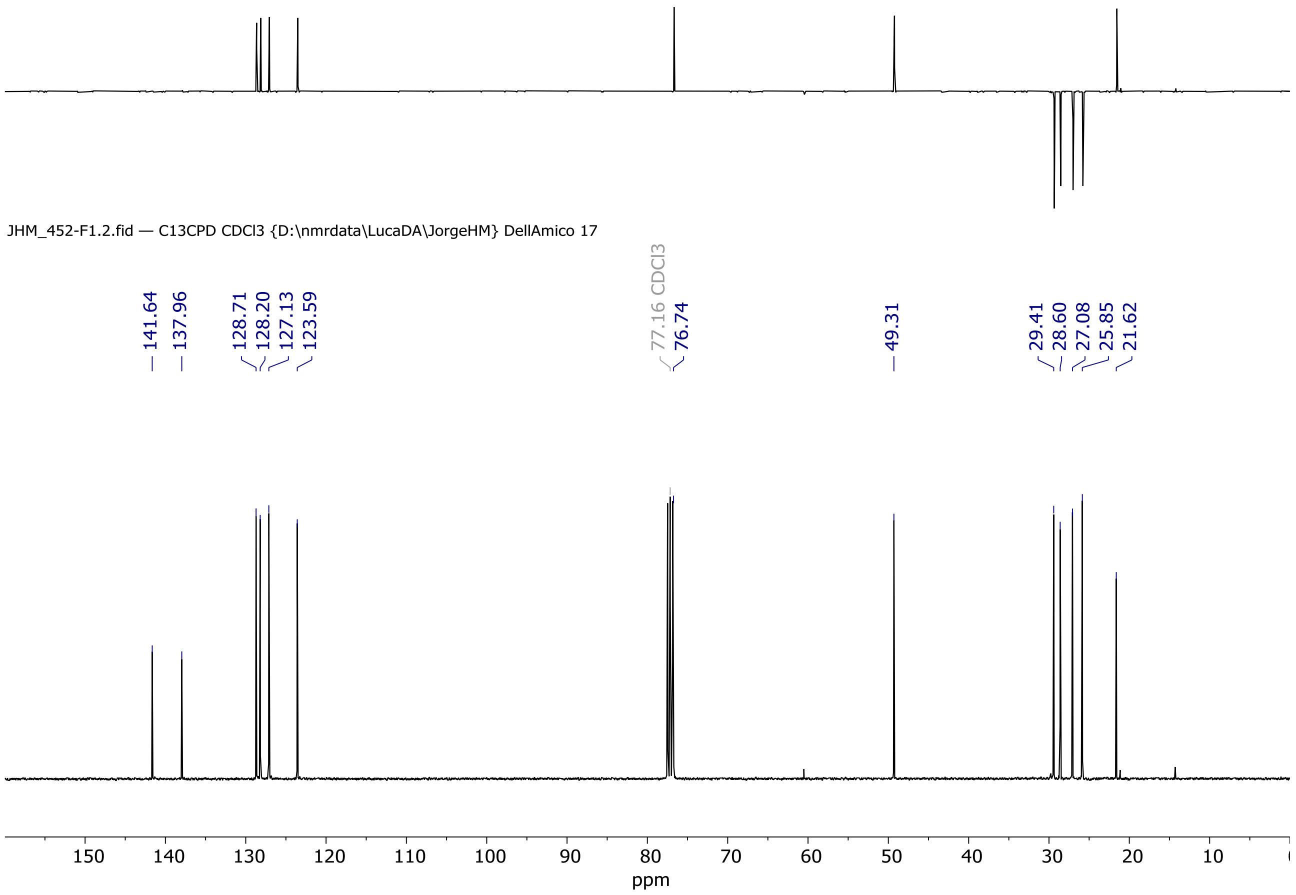

Figure S61. *^1H and ^{13}C NMR spectra of compound **52** in $CDCl_3$.*

(*S*)-((*R*)-tetrahydro-2*H*-thiopyran-2-yl)(*o*-tolyl)methanol 53

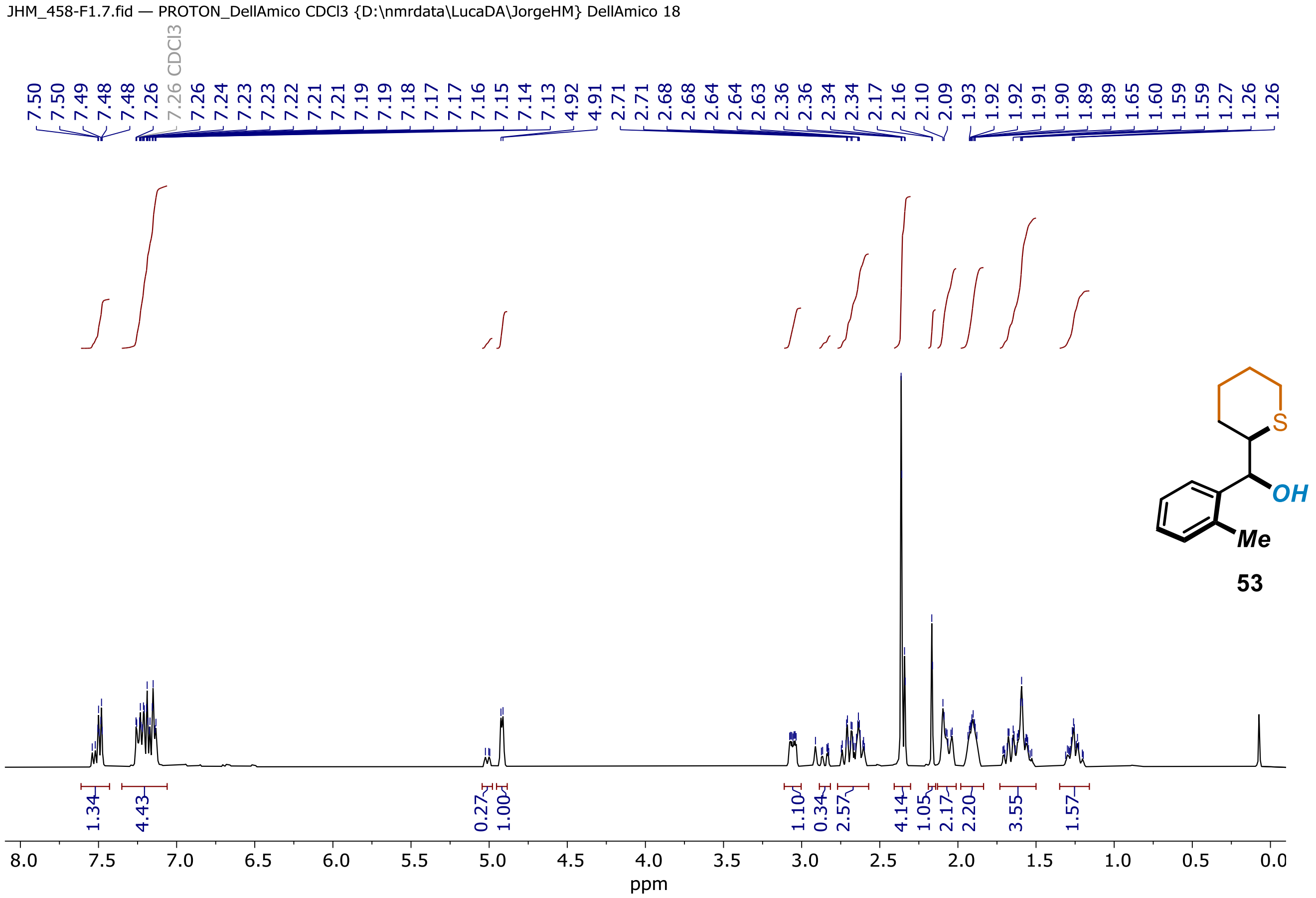

53

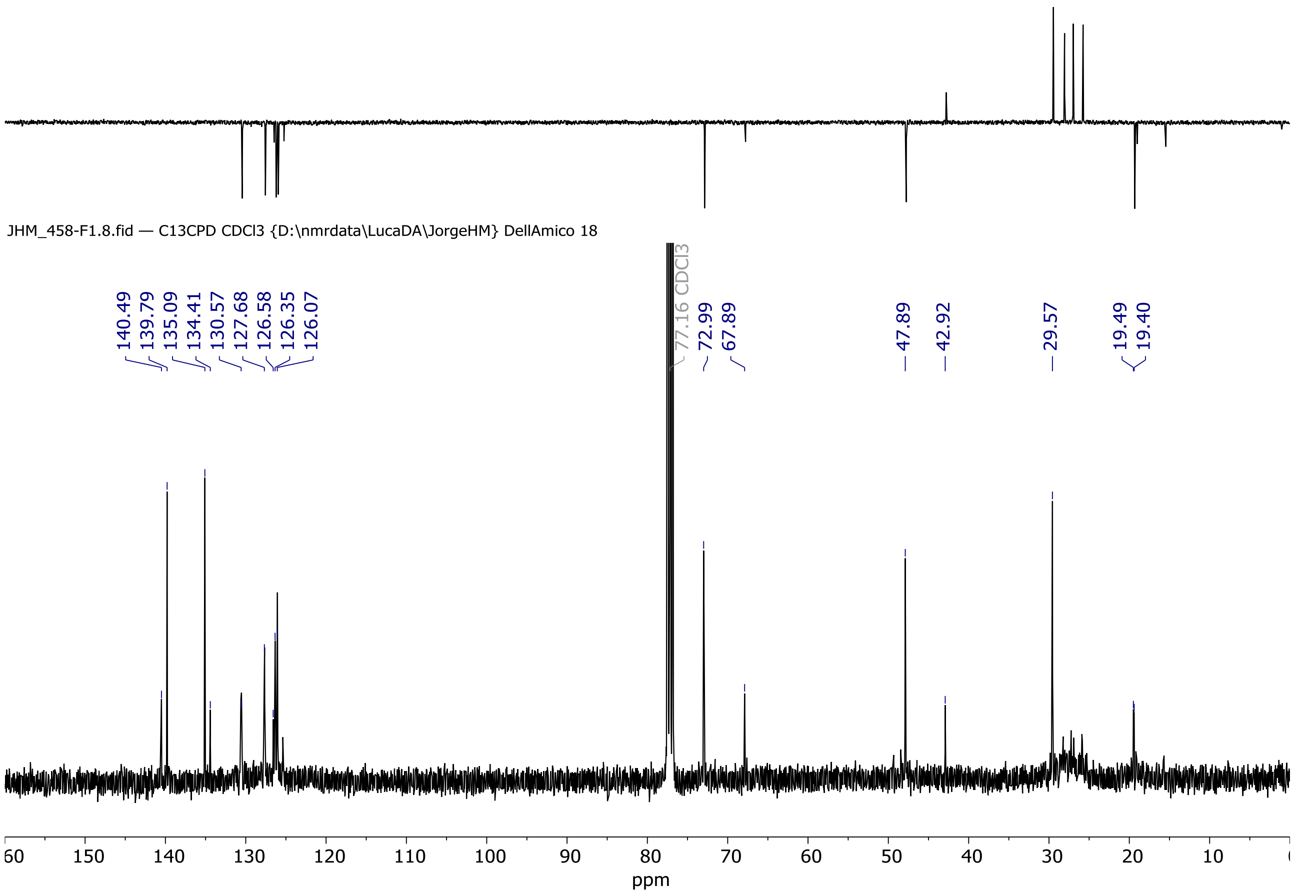

Figure S62. *^{1}H and ^{13}C NMR spectra of compound* ***53*** *in $CDCl_3$.*

diphenyl(tetrahydro-2*H*-thiopyran-2-yl)methanol 54

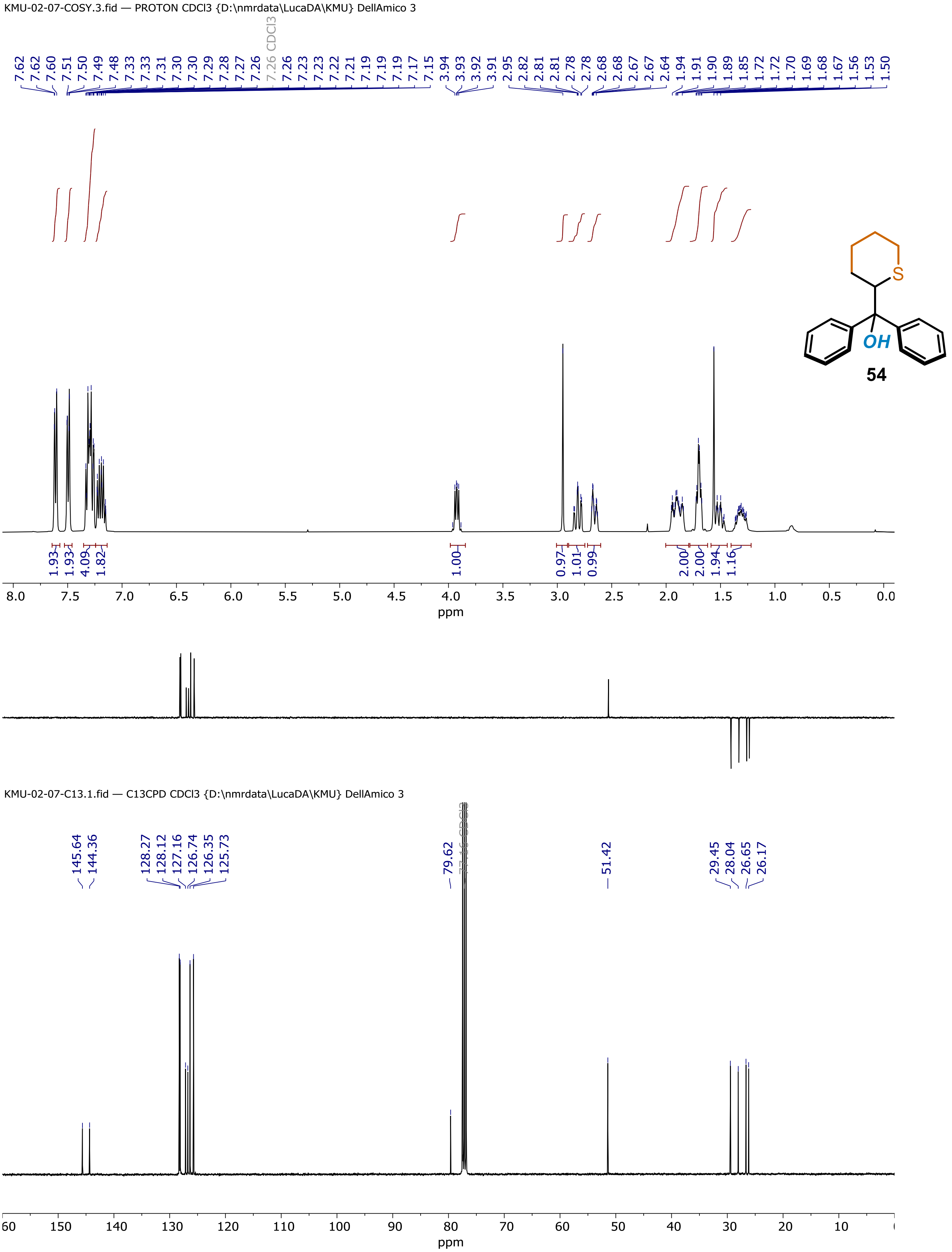

Figure S63. *^1H and ^{13}C NMR spectra of compound **54** in $CDCl_3$.*

(1*S*,2*R*)-3,3',4,4',5',6'-hexahydro-1*H*-spiro[naphthalene-2,2'-thiopyran]-1-ol 55

JHM_420F4F2.1.fid — PROTON_DellAmico CDCl3 {D:\nmrdata\LucaDA\JorgeHM} DellAmico 2

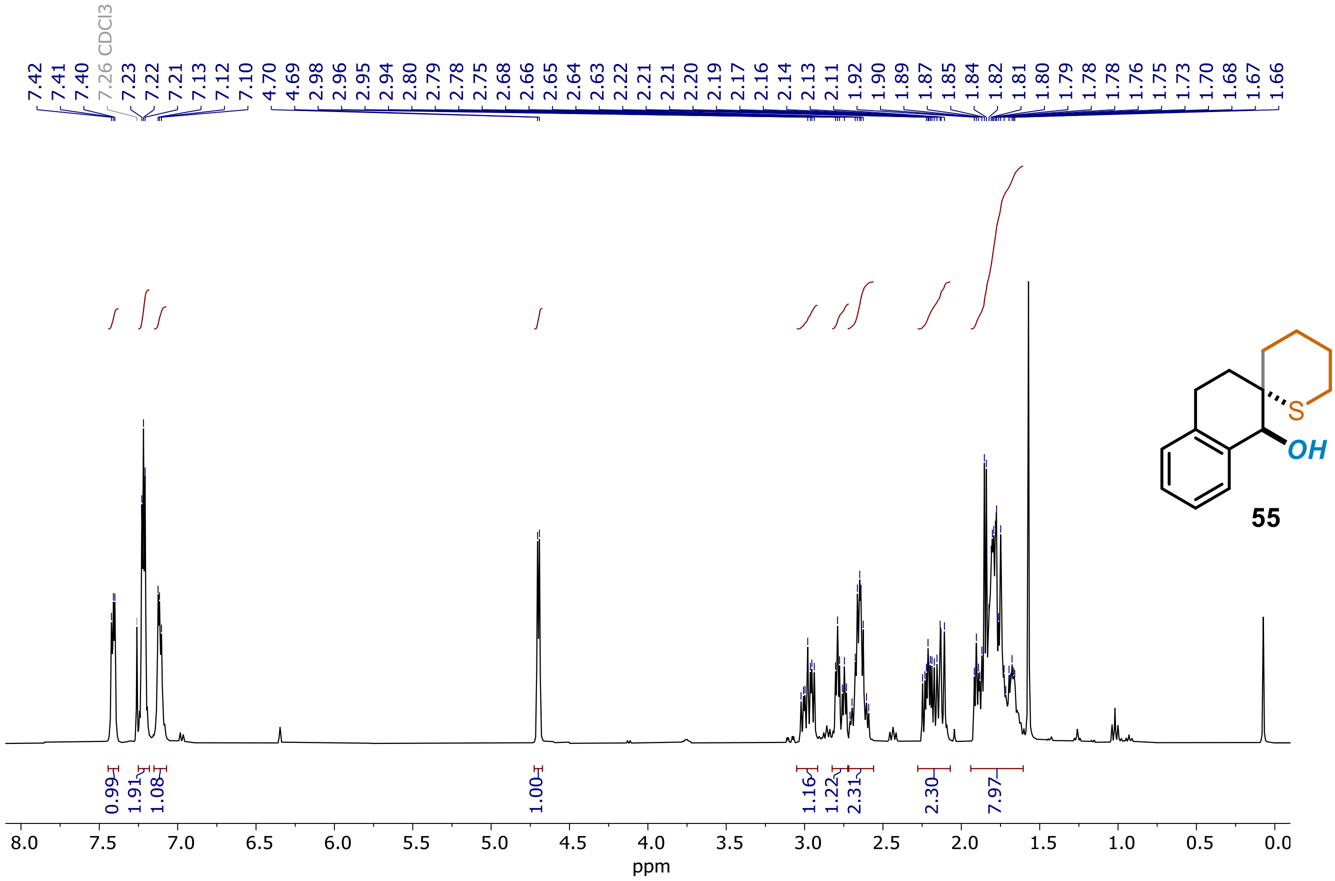

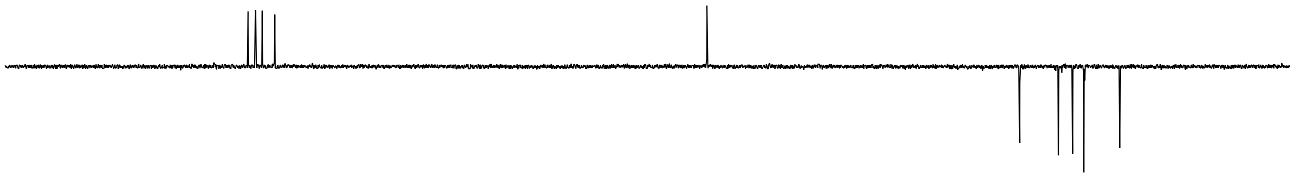

JHM_420F4F2.2.fid — C13CPD CDCl3 {D:\nmrdata\LucaDA\JorgeHM} DellAmico 5

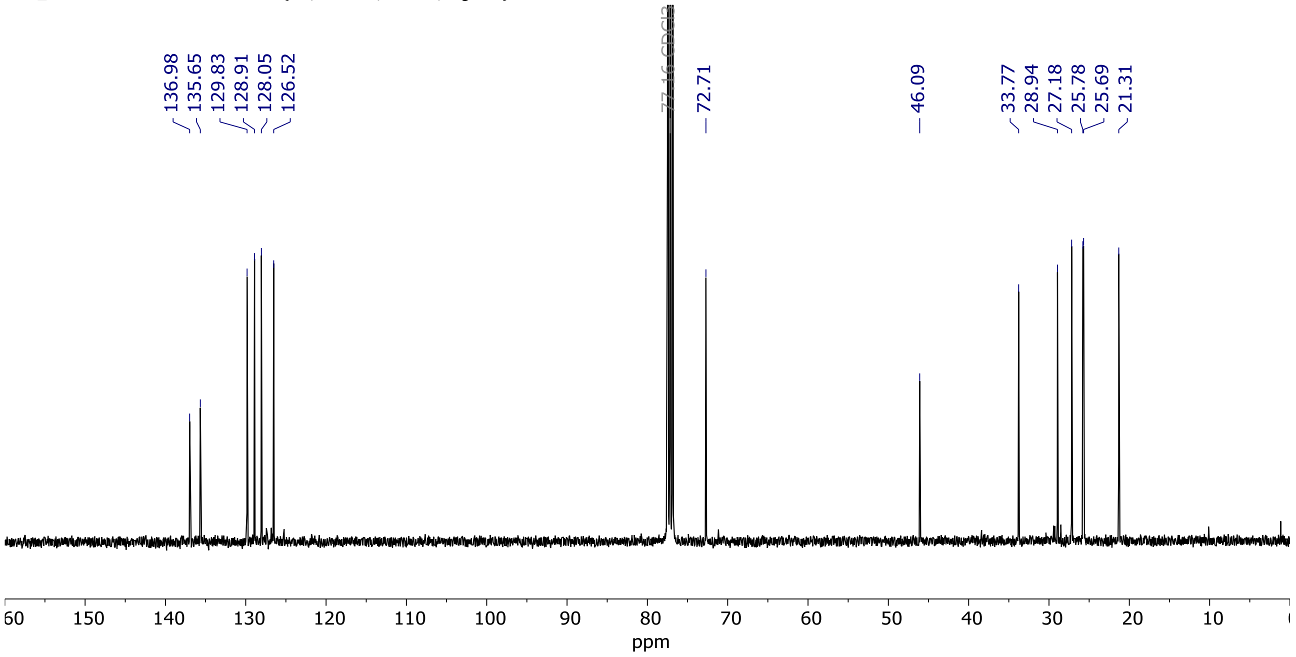

Figure S64. *^{1}H and ^{13}C NMR spectra of compound **55** in $CDCl_3$.*

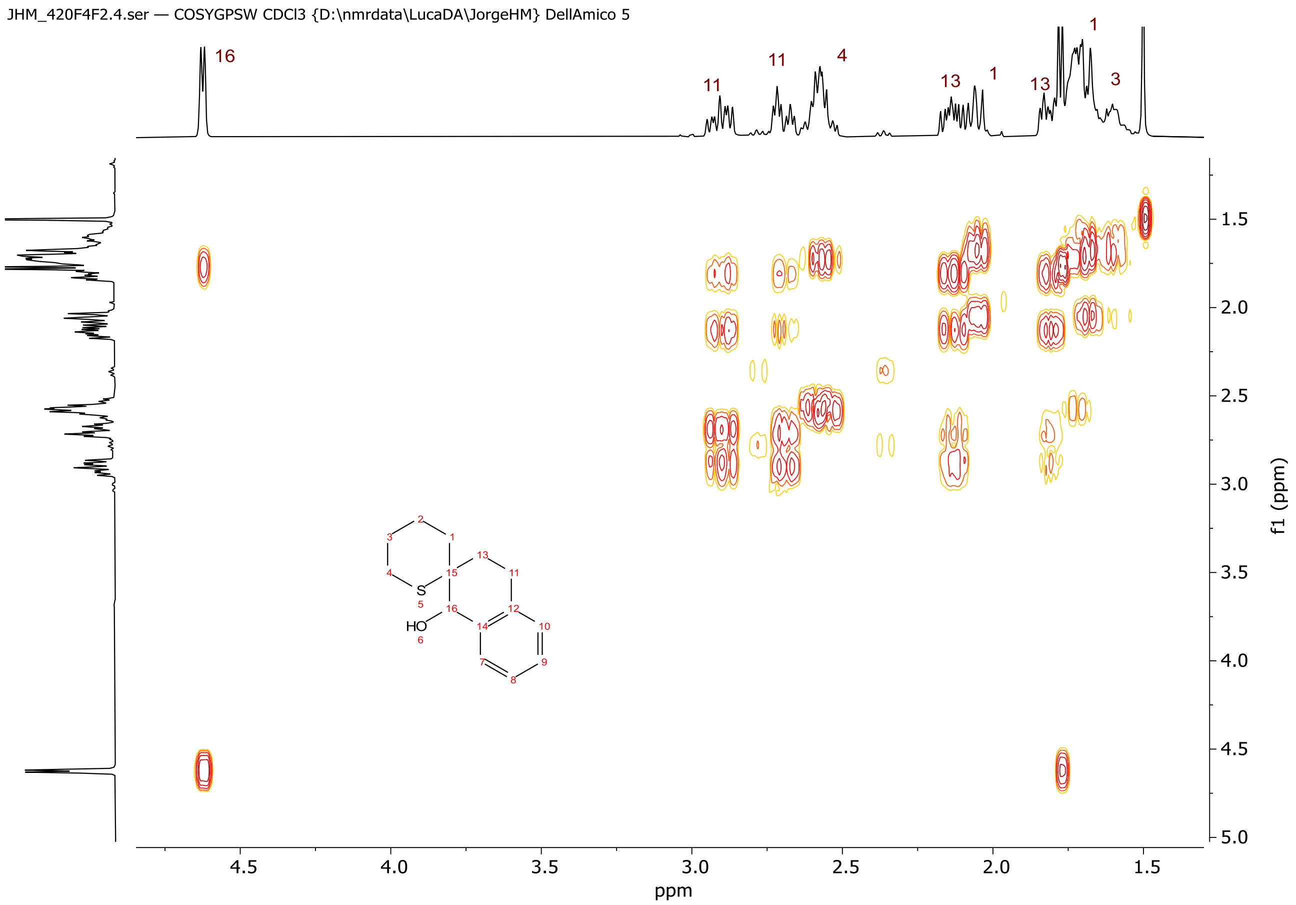

Figure S65. *COSY NMR spectrum of compound **55** in CDCl$_3$.*

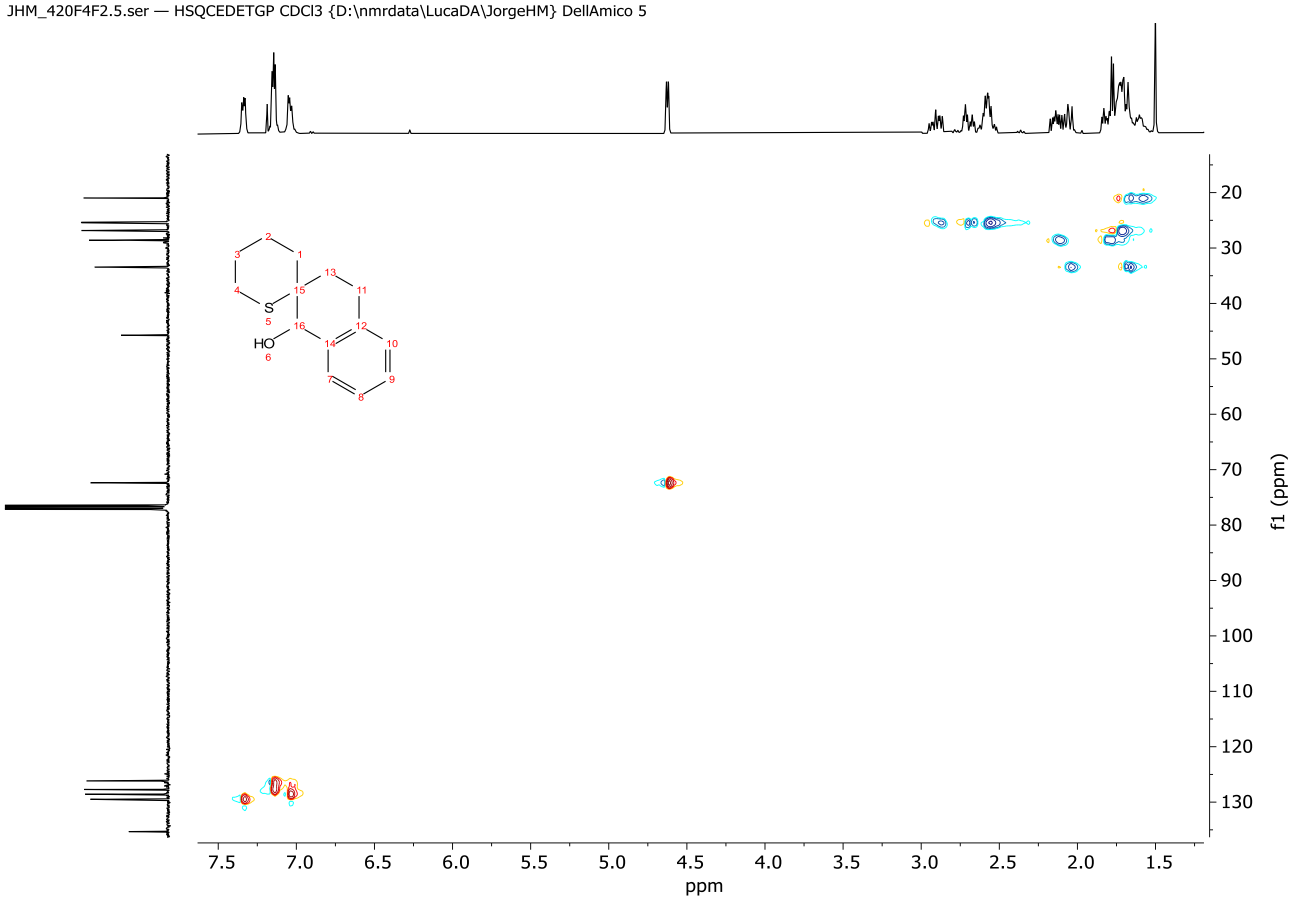

Figure S66. *HSQC NMR spectrum of compound **55** in CDCl$_3$.*

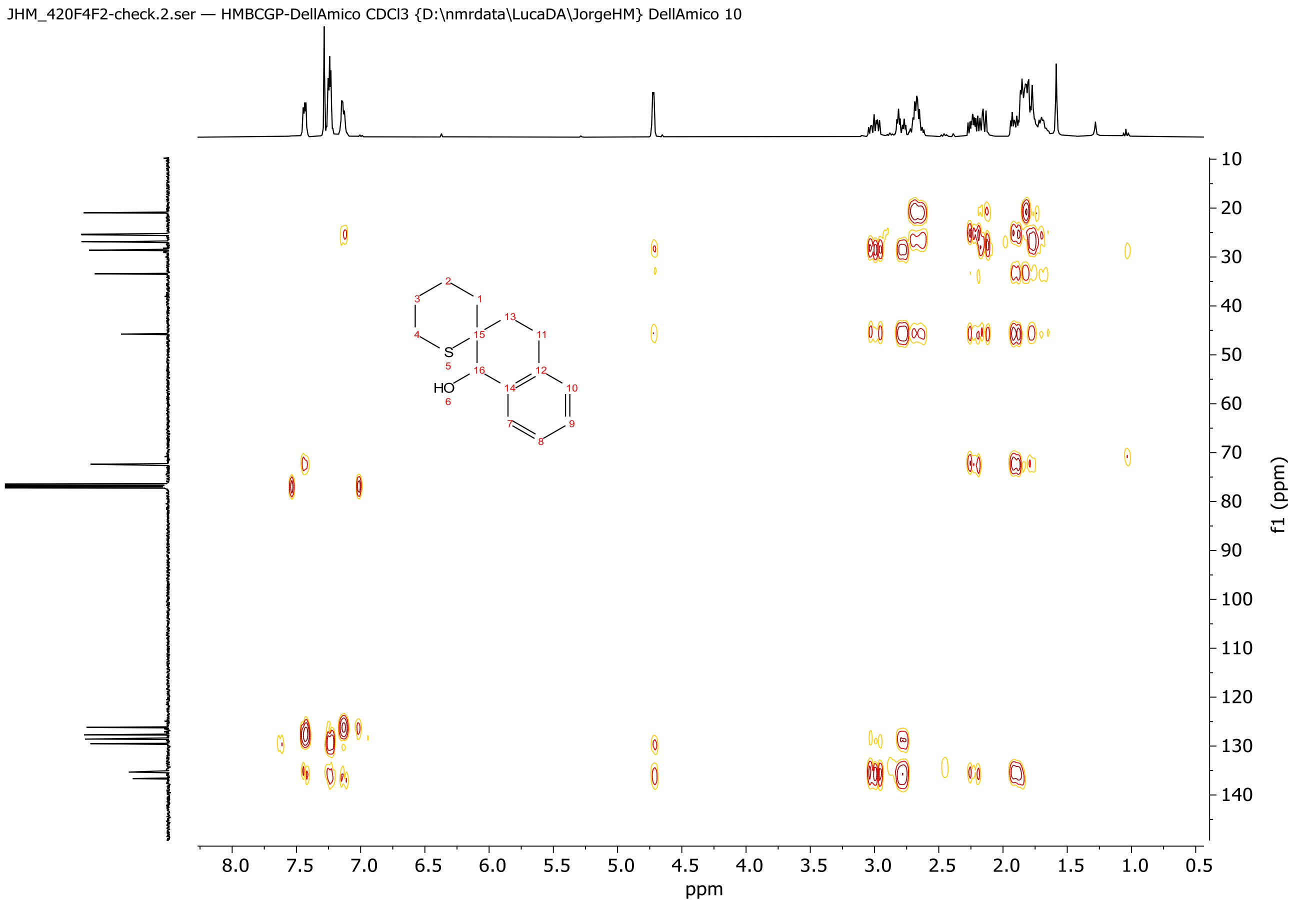

Figure S67. *HMBC NMR spectrum of compound* ***55*** *in* $CDCl_3$.

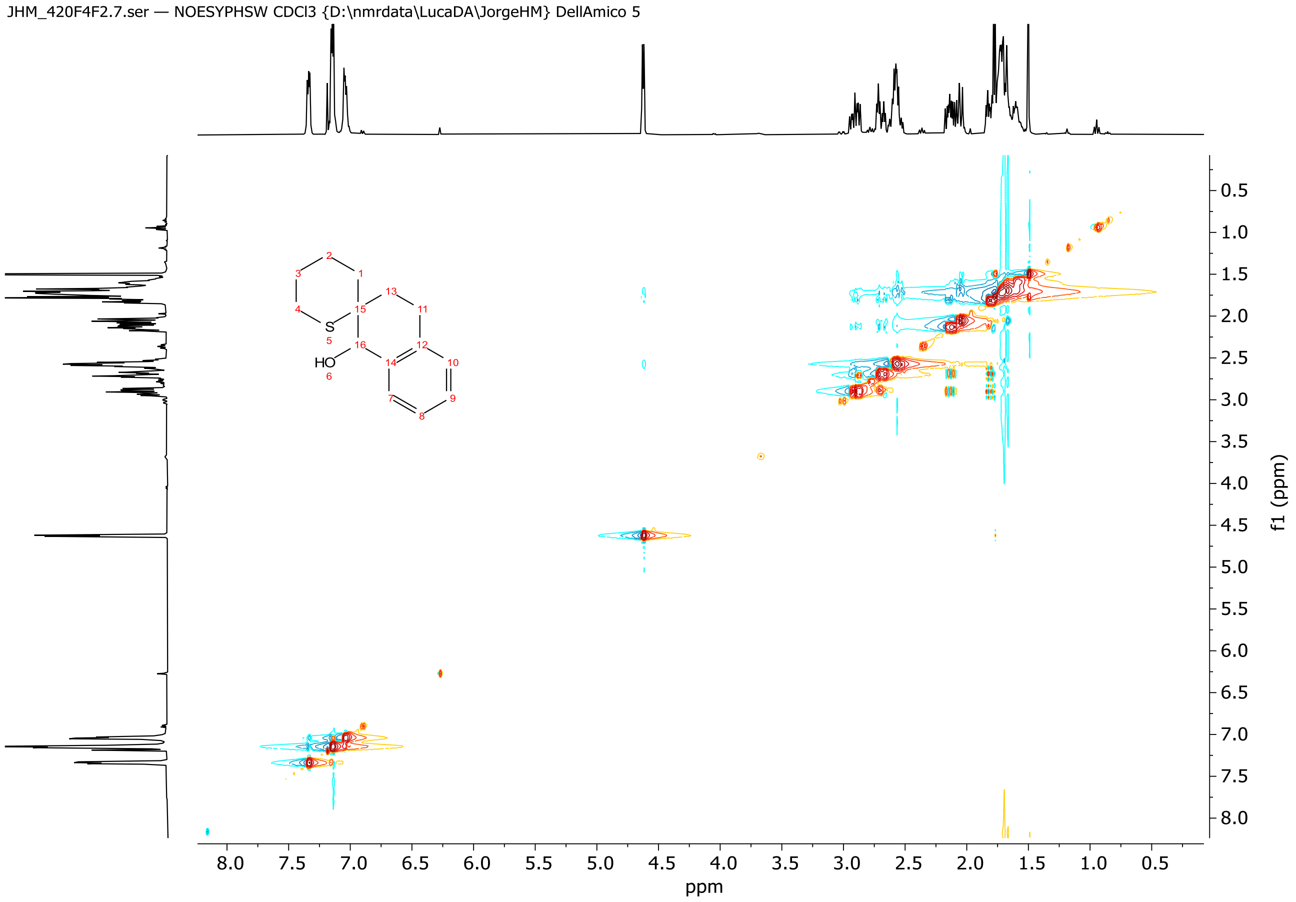

Figure S68. *NOESY NMR spectrum of compound* ***55*** *in* $CDCl_3$.

(*S*)-(2-fluorophenyl)((*R*)-tetrahydro-2*H*-thiopyran-2-yl)methanol 56

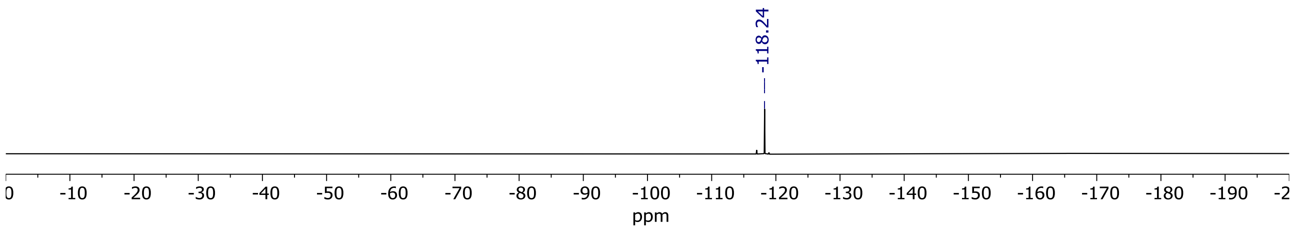

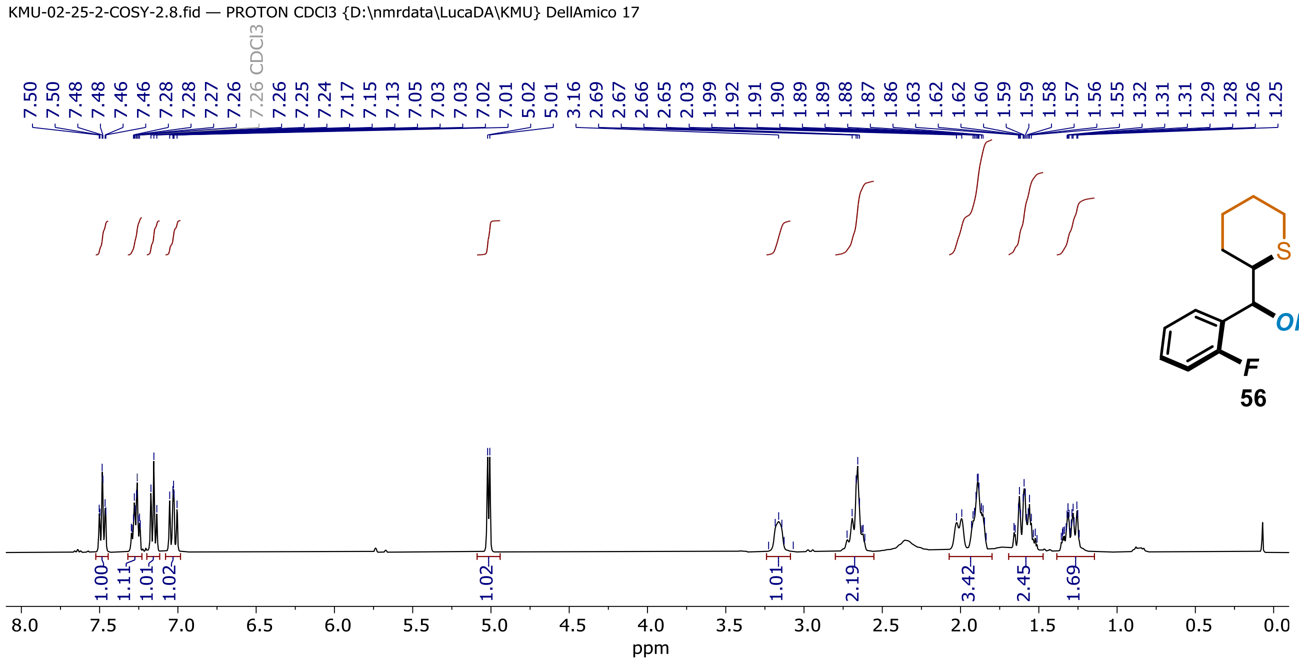

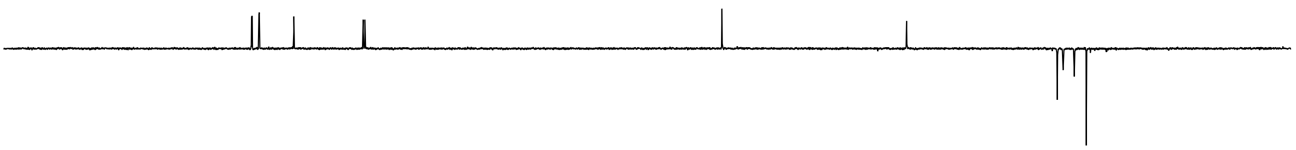

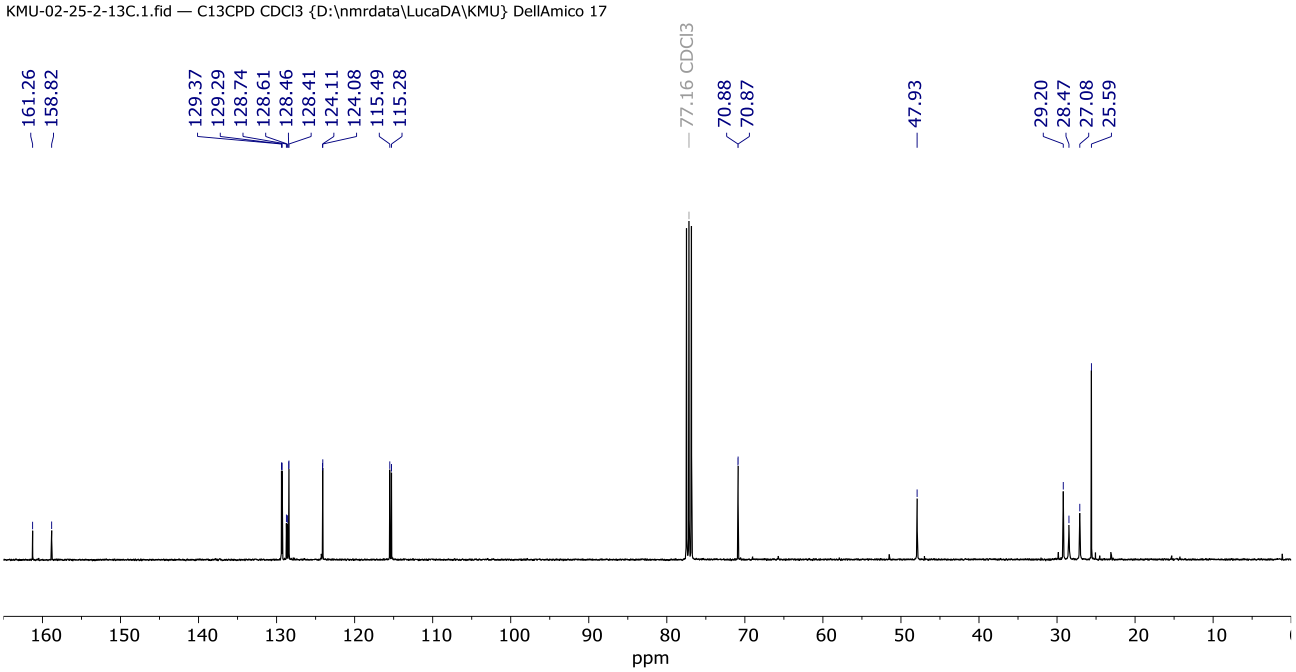

Figure S69. *^{1}H and ^{13}C NMR spectra of compound* ***56*** *in $CDCl_3$.*

2-(methoxy(phenyl)methyl)tetrahydro-2*H*-thiopyran 57

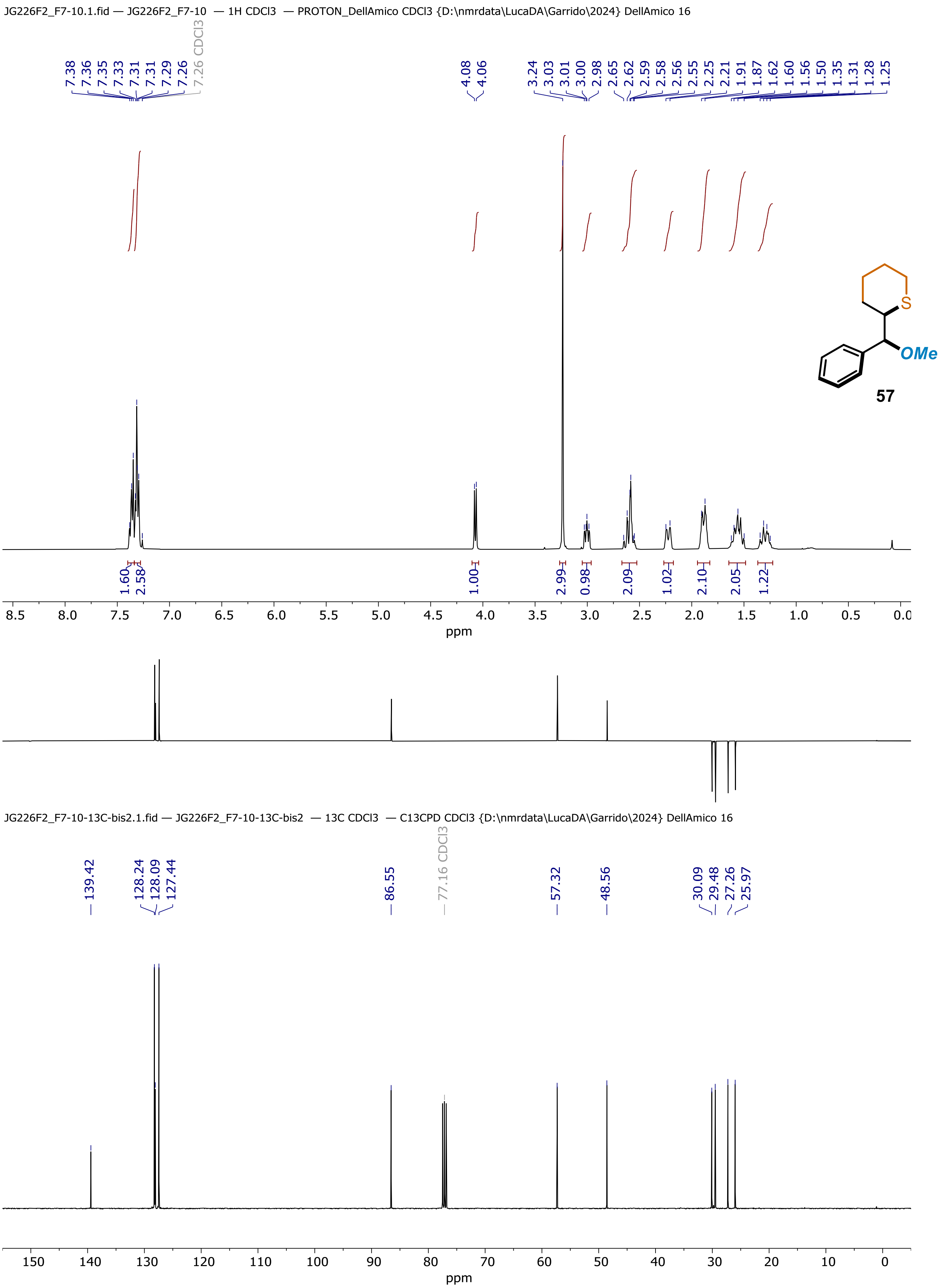

Figure S70. *^1H and ^{13}C NMR spectra of compound **57** in $CDCl_3$.*

(*R*)-2-((*S*)-(cyclohexyloxy)(phenyl)methyl)tetrahydro-2*H*-thiopyran 58

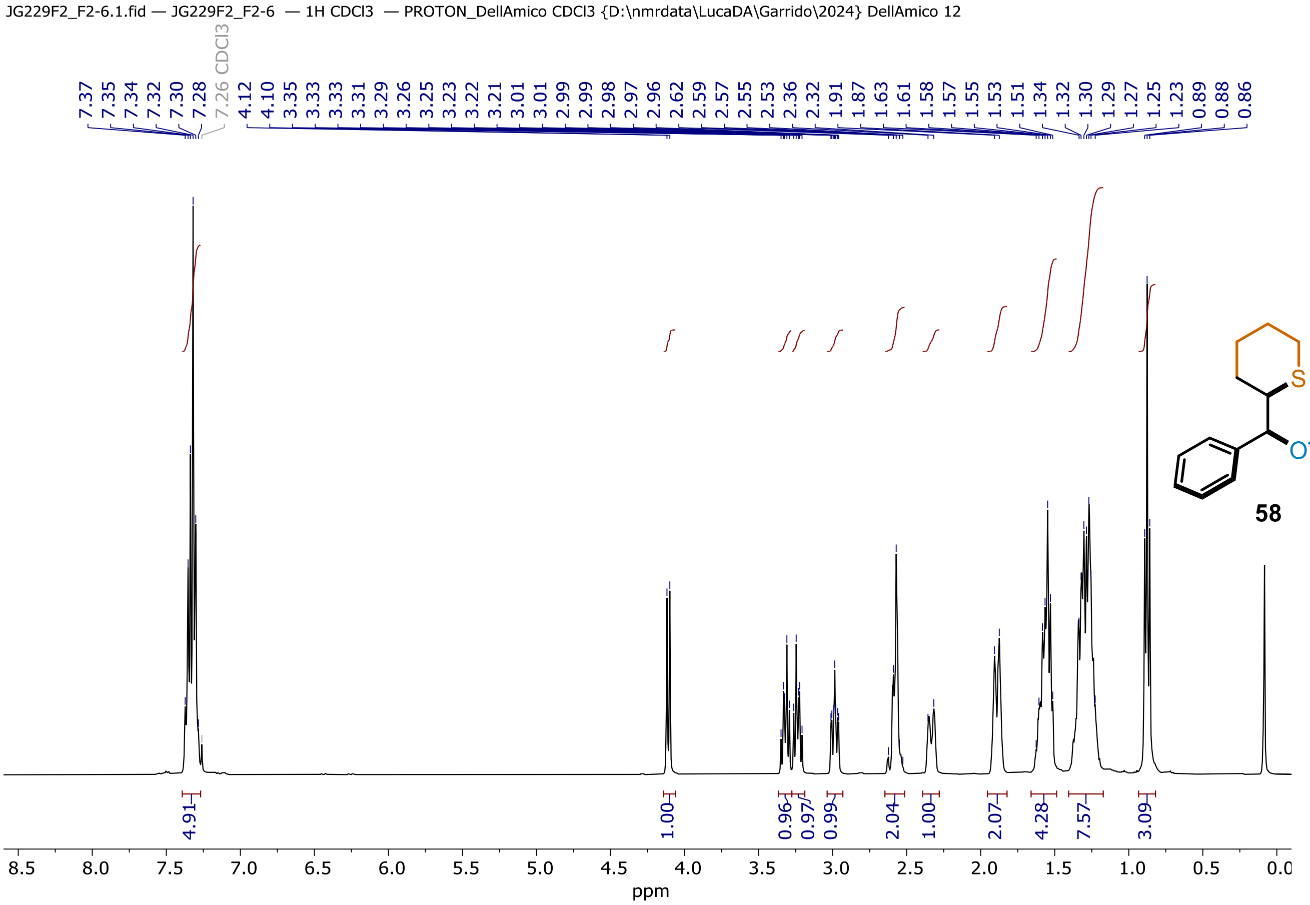

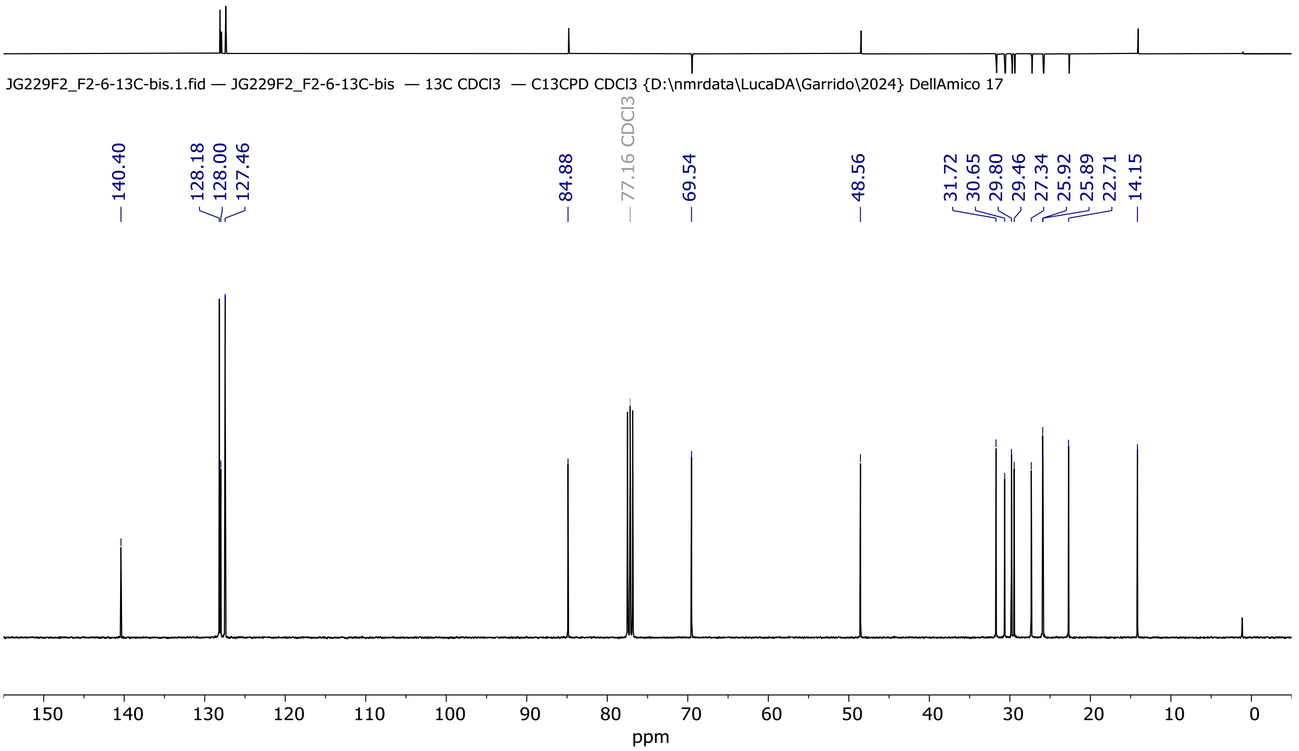

Figure S71. ^{1}H and ^{13}C NMR spectra of compound **58** in $CDCl_3$.

4-methyl-*N*-((*S*)-phenyl((*R*)-tetrahydro-2*H*-thiopyran-2-yl)methyl)aniline 59

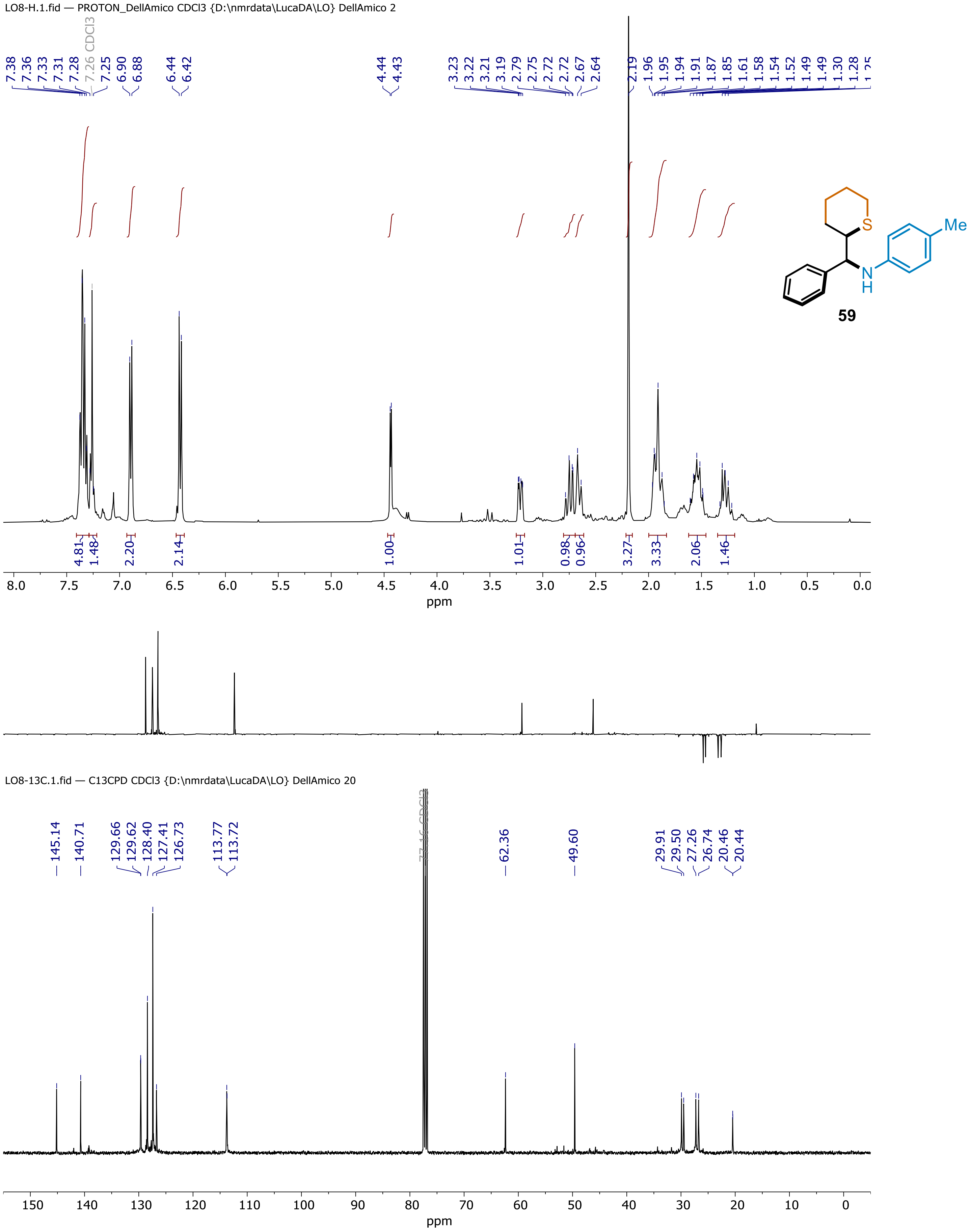

Figure S72. *^{1}H and ^{13}C NMR spectra of compound **59** in $CDCl_3$.*

(*R*)-2-((*S*)-chloro(phenyl)methyl)tetrahydro-2*H*-thiopyran 60

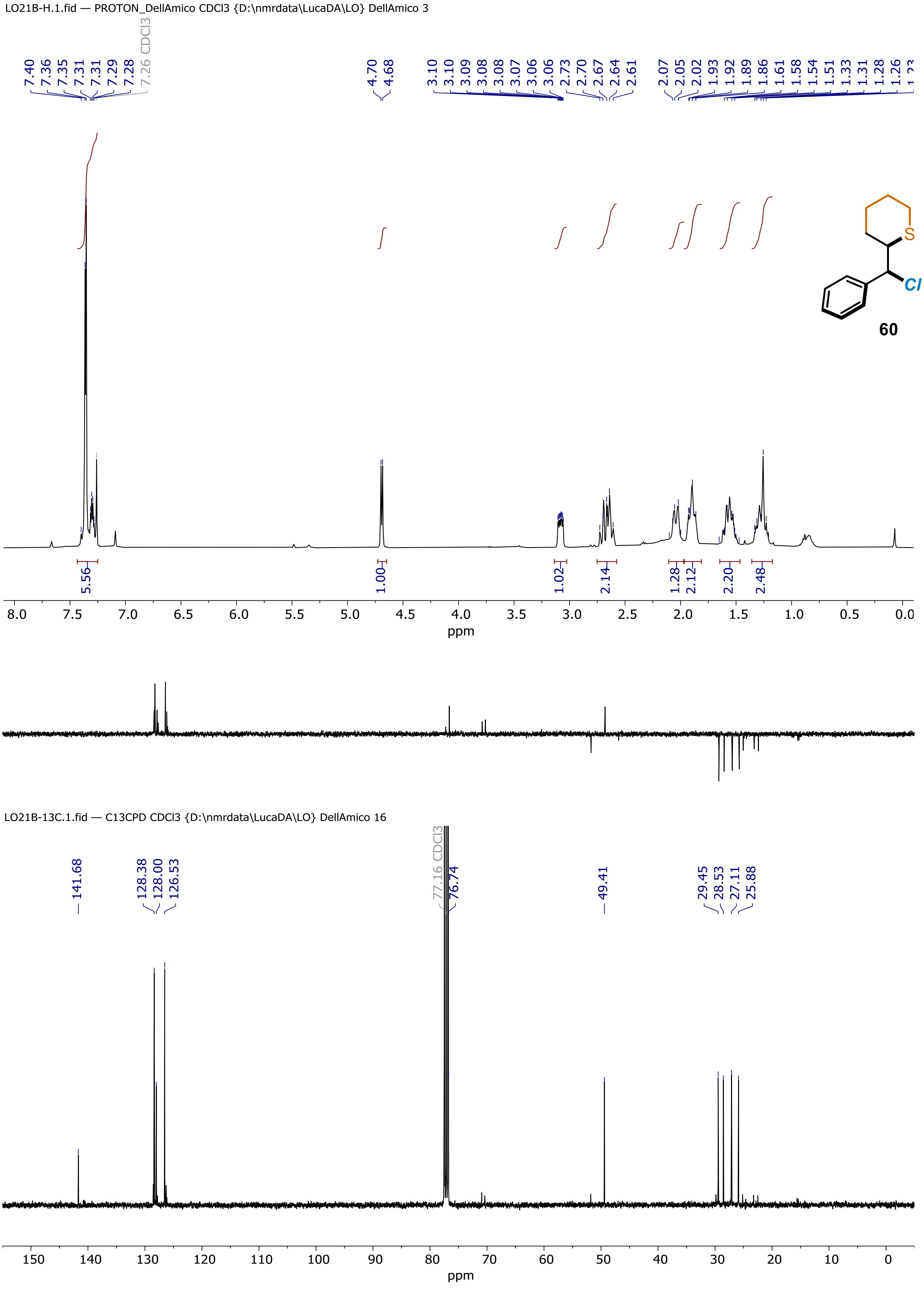

Figure S73. *^1H and ^{13}C NMR spectra of compound* ***60*** *in $CDCl_3$.*

(*R*)-2-((*S*)-bromo(phenyl)methyl)tetrahydro-2*H*-thiopyran 61

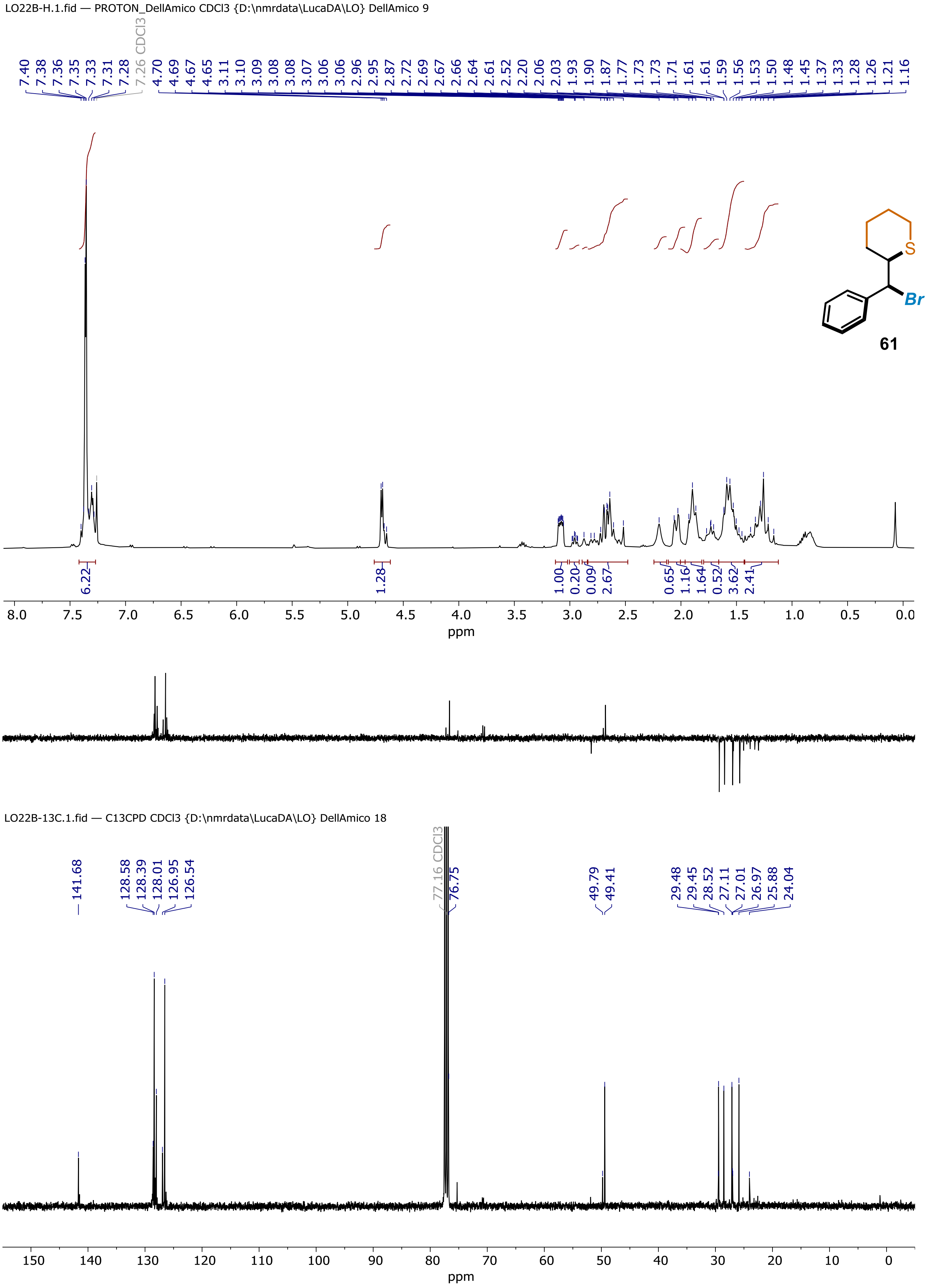

Figure S74. *^{1}H and ^{13}C NMR spectra of compound **61** in $CDCl_3$.*

(R)-2-((S)-3-methyl-1-phenylbut-3-en-1-yl)tetrahydro-2H-thiopyran 62

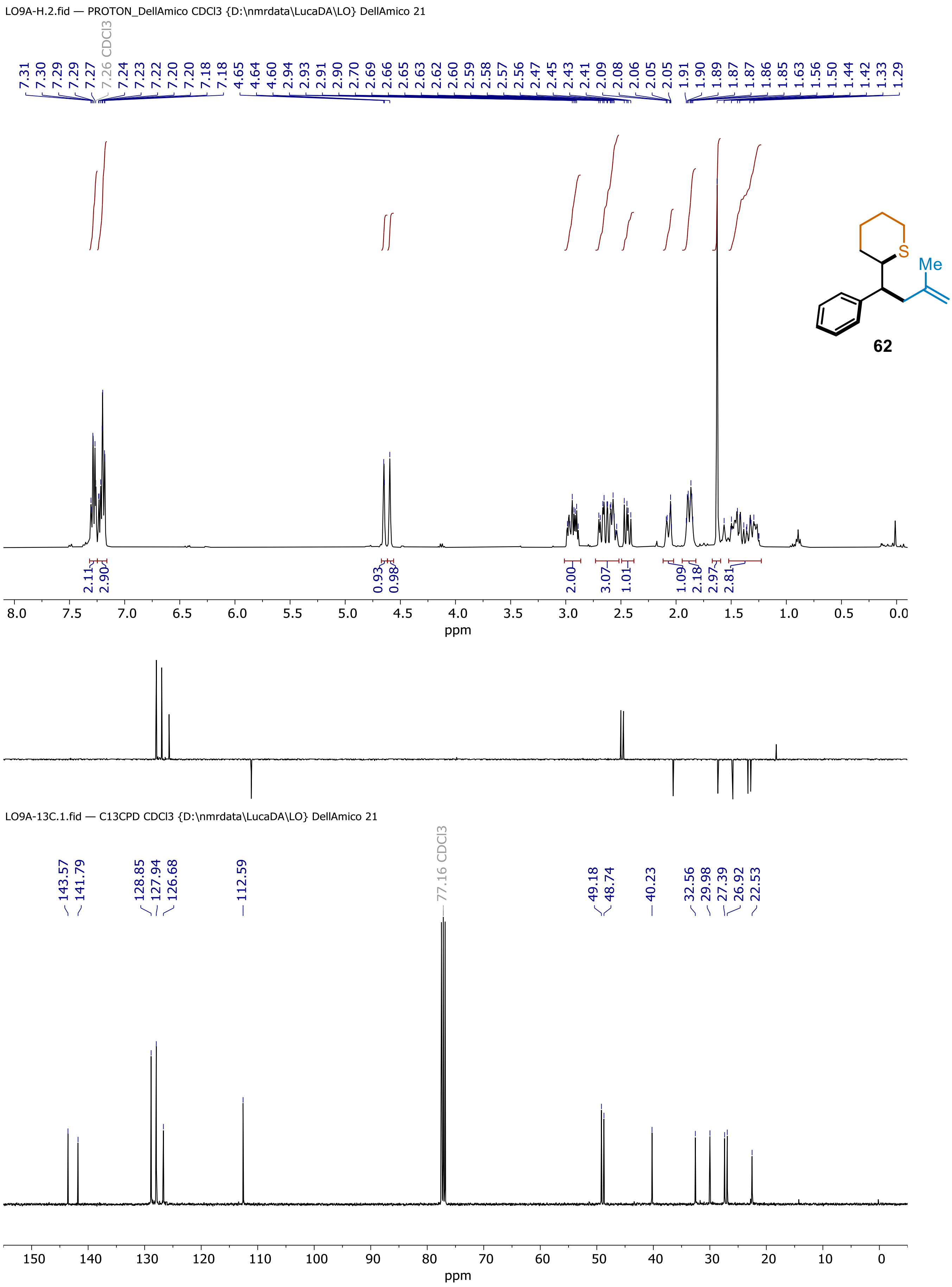

Figure S75. *^{1}H and ^{13}C NMR spectra of compound **62** in $CDCl_3$.*

(*S*)-2-((*S*)-phenyl((*R*)-tetrahydro-2*H*-thiopyran-2-yl)methyl)cyclopentan-1-one 63

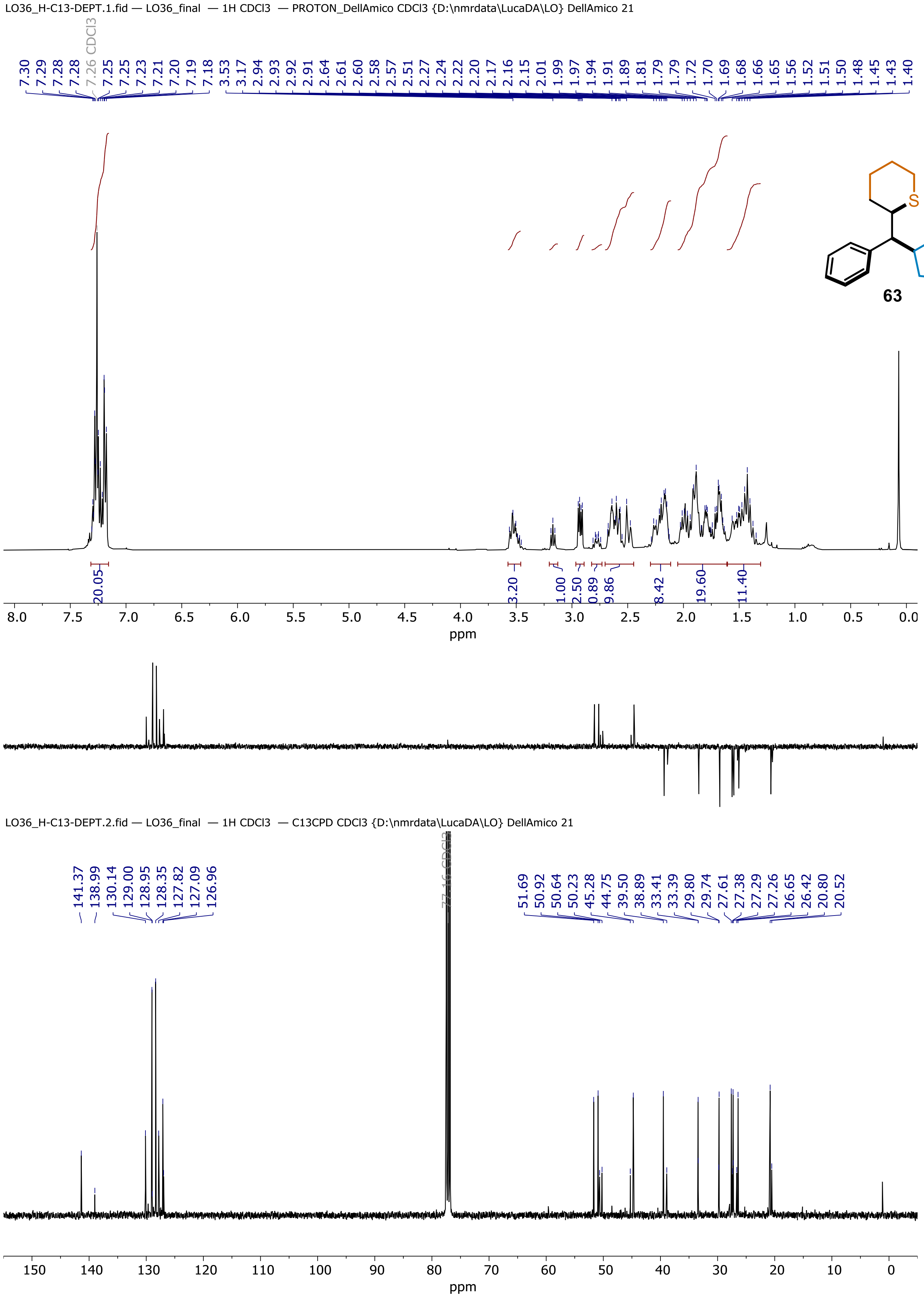

Figure S76. *^{1}H and ^{13}C NMR spectra of compound* ***63*** *in $CDCl_3$.*

(1*R*,2*R*)-2-((*S*)-hydroxy(phenyl)methyl)tetrahydro-2*H*-thiopyran 1-oxide 64

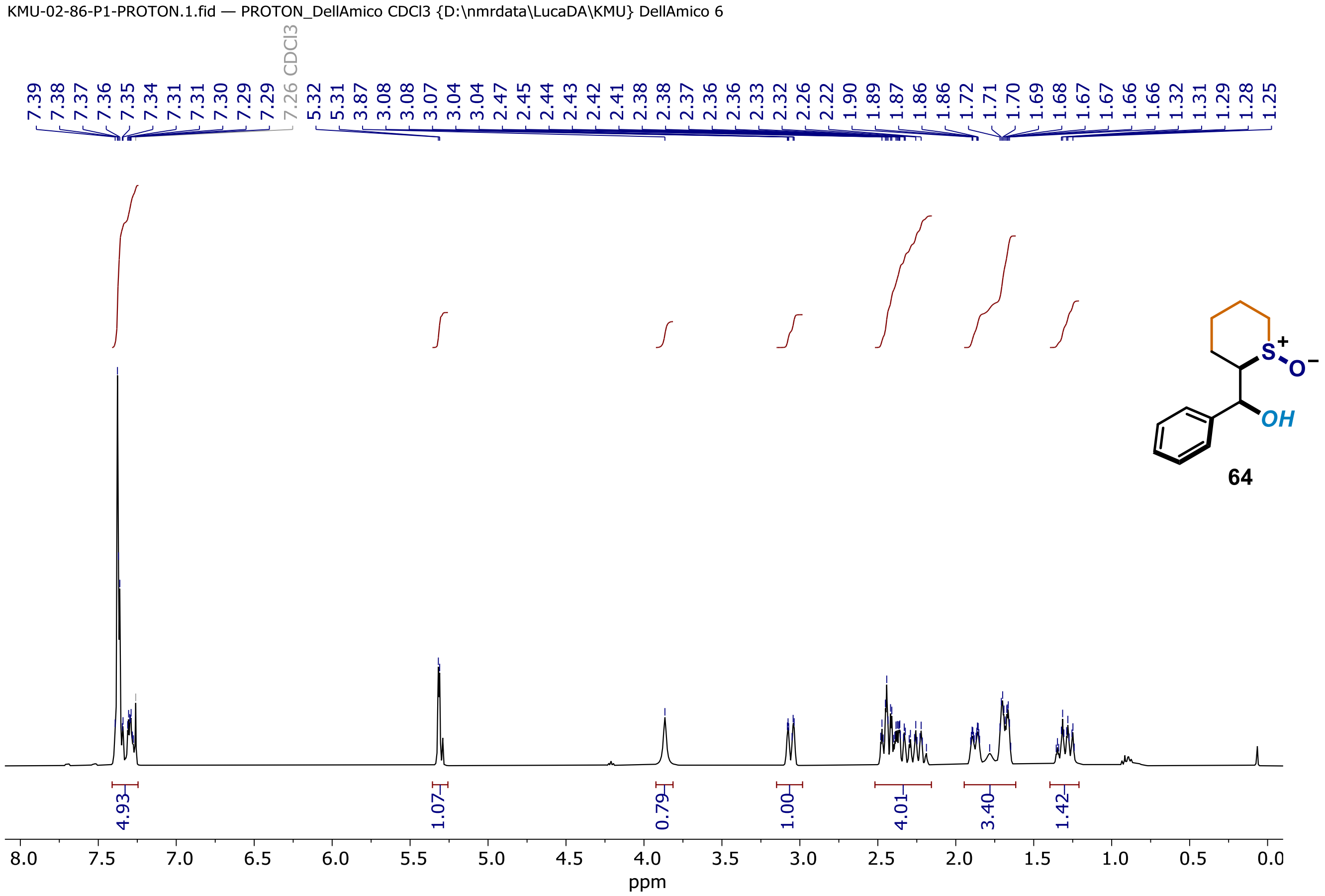

64

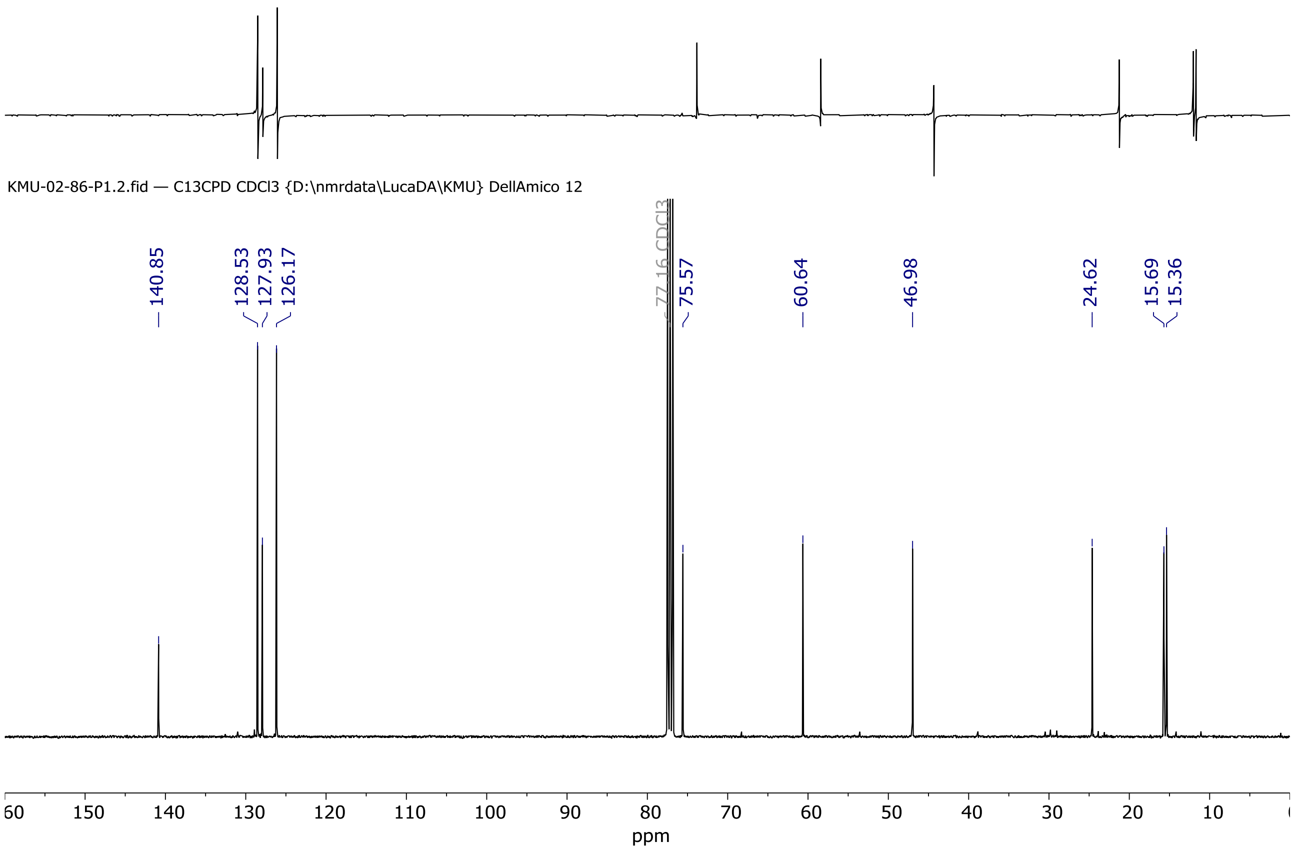

Figure S77. *¹H and ¹³C NMR spectra of compound **64** in CDCl₃.*

(2*R*)-2-((*S*)-hydroxy(phenyl)methyl)tetrahydro-2*H*-thiopyran 1-oxide 65

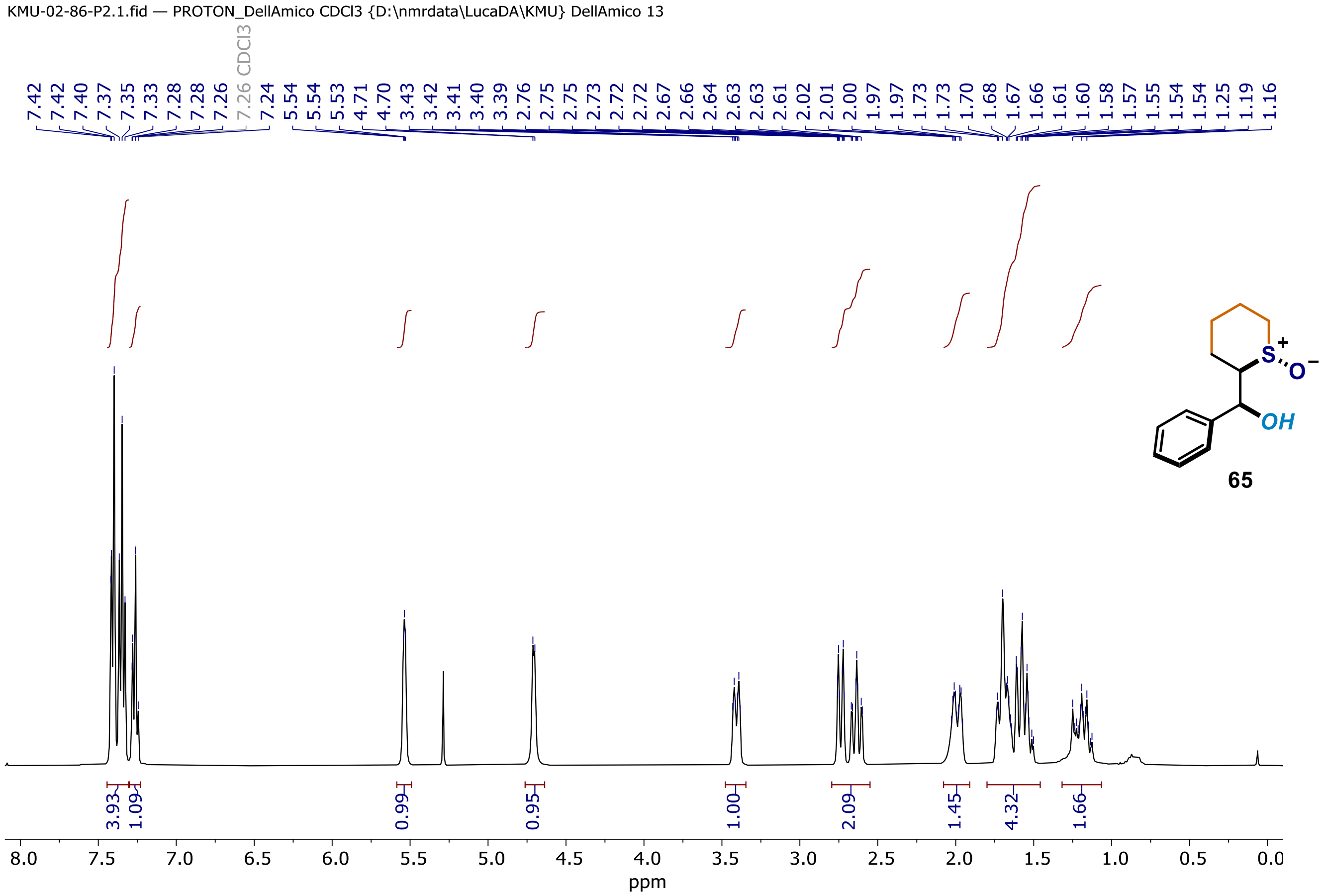

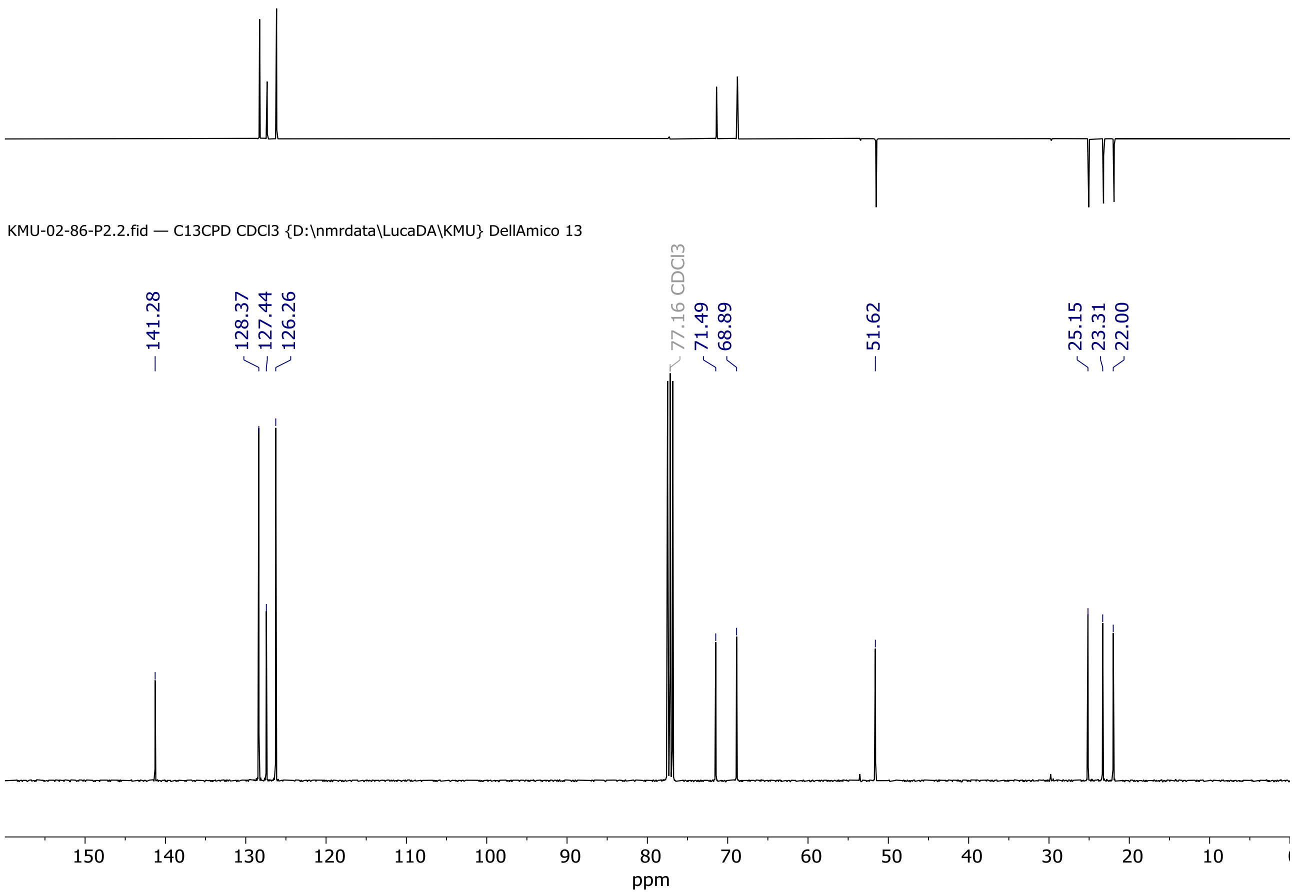

Figure S78. *^{1}H and ^{13}C NMR spectra of compound* ***65*** *in $CDCl_3$.*

(*R*)-2-((*S*)-hydroxy(phenyl)methyl)tetrahydro-2*H*-thiopyran 1,1-dioxide 66

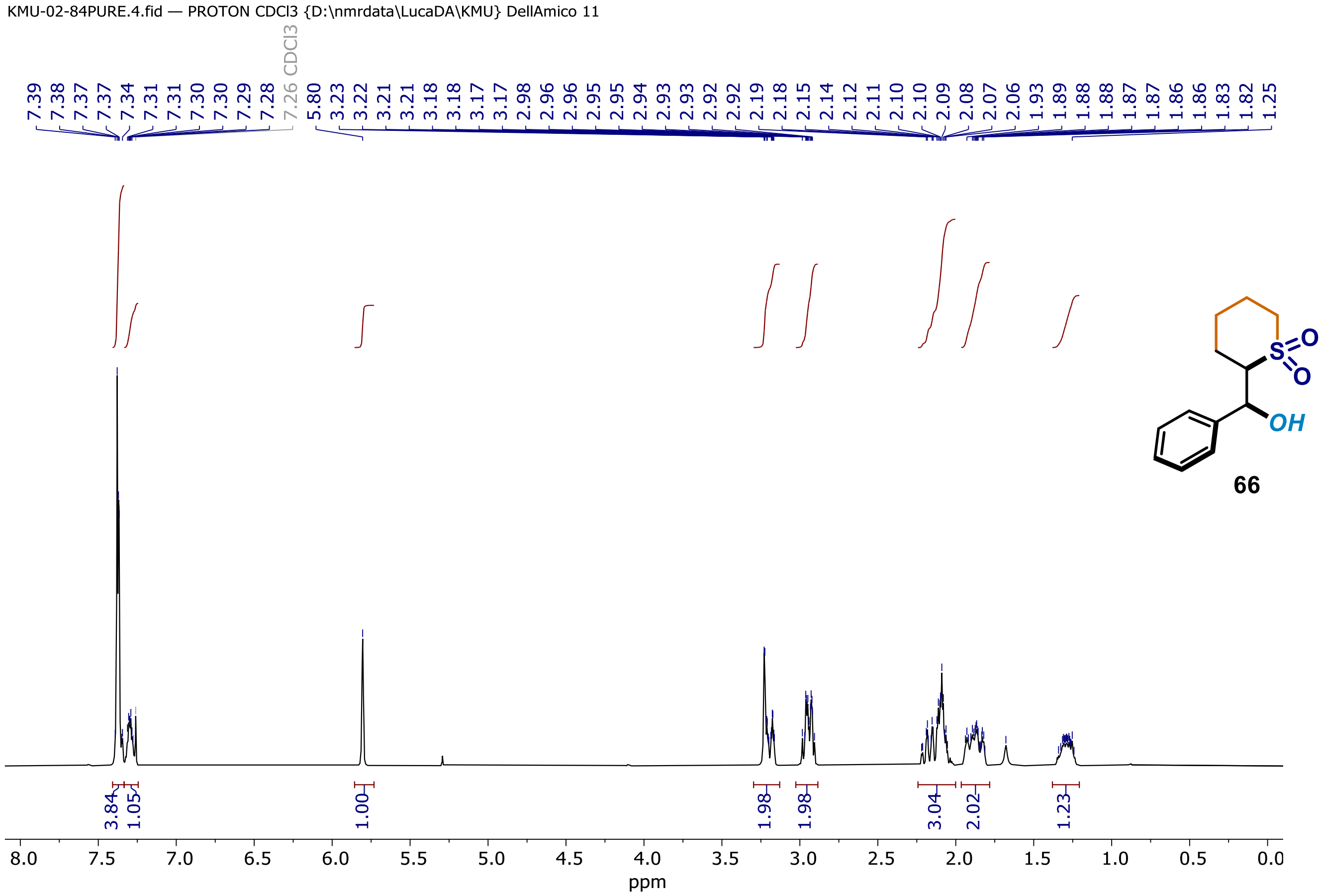

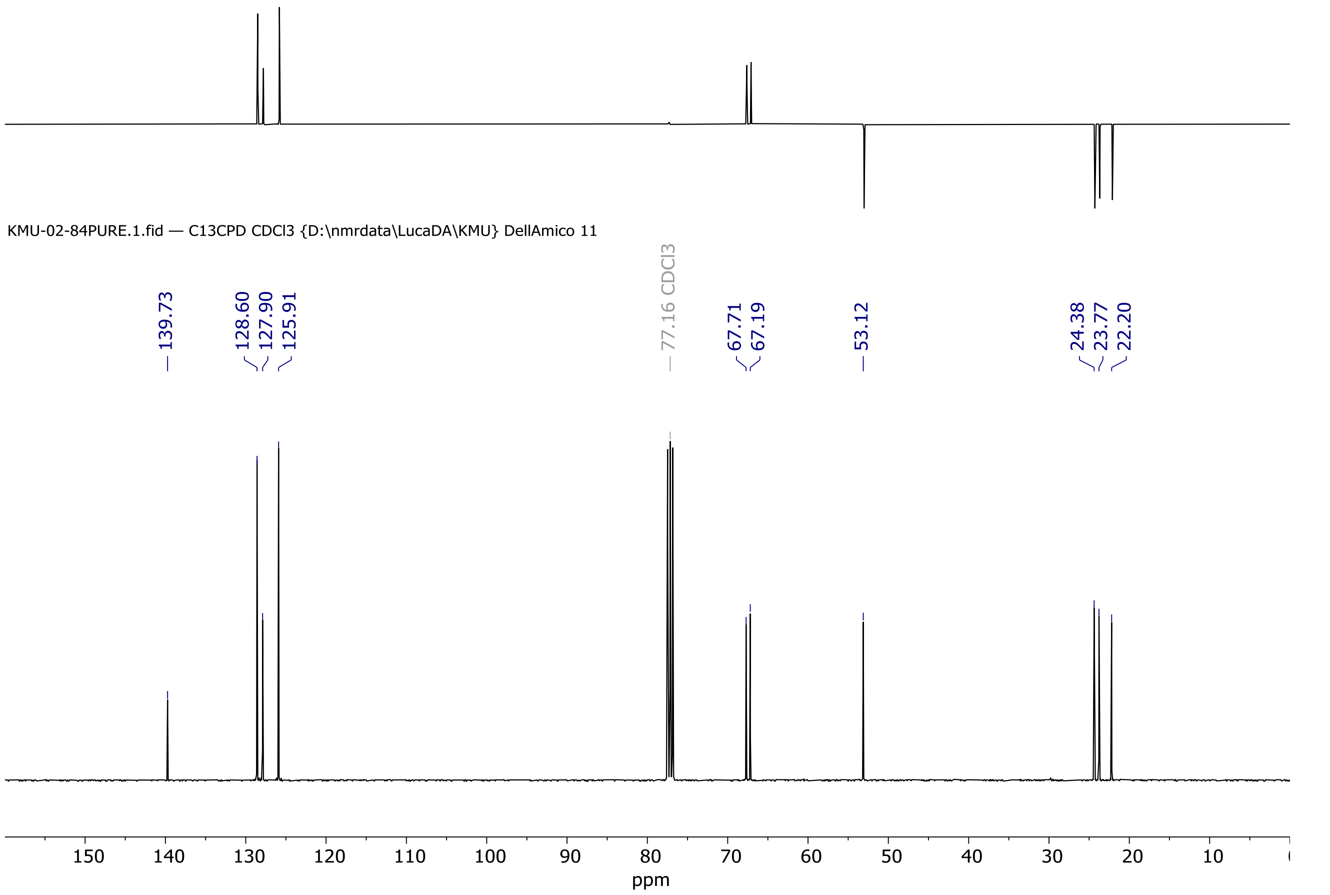

Figure S79. *^{1}H and ^{13}C NMR spectra of compound **66** in $CDCl_3$.*

7-phenyl-2,3,4,5-tetrahydrothiepine 1,1-dioxide 67

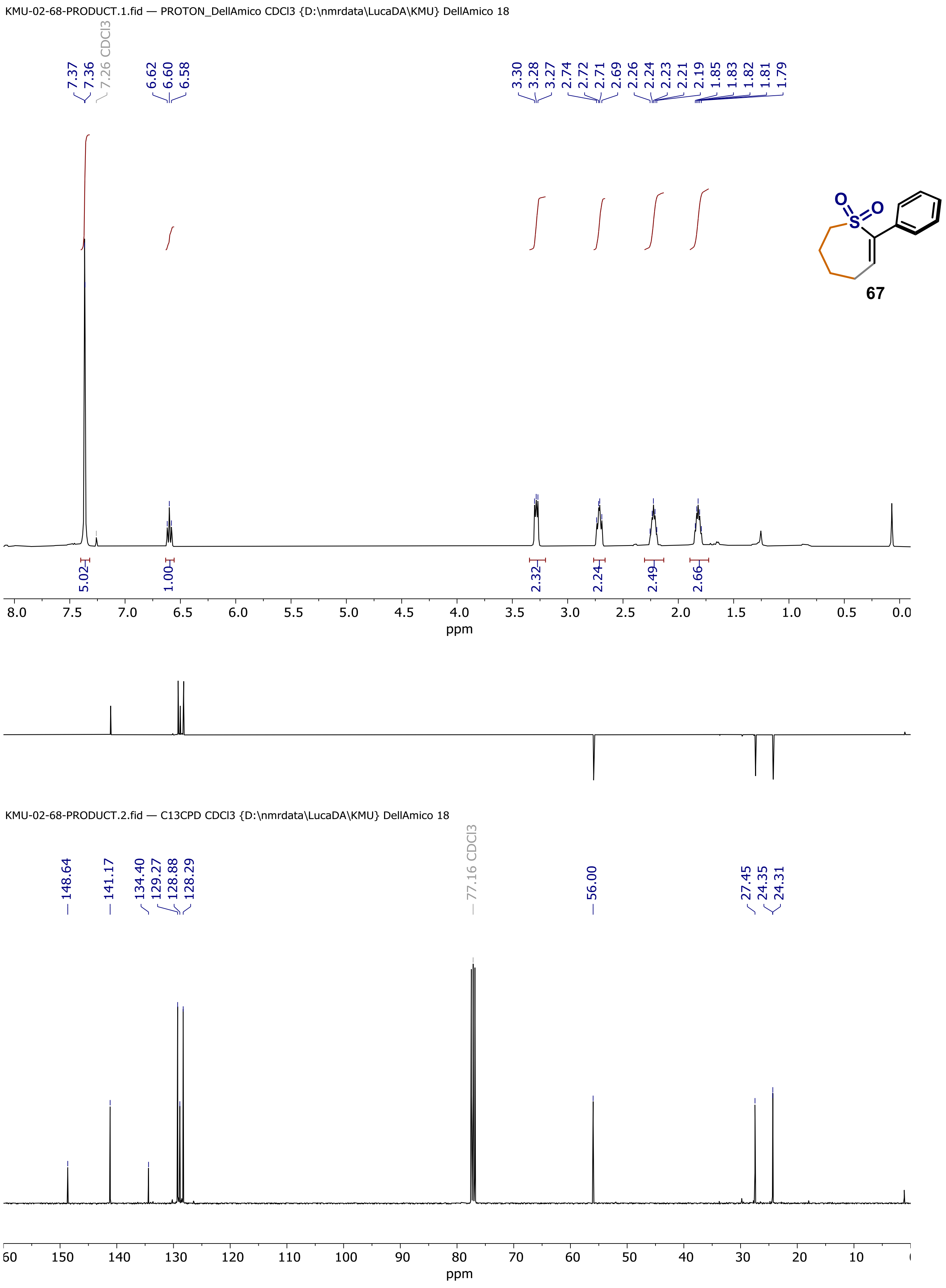

Figure S80. *^1H and ^{13}C NMR spectra of compound **67** in $CDCl_3$.*

(5a*R*,5b*R*,10a*R*,10b*R*)-10a,10b-di(naphthalen-2-yl)dodecahydrocyclobuta[1,2-b:4,3-b']bis(thiepine)68

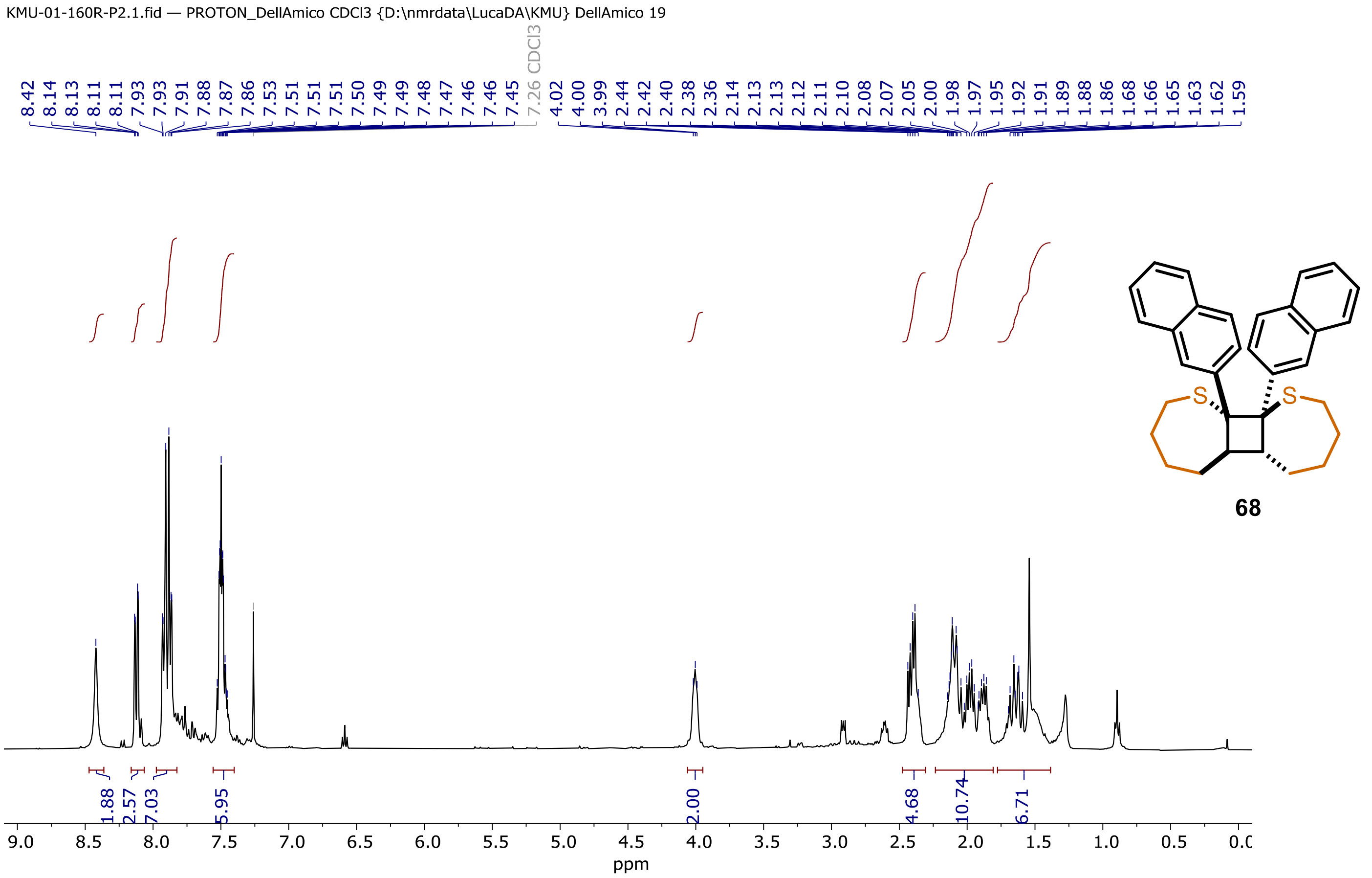

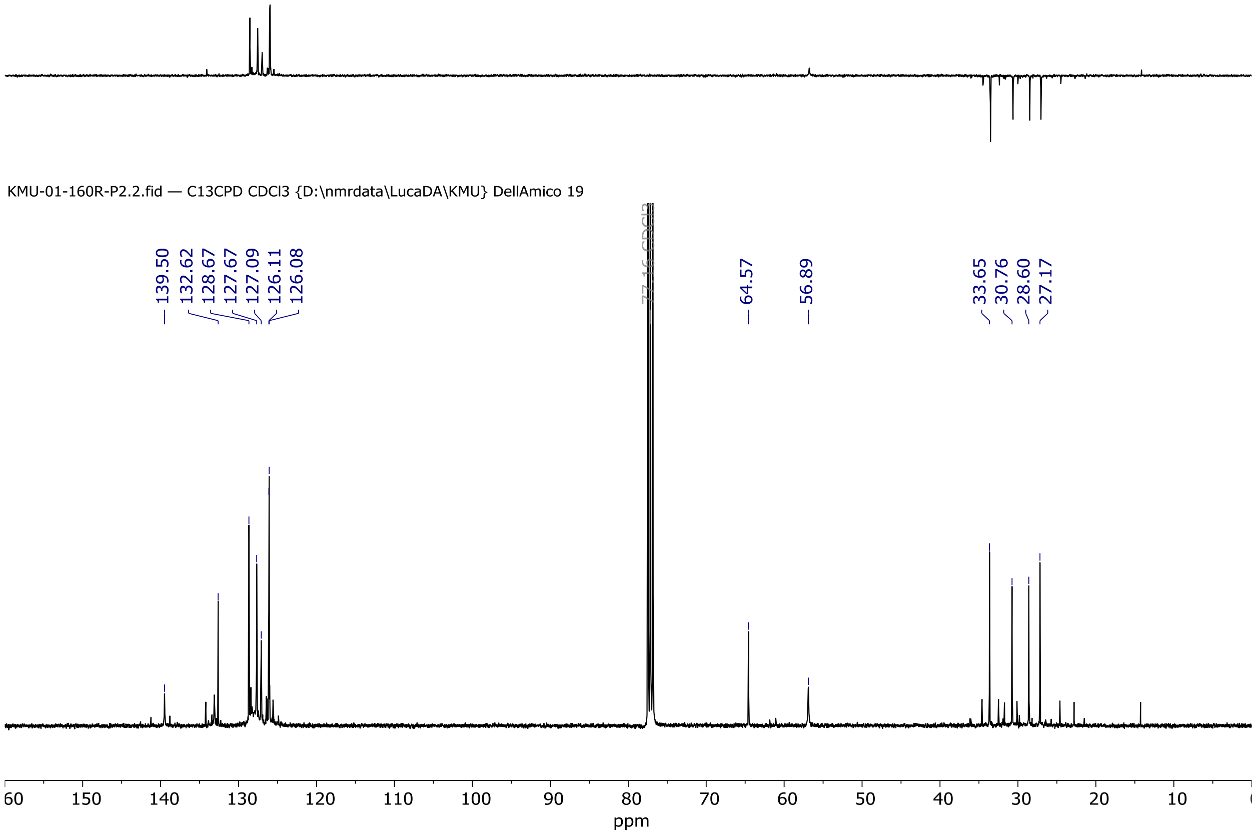

Figure S81. *^{1}H and ^{13}C NMR spectra of compound **68** in $CDCl_3$.*
